# Screening of a Library of Oligosaccharides Targeting Lectin LecB of *Pseudomonas Aeruginosa* and Synthesis of High Affinity Oligoglycoclusters

**DOI:** 10.3390/molecules23123073

**Published:** 2018-11-24

**Authors:** Lucie Dupin, Mathieu Noël, Silvère Bonnet, Albert Meyer, Thomas Géhin, Ludovic Bastide, Mialy Randriantsoa, Eliane Souteyrand, Claire Cottin, Gérard Vergoten, Jean-Jacques Vasseur, François Morvan, Yann Chevolot, Benoît Darblade

**Affiliations:** 1 Ecole Centrale de Lyon, UMR 5270 CNRS, Institut des Nanotechnologies de Lyon, Université de Lyon, 36 Avenue Guy de Collongue, 69134 Ecully CEDEX, France; dupin.lucie26@gmail.com (L.D.); thomas.gehin@ec-lyon.fr (T.G.); eliane.souteyrand@orange.fr (E.S.); 2 IBMM, Université Montpellier, CNRS, ENSCM, 34095 Montpellier, France; mathieu.noel@umontpellier.fr (M.N.); albert.meyer@umontpellier.fr (A.M.); jean-jacques.vasseur@umontpellier.fr (J.-J.V.); 3 Elicityl SA, 746 avenue Ambroise Croizat, 38920 Crolles, France; silvere.bonnet@elicityl.fr (S.B.); ludovic.bastide@elicityl.fr (L.B.); mialy.randriantsoa@elicityl.fr (M.R.); claire.cottin@elicityl.fr (C.C.); benoit.darblade@elicityl.fr (B.D.); 4 Unité de Glycobiologie Structurale et Fonctionnelle (UGSF) - UMR 8576 CNRS - Université de Lille 1, Cité Scientifique, Avenue Mendeleiev, Bat C9, 59655 Villeneuve d’Ascq CEDEX, France; gerard.vergoten@univ-lille1.fr

**Keywords:** oligosaccharide, glycocluster, *Pseudomonas aeruginosa*, anti-bacterial, glycoconjugate

## Abstract

The Gram negative bacterium *Pseudomonas aeruginosa* (PA) is an opportunistic bacterium that causes severe and chronic infection of immune-depressed patients. It has the ability to form a biofilm that gives a selective advantage to the bacteria with respect to antibiotherapy and host defenses. Herein, we have focused on the tetrameric soluble lectin which is involved in bacterium adherence to host cells, biofilm formation, and cytotoxicity. It binds to l-fucose, d-mannose and glycan exposing terminal fucose or mannose. Using a competitive assay on microarray, 156 oligosaccharides and polysaccharides issued from fermentation or from the biomass were screened toward their affinity to LecB. Next, the five best ligands (Lewis^a^, Lewis^b^, Lewis^x^, siayl-Lewis^x^ and 3-fucosyllactose) were derivatized with a propargyl aglycon allowing the synthesis of 25 trivalent, 25 tetravalent and 5 monovalent constructions thanks to copper catalyzed azide alkyne cycloaddition. The 55 clusters were immobilized by DNA Directed immobilization leading to the fabrication of a glycocluster microarray. Their binding to LecB was studied. Multivalency improved the binding to LecB. The binding structure relationship of the clusters is mainly influenced by the carbohydrate residues. Molecular simulations indicated that the simultaneous contact of both binding sites of monomer A and D seems to be energetically possible.

## 1. Introduction

The emergence of antibiotic-resistant bacterial strains calls for new anti-bacterial strategies. One of them consists of inhibiting virulent factors in particular lectins involved in bacterial adhesion and/or biofilm formation using monovalent glycomimetics [1] or multivalent glycoclusters. Lectins generally display multiple carbohydrate recognition domains (CRD) and high avidity ligands can be obtained through multivalent contact thanks to the glycocluster effect [2,3,4,5]. The extent of the glycoside effect depends on both the structure of the CRDs and the topology of the glycocluster.

*Pseudomonas aeruginosa* (PA) is a Gram-negative opportunistic bacterium involved in severe infections of the respiratory and/or urinary tracts, skin and eyes [6]. It is one of the most prevalent nosocomial bacterial pathogens [7,8] and it is the major cause of cystic fibrosis patient mortality. The threat of PA infection stems from its ability to develop antibiotic resistance and to protect itself by forming a biofilm leading to chronic inflammation and eventually to death, despite aggressive antibiotic therapy. One strategy consists of targeting PA virulence factors in order to reduce its aggressiveness and/or to increase the efficacy of the host immune system for pathogen clearance. Virulence factors are molecules (secreted or surface-bound) that trigger bacterial adherence to tissue, biofilm formation or mitigate host defenses [9].

The soluble homotetrameric fucophilic lectin LecB of PA is classified as a virulent factor and is involved in biofilm formation, host/bacteria and bacteria/bacteria interaction, cytotoxicity and inhibition of ciliated removal [10]. It consists of four subunits of 11.7 kDa (114 amino-acids). Two calcium atoms are structurally present in the binding sites. These two calcium atoms are coordinated by the fucose upon binding, and they are responsible for the unusual sub-micromolar dissociation constant observed for a monomeric ligand [11]. Indeed, dissociation constants (*K*_d_) for FucOMe are 0.43 µM for LecB from PAO1 [12,13], 2.2 µM for LecB from PA7 [14], and 0.2 µM for LecB from PA14 [12]. Furthermore, *N*-fucosyl amide derivatives showed *K*_d_ values between 1.2 to 2.1 µM [15]. To a lesser extent, it also displays binding to l-galactose, d-arabinose and d-mannose [11]. d-galactose is not recognized. Cinnamide and sulfonamide derivatives of mannose were found to be inhibitors of LecB with a *K*_d_ value of 18.5 and 3.3 µM respectively [16]. More complex glycans are also bound, provided that terminal fucoses or mannoses are available. Binding to Lewis^a^ (Le^a^) [17], sialyl-Lewis^a^ [18], antigen H [19], 3-fucosyllactose [17], LNFP-II, LNnFP-V, Lewis^x^ (Le^x^) [20], sialyl-Lewis^x^ [18] and mannan [21] have been reported. However, slight differences between different isolates were observed. Lewis^a^ had dissociation constants of 70, 210 and 2003 nM for LecB issued from PA14 [12], PAO1 [20] and PA7 [14], respectively. Sialylation of Lewis^a^ seems to only slightly affect the binding to PAO1-LecB and does not affect at all the binding of PA7-LecB [14].

Aiming at inhibiting LecB, synthetic efforts have been devoted to the synthesis of Lewis^a^ mimics and multivalent ligands. Indeed, it was demonstrated that the dimer αFuc-1-4GlcNAc, a truncated version of Lewis^a^, also displayed a high affinity for LecB (290–310 nM) [22]. Therefore, heterocycle α-L-fucoside ligands mimicking the dimer were synthesized and screened toward LecB. An isoxazole derivative was found to be as potent as Lewis^a^ [22].

Alternatively, multivalent ligands can take advantage of the so-called glycocluster effect [2] to reach high affinities. Various scaffolds have been studied, such as oligoethylene [23], penta-erythritylphosphodiester [24,25], cyclopeptide [26], carbohydrate-centered ligands [25,27,28], peptide [29,30], calix [4] arene [31], fullerene [32], Pillar [5] arene [33] and photo-switchable Janus dendrimer. [34]. To date, the highest affinity reached was less than 30 nM using an hexadecavalent fucose cluster [29]. To the best of our knowledge, these multivalent molecules only contain fucose. However, the synthesis of multivalent oligosaccharide ligands taking advantage of both multivalency and the high affinity of LecB for several oligosaccharides has only been described by Marotte et al. They reported the synthesis of divalent and trivalent clusters of Fuc-1-4GlcNAc and reached dissociation constants in the 100 nM range [23]. A 2:1 LecB/ligand stoichiometry was measured by ITC suggesting that a divalent interaction occurred.

Herein, with the aim to find oligosaccharide of high affinity to LecB and then to synthesize oligosaccharide clusters of higher affinity to LecB that could compete with the natural endogenous ligands of the lectin, we developed a double screening strategy. First, 156 natural oligosaccharides exhibiting different carbohydrate compositions and degree of polymerization were qualitatively classified thanks to a competitive assay on microarray. Then, the best epitopes were introduced on different scaffolds leading to oligoglycoclusters exhibiting different topologies and valences (1, 3 or 4). To perform their screening, the oligoglycoclusters were conjugated to a DNA sequence allowing their immobilization by DNA directed immobilization (DDI) on a DNA array. The resulting glycocluster array was used to assess the affinity of the oligoglycoclusters to LecB by a direct readout of the fluorescence signal in comparison to the corresponding monovalent ligand.

## 2. Results and Discussion

**Figure 1:**
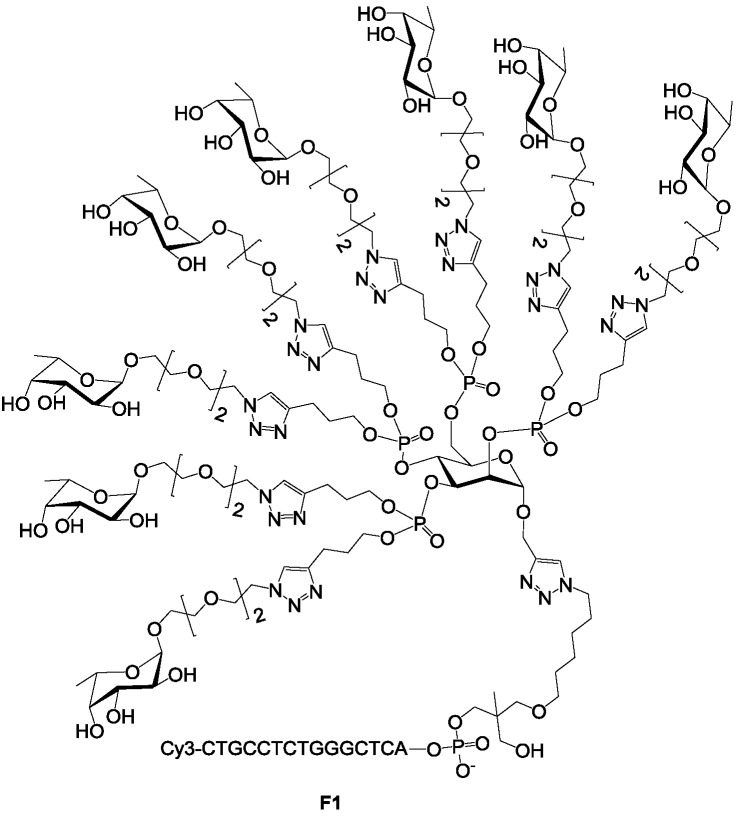
Structure of the fucosyl cluster **F1** used for the first screening.

We have previously reported a microarray-based IC_50_ assay to screen the binding of immobilized fucosylated clusters to LecB using fucose as a competitor [27]. A similar assay can be used to probe the binding of the 156 sugar candidates (see supporting information S1). To this end, we selected a Cy3-oligonucleotide-fucosylated cluster exhibiting eight fucoses **F1** (Figure 1). The IC_50_ value of **F1** with respect to fucose, used as inhibitor, is 16 µM [27]. This IC_50_ value corresponds to the concentration of fucose required to reduce 50% of LecB interaction with the glycocluster. 

**Figure 2:**
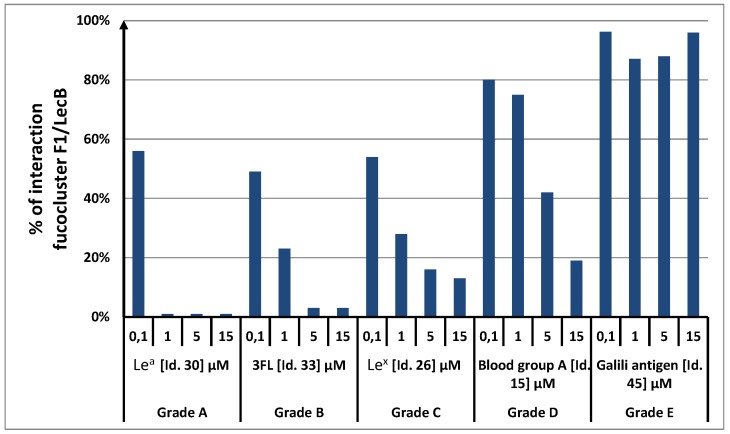
Examples of results obtained for five natural oligosaccharides. The reference fucocluster was **F1**. On the y-axis, the fluorescent signal recorded after incubation of **F1** with LecB and 0.1 µM of fucose was set at 100%. All other fluorescent signals were then normalized to that reference. (Le^a^: Lewis^a^ tetraose, Galβ1-3(Fucα1-4)GlcNAcβ1-3Gal; 3FL: 3-Fucosyllactose, Galβ1-4(Fucα1-3)Glc; Le^x^: Lewis^x^ triose, Galβ1-4( Fucα1-3)GlcNAcβ1-3Gal; Blood group A: A trisaccharide antigen, GalNAcα1-3(Fucα1-2)Gal; Galili antigen: Galili pentasaccharide, Galα1-3(Galβ1-4GlcNAcβ1-3)_2_Galβ1-4Glc); see supporting information S1).

**F1** was immobilized on a DNA chip, thanks to its DNA sequence. The distance between two **F1** on the surface was estimated to be nearly 50 nm [35]. Therefore, the lectin LecB can only bind to one **F1** glycocluster at the time. The binding of immobilized **F1** to LecB was competed with each of the 156 glycans at four different concentrations (0.1, 1.0, 5.0 and 15 µM). For each glycan and each concentration, the alexa 647-LecB fluorescent signal was recorded and averaged over 16 spots, leading to four-point competition histograms (Figure 2 and Figure S2). 100% signal (see Figure 2) corresponded to the fluorescent signal measured with a reference assay under which no competition could occur. Glycans able to compete with **F1** to bind LecB lead to a decrease of the fluorescent signal. Therefore, for a given glycan concentration, strong binding glycans lead to stronger decrease of the fluorescent signal so to lower percentages. Accordingly, tested glycans were classified qualitatively into 5 groups (A, B, C, D and E) depending to their ability to reduce the measured fluorescent signal at the four different concentrations (Figure 2 and Table 1). Grade A are stronger competitor than grade B which are stronger than grade C, and so on. A Grade A was given for oligosaccharides that were able to reduce 80 to 40% of the fluorescent signal at a concentration of 0.1 µM and to reduce it by more than 90% at concentrations of 1, 5 and 15 µM. Grade B indicated a decrease of fluorescence by 70% to 90% at 1 µM and more than 90% at 5 and 15 µM. Grade C corresponded to a decrease of 50 to 70% for 1 µM, around 80% for 5 µM and more than 90% at 15 µM. D indicated a decrease of less than 50% for 1 µM and 80% at 5 and 15 µM. When no inhibition was observed for the four concentrations tested, the grade was E.

Such a screening has two main advantages: The oligosaccharides do not need to be derivatized for their immobilization and their surface densities do not have to be taken into consideration, as they remain in solution. Indeed, as underlined by several authors [36,37], the binding of lectins with immobilized carbohydrates depends on their intrinsic affinity, but also on their surface density. Indeed, high surface densities can result in multiple contacts and, similar to a cluster effect, can increase the measured avidity (surface cluster effect). 

In our case, the surface density of **F1** cannot result in a surface cluster effect [35] and its surface density is similar for each assay (535 nm fluorescent issued from surface-bound **F1** deviated by less than 15%). Furthermore, the competing oligosaccharides are in solution. Therefore, the classified binding is solely related to the intrinsic binding of the oligosaccharides with LecB.

**Figure 3:**
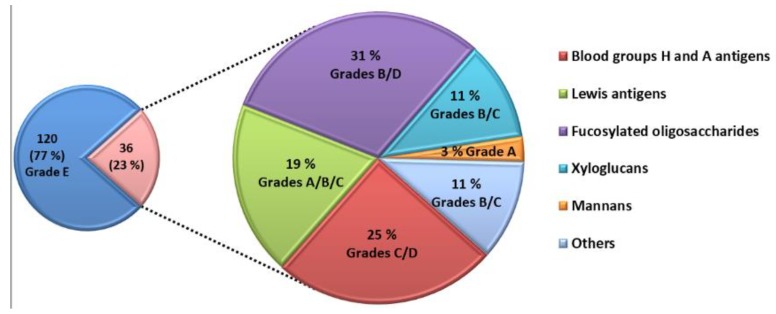
Overview of the screening of the 156 glycans. 36 competed for the interaction of LecB with the immobilized fucocluster F1.

The competitive assay was able to identify 36 natural glycans interacting with LecB (Figure 3). The majority of these glycans belong to the families of A and H blood group antigens, Lewis antigens and fucosylated glycans. They were produced by bacterial fermentation. Five glycans interacting with LecB are extracted from biomass such as xyloglucans and mannans.

Table S2 (Supporting Information) gives the structure and the grade obtained for each glycan interacting with LecB. Several parameters seem to drive this interaction. All glycans, except the mannan (Id. 151), contain at least one terminal fucose residue at their non-reducing end or on their side chain, which allows the interaction with LecB. The interaction of the mannan polysaccharide with LecB is mediated by d-mannose on its side chain as suggested by Zinger-Yosovich [21]. Indeed, LecB is able to interact with both monosaccharide, l-fucose and d-mannose. Despite the fact that the interaction of LecB with the d-mannose was described to be 25 times weaker than the one with the l-fucose, the mannan obtained grade A [17]. Furthermore, oligosaccharides containing one fucose on their side chain with an α1-2 glycosidic bond obtained the grade of D or C, the ones with an α1-3glycosidic bond, the grade of C or B, and finally, those with an α1-4 glycosidic bond, the grade of A. Therefore, LecB seems to preferentially bind Fucα1-4 > Fucα1-3 > Fucα1-2 oligosaccharides. Then, the presence of a Fucα1-2 close to the non-reducing end of the oligosaccharide seems to disturb the interaction between Fucα1-3/4 and LecB. For example, Galβ1-3(Fucα1-4)GlcNAcβ1-3Gal (Grade A, Id.30) interacted more strongly with LecB than Fucα1-2Galβ1-3(Fucα1-4)GlcNAcβ1-3Gal (Grade C, Id. 32), despite the fact that two fucose motifs are present in the latter structure. Finally, as described in the literature, LecB preferentially interacts with Lewis^a^ antigens (Grade A, Id. 30 and 31) and 3-Fucosyllactose (3FL) oligosaccharides (Grade B, Id. 33 to 37) [18,38]. The co-cristal LecB/Lewis^a^ (Galβ1-3(Fucα1-4)GlcNAc) showed that all monosaccharides of Lewis^a^ interact with the surface of LecB. The authors have shown that, in addition to a network of hydrogen bonds between fucose and LecB, the GlcNAc residue makes an hydrogen bond between its *O*-6 atom and the Asp96 of LecB, and that both GlcNAc and Gal residues establish additional interactions with LecB through the bridging of water molecules [20]. In comparison to Lewis^a^ (Grade A, Id. 30), Lewis^x^ antigen displays a weaker binding for LecB (Grade C, Id. 26). Perret et al. have also shown by molecular simulation that in the case of Lewis^x^ antigen, *N*-acetyl moiety of the glucose was at the same position as the *O*-6 atom of GlcNAc of Lewis^a^ antigen, which seems to hinder the additional hydrogen bond with Asp96 of LecB [20]. The 3FL oligosaccharide (Id. 33) differs from Lewis^x^ antigen by the absence of the *N*-acetyl modification on the glucose, and a stronger affinity for LecB is observed. It suggests that steric hindrance is lower for 3FL oligosaccharide than for Lewis^x^ antigen.

Five oligosaccharides with various grades were selected for the synthesis of oligoglycoclusters and to address the effect of clustering on their binding with LecB (Table 1). For each family, the oligosaccharide with a lower polymerization degree was chosen.

**Figure 4:**
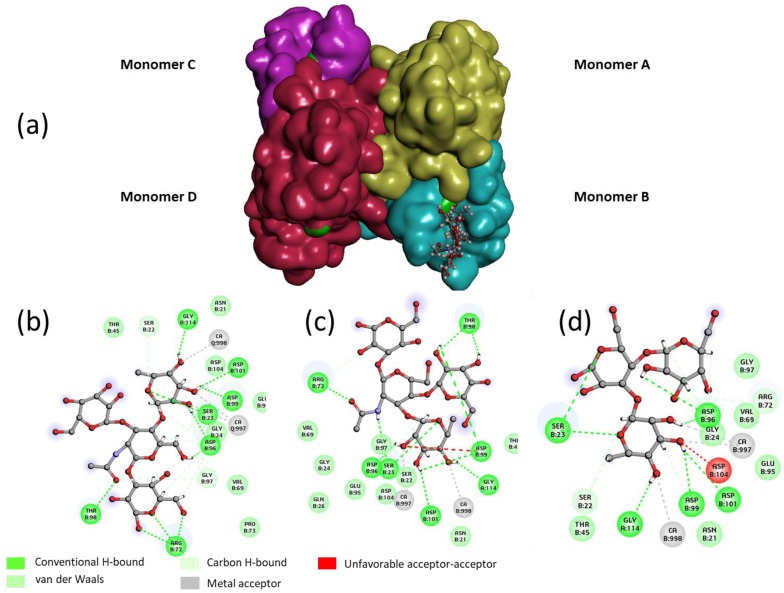
(**a**) Lewis^a^ interaction with Lectin LecB tetramer (calcium ions in green). 2D diagram (Biovia Discovery Studio 2016) of the interaction of LecB tetramer with (**b**) Lewis^a^ tetrasaccharide, (**c**) Lewis^x^ tetrasaccharide and (**d**) 3-Fucosyllactose (3FL).

In order to rationalize the binding of Lewis^a^ tetrasaccharide, Lewis^x^ tetrasaccharide and 3-Fucosyl lactose (as examples for each grade), a docking study was performed with the tetrameric LecB (Figure 4). An empirical calculation of the potential energy of interaction (ΔE) between LecB and Lewis^a^ (Id. 30), Lewis^x^ (id 26) and 3-Fucosyllactose (Id. 33) [25] gave a value of −98, −81 and −67 kcal/mol respectively. Similarly to the screening on microarray and to the published results of Perret [20], Lewis^a^ was found as the most potent ligand for LecB.

For the three oligosaccharides, the coordination involves the OH at position 2, 3 and 4 of the fucose as hydrogen bond (hb) donors and the oxygen of the ring as an hb acceptor (see Table 2). The O_1_, O_ring_ and O_6_ of the galactose residue of the non-reducing are involved in the binding as hb acceptors. In the case of the Lewis^x^ and 3FL, only the OH at the 2 position seems to be involved in an interaction with the lectin, suggesting that the stabilization of the complex between the Lewis^a^/LecB versus Lewis^x^/LecB or 3FL/LecB is mainly due to the three contributions of the non-reducing galactose of Lewis^a^ (Table 2 and Figure 4).

For the synthesis of oligoglycoclusters, six different centers were used as scaffolds: Three pentoses (arabinose, xylose and ribose) and three hexoses (glucose, galactose and mannose). These different cores allowed the effect of valence (trivalent for Ara, Xylo and Ribo vs. tetravalent for Glc, Gal and Man) and topology to be explored. Furthermore, two linkers, corresponding to diethyleneglycol and tetraethyleneglycol, were used to study the impact of the length between the core and the oligosaccharides on the affinity to LecB. The oligosaccharide cluster-oligonucleotide conjugates were synthesized by a combination of phosphoramidite chemistry and Cu(I) catalyzed azide alkyne cycloaddition (CuAAC) [39]. The oligoglycoclusters were tagged with a Cy3-DNA sequence and immobilize on a DNA chip by duplex formation with their immobilized complementary sequence. Their affinity for LecB was then evaluated.

**Figure 5:**
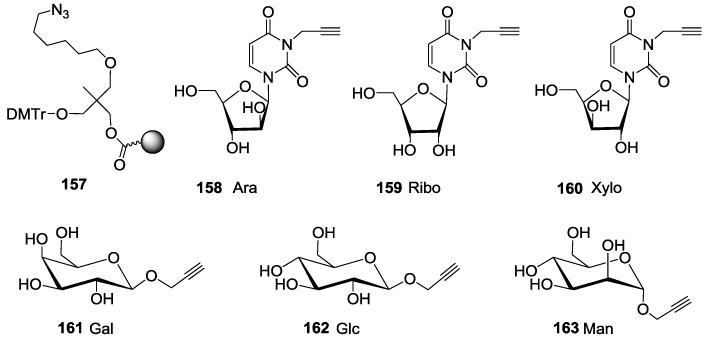
Structure of azide solid support **157** *N^3^*-propargyl furanoside uracyl **158**–**160** and propargyl pyranoside **161**–**163** scaffolds.

**Scheme 1:**
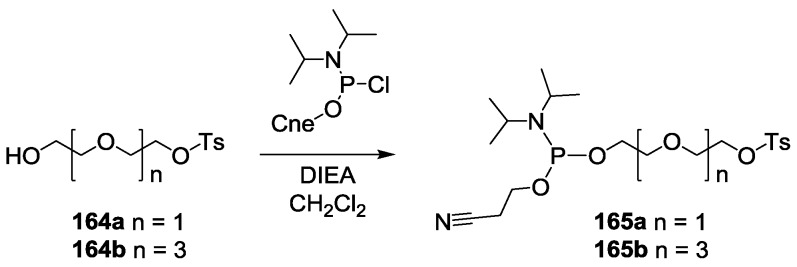
Synthesis of tosyl di- and tetra-ethyleneglycyl (2-cyanoethyl) *N,N*-diisopropyl phosphoramidites **165a**–**b**. Cne: 2-cyano-ethyl.

**Scheme 2:**
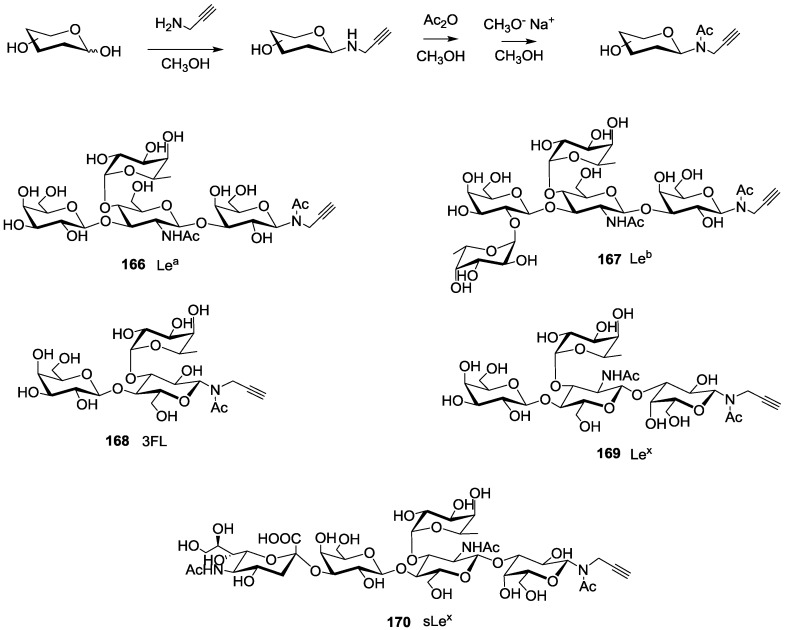
Synthesis of propargylated *N*-acetyl oligosaccharides **166**–**170**. Le^a^: Lewis^a^ tetrasaccharide, Le^b^: Lewis^b^ tetrasaccharide, 3FL: 3-Fucosyllactose, Le^x^: Lewis^x^ tetrasaccharide and sLe^x^: sialyl-Lewis^x^ pentasaccharide.

For this purpose, we first prepared the different building blocks corresponding to an azide solid support **157** [40], trihydroxylated alkyne scaffolds prepared from arabino-, ribo- and xylo- furanosyl uracils (**158**–**160**) [28], tetrahydroxylated scaffolds corresponding to propargyl-galactoside, glucoside and mannoside (**161**–**163**) [27] (Figure 5), two different linkers corresponding to tosyl-di- or tetra-ethyleneglycol phosphoramidites (**165a**–**b**) (Scheme 1, see supporting information S3 for NMR characterization) and the five *N*-acetyl-propargyl oligosaccharides **166**–**170** selected from the first screening (Scheme 2, see supporting information S4 for NMR and MALDI-TOF characterizations). Both tosyl-linkers (**165a**–**b**) were obtained by phosphitylation of di- and tetra-ethyleneglycols (**164a**–**b**). The five selected oligosaccharides were converted as alkyne derivatives by means of a treatment with an excess of propargylamine in methanol to afford the resulting glycosylamines. Then the nitrogen was *N*-acetylated using acetic anhydride to avoid any further hydrolysis [41].

**Scheme 3:**
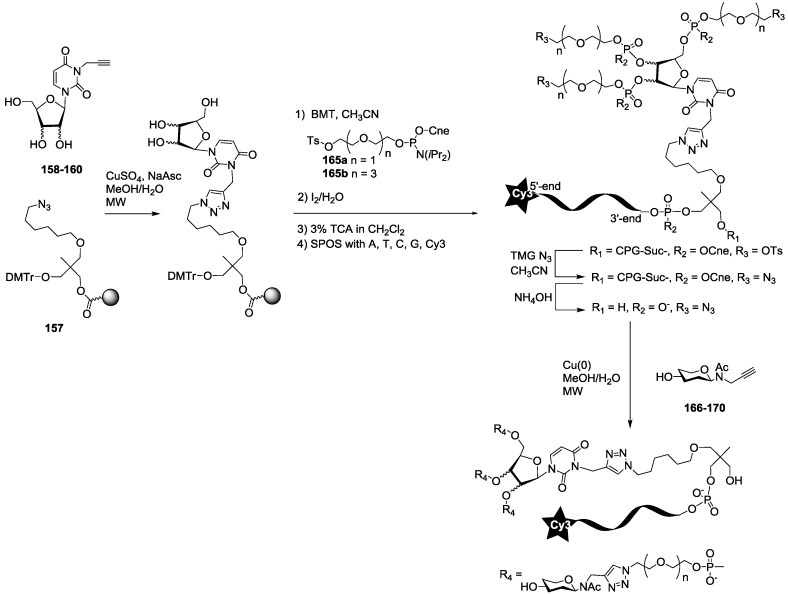
Synthesis of uracylfuranoside-centered oligo-saccharide cluster oligonucleotide conjugates (**G1** to **G6** series) SPOS Solid-phase oligonucleotide synthesis, Cne: Cyanoethyl, grey ball represents CPG: Controlled pore glass.

**Scheme 4:**
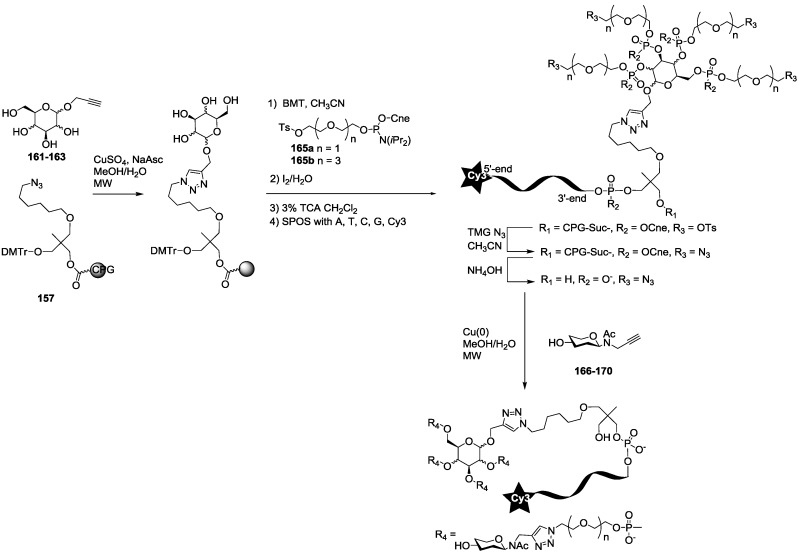
Synthesis of pyranoside-centered oligosaccharide cluster oligonucleotide conjugates (**G7** to **G12** series). SPOS Solid-phase oligonucleotide synthesis, Cne: Cyanoethyl, grey ball represents CPG: Controlled pore glass.

The synthesis of the polyazide scaffold oligonucleotide conjugates was performed on a solid support allowing their efficient, automated and rapid synthesis at about 200 to 300 nmol scale. Finally, the conjugation with the five oligosaccharides **166**–**170** was performed in solution allowing a divergent strategy, since only 20 nmol of scaffold were used for each. Thus, the synthesis required six steps (Scheme 3 and Scheme 4). Firstly, polyhydroxylated scaffolds (**2**–**7**) were immobilized on the azide solid support **157** by CuAAC. Secondly, each free hydroxyl was phosphorylated with tosyl-di- or tetra-ethylene glycol phosphoramidites (**165a**–**b**). Thirdly, the DNA sequence was elongated and labelled with Cy3 fluorescent dye. Fourthly, tosyl groups were substituted by azide using tetramethylguanidine azide. Finally, an ammonia treatment afforded the Cy3-oligonucleotides bearing a tri- or tetra-azidolinker scaffold. After HPLC purification on a C_18_ reverse-phase column, each construction was aliquoted to 20 nmol. The final CuAAC was carried out on a 20 nmol scale with the different *N*-acetyl-propargyl oligosaccharides **166**–**170** affording the expected oligosaccharide cluster-Cy3-oligonucleotide conjugates **G1**–**G12**. 

Each of them was analyzed by HPLC and characterized by MALDI-TOF mass spectrometry (See Supporting Information S5). As a result, 12 oligoglycocluster-Cy3-oligonucleotides exhibiting three or four oligoglycosides with di- or tetra-ethyleneglycol arms were obtained for Lewis^a^ tetrasaccharide, Lewis^b^ tetrasaccharide, Lewis^x^ tetrasaccharide and 3FL, while for sialyl-Lewis^x^ pentasaccharide only two oligoglycocluster-oligonucleotides built from ribose and mannose core with tetra-ethyleneglycol arms were synthesized.

**Scheme 5:**
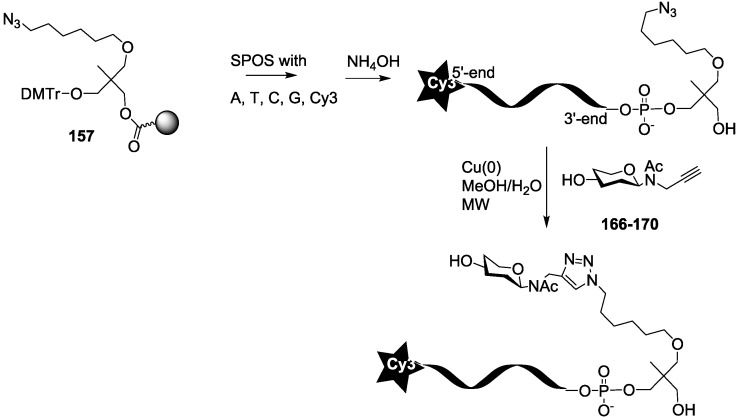
Synthesis of oligosaccharide-oligonucleotide conjugates (M-series).

In order to evaluate the effect of multivalence of the oligosaccharide clusters, we also conjugated each oligosaccharide **166**–**170** only once on an azide Cy3-oligonucleotide (Scheme 5). The Cy3 oligonucleotide was synthesized from the azide solid support **157** by standard phosphoramidite chemistry, and the resulting monoazide oligonucleotide was conjugated in solution as described above with the different *N*-acetyl-propargyl oligosaccharides **166**–**170** affording the mono-oligoglyco-oligonucleotides (M-series).

**Figure 6:**
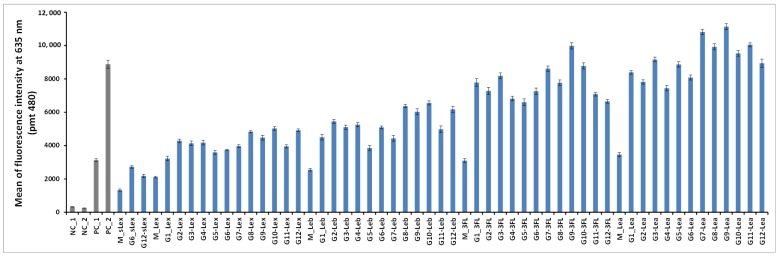
The fluorescent signal of Alexa 647 LecB conjugate was recorded as a function of the oligoglycoclusters. NC and PC stand for negative and positive control. Structures of NC_2, PC_1 and PC_2 are displayed Figure 7. NC_1 is a single stranded DNA. **G1** and **G2** are arabinose-centered clusters with an EG_2_ or EG_4_ linker respectively. In a similar manner, **G3** and **G4** are xylose-centered, **G5** and **G6** ribose-centered, **G7** and **G8** glucose-centered, **G9** and **G10** galactose-centered, and finally **G11** and **G12** mannose-centered. Average and error bars were calculated from 32 repetition spots from one experiment.

The 50 oligoglycoclusters were tested for their binding toward LecB (Figure 6) displaying Lewis^a^ tetrasaccharide (G1-Le^a^ to G12-Le^a^), 3-Fucosyllactose (G1-3FL to G12-3FL), Lewis^x^ tetrasaccharide (G1-Le^x^ to G12-Le^x^), Lewis^b^ tetrasaccharide (G1-Le^b^ to G12-Le^b^) and sialyl-Lewis^x^ pentasaccharide (G6-sLe^x^ and G12-sLe^x^), as well as the five monovalent oligosaccharides (M-Le^a^, M-3FL, M-Le^x^, M-Le^b^ and M-sLe^x^). For a given carbohydrate, oligoglycoclusters varied by their linkers (EG_2_ or EG_4_), their valences (3 or 4) and their topologies. 

**Figure 7:**
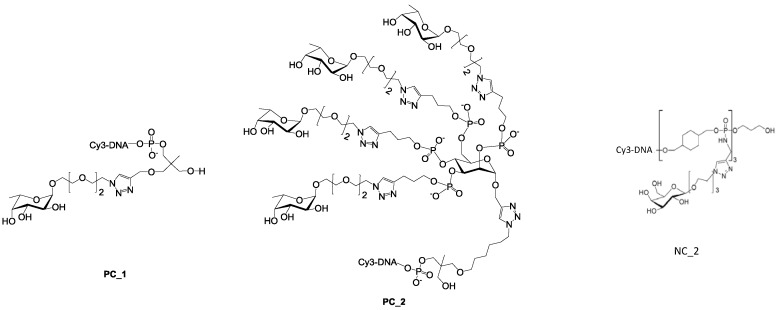
Structure of the two positive controls PC_1 and PC_2 [27] and the negative control NC_2. [42].

For the latest parameter, the topology of oligoglycoclusters was varied using different carbohydrate-centered scaffolds (i.e. pentofuranose: Arabinose, xylose, ribose; hexapyranose: Glucose, galactose, and mannose). Two negative controls, NC_1 and NC_2, corresponded respectively to a single oligonucleotide immobilized through an amide bond and a d-galactocluster immobilized by DDI. Both negative controls exhibited a very low alexa 647 fluorescent signal (below 350 a.u.), illustrating the very low non-specific adsorption of LecB on the surface and its specificity towards fucose moieties. Two positive controls, PC_1 and PC_2, corresponding to a monofucoside (PC_1) and a mannose-centered tetravalent fucoside (PC_2) were used. (Figure 7). Oligoglycocluster immobilization deviated by less than 18%.

According to the Alexa 647 fluorescence signal obtained with monovalent constructions (M-oligosaccharide series), the affinity of LecB increases as follows Le^x^ < Le^x^ < Le^b^ < 3FL < Le^a^. The same trend was observed with the oligoglycoclusters (Figure 6). This ranking corresponds to the one observed with the free oligosaccharides, confirming the relevance of the first screening by competition (Table S2, in Supporting Information).

In all cases, clustering led to an increase of binding (M < G1–12). On average, tetravalent clusters (**G7** to **G12**) gave a slightly higher Alexa 647 fluorescent signal than trivalent clusters (**G1** to **G6**). Furthermore, for each oligosaccharide, the best tetravalent cluster was higher than the best trivalent cluster. However, for each oligosaccharide, the best trivalent cluster was better than some tetravalent clusters. This result shows that the topology seems to be more important than the valence on the binding to LecB, as previously observed with fucoclusters [25].

Concerning the influence of the length of the linker, our study showed that a shorter linker (EG_2_) improves the binding of LecB for oligoglycoclusters bearing Le^a^ tetrasaccharide and 3FL (**G1**, **G3**, **G5**, **G7**, **G9**, **G11**), while the opposite was observed for oligoglycoclusters bearing Le^x^ and Le^b^ tetrasaccharides, respectively. In the case of pentose core (**G1** to **G6**), the binding to LecB is slightly influenced by the type of the furanoside core.

The data shows that the combination of xylose with an EG_2_ linker (**G3**) core is preferred, while with the EG_4_ linker, the arabinose core leads to a higher binding-(**G2**). For hexose core (**G7** to **G12**), whatever the linker, the galactoside core (**G9**–**G10**) gave the highest fluorescent signals, while the mannoside core (**G11**–**G12**) leads to mainly the lowest ones. Strong differences of fluorescent signal were observed between the hexose cores for 3FL and Le^a^ tetrasaccharide.

Previous results on the binding of hexose-centered glycoclusters made with four L-fucose showed that a mannose core was preferred in comparison with the galactose and glucose cores [27]. Therefore, the interaction of a lectin with a set of oligoglycoclusters convolutes the core and the carbohydrate residue, leading to an avidity profile that can be viewed as a fingerprint of the lectin. 

The three best oligosaccharide clusters targeting LecB were found for **G9-Le^a^**, **G7-Le^a^** and **G9-3FL,** all built with an EG_2_ linker and galactoside core for **G9** and glucoside core for **G7**.

In order to ensure the effect of the carbohydrate epitope and the core, the *K*_d_ values of the **G1**, **G3**, **G7** and **G9** series were measured for the two best oligosaccharides, such as Le^a^ and 3FL. As expected, all the oligoglycoclusters exhibited a high affinity for LecB. The differences between the *K*_d_ values of each glycocluster were not significant, around 50 ± 7 nM for Le^a^-clusters and around 60 ± 11 nM for 3FL-clusters. This data shows a cluster effect, whatever the topology of the oligoglycoclusters. So it was not possible to draw further quantitative conclusions according to the shape of the glycoclusters. However, these glycoclusters exhibit a better affinity for LecB than the previously synthesized mannose-centered fucocluster PC_2 (*K*_d_ 153 nM) [25].

Finally, aiming at getting insight on the possible multiple contact between the lectin LecB and the following glycoclusters **G7-3FL**, **G7-Le^a^**, **G9-3FL** and **G9-Le^a^**, have been investigated using docking techniques and molecular simulations (namely Monte Carlo computations), as previously described [25].

An empirical potential energy of interaction ΔE was calculated for the six different possible combinations for the simultaneous binding of the Le^a^ or 3FL epitopes displayed at the tip of two branches attached at the pyranoside core. The mean ΔE (kcal/mol) value among the six is given for each glycocluster and ranked as follow: **G9-Le^a^**: −262.9, **G7-Le^a^**: −253.7, **G9-3FL**: −224.6 and **G7-3FL**: −205.6.

**Figure 8:**
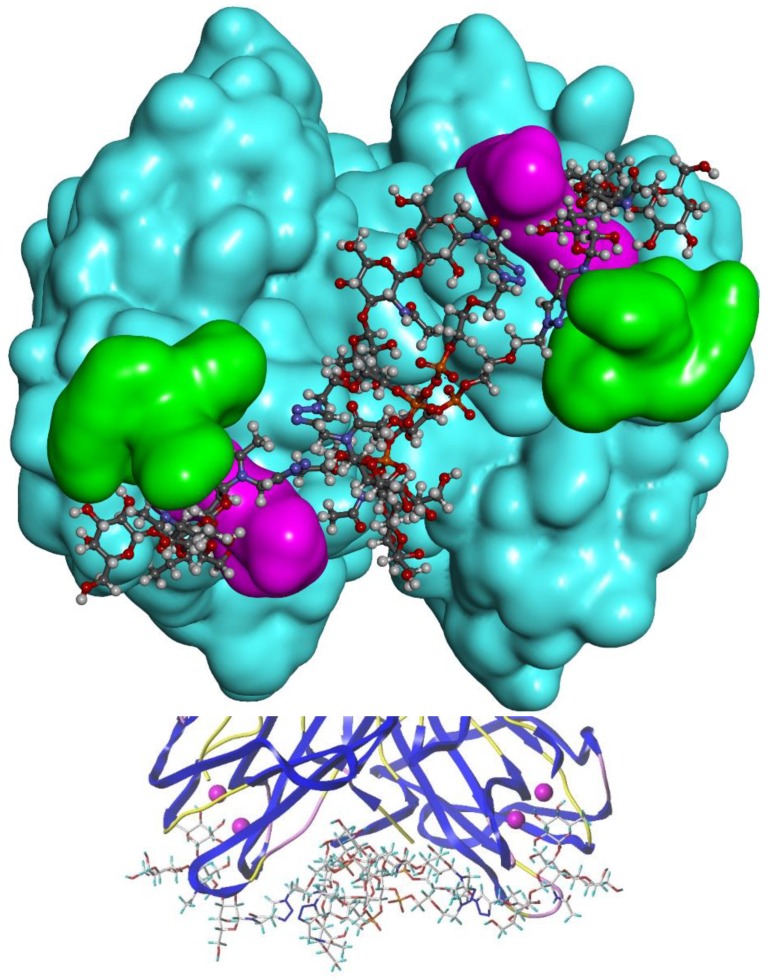
(top) Modelling of the interaction of **G9-Lewis^a^** with LecB. The two loops, V69-P73 and D96-D99, are in green and purple respectively. (bottom) Details of the interaction of **G9-Lewis^a^** with LecB.

For the "best" glycocluster **G9-Le^a^**, a careful inspection of atomic interactions with LecB was performed and is shown in Figure 8. The simultaneous contact of both binding sites of monomer A and D seems to be energetically possible. However, it seems that the two loops, V69-P73 and D96-D99, generate geometrical hindrance and these loops have to be avoided by the two interacting branches. According to our results, longer linkers, such as EG4 (**G10-Lewis^a^**), did not improve the binding (see Figure 6).

## 3. Materials and Methods

### 3.1. Synthesis of β-NAc-Propargyl-Oligoglycoside from Free Oligosaccharides

Starting oligosaccharides were obtained by biofermentation or extracted from biomass (Elicityl catalog: www.elicityl-oligotech.com). Other reagents (propargylamine, Ac_2_O, solvents) were purchased from Thermo Fisher Scientific (Illkirch, France).

The starting free oligosaccharides were converted into oligoglycosylamines by condensation with propargylamine; and in a second step, made non-hydrolysable by selective *N*-acylation, as previously described [41]. Briefly, each starting oligosaccharide (1 eq) was suspended in a pure propargylamine (9 eq.)/methanol mixture. The mixture was stirred at 43 °C for 16 h, to obtain a transient oligoglycosylamine. After completion of the reaction, monitored by thin-layer chromatography, the excess of propargylamine was removed by co-evaporation with MeOH/toluene (1:1, *v/v*). The residual solid was then suspended in a solution of MeOH and Ac_2_O (2:1, *v/v*, 1mL) that corresponds to a large excess of Ac_2_O (10 to 30 eq.). The mixture was stirred at room temperature until total consumption of oligoglycosylamine (checked by TLC) (around 2 h). Next, the acetylated oligoglycosylamine was recovered by co-evaporation with toluene/MeOH (1:1, *v/v*), solubilized in water, and the solution was concentrated and freeze-dried. To avoid any undesired *O*-acetylation, the product was treated by the following additional step. It was dissolved in anhydrous MeOH and MeONa was added to obtain a final MeONa concentration of 0.03 M. The mixture was left for 2 h at room temperature. Water was added, and the solution was brought to pH 7 with aqueous HCl. Finally, the mixture was concentrated. Finally, the final β-*N*Ac-propargyl-oligoglycoside with a purity of 95% was obtained by C_18_ reverse-phase chromatography. The structure was validated by ^1^H-NMR (Avance III 400 MHz, Bruker, Billerica, MA, USA) and MALDI-TOF (Autoflex speed, Bruker, Billerica, MA, USA) analysis.

### 3.2. Cy3-Oligonucleotide Oligoglycoclusters Synthesis

Azide solid support **157** [39,40], *N^3^*-propargyl pentofuranosyl uracils **158–160 [28]** and propargyl-hexopyranoside **161**–**163** [27] were previously synthesized. Tosylated diethyleneglycol **164a [43]** (3.0 g, 11.54 mmol) was co-evaporated three times with dry acetonitrile and then dissolved in dry CH_2_Cl_2_ (20 mL). A few molecular beads of molecular sieve (3Å) were added and diisopropylethylamine (DIEA) (3 mL, 17.3 mmol) under argon. After 15 min, 2-cyanoethyl-*N,N*-diisopropylchlorophosphoramidite (3 g, 12.7 mmol) was added and the solution was stirred at room temperature for 3 h. Then water was added (1 mL) and after 10 min diluted with 200 mL of CH_2_Cl_2_ and washed with a saturated solution of NaHCO_3_ (2 × 200 mL), dried with Na_2_SO_4_ and concentrated. The crude product was eluted from a column of silica gel (110 g) with cyclohexane/EtOAc (9:1 to 7:3, containing 4% of Et_3_N) to afford tosylated diethyleneglycol phosphoramidite **165a**, 4.30 g, 81%. ^1^H- NMR (CDCl_3_, 300 MHz): δ 1.17 (t, *J* = 6.8 Hz, 12H, 4× CH_3_); 2.45 (s, 3H, CH_3_); 2.64 (t, *J* = 6.0 Hz, 2H, CH_2_CN); 3.56 (m, 2H, 2× CHMe_2_); 3.59 (m, 2H, POCH_2_); 3.89 (m, 6H, OCH_2_ ); 4.15 (t, *J* = 4.8 Hz, 2H, SO_3_CH_2_); 7.35 (d, 2H, *J* = 8.1 Hz, Ar); 7.79 (d, 2H, *J* = 8.1 Hz, Ar). ^13^C-NMR (CDCl_3_, 75 MHz): δ.15.08 (CH_2_CN), 15.17 (CH_3_Ar), 19.35, 19.40, 19.44 (4× CH_3_CH), 37.80 and 37.93 (CHMe_2_), 53.17 and 53.41 (POCH_2_CH_2_CN), 57.22 and 57.45 (C5, POCH_2_CH_2_), 63.42 (C7), 64.08 (C8), 66.08 and 66.18 (C6, POCH_2_CH_2_), 112.6 (CN), 122.7 and 124.6 (ArH), 127.7 (Ar-S), 139.6 (Ar-C). ^31^P-NMR (CDCl_3_, 121 MHz): δ 148.65. HRMS (ESI/Q-TOF): Calcd. for C_20_H_34_N_2_O_6_PS 461.1875 [M + H]^+^; found 461.1877.

The same protocol was applied to tosylated tetraethyleneglycol **164b [44]** (3.3 g, 9.48 mmol) affording 4.04 g (78%%) of phosphoramidite derivative **165b**. ^1^H-NMR (CDCl_3_, 300 MHz): δ 1.16 (dd, *J* = 4.1 and 6.6 Hz, 12H, 4× CH_3_); 2.43 (s, 3H, CH_3_); 2.63 (t, 2H, *J* = 6.4 Hz, CH_2_CN); 3.52 (m, 2H, 2× CHMe_2_); 3.72 (m, 2H, POCH_2_); 3.88 (m, 14H, OCH_2_ ); 4.14 (t, *J* = 4.8 Hz, 2H, SO_3_CH_2_); 7.32 (d, 2H, *J* = 8.1 Hz, Ar); 7.77 (d, 2H, *J* = 8.1 Hz, Ar). ^13^C-NMR (CDCl_3_, 75 MHz): δ.22.26 and 22.34 (CH_2_CN), 23.6 (CH3Ar), 26.50, 26.57, 26.60, 26.67 (4× CH_3_CH), 44.95 and 45.12 (CHMe_2_), 60.37 and 60.61 (POCH_2_CH_2_CN), 64.46 and 64.69 (C5, POCH_2_CH_2_), 70.65, 71.25, 72.50, 72.55, 72.64, 72.72 (C7-C12 OCH_2_), 73.15 and 73.24, (C6, POCH_2_CH_2_), 119.8 (CN), 130.0 and 131.8 (ArH), 135.0 (Ar-S), 146.8 (Ar-C). ^31^P-NMR (CDCl_3_, 121 MHz): δ 148.59. HRMS (ESI/Q-TOF): Calcd. for C_24_H_42_N_2_O_8_PS 549.2400 [M + H]^+^; found 549.2393.

### 3.3. 3’-Oligoglycoside-5’-Cy3-Oligonucleotide Synthesis

The oligonucleotide synthesis on solid support was performed on a 381A or 394 DNA synthesizer from Applied Biosystems (Foster City, CA, USA). Reactions under microwave activation were performed on an Monowave 300 system (Anton Paar, Graz, Austria). Solutions of Cap A, Cap B and iodide were purchased from Link Technologies as well as the commercial solid supports. Cap A: Acetic anhydride/pyridine/THF, 1:1:8, *v/v/v*; Cap B: 10% *N*-methylimidazole in THF; Oxidizer solution: 0.1 M iodide in THF/pyridine/H_2_O, 78:20:2, *v/v/v*. Detritylation solution: 3% trichloroacetic acid (TCA) in CH_2_Cl_2_ and dry CH_3_CN for DNA synthesis were purchased from Biosolve (Dieuze, France). Cy3-amidite was purchased from GE Healthcare. All oligoglycooligonucleotides were purified and analyzed by C_18_ reversed-phase HPLC (Macherey-Nagel, Nucleodur 100-3 C18 ec, 4.6 × 75 mm, 3 µM or Nucleodur 100-7 C18 ec, 8 × 125 mm, 7 µm) on an Ultimate 3000 system (Dionex, Sunnyvale, CA, USA) with a Reodyne (Rohnert Park, CA, USA) injector and a UV DAD 3000 detector. Oligonucleotides were dosed by UV-Vis spectrophotometry at 550 nm on a 300 Bio UV-Vis spectrophotometer (Varian Cary, Victoria, Australia).

### 3.4. General Procedure for Immobilization of Polyol Scaffolds (***158***–***163***) on an Azide Solid Support (***157***) by Cu(I)-Catalyzed Alkyne Azide 1,3-Dipolar Cycloaddition

An aqueous solution of propargyl derivatives **158**–**163** (10 µmol in 100 μL H_2_O), freshly prepared degassed aqueous solutions of CuSO_4_ (40 mM, 8 µmol, 50 μL) and sodium ascorbate (100 mM, 40 µmol, 100 μL), degassed water (400 μL) and MeOH (350 μL) were added to 5 µmol of azide solid support **157**. The resulting mixture in a sealed tube G10 was heated to 60 °C for 60 min, stirring at 400 r/min, using a microwave synthesizer. The temperature was monitored with an internal infrared probe. The solution was removed, and CPG beads were washed with H_2_O (3 × 2 mL), MeOH (3 × 2 mL) and CH_2_Cl_2_ (3 × 2 mL), and dried affording the solid-supported polyol scaffolds. 

### 3.5. Introduction of Linkers ***165a***–***b***, Oligonucleotide Synthesis and Cy3 Labeling

Introduction of tosyl linkers: Solid-supported polyol scaffolds (1 µmol) were treated, on a DNA synthesizer, with phosphoramidites **165a** or **165b** using coupling, oxidation and capping steps. For the coupling step, benzylmercaptotetrazole (BMT) was used as an activator (0.3 M in anhydrous CH_3_CN) and phosphoramidites **165a** or **165b** (0.15 M in anhydrous CH_3_CN) were introduced four times with a 180 s coupling time. Oxidation was performed with oxidizer solution for 15 s. Capping was applied using CapA + CapB for 120 s.

Then, the elongation of DNA sequences was carried out using a standard synthesis cycle at the 1 µmol scale by phosphoramidite chemistry. For the coupling step, BMT and commercially available nucleoside phosphoramidites (0.1 M in anhydrous CH_3_CN) were introduced with a 20 s coupling time and Cy3 amidite (0.06 M in anhydrous CH_3_CN) with a 180 s coupling time. The capping step was performed with CapA + CapB for 15 s. Each oxidation was performed with oxidizer solution for 15 s. Detritylation was performed with 3% TCA in CH_2_Cl_2_ for 65 s.

### 3.6. General Procedure for Azidation

The solid-supported oligonucleotides bearing the tri—or tetra-tosyl scaffold (1 µmol) were treated with a solution of tetramethylguanidinuim azide (TMG-N_3_) (0.4 M in CH_3_CN, 1 mL) for 2 h at 65 °C. The beads were washed with acetonitrile (5 × 2 mL) and dried by flushing with argon.

### 3.7. General Procedure for Deprotection

The CPG beads bearing modified oligonucleotides with Cy3 and azide linkers were transferred to a 4 mL screw-top vial and treated with 2 mL of concentrated aqueous ammonia overnight at rt. The supernatants were withdrawn and evaporated, affording crude oligonucleotide mono-, tri- or tetra-azide conjugates.

### 3.8. Purification

Each construction was purified on a C_18_ reverse-phase column. The pure oligonucleotide azide derivatives were aliquoted to 20 nmol for subsequent conjugation with oligosaccharides **166**–**170**.

### 3.9. General Procedure for CuAAC in Solution 

Method A: Mono-conjugation. In a G4 microwave tube from Anton-Paar, Oligonucleotide-hexylazide (20 nmol in 20 µL H_2_O) oligosaccharide **166**–**170** (40 nmol in 40 µL H_2_O), ~0.1 mg of copper nanopowder were added and the mixture was sonicated for 2 s and then stirred at 65 °C with microwave assistance for 90 min. 

Method B: Tri-conjugation. In a microwave tube G4 from Anton-Paar, Oligonucleotide-pentafuranosyl triazide (20 nmol in 20 µL H_2_O) oligosaccharide **166**–**169** (100 nmol in 100 µL H_2_O), ~0.1 mg of copper nanopowder were added and the mixture was sonicated for 2 s and then stirred at 65 °C under microwave assistance for 90 min. 

Method C: Tri-conjugation. In a G4 microwave tube from Anton-Paar, Oligonucleotide-pentafuranosyl triazide (20 nmol in 20 µL H_2_O), oligosaccharide **166**–**169** (100 nmol in 50 µL H_2_O), THPTA (60 nmol, 3 µL) and ~0.1 mg of copper nanopowder were added and the mixture was sonicated for 2 s and then stirred at 65 °C under microwave assistance for 60 min. 

Method D: Tetra-conjugation. In a G4 microwave tube from Anton-Paar, Oligonucleotide-hexapyranosyl tetraazide (20 nmol in 20 µL H_2_O), oligosaccharide **166**–**169** (140 nmol in 140 µL H_2_O) and ~0.1 mg of copper nanopowder were added and the mixture was sonicated for 2 s and then stirred at 65 °C under microwave assistance for 90 min. 

Method E: Tri-conjugation. In a G4 microwave tube from Anton-Paar, Oligonucleotide- hexapyranosyl tetraazide (20 nmol in 20 µL H_2_O), oligosaccharide **166**–**169** (100 nmol in 50 µL H_2_O), THPTA (60 nmol, 3 µL) and ~0.1 mg of copper nanopowder were added and the mixture was sonicated for 2 s and then stirred at 65 °C under microwave assistance for 60 min. 

Method F: sLe^x^ Tri-conjugation. In a G4 microwave tube from Anton-Paar, Oligonucleotide-pentafuranosyl triazide (20 nmol in 20 µL H_2_O), oligosaccharide **170** (100 nmol in 100 µL H_2_O), 0.4 M TEAAc buffer pH 7.5 (20 µL) and ~0.1 mg of copper nanopowder were added and the mixture was sonicated for 2 s and then stirred at 65 °C under microwave assistance for 120 min. 

Method G: sLe^x^ Tetra-conjugation. In a G4 microwave tube from Anton-Paar, Oligonucleotide-hexapyranosyl tetraazide (20 nmol in 20 µL H_2_O), oligosaccharide **170** (140 nmol in 140 µL H_2_O), 0.4 M TEAAc buffer pH 7.5 (20 µL) and ~0.1 mg of copper nanopowder were added and the mixture was sonicated for 2 s and then stirred at 65 °C under microwave assistance for 120 min. 

### 3.10. General Work-up and Purification

The crude solutions were centrifuged and the supernatant was directly purified by C_18_ reverse-phase HPLC. The products were co-evaporated with water, and then lyophilized.

MALDI-TOF mass spectra were recorded on a Voyager DE mass spectrometer (Applied Biosystems, Foster City, CA, USA) equipped with a nitrogen laser. MALDI conditions were: accelerating voltage 24,000 V; guide wire 0.05% of the accelerating voltage; grid voltage 94% of the accelerating voltage; delay extraction time 500 ns. 1 µL of sample was mixed with 5 µL of a saturated solution of HPA in acetonitrile/water (1:1, *v/v*) containing 10% of ammonium citrate and a few beads of DOWEX 50W-X8 ammonium sulfonic acid resin were added. Then, 1 µL of the mixture was placed on a plate and dried at room temperature and pressure.

### 3.11. General Procedure for Microarray Production

All chemicals were provided by Sigma-Aldrich (Saint Quentin Fallavier, France). When they are purchased from other providers, their origin is then specified.

### 3.12. Microstructured Glass Slide Fabrication

Microarrays were made from Nexterion glass D slides (Schott). They were microstructured with 40 square wells (3 mm width, 102 ± 1 µm depth, with 4.5 mm spacings between each well). The slide microstructuring was performed using xurography and oxide-buffered etch (BOE): (Ammonium fluoride/hydrofluoric acid mixture)/(37% hydrochloric acid)/(Ultra-Pure water, 18.2 MΩ); 1:2:2 (*v/v/v*). 

The slides were washed with a piranha mixture (30% H_2_O_2_/ 97% H_2_SO_4_; 1:2 (*v/v*)) for 30 min in an ultrasound bath and rinsed with water (3 × 5 min). A structured vinyl adhesive mask (40 squares: side 3 mm, spacing 4.5 mm) was stuck on each slide and slides were incubated in the BOE mixture for 2 h at room temperature under agitation. Then, the slides were rinsed with water (3 × 5 min) and the masks were removed from the slides.

### 3.13. Microarray Silanization

The microstructured slides were functionalized by silanization using a gas phase protocol previously described [45]. Briefly, the microstructured slides were cleaned with a piranha solution (30 min), rinsed and heated at 150 °C for 2 h under vacuum (10^−1^ mbar). After introduction of the *tert*-butyl-11-(dimethylamino)silylundecanoate (TDSUM), it was vaporized under reduced pressure and allowed to react for 12 h at pressure ranging between 4 × 10^−1^ and 10^−1^ mbar and the temperature was set at 145 °C. The slides were washed with tetrahydrofuran (THF) and then water (10 min, under ultrasound). The *tert* butyl ester functionalized slides were then converted into the corresponding acid by incubation for 7 h in glacial formic acid at room temperature. After washing with dichloromethane and water (10 min, under ultrasound), acid functions were activated for amine coupling using *N*-hydroxysuccinimide (NHS, 0.1 M) and *N,N*’-diisopropylcarbodiimide (0.1 M) in dry THF overnight at room temperature. The slides were finally rinsed with THF and dichloromethane (10 min, under ultrasound).

### 3.14. Amino-Modified Oligonucleotides Immobilization

Two amino-modified oligonucleotides (sequence c1 and c2) (Eurogentec, Seraing, Belgium) were used. Each oligonucleotide was prepared at 25 µM in phosphate-buffered saline (PBS) 10× (pH 8.5) and spotted using the sciFLEXARRAYER s3 piezo-electric system (Scienion, Berlin, Germany). Thirty two spots (16 spots per amino-modified oligonucleotide) of 0.3 nL were spotted at the bottom of each well of the microarray. The substitution reaction between the amino-modified oligonucleotides and NHS-activated microarray surface was performed for 3 h under water-saturated atmosphere and overnight in normal atmosphere to allow for the gentle drying of the spots. To remove the excess of oligonucleotides, the slides were washed for 30 min at 70 °C in sodium dodecyl sulfate (SDS) 0.1% and rinsed with water.

To avoid nonspecific adsorption, the slides were incubated for 2 h with bovine serum albumin (BSA) at 4% in PBS 1× (pH 7.4). Then, the slides were washed in 0.05% PBS 1×-Tween20 (3 × 3 min), in PBS 1× (3 × 3 min) and rinsed with water. BSA blocking was repeated following the same procedure, after the oligoglycocluster immobilization.

### 3.15. Oligoglycocluster Immobilization

Cy3-oligonucleotide oligoglycoclusters **F1** [46] (Figure 1) exhibiting a complementary sequence of sequence c1 and c2 respectively were prepared at 1 µM in PBS 1× (pH 7.4). In each microwell, 1.5 µL of the oligoglycocluster mixture was incubated for DNA-directed immobilization (DDI) for 3 h at 37 °C in a water-saturated atmosphere. The slides were washed with saline sodium citrate (SSC) 2× SDS 0.1% at 51 °C for 1 min, SSC 2× for 5 min and rinsed with water. Hence, in each microwell, both oligoglycoclusters were immobilized with 16 repetition spots.

### 3.16. General Procedure for the Screening of a Library of Glycans

A library of 156 sugars extracted from biomass or produced by bacterial fermentation (see Supporting Information) issued from the OligoTech^®^ (Elicityl) was screened in a competitive assay. The binding of the 156 carbohydrates was screened toward the lectin LecB (PAO1, Elicityl). Lectin was labeled as previously described [47] using the Alexa Fluor^®^ 647 Microscale Protein Labelling Kit (Invitrogen).

On each slide, nine oligosaccharides and α-l-fucose (as a control) were tested. The inhibitory effect of each oligosaccharide was determined at four concentrations 0.1, 1.0, 5.0 and 15 µM and at 0.5, 2.5, 5.0 and 7.5 mg/L for polymers: Aliginate, galacturonan, galactomannan, glucomannan, mannan, ulvan, xylan and the glucan. To this end, the labeled LecB lectin (0.12 µM final concentration), BSA (2%, final concentration), CaCl_2_ (7.5 µM, final concentration) in PBS 1× (pH 7.4) were mixed with the solutions of oligosaccharides (from the OligoTech^®^, Crolles, France). For each concentration, 1.5 µL of the mixture was poured into a microwell, where **F1** was previously immobilized. The slides were incubated for 3 h at 37 °C in a water-saturated atmosphere. In each microwell, a competition between the two types of glycoclusters (**F1**) and oligosaccharide occurred to bind LecB. The microarrays were washed with 0.02% PBS-Tween20 for 5 min at room temperature. The fluorescence signal emitted by the complex glycocluster/Alexa 647-lectin was detected and an average of the mean fluorescence signal of 16 spots per glycocluster was calculated. The percentage of inhibition was calculated as follows: The alexa-647 fluorescent signal for a given sugar was normalized by the Alexa 647 fluorescent signal measured at 0.1 µM of fucose. Under these conditions, no inhibition of the interaction between LecB and the two fucosylated clusters by fucose is observed (IC_50_ 10 and 20 µM for fucose).

### 3.17. General Procedure for the Screening of Oligoglycoclusters

#### 3.17.1. Probing Oligoglycocluster-LecB Interaction on a Microarray

The interaction of LecB with the oligoglycoclusters was measured with Alexa 647 –labeled LecB. For the screening of the library of oligonucleotide oligoglycoclusters bearing several oligosaccharides (sLe^x^ pentasaccharide, Le^x^ tetrasaccharide, Le^b^ tetrasaccharide, 3FL and Le^a^ tetrasaccharide), two oligoglycoclusters were immobilized per well by DDI with 32 repetition spots. In order to assess the relative surface densities of the oligoglycoclusters, the slides were scanned at 532 nm (excitation of Cy3) using the Axon microarray scanner, GenePix 4100A software package. The fluorescence signal of each oligoglycocluster was determined as the average of the mean fluorescence signal of 32 spots. The fluorescent signal of the oligoglycoclusters deviated by less than 15%.

1.5 µL of Alexa 647-LecB (0.12 µM, 7.5 μM CaCl_2_, 2% BSA in PBS 1×) were added in each well. Incubation was performed at 37 °C for 3 h in a water-saturated atmosphere. The slides were washed with 0.02% PBS-Tween20 for 5 min and rinsed with water. The slides were scanned at 635 nm (excitation of alexa 647) using the Axon microarray scanner, GenePix 4100A software package (Molecular device, San Jose, CA, USA). The Alexa 647-lectin fluorescence signal for each oligoglycocluster was determined as the average of the mean fluorescence signal of 32 spots. Error bars were calculated from these 32 spots.

#### 3.17.2. Dissociation Constant Determination on a Microarray

A similar protocol was used for dissociation constant determination [47,48]. In this case, the lectin LecB concentration was varied from 1 nM to 2400 nM. The Alexa 647 fluorescent signal was recorded, and from the resulting isotherm, the *K_d_* values were determined using the equation:

[LecB]/FI = 1/FI_max_ × [LecB] + Kd/FI_max_ (1)

where FI represents the fluorescent signal recorded at 635 nm for the given concentration of LecB ([LecB]) and FI_max_ is the maximum fluorescent signal obtained at 635 nm. *K_d_* value determination on a microarray deviated by less than 10% [35].

### 3.18. In-Silico Molecular Docking

The lectin structure file was retrieved from the RCSB Protein Data Bank website (PDB code 1UZV). Docking experiments were performed with the GOLD software (The Cambridge Crystallographic Data Centre, Cambridge, UK). The six heavy atoms of the ligand fucose ring found in the structure file were used as a scaffold in the active site. The rest of the ligand was considered as flexible during the docking process. Side chains of the following residues in the vicinity of the active site of the same monomer, S22, S23, T45, E95, D96, T98, D99, N100, D101 and D104 were also defined as flexible during the docking procedure. For each ligand, 10 poses that are energetically reasonable were kept while searching for the correct binding mode of the ligand. The decision to keep a trial pose is based on a computed ligand-receptor interaction energy (score) of that pose. The ChemPLP fitness scoring function is used to rank the poses. The ChemPLP scoring function is the default in Gold version 5.2 used here. Additionally, an empirical potential energy of interaction ΔE for the ranked complexes is evaluated using the simple expression (2):

ΔE (interaction) = E(complex) − (E(protein) + E(ligand))(2)

For that purpose, the Spectroscopic Empirical Potential Energy function SPASIBA and the corresponding parameters are used [49,50]. Molecular graphics and analysis were performed using the Discovery Studio Visualizer 4.0 software (Accelrys, San Diego, CA, USA).

## 4. Conclusions

The gram negative bacterium *Pseudomonas aeruginosa* is an opportunistic bacterium. It is a causative agent of lung infections, the major cause of morbidity and mortality among cystic fibrosis patients and it remains a leading pathogen in nosocomial infections. It causes severe and chronic infections due to its ability to form a biofilm that gives a selective advantage to the bacteria with respect to antibiotherapy and host defenses. During the formation of the biofilm, several virulent factors are involved. Among these virulence factor, lectins play a key role. Therefore, molecules inhibiting these lectins are envisioned as new anti-bacterial agents. The soluble homotetrameric fucophilic lectin LecB of PA is classified as a virulent factor and is involved in biofilm formation, host/bacteria and bacteria/bacteria interaction, cytotoxicity and inhibition of ciliated removal. 

Herein, 156 glycans were screened for their binding toward LecB through screening on a glycoarray, where they competed with an immobilized fucocluster. This screening by competition is straightforward and allows for the rapid evaluation of the binding of simple to complex oligosaccharides using only a few mg of them. The glycans to be evaluated were directly used without any derivatization. Among these 156 glycans, 36 were able to interact with LecB with different bindings from low to high. Five oligosaccharides, Lewis^a^, Lewis^b^, Lewis^x^ tetrasaccharides, sialyl-Lewis^x^ pentasaccharide and 3’-Fucosyllactose (3FL), exhibiting good affinity for LecB, were selected for the synthesis of further oligoglycoclusters. To this end, they were first propargylated and then introduced by CuAAC on furanoside or pyranoside scaffolds exhibiting di- or tetra-ethyleneglycolazide arms. A library of 55 glycoclusters (50 multivalent ligands + 5 monovalent ligands) exhibiting one, three or four times the five selected oligocarbohydrates and a DNA tag was prepared. Thanks to the DNA tag, they were easily immobilized on a DNA chip allowing for their rapid screening against LecB using only few tens of picomoles of material. The highest bindings were found for Le^a^, closely followed by 3FL and then by Le^b^, Le^x^ and with the lowest affinity for sLe^x^. From these two screenings, LecB seems to preferentially bind Fucα1-4 > Fucα1-3 > Fucα1-2 oligosaccharides. Additional fucose on the side chain does not necessarily increase the binding. The clusters built from a galactose core were found to be the best. However, multivalence does increase the binding and simulation seems to be in favor of a divalent contact. However, the loops V69–P73 and D96–D99 seem to generate a geometrical constrain. However, the stoichiometry of the binding remains to be confirmed by Isothermal Titration Calorimetry.

The data showed that oligoglycoclusters built from Le^a^ tetrasaccharide and 3FL display higher affinity to LecB than the mannose-centered tetrafucocluster PC_2, confirming the interest to search for new glycans to improve the affinity of glycoclusters to LecB.

Several authors have demonstrated that LecB inhibitors are able to reduce biofilm formation, bacterial adhesion and consequently to provide protection against PA infections. [1,31] Therefore future work will focus on in-vitro and in-vivo testing for the evaluation of these clusters as anti-infectious molecules, eventually in combination with conventional bactericide molecules.

## Figures and Tables

**Table:** 

Oligoglycoclusters	Scaffold	Oligosaccharide Used
G1-Le^a^, Le^b^, 3FL, Le^x^	Ara-EG_2_ *n* = 1	166, 167, 168, 169
G2-Le^a^, Le^b^, 3FL, Le^x^	Ara-EG_4_ *n* = 3	166, 167, 168, 169
G3-Le^a^, Le^b^, 3FL, Le^x^	Xylo-EG_2_ *n* = 1	166, 167, 168, 169
G4-Le^a^, Le^b^, 3FL, Le^x^	Xylo-EG_4_ *n* = 3	166, 167, 168, 169
G5-Le^a^, Le^b^, 3FL, Le^x^	Ribo-EG_2_ *n* = 1	166, 167, 168, 169
G6-Le^a^, Le^b^, 3FL, Le^x^, sLe^x^	Ribo-EG_4_ *n* = 3	166, 167, 168, 169, 170

**Table:** 

Oligoglycoclusters	Scaffold	Oligosaccharide Used
G7-Le^a^, Le^b^, 3FL, Le^x^	Glc-EG_2_ *n* = 1	166, 167, 168, 169
G8-Le^a^, Le^b^, 3FL, Le^x^	Glc-EG_4_ *n* = 3	166, 167, 168, 169
G9-Le^a^, Le^b^, 3FL, Le^x^	Gal-EG_2_ *n* = 1	166, 167, 168, 169
G10-Le^a^, Le^b^, 3FL, Le^x^	Gal-EG_4_ *n* = 3	166, 167, 168, 169
G11-Le^a^, Le^b^, 3FL, Le^x^	Man-EG_2_ *n* = 1	166, 167, 168, 169
G12-Le^a^, Le^b^, 3FL, Le^x^, sLe^x^	Man-EG_4_ *n* = 3	166, 167, 168, 169, 170

**Table:** 

Oligoglycoclusters	Oligosaccharide used
M-Le^a^, Le^b^, 3FL, Le^x^, sLe^x^	166, 167, 168, 169, 170

**Table 1:** Oligosaccharides selected for the synthesis of oligoglycoclusters.

	Structure	Grade	Id.
Lewis^a^ (Le^a^) tetrasaccharide	Galβ1-3(Fucα1-4)GlcNAcβ1-3Gal	A	30
3-Fucosyllactose (3FL)	Galβ1-4(Fucα1-3)Glc	B	33
Lewis^b^ (Le^b^) pentasaccharide	Fucα1-2Galβ1-3(Fucα1-4)GlcNAcβ1-3Gal	C	32
Sialyl-Lewis^x^ (sLe^x^) pentasaccharide	Neu5Acα2-3Galβ1-4(Fucα1-3)GlcNAcβ1-3Gal	C	29
Lewis^x^ (Le^x^) tetrasaccharide	Galβ1-4(Fucα1-3)GlcNAcβ1-3Gal	C	26

**Table 2:** Summary of the interaction of Lewis^a^ tetrasaccharide, Lewis^x^ tetrasaccharide and 3-Fucosyllactose (3FL) with LecB. Red. and nonred. stand for reductive and nonreductive ends. (all amino acids belong to monomer B except G114 (monomer A).

	Lewis^a^	Lewis^x^	3FL
Gal _red_	/	O_4_H <-> T98	/
Glc or GlcNAc	O sp2 amide < -T98	O sp2 amide <-R72	O _ring_ < -S23
Gal _nonred_	O_1_H, O _ring_, O_6_H < -R72	O_2_H <-R72	O_2_H- > D96
Fuc	O_2_H- > D96 and D99 O _ring_ < -S23 and S22 O_3_H- > D99 and D101 O_4_H- > G114 and D104	O_2_H-> D96, E95, D99 and D104 O _ring_ < -S23 and S22 O_3_H- > D99 and D101 O_4_H- > G114	O_2_H- > D96, D104 and D99 O _ring_ < -S23 O_3_H- > D99 and D101 O_4_H- > G114 and D104

## Supplementary Materials

The following are available online, Figure S1: List of the 156 oligosaccharides screened in the competitive assay on a microarray, S2 Results of the competitive screening of the 156 glycans toward LecB, S3 NMR spectra of compounds 165a and 165b, S4 characterization for propargylated oligosaccharides: Compounds 166 to 170, S5 Coupling conditions and characterization data for the 50 oligoglycoclusters and the 5 monovalent ligands, S6 HPLC chromatograms of oligoglycoclusters with 3-Fucosyl-Lactose (3-FL), S7 HPLC chromatograms of oligoglycoclusters with Lewis^a^, S8 HPLC chromatograms of oligoglycoclusters with Lewis^b^, S9 HPLC chromatograms of oligoglycoclusters with Lewis^x^, S10 HPLC chromatograms of oligoglycoclusters with Sialyl Lewis^x^.

## References

[1] Sommer R., Wagner S., Rox K., Varrot A., Hauck D., Wamhoff E.C., Schreiber J., Ryckmans T., Brunner T., Rademacher C. (2018). Glycomimetic, Orally Bioavailable LecB Inhibitors Block Biofilm Formation of Pseudomonas aeruginosa. J. Am. Chem. Soc..

[2] Lundquist J.J., Toone E.J. (2002). The cluster glycoside effect. Chem. Rev..

[3] Bernardi A., Jimenez-Barbero J., Casnati A., De Castro C., Darbre T., Fieschi F., Finne J., Funken H., Jaeger K.E., Lahmann M. (2013). Multivalent glycoconjugates as anti-pathogenic agents. Chem. Soc. Rev..

[4] Chabre Y.M., Roy R. (2013). Multivalent glycoconjugate syntheses and applications using aromatic scaffolds. Chem. Soc. Rev..

[5] Branson T.R., Turnbull W.B. (2013). Bacterial toxin inhibitors based on multivalent scaffolds. Chem. Soc. Rev..

[6] Engel J., Hauser A., Rello J. (2003). Molecular pathogenesis of acute Pseudomonas aeruginosa infections. Severe Infections Caused by Pseudomonas Aeruginosa.

[7] Gibson R.L., Burns J.L., Ramsey B.W. (2003). Pathophysiology and management of pulmonary infections in cystic fibrosis. Am J Resp Crit Care.

[8] Gellatly S.L., Hancock R.E.W. (2013). Pseudomonas aeruginosa: New insights into pathogenesis and host defenses. Pathog. Dis..

[9] Munguia J., Nizet V. (2017). Pharmacological Targeting of the Host-Pathogen Interaction: Alternatives to Classical Antibiotics to Combat Drug-Resistant Superbugs. Trends Pharmacol. Sci..

[10] Imberty A., Wimmerova M., Mitchell E.P., Gilboa-Garber N. (2004). Structures of the lectins from Pseudomonas aeruginosa: Insights into the molecular basis for host glycan recognition. Microb. Infect..

[11] Mitchell E.P., Sabin C., Snajdrova L., Pokorna M., Perret S., Gautier C., Hofr C., Gilboa-Garber N., Koca J., Wimmerova M. (2005). High affinity fucose binding of Pseudomonas aeruginosa lectin PA-IIL: 1.0 angstrom resolution crystal structure of the complex combined with thermodynamics and computational chemistry approaches. Proteins.

[12] Sommer R., Wagner S., Varrot A., Nycholat C.M., Khaledi A., Haussler S., Paulson J.C., Imberty A., Titz A. (2016). The virulence factor LecB varies in clinical isolates: Consequences for ligand binding and drug discovery. Chem. Sci..

[13] Sabin C., Mitchell E.P., Pokorna M., Gautier C., Utille J.P., Wimmerova M., Imberty A. (2006). Binding of different monosaccharides by lectin PA-IIL from Pseudomonas aeruginosa: Thermodynamics data correlated with X-ray structures. FEBS Lett..

[14] Boukerb A.M., Decor A., Ribun S., Tabaroni R., Rousset A., Commin L., Buff S., Doleans-Jordheim A., Vidal S., Varrot A. (2016). Genomic Rearrangements and Functional Diversification of lecA and lecB Lectin-Coding Regions Impacting the Efficacy of Glycomimetics Directed against Pseudomonas aeruginosa. Front. Microbiol..

[15] Andreini M., Anderluh M., Audfray A., Bernardi A., Imberty A. (2010). Monovalent and bivalent N-fucosyl amides as high affinity ligands for Pseudomonas aeruginosa PA-IIL lectin. Carbohydr. Res..

[16] Hauck D., Joachim I., Frommeyer B., Varrot A., Philipp B., Moller H.M., Imberty A., Exner T.E., Titz A. (2013). Discovery of Two Classes of Potent Glycomimetic Inhibitors of Pseudomonas aeruginosa LecB with Distinct Binding Modes. ACS Chem. Biol..

[17] Mitchell E., Houles C., Sudakevitz D., Wimmerova M., Gautier C., Perez S., Wu A.M., Gilboa-Garber N., Imberty A. (2002). Structural basis for oligosaccharide-mediated adhesion of Pseudomonas aeruginosa in the lungs of cystic fibrosis patients. Nat. Struc. Biol..

[18] Topin J., Arnaud J., Sarkar A., Audfray A., Gillon E., Perez S., Jamet H., Varrot A., Imberty A., Thomas A. (2013). Deciphering the Glycan Preference of Bacterial Lectins by Glycan Array and Molecular Docking with Validation by Microcalorimetry and Crystallography. PLoS ONE.

[19] Gilboa-Garber N., Sudakevitz D., Sheffi M., Sela R., Levene C. (1994). PA-I and PA-II Lectin Interactions with the Abo(H)-Blood and P-Blood Group Glycosphingolipid Antigens May Contribute to the Broad-Spectrum Adherence of Pseudomonas-Aeruginosa to Human Tissues in Secondary Infections. Glycoconj. J..

[20] Perret S., Sabin C., Dumon C., Pokorna M., Gautier C., Galanina O., Ilia S., Bovin N., Nicaise M., Desmadril M. (2005). Structural basis for the interaction between human milk oligosaccharides and the bacterial lectin PA-IIL of Pseudomonas aeruginosa. Biochem. J..

[21] Zinger-Yosovich K.D., Gilboa-Garber N. (2009). Blocking of Pseudomonas aeruginosa and Ralstonia solanacearum Lectins by Plant and Microbial Branched Polysaccharides Used as Food Additives. J. Agric. Food Chem..

[22] Imberty A., Chabre Y.M., Roy R. (2008). Glycomimetics and glycodendrimers as high affinity microbial anti-adhesins. Chem. Eur. J..

[23] Marotte K., Eville C.P., Sabin C., Pymbock M.M., Imberty A., Roy R. (2007). Synthesis and binding properties of divalent and trivalent clusters of the Lewis a disaccharide moiety to Pseudomonas aeruginosa lectin PA-IIL. Org. Biomol. Chem..

[24] Morvan F., Meyer A., Jochum A., Sabin C., Chevolot Y., Imberty A., Praly J.P., Vasseur J.J., Souteyrand E., Vidal S. (2007). Fucosylated pentaerythrityl phosphodiester oligomers (PePOs): Automated synthesis of DNA-Based glycoclusters and binding to Pseudomonas aeruginosa lectin (PA-IIL). Bioconjugate Chem..

[25] Ligeour C., Audfray A., Gillon E., Meyer A., Galanos N., Vidal S., Vasseur J.J., Imberty A., Morvan F. (2013). Synthesis of branched-phosphodiester and mannose-centered fucosylated glycoclusters and their binding studies with Burkholderia ambifaria lectin (BambL). RSC Adv..

[26] Johansson E.M.V., Kadam R.U., Rispoli G., Crusz S.A., Bartels K.M., Diggle S.P., Camara M., Williams P., Jaeger K.E., Darbre T. (2011). Inhibition of Pseudomonas aeruginosa biofilms with a glycopeptide dendrimer containing D-amino acids. Med. Chem. Comm..

[27] Gerland B., Goudot A., Pourceau G., Meyer A., Dugas V., Cecioni S., Vidal S., Souteyrand E., Vasseur J.J., Chevolot Y. (2012). Synthesis of a Library of Fucosylated Glycoclusters and Determination of their Binding toward Pseudomonas aeruginosa Lectin B (PA-IIL) Using a DNA-Based Carbohydrate Microarray. Bioconjugate Chem..

[28] Ligeour C., Dupin L., Angeli A., Vergoten G., Vidal S., Meyer A., Souteyrand E., Vasseur J.J., Chevolot Y., Morvan F. (2015). Importance of topology for glycocluster binding to Pseudomonas aeruginosa and Burkholderia ambifaria bacterial lectins. Org. Biomol. Chem..

[29] Berthet N., Thomas B., Bossu I., Dufour E., Gillon E., Garcia J., Spinelli N., Imberty A., Dumy P., Renaudet O. (2013). High Affinity Glycodendrimers for the Lectin LecB from Pseudomonas aeruginosa. Bioconjugate Chem..

[30] Michaud G., Visini R., Bergmann M., Salerno G., Bosco R., Gillon E., Richichi B., Nativi C., Imberty A., Stocker A. (2016). Overcoming antibiotic resistance in Pseudomonas aeruginosa biofilms using glycopeptide dendrimers. Chem. Sci..

[31] Boukerb A.M., Rousset A., Galanos N., Mear J.-B., Thepaut M., Grandjean T., Gillon E., Cecioni S., Abderrahmen C., Faure K. (2014). Antiadhesive Properties of Glycoclusters against Pseudomonas aeruginosa Lung Infection. J. Med. Chem..

[32] Buffet K., Gillon E., Holler M., Nierengarten J.F., Imberty A., Vincent S.P. (2015). Fucofullerenes as tight ligands of RSL and LecB, two bacterial lectins. Org. Biomol. Chem..

[33] Buffet K., Nierengarten I., Galanos N., Gillon E., Holler M., Imberty A., Matthews S.E., Vidal S., Vincent S.P., Nierengarten J.F. (2016). Pillar [5] arene-Based Glycoclusters: Synthesis and Multivalent Binding to Pathogenic Bacterial Lectins. Chem. Eur. J..

[34] Hu Y.X., Beshr G., Garvey C.J., Tabor R.F., Titz A., Wilkinson B.L. (2017). Photoswitchable Janus glycodendrimer micelles as multivalent inhibitors of LecA and LecB from Pseudomonas aeruginosa. Colloid Surf..

[35] Dupin L., Zuttion F., Gehin T., Meyer A., Phaner-Goutorbe M., Vasseur J.J., Souteyrand E., Morvan F., Chevolot Y. (2015). Effects of the Surface Densities of Glycoclusters on the Determination of Their IC_50_ and K_d_ Value Determination by Using a Microarray. Chem. Bio. Chem..

[36] Liang P.H., Wang S.K., Wong C.H. (2007). Quantitative analysis of carbohydrate-protein interactions using glycan microarrays: Determination of surface and solution dissociation constants. J. Am. Chem. Soc..

[37] Smith E.A., Thomas W.D., Kiessling L.L., Corn R.M. (2003). Surface plasmon resonance imaging studies of protein-carbohydrate interactions. J. Am. Chem. Soc..

[38] Wu A.M., Wu J.H., Singh T., Liu J.H., Tsai M.S., Gilboa-Garber N. (2006). Interactions of the fucose-specific Pseudomonas aeruginosa lectin, PA-IIL, with mammalian glycoconjugates bearing polyvalent Lewis(a) and ABH blood group glycotopes. Biochimie.

[39] Pourceau G., Meyer A., Chevolot Y., Souteyrand E., Vasseur J.J., Morvan F. (2010). Oligonucleotide Carbohydrate-Centered Galactosyl Cluster Conjugates Synthesized by Click and Phosphoramidite Chemistries. Bioconjugate Chem..

[40] Pourceau G., Meyer A., Vasseur J.J., Morvan F. (2009). Azide Solid Support for 3’-Conjugation of Oligonucleotides and Their Circularization by Click Chemistry. J. Org. Chem..

[41] Chwalek M., Auzely R., Fort S. (2009). Synthesis and biological evaluation of multivalent carbohydrate ligands obtained by click assembly of pseudo-rotaxanes. Org. Biomol. Chem..

[42] Gerland B., Goudot A., Ligeour C., Pourceau G., Meyer A., Vidal S., Gehin T., Vidal O., Souteyrand E., Vasseur J.J. (2014). Structure Binding Relationship of Galactosylated Glycoclusters toward Pseudomonas aeruginosa Lectin LecA Using a DNA-Based Carbohydrate Microarray. Bioconjugate Chem..

[43] Ashton P.R., Huff J., Menzer S., Parsons I.W., Preece J.A., Stoddart J.F., Tolley M.S., White A.J.P., Williams D.J. (1996). Bis 2 catenanes and a bis 2 rotaxane—Model compounds for polymers with mechanically interlocked components. Chem. Eur. J..

[44] Zhang L., Sun L.L., Cui Z.Y., Gottlieb R.L., Zhang B.L. (2001). 5’-sulfhydryl-modified RNA: Initiator synthesis, in vitro transcription, and enzymatic incorporation. Bioconjugate Chem..

[45] Phaner-Goutorbe M., Dugas V., Chevolot Y., Souteyrand E. (2011). Silanization of silica and glass slides for DNA microarrays by impregnation and gas phase protocols: A comparative study. Mat. Sci. Eng. C-Mater..

[46] Zhang J., Pourceau G., Meyer A., Vidal S., Praly J.P., Souteyrand E., Vasseur J.J., Morvan F., Chevolot Y. (2009). Specific recognition of lectins by oligonucleotide glycoconjugates and sorting on a DNA microarray. Chem. Commun..

[47] Goudot A., Pourceau G., Meyer A., Gehin T., Vidal S., Vasseur J.J., Morvan F., Souteyrand E., Chevolot Y. (2013). Quantitative analysis (Kd and IC50) of glycoconjugates interactions with a bacterial lectin on a carbohydrate microarray with DNA Direct Immobilization (DDI). Biosens. Bioelectron..

[48] Park S., Shin I. (2007). Carbohydrate microarrays for assaying galactosyltransferase activity. Org. Lett..

[49] Vergoten G., Mazur I., Lagant P., Michalski J.C., Zanetta J.P. (2003). The SPASIBA force field as an essential tool for studying the structure and dynamics of saccharides. Biochimie.

[50] Lagant P., Nolde D., Stote R., Vergoten G., Karplus M. (2004). Increasing normal modes analysis accuracy: The SPASIBA spectroscopic force field introduced into the CHARMM program. J. Phys. Chem. A.

