# Supplementary material for: Screening of a Library of Oligosaccharides Targeting Lectin LecB of Pseudomonas Aeruginosa and Synthesis of High Affinity Oligoglycoclusters
Source: Molecules. 2018 Nov 24;23(12):3073. doi: 10.3390/molecules23123073 (PMC6321166; doi:10.3390/molecules23123073)
Supplement: Supplementary file 1 [file molecules-23-03073-s001.pdf]

Supplementary Materials

S1 List of the 156 glycans screened.

Structures	Id.
Galβ1-3GlcNAcβ1-4Gal 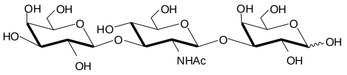	1
Galβ1-4GlcNAcβ1-4Gal 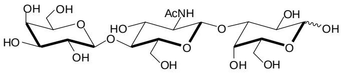	2
Galβ1-3GlcNAc 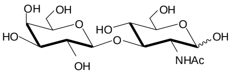	3
Galβ1-3GlcNAcβ1-3Galβ1-4Glc 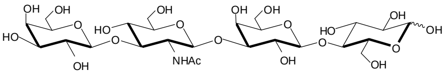	4
GlcNAcβ1-3Galβ1-4Glc 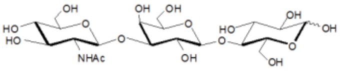	5
Galβ1-4GlcNAcβ1-3Galβ1-4Glc 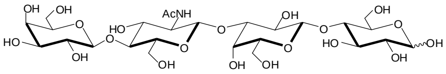	6
Galβ1-4(GlcNAcβ1-3Galβ1-4)$_2$Glc 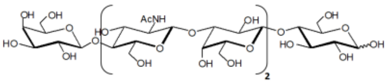	7
Galβ1-4(GlcNAcβ1-3Galβ1-4)$_3$Glc 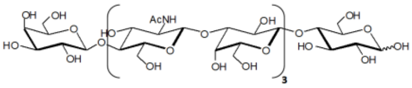	8
Fucα1-2Gal 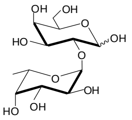	9
Fucα1-2Galβ1-4Glc 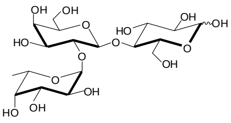	10

Fucα1-2Galβ1-3GlcNAcβ1-3Gal 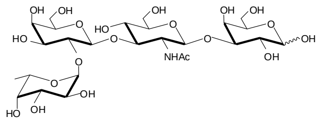	11
Fucα1-2Galβ1-4GlcNAcβ1-3Gal 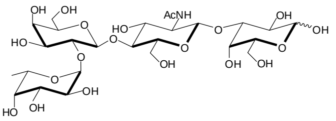	12
Fucα1-2Galβ1-3GlcNAcβ1-3Galβ1-4Glc 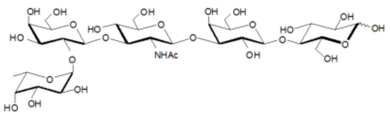	13
Fucα1-2Galβ1-4GlcNAcβ1-3Galβ1-4Glc 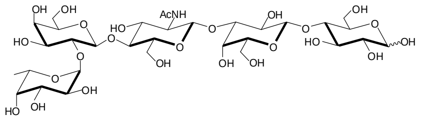	14
GalNAcα1-3(Fucα1-2)Gal 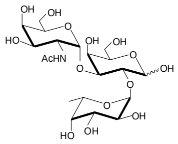	15
GalNAcα1-3(Fucα1-2)Galβ1-4Glc 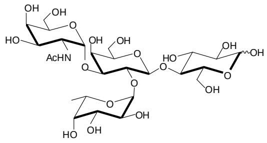	16
GalNAcα1-3(Fucα1-2)Galβ1-3GlcNAcβ1-3Gal 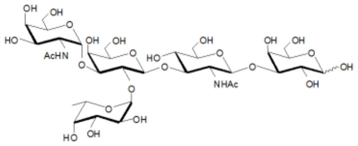	17
GalNAcα1-3(Fucα1-2)Galβ1-4GlcNAcβ1-3Gal 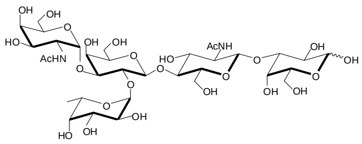	18
GalNAcα1-3(Fucα1-2)Galβ1-3GlcNAcβ1-3Galβ1-4Glc 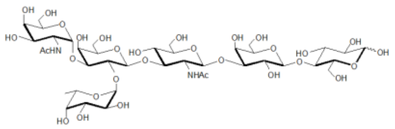	19

GalNAcα1-3(Fucα1-2)Galβ1-4GlcNAcβ1-3Galβ1-4Glc 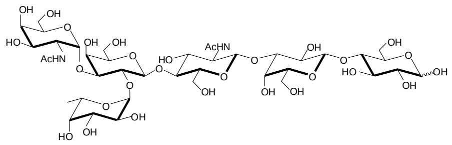	20
Galα1-3(Fucα1-2)Galβ1-4Glc 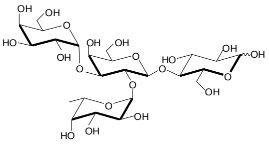	21
Galα1-3(Fucα1-2)Galβ1-3GlcNAcβ1-3Gal 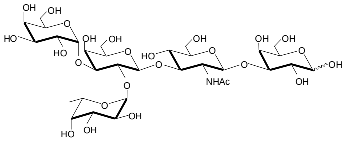	22
Galα1-3(Fucα1-2)Galβ1-4GlcNAcβ1-3Gal 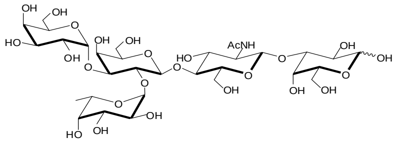	23
Galα1-3(Fucα1-2)Galβ1-3GlcNAcβ1-3Galβ1-4Glc 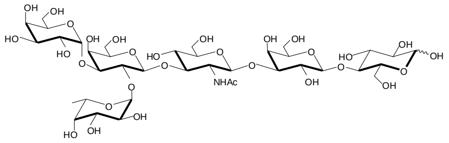	24
Galα1-3(Fucα1-2)Galβ1-4GlcNAcβ1-3Galβ1-4Glc 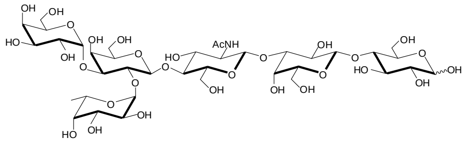	25
Galβ1-4(Fucα1-3)GlcNAcβ1-3Gal 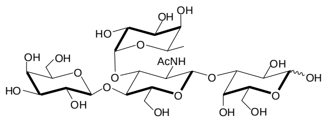	26
Galβ1-4(Fucα1-3)GlcNAcβ1-3Galβ1-4(Fucα1-3)Glc 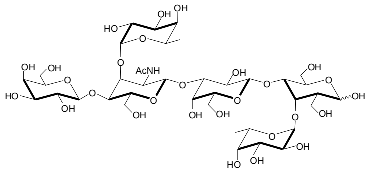	27
Fucα1-2Galβ1-4(Fucα1-3)GlcNAcβ1-3Gal 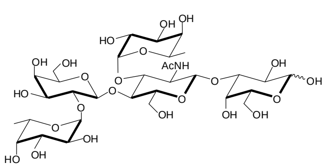	28

Neu5Acα2-3Galβ1-4(Fucα1-3)GlcNAcβ1-3Gal 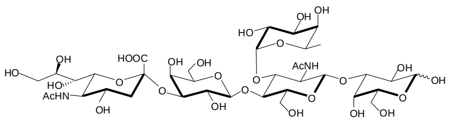	29
Galβ1-3(Fucα1-4)GlcNAcβ1-3Gal 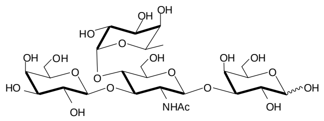	30
Galβ1-3(Fucα1-4)GlcNAcβ1-3Galβ1-4(Fucα1-3)Glc 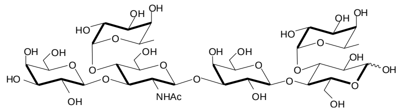	31
Fucα1-2Galβ1-3(Fucα1-4)GlcNAcβ1-3Gal 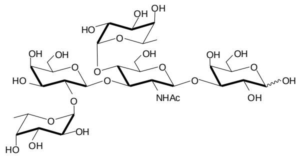	32
Galβ1-4(Fucα1-3)Glc 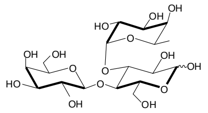	33
Galβ1-4GlcNAcβ1-3Galβ1-4(Fucα1-3)Glc 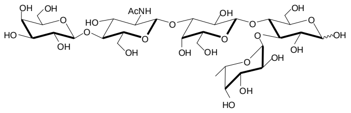	34
Galβ1-3GlcNAcβ1-3Galβ1-4(Fucα1-3)Glc 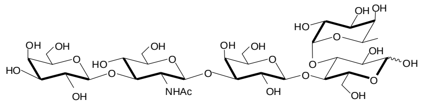	35
Neu5Acα2-3Galβ1-4(Fucα1-3)Glc 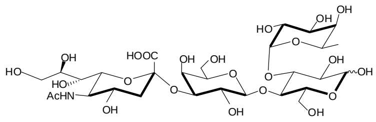	36
Galα1-3Galβ1-4(Fucα1-3)Glc 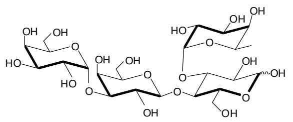	37
Fucα1-2Galβ1-4(Fucα1-3)Glc 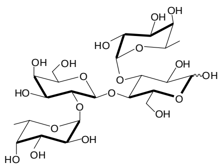	38

GalNAcα1-3(Fucα1-2)Galβ1-4(Fucα1-3)Glc 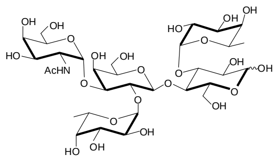	39
Galα1-3(Fucα1-2)Galβ1-4(Fucα1-3)Glc 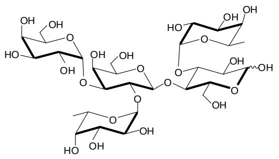	40
Galα1-3Galβ1-4Glc 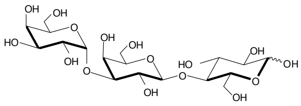	41
Galα1-3Galβ1-4GlcNAcβ1-3Galβ1-4Glc 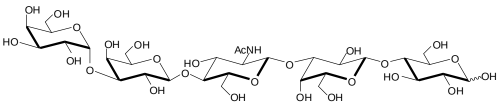	42
GalNAcβ1-3Galα1-3Galβ1-4Glc 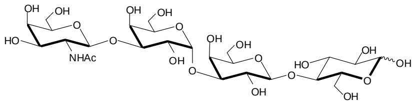	43
Galβ1-3GalNAcβ1-3Galα1-3Galβ1-4Glc 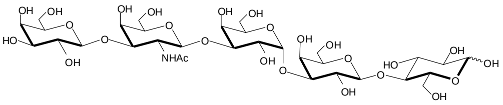	44
Galα1-3(Galβ1-4GlcNAcβ1-3)$_2$Galβ1-4Glc 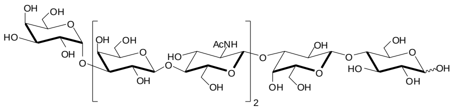	45
Galα1-3(Galβ1-4GlcNAcβ1-3)$_3$Galβ1-4Glc 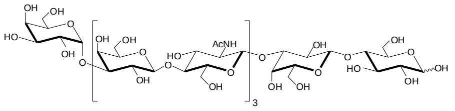	46
Galα1-3(Galβ1-4GlcNAcβ1-3)4Galβ1-4Glc 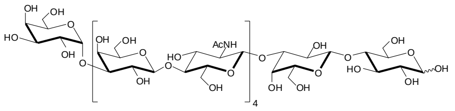	47
Neu5Acα2-3Galβ1-3GlcNAcβ1-3Gal 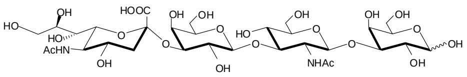	48
Neu5Acα2-3Galβ1-3GlcNAcβ1-3Galβ1-4Glc 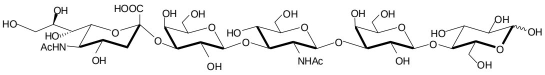	49

Neu5Acα2-3Galβ1-4GlcNAcβ1-3Gal 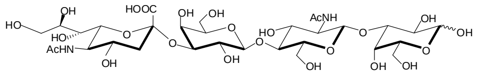	50
Neu5Acα2-3Galβ1-4GlcNAcβ1-3Galβ1-4Glc 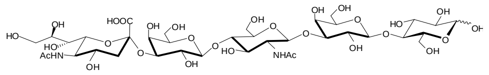	51
Neu5Acα2-6Galβ1-4Glc 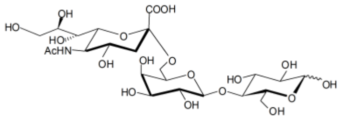	52
Neu5Acα2-3Galβ1-4Glc 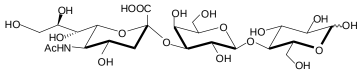	53
Neu5Acα2-8Neu5Acα2-3Galβ1-4Glc 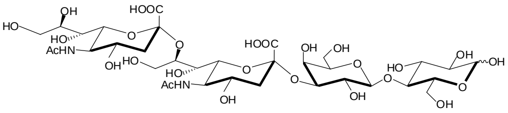	54
Neu5Acα2-8Neu5Acβ2-8Neu5Acα2-3Galβ1-4Glc 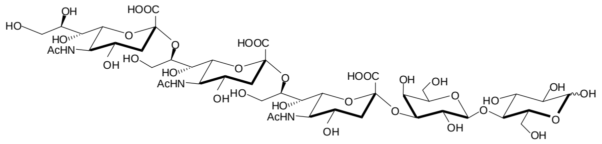	55
GalNAcβ1-4(Neu5Acα2-3)Galβ1-4Glc 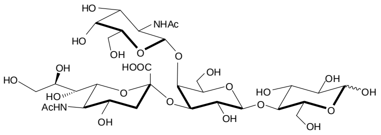	56
GalNAcβ1-4(Neu5Acα2-8Neu5Acα2-3)Galβ1-4Glc 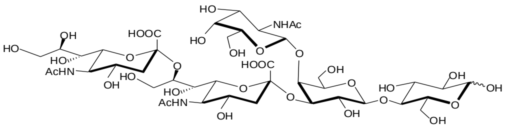	57
GalNAcβ1-4(Neu5Acα2-8Neu5Acα2-8Neu5Acα2-3)Galβ1-4Glc 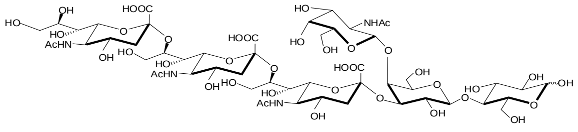	58
Galβ1-3GalNAcβ1-4(Neu5Acα2-3)Galβ1-4Glc 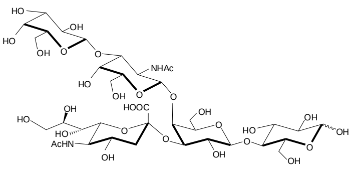	59

Neu5Acα2-3Galβ1-3GalNAcβ1-4Galβ1-4Glc 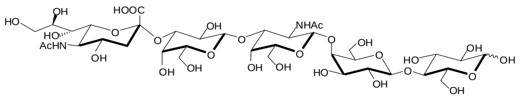	60
Neu5Acα2-3Galβ1-3GalNAcβ1-4(Neu5Acα2-3)Galβ1-4Glc 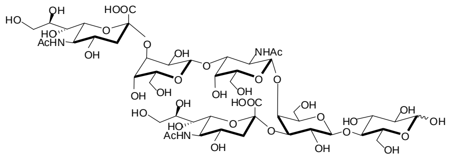	61
Galβ1-3GalNAcβ1-4(Neu5Acα2-8Neu5Acα2-3)Galβ1-4Glc 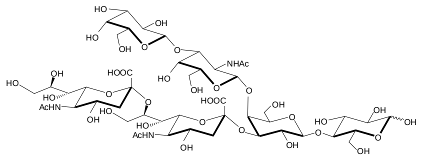	62
Neu5Acα2-8Neu5Acα2-3Galβ1-3GalNAcβ1-4(Neu5Acα2-3)Galβ1-4Glc 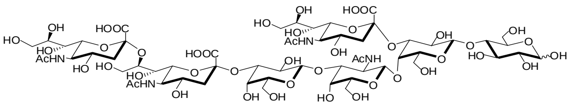	63
Galβ1-3GalNAcβ1-4(Neu5Acα2-8Neu5Acα2-8Neu5Acα2-3)Galβ1-4Glc 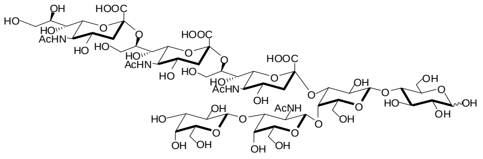	64
Galβ1-3GalNAcβ1-4Galβ1-4Glc 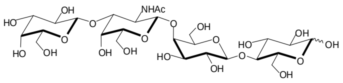	65
Fucα1-2Galβ1-3GalNAcβ1-4(Neu5Acα2-3)Galβ1-4Glc 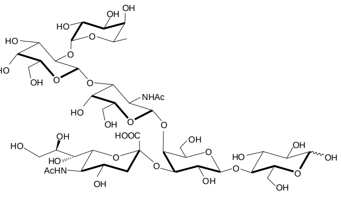	66
GalNAcβ1-4Galβ1-4Glc 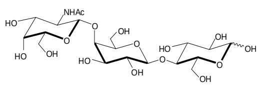	67
Galβ1-4(Neu5Acα2-3)Galβ1-4Glc 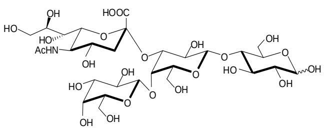	68
Galα1-4Galβ1-4Glc 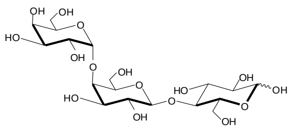	69

GalNAcβ1-3Galα1-4Galβ1-4Glc 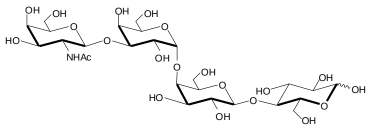	70
Galβ1-3GalNAcβ1-3Galα1-4Galβ1-4Glc 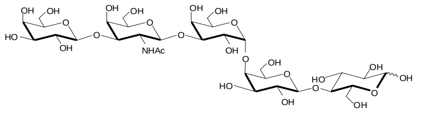	71
Fucα1-2Galβ1-3GalNAcβ1-3Galα1-4Galβ1-4Glc 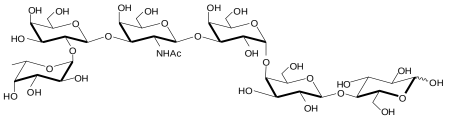	72
GalNAcα1-3(Fucα1-2)Galβ1-3GalNAcβ1-3Galα1-4Galβ1-4Glc 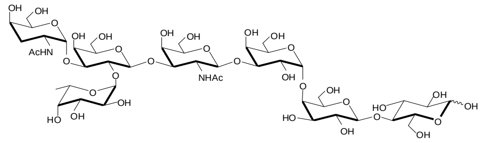	73
Galα1-3(Fucα1-2)Galβ1-3GalNAcβ1-3Galα1-4Galβ1-4Glc 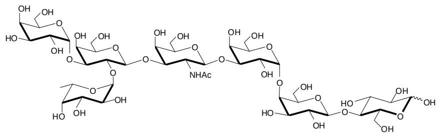	74
Galβ1-3GalNAc β1-3Gal 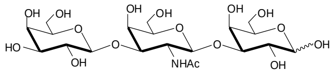	75
Fucα1-2Galβ1-3GalNAcβ1-3Gal 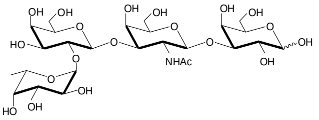	76
GalNAcα1-3(Fucα1-2)Galβ1-3GalNAcβ1-3Gal 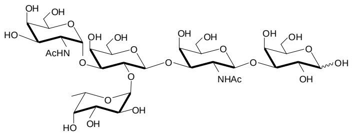	77
Galα1-3(Fucα1-2)Galβ1-3GalNAcβ1-3Gal 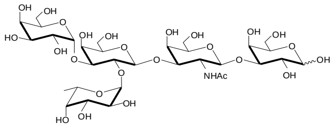	78
Neu5Acα2-3Galβ1-3GalNAcβ1-3Gal 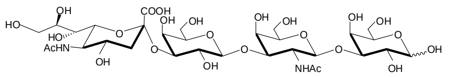	79

Neu5Acα2-3Galβ1-3GalNAcβ1-3Galα1-4Galβ1-4Glc 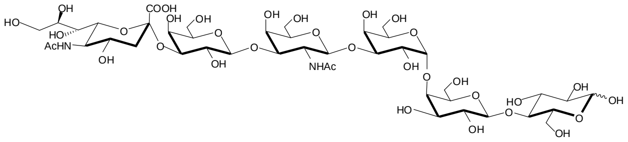	80
GalNAcα1-3GalNAcβ1-3Galα1-4Galβ1-4Glc 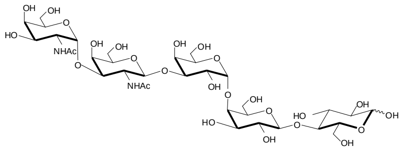	81
GalNAcα1-3GalNAcβ1-3Gal 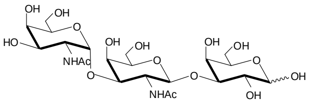	82
GalNAcα1-3GalNAcβ1-3Galα1-3Galβ1-4Glc 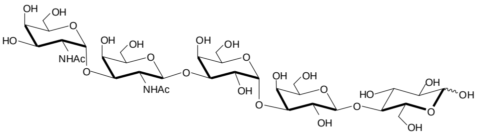	83
GalNAcβ1-3Gal 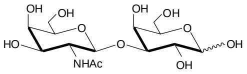	84
Galα1-3Gal 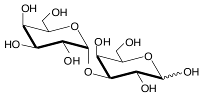	85
Galα1-4Gal 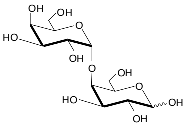	86
GlcNAcβ1-3Gal 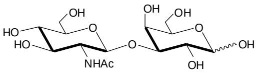	87
Neu5Acα2-3Gal 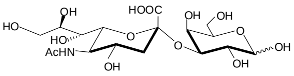	88
Galβ1-4Glcβ1-1βGal 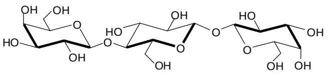	89
Fucα1-2Galβ1-4Glcβ1-1βGalα2-1Fuc 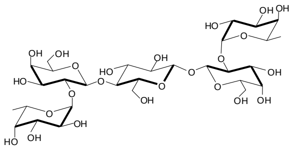	90

Neu5Acα2-3Galβ1-4Glcβ1-1βGalα2-3Neu5Ac 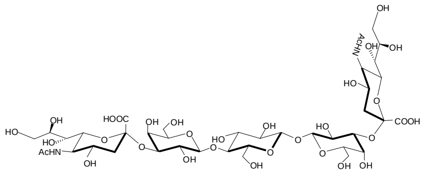	91
Fucα1-2Galβ1-4(Fucα1-2)Glc 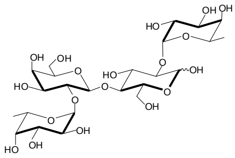	92
GalNAcα1-3(Fucα1-2)Galβ1-4(Fucα1-2)Glc 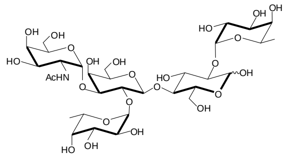	93
Galα1-3(Fucα1-2)Galβ1-4(Fucα1-2)Glc 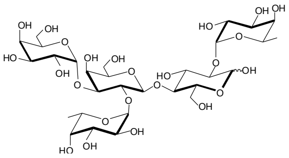	94
Neu5Acα2-3Galα1-4Galβ1-4Glc 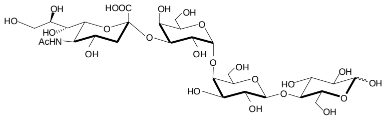	95
NeuAcα2-8Neu5Acα2-3Galβ1-3GlcNAcβ1-3Gal 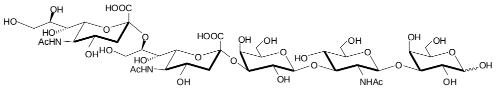	96
Galβ1-3GalNAcβ1-4Galβ1-3GalNAcβ1-4Galβ1-4Glc 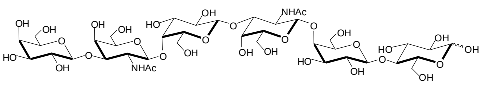	97
GlcAβ1-3Galβ1-4Glc 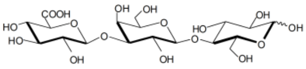	98
GlcAβ1-3Galβ1-3GlcNAcβ1-3Galβ1-4Glc 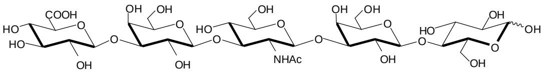	99
Galβ1-3GlcNAcβ1-3Galα1-4Galβ1-4Glc 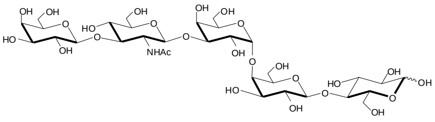	100

Galβ1-4GlcNAcβ1-3Galα1-4Galβ1-4Glc<br>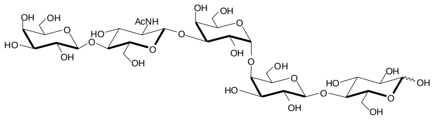	101
NeuAcα2-3Galβ1-3GlcNAcβ1-3Galα1-4Galβ1-4Glc<br>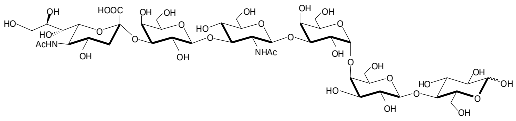	102
Fucα1-2Galβ1-3GlcNAcβ1-3Galα1-3Galβ1-4Glc<br>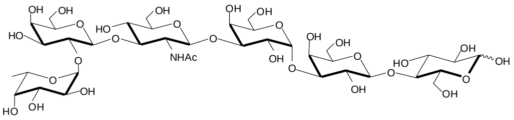	103
L-guluronate linked α(1-4), DP ≈ 20 ; M/G ratio<0.25<br>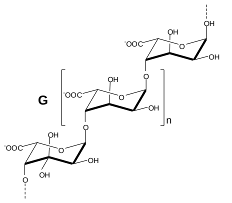	104
L-guluronate linked α(1-4), DP ≈ 10 ; M/G ratio<0.25	105
L-guluronate linked α(1-4), DP ≈ 5 ; M/G ratio<0.25	106
L-guluronate linked α(1-4), DP ≈ 3 ; M/G ratio<0.25	107
D-mannuronate linked β(1-4), DP ≈ 20, M/G ratio>4<br>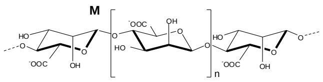	108
D-mannuronate linked β(1-4), DP ≈ 10, M/G ratio>4	109
D-mannuronate linked β(1-4), DP ≈ 5, M/G ratio>4	110
D-mannuronate linked β(1-4), DP ≈ 3, M/G ratio>4	111
D-mannuronate linked β(1-4), DP ≈ 30-50, M/G ratio>4	112
D-mannuronate linked β(1-4), DP ≈ 20-35, M/G ratio>4	113
D-galacturonate linked α(1-4), DP ≈ 25<br>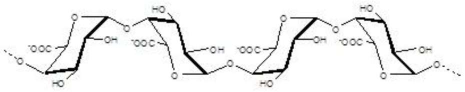	114
D-galacturonate linked α(1-4), DP = 3/4	115
D-galacturonate linked α(1-4), DP = 7/8	116

<table>
<tr><td>Oligosaccharides with approx. 80 % GXLF
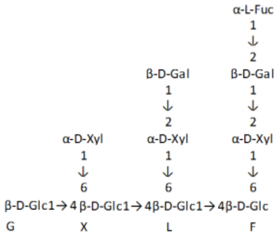
</td><td>117</td></tr>
<tr><td>Oligosaccharides with approx. 80 % XFG
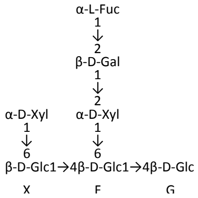
</td><td>118</td></tr>
<tr><td>Oligosaccharides with approx. 60 % XXFG
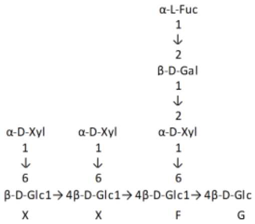
</td><td>119</td></tr>
<tr><td>Oligosaccharides with approx. 60 % XLFG
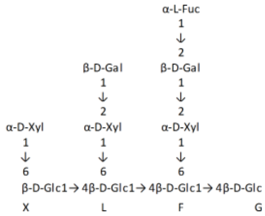
</td><td>120</td></tr>
<tr><td>Glcβ1-4Glc
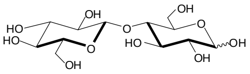</td><td>121</td></tr>
<tr><td>Glcβ1-4Glcβ1-4Glc
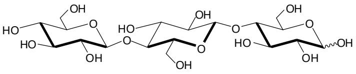</td><td>122</td></tr>
<tr><td>Glcβ1-4Glcβ1-4Glcβ1-4Glc
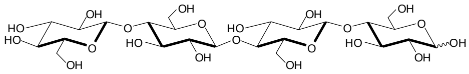</td><td>123</td></tr>
<tr><td>[Glcβ1-4Glcβ1-4]2Glc
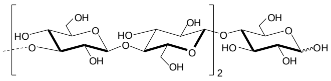</td><td>124</td></tr>
<tr><td>Glcβ1-4[Glcβ1-4Glcβ1-4]2Glc
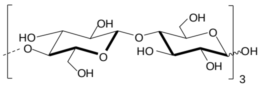</td><td>125</td></tr>
</table>

Glcα1-4Glc 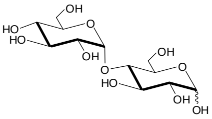	126
Glcα1-4Glcα1-4Glc 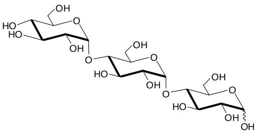	127
Glcα1-4Glcα1-4Glcα1-4Glc 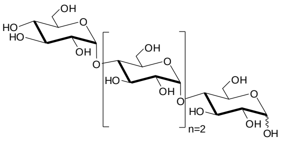	128
[Glcα1-4Glcα1-4]2Glc 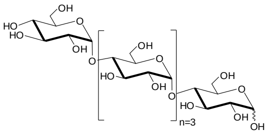	129
Glcα1-4[Glcα1-4Glcα1-4]2Glc 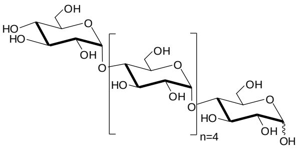	130
[Glcα1-4Glcα1-4Glcα1-4]2Glc 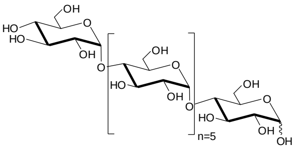	131
GlcNAcβ1-4GlcNAc 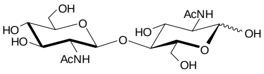	132
GlcNAcβ1-4GlcNAcβ1-4GlcNAc 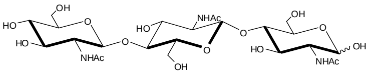	133
GlcNAcβ1-4GlcNAcβ1-4GlcNAcβ1-4GlcNAc 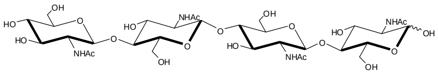	134
GlcNAcβ1-4[GlcNAcβ1-4]3Glc 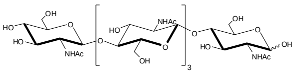	135

GlcNAcβ1-4[GlcNAcβ1-4GlcNAcβ1-4]2GlcNAc 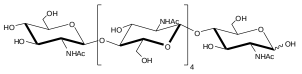	136
[Glcβ1-6Glc]n, DP ≤ 10 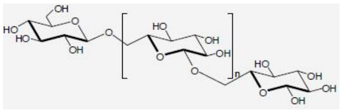	137
[Manβ1-4(Galα1-6)Man]n, Ratio Man/Gal ≈ 2:1 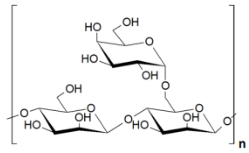	138
[Manβ1-4Manβ1-4(Galα1-6)Manβ1-4Man]n, Ratio Man/Gal ≈ 4:1 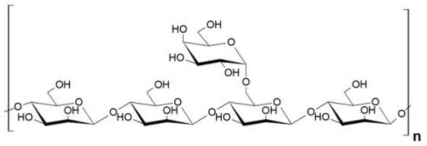	139
[(Galα1-6)Manβ1-4[(Galα1-6)Man]n, Ratio Man/Gal ≈ 1:1 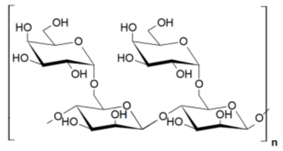	140
[Manβ1-4 Manβ1-4(Galα1-6)Manβ1-4Man]n, Ratio Man/Gal ≈ 4:1, DP ≈ 3. 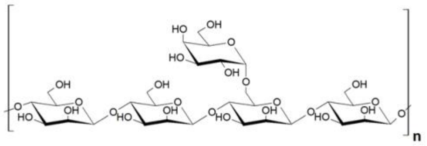	141
[Manβ1-4 Manβ1-4(Galα1-6)Manβ1-4Man]n, Ratio Man/Gal ≈ 4:1, DP ≈ 5. 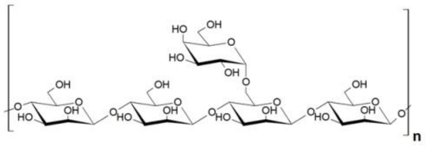	142
[Manβ1-4 Manβ1-4(Galα1-6)Manβ1-4Man]n, Ratio Man/Gal ≈ 4:1, DP ≈ 9. 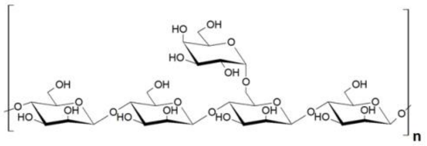	143

Manβ1-4(Galα1-6)Manβ1-4(Galα1-6)Manβ1-4Manβ1-4Man<br>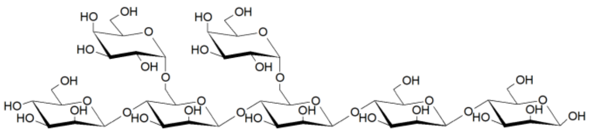	144
[Manβ1-4 Manβ1-4(Galα1-6)Manβ1-4Man]n, Ratio Man/Gal ≈ 4:1, DP = 3.<br>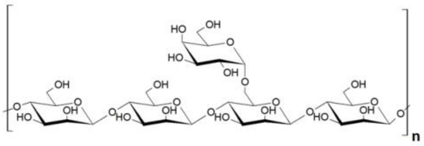	145
Manβ1-4Manβ1-4(Galα1-6)Man<br>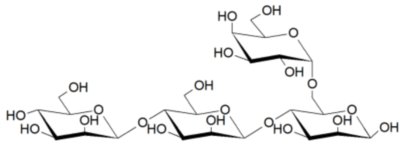	146
Typical polysaccharide motifs:<br>[Glcβ1-4GlcAcβ1-4Manβ1-4Man]n, Ratio Man/Glc ≈ 1/5<br>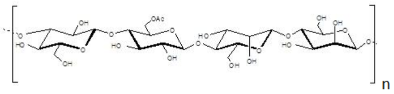	147
Typical polysaccharide motifs:<br>[Glcβ1-4GlcAcβ1-4Manβ1-4Man]n<br>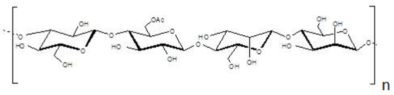	148
D-mannose linked β(1-4) (coconuts).<br>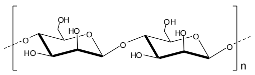	149
D-mannose linked β(1-4) partially O-acetylated (*Aloe vera*)	150
D-mannose polysaccharide linked α(1-6) and highly branched with α(1-2) and α(1-3) D-mannose (Yeast).	151
D-mannose linked β(1-4), DP = 2 to 24	152
Oligosaccharides MW between 3 and 5 kDa (*Ulva armoricana*)	153
Oligosaccharides MW between 1 and 3 kDa (*Ulva armoricana*)	154
Oligosaccharides MW between 0.65 and 1 kDa (*Ulva armoricana*)	155
Oligosaccharides with DP MW 2 to 25 (*Palmaria palmata*)<br>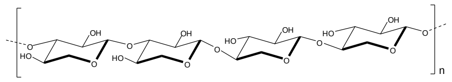	156

S2 Results of the competitive screening of the 156 glycans toward LecB. Only oligasaccharides having a grade better than E are presented

Family	Sub Family	Structure	Grade	Id
ABO blood ag	H antigenes	Fucα1-2Gal	C	9
		Fucα1-2Galβ1-4Glc	C	10
		Fucα1-2Galβ1-3GlcNAcβ1-3Gal	D	11
		Fucα1-2Galβ1-4GlcNAcβ1-3Gal	D	12
		Fucα1-2Galβ1-3GlcNAcβ1-3Galβ1-4Glc	D	13
		Fucα1-2Galβ1-4GlcNAcβ1-3Galβ1-4Glc	C	14
		Fucα1-2Galβ1-3GalNAcβ1-3Gal	C	76
	A antigenes	GalNAcα1-3(Fucα1-2)Gal	D	15
		GalNAcα1-3(Fucα1-2)Galβ1-3GalNAcβ1-3Gal	D	77
Lewis ag	Le^X antigenes	Galβ1-4(Fucα1-3)GlcNAcβ1-3Gal	C	26
		Galβ1-4(Fucα1-3)GlcNAcβ1-3Galβ1-4(Fucα1-3)Glc	B	27
	Le^Y antigenes	Fucα1-2Galβ1-4(Fucα1-3)GlcNAcβ1-3Gal	C	28
	sLe^X antigenes	Neu5Acα2-3Galβ1-4(Fucα1-3)GlcNAcβ1-3Gal	C	29
	Le^A antigenes	Galβ1-3(Fucα1-4)GlcNAcβ1-3Gal	A	30
		Galβ1-3(Fucα1-4)GlcNAcβ1-3Galβ1-4(Fucα1-3)Glc	A	31
	Le^B antigenes	Fucα1-2Galβ1-3(Fucα1-4)GlcNAcβ1-3Gal	C	32

**Fucosylated oligosacch.**	"3 fucosyl lactose core"	Galβ1-4(Fucα1-3)Glc	B	33
		Galβ1-4GlcNAcβ1-3Galβ1-4(Fucα1-3)Glc	B	34
		Galβ1-3GlcNAcβ1-3Galβ1-4(Fucα1-3)Glc	B	35
		Neu5Acα2-3Galβ1-4(Fucα1-3)Glc	B	36
		Galα1-3Galβ1-4(Fucα1-3)Glc	B	37
	" 2’3 difucosyl lactose core"	Fucα1-2Galβ1-4(Fucα1-3)Glc	C	38
		GalNAcα1-3(Fucα1-2)Galβ1-4(Fucα1-3)Glc	D	39
		Galα1-3(Fucα1-2)Galβ1-4(Fucα1-3)Glc	D	40
	" 2’2 difucosyl lactose core"	Fucα1-2Galβ1-4(Fucα1-2)Glc	C	92
		GalNAcα1-3(Fucα1-2)Galβ1-4(Fucα1-2)Glc	D	93
		Galα1-3(Fucα1-2)Galβ1-4(Fucα1-2)Glc	D	94
**Misc.**	Fuc-GM1	Fucα1-2Galβ1-3GalNAcβ1-4(Neu5Acα2-3)Galβ1-4Glc	D	66
	difucosyl-pentaose	Fucα1-2Galβ1-4Glcβ1-1βGalα2-1Fuc	C	90
	Globo-H	Fucα1-2Galβ1-3GalNAcβ1-3Galα1-4Galβ1-4Glc	C	72
	iGbH analogue	Fucα1-2Galβ1-3GlcNAcβ1-3Galα1-3Galβ1-4Glc	D	103

**Glucan**	Xyloglucan	Main motifs are GXLF (80 %)	C	117
		Main motifs are XFG (80%)	D	118
		Main motifs are XXFG (60%)	D	119
		Main motifs are XLFG (60%)	C	120
**Mannan**	Mannan	Scaffold consist of D-mannose residues linked α(1-6). D-Mannose side chains linked α(1-2) et α(1-3)	A	151

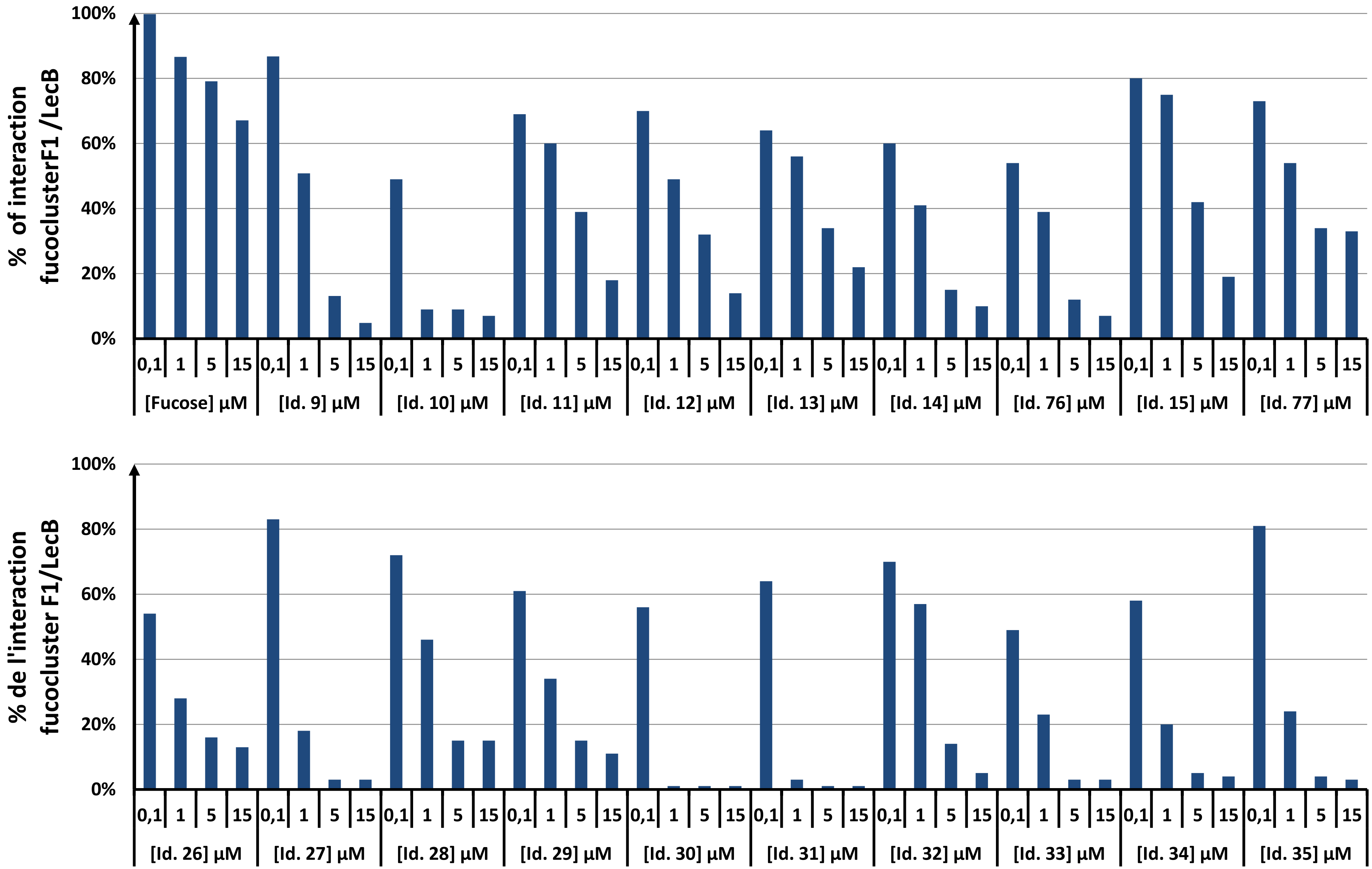

100%
80%
60%
40%
20%
0%
% of interaction fucoclusterF1 /LecB
0,1
1
5
15
[Fucose] µM
[Id. 9] µM
[Id. 10] µM
[Id. 11] µM
[Id. 12] µM
[Id. 13] µM
[Id. 14] µM
[Id. 76] µM
[Id. 15] µM
[Id. 77] µM
% de l'interaction fucocluster F1/LecB
[Id. 26] µM
[Id. 27] µM
[Id. 28] µM
[Id. 29] µM
[Id. 30] µM
[Id. 31] µM
[Id. 32] µM
[Id. 33] µM
[Id. 34] µM
[Id. 35] µM

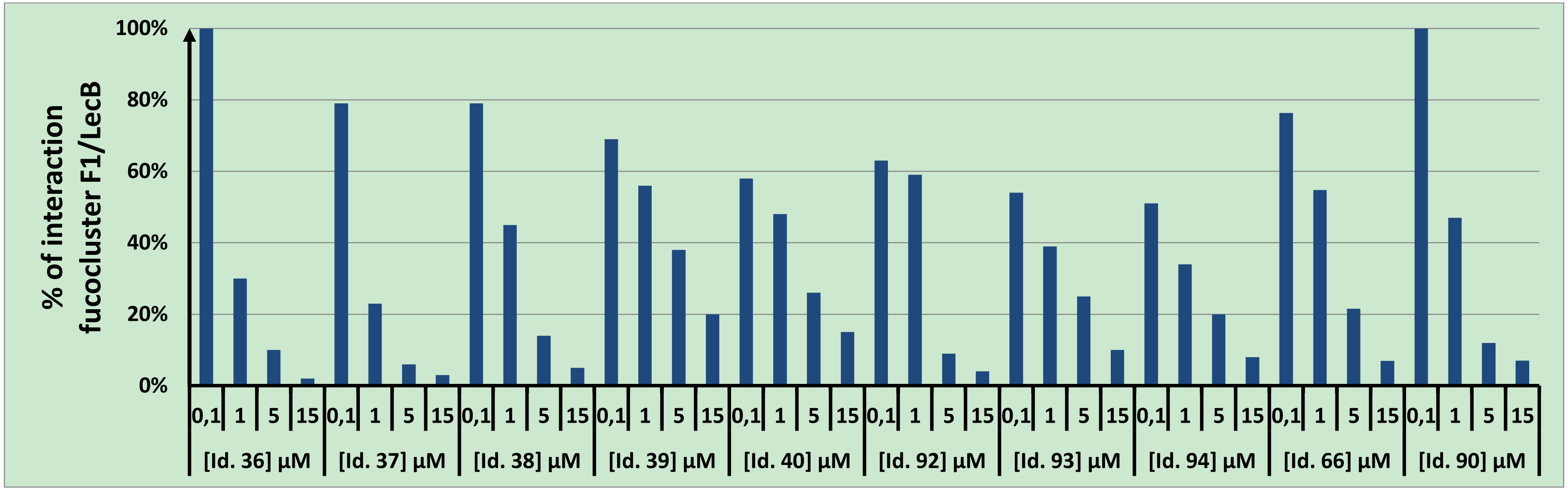
% of interaction fucocluster F1/LecB
100%
80%
60%
40%
20%
0%
0,1 1 5 15
[Id. 36] µM
[Id. 37] µM
[Id. 38] µM
[Id. 39] µM
[Id. 40] µM
[Id. 92] µM
[Id. 93] µM
[Id. 94] µM
[Id. 66] µM
[Id. 90] µM

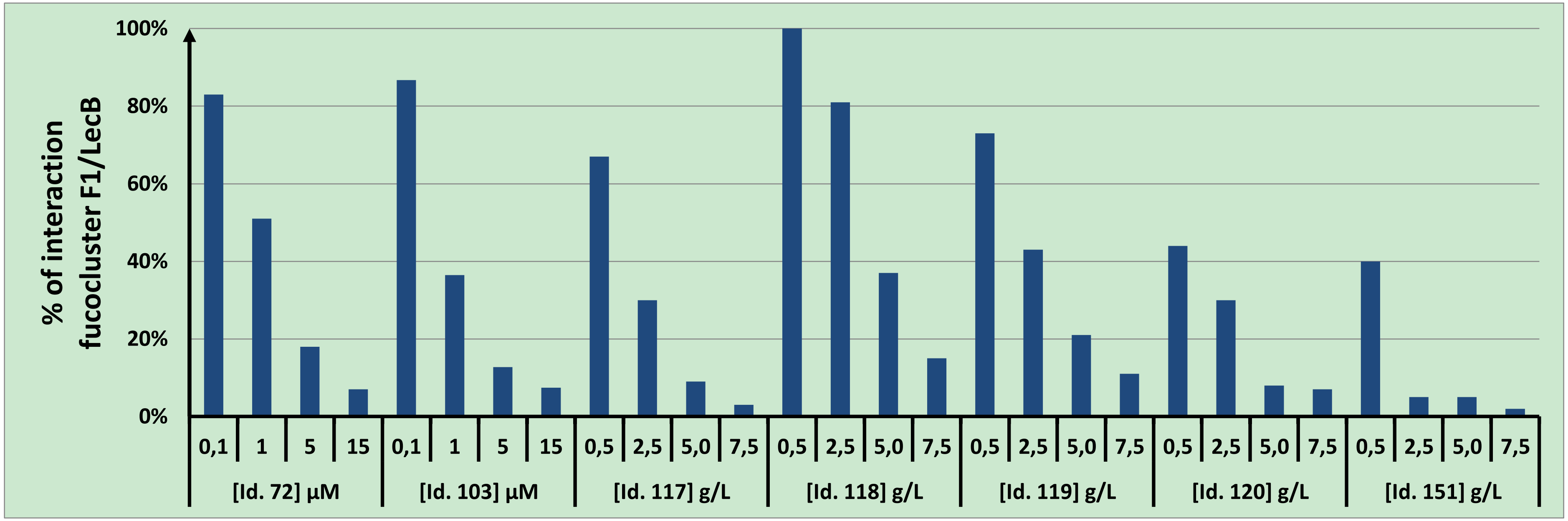
% of interaction fucocluster F1/LecB
100%
80%
60%
40%
20%
0%
0,1 1 5 15
0,5 2,5 5,0 7,5
[Id. 72] µM
[Id. 103] µM
[Id. 117] g/L
[Id. 118] g/L
[Id. 119] g/L
[Id. 120] g/L
[Id. 151] g/L

S3 NMR spectra of compounds 165a and 165b.

165a

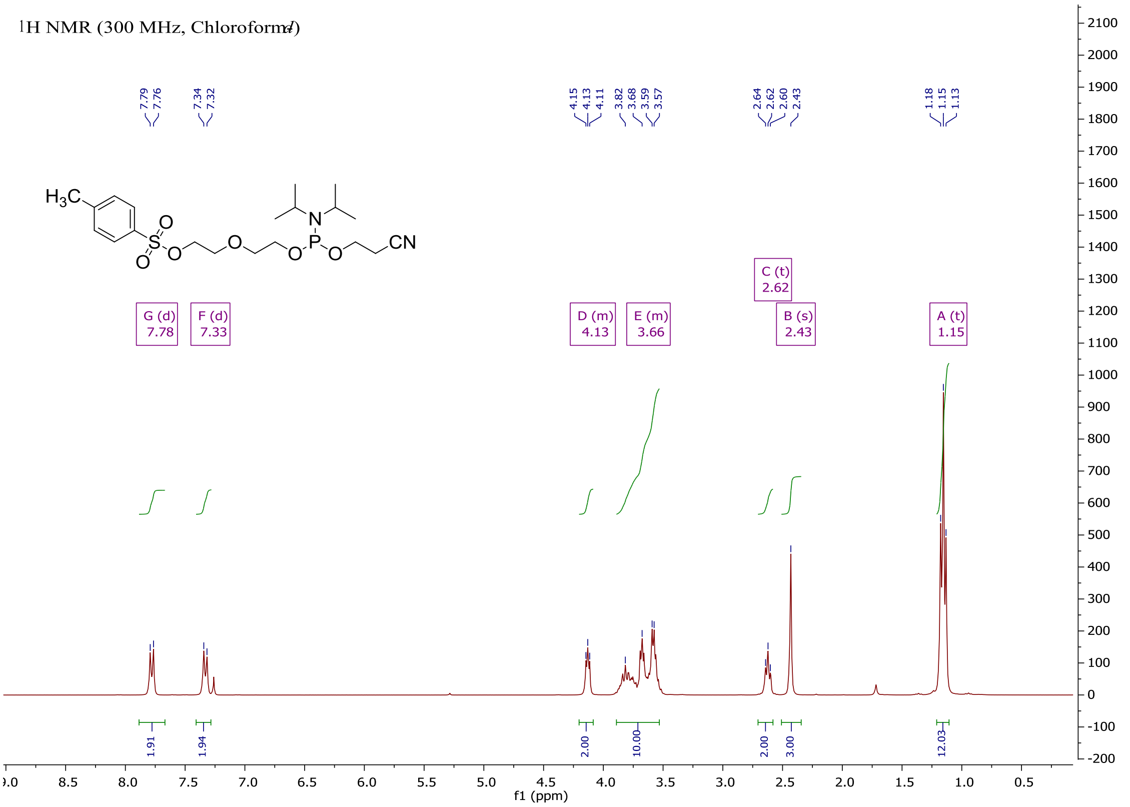

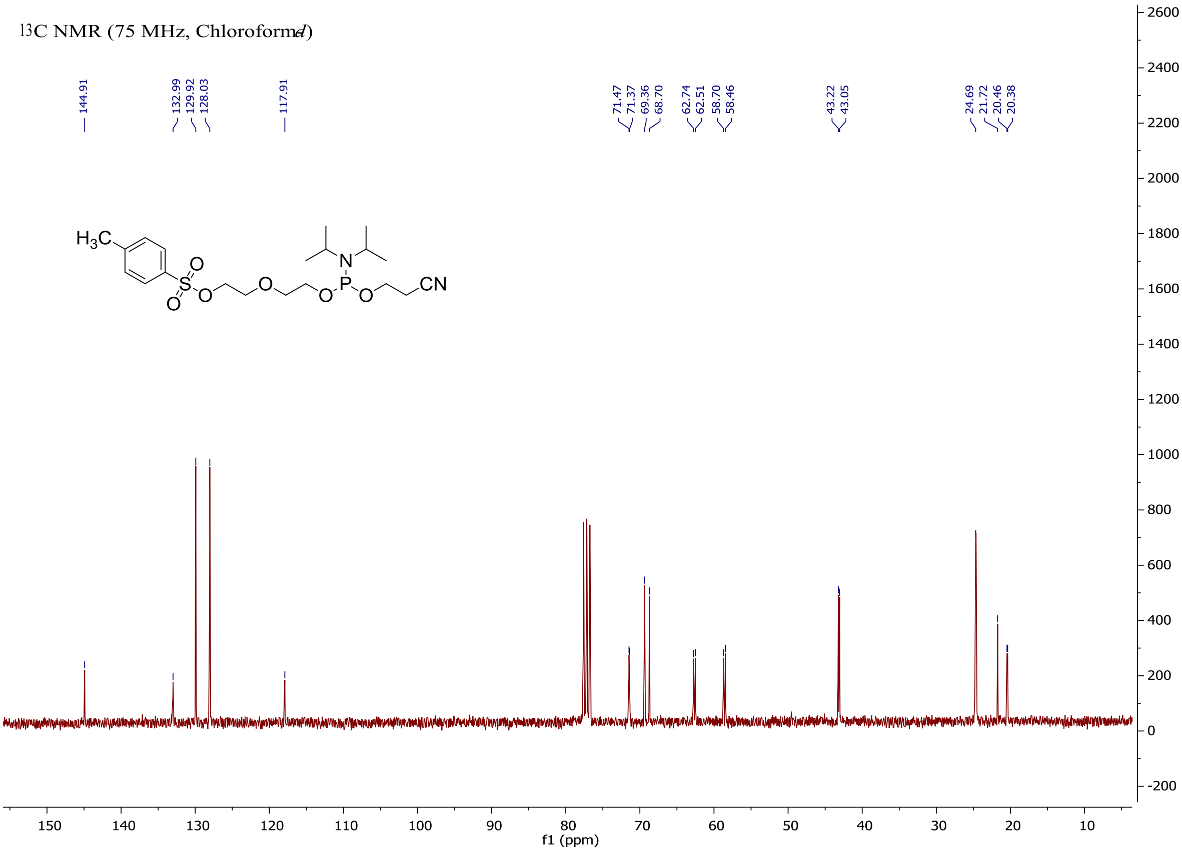

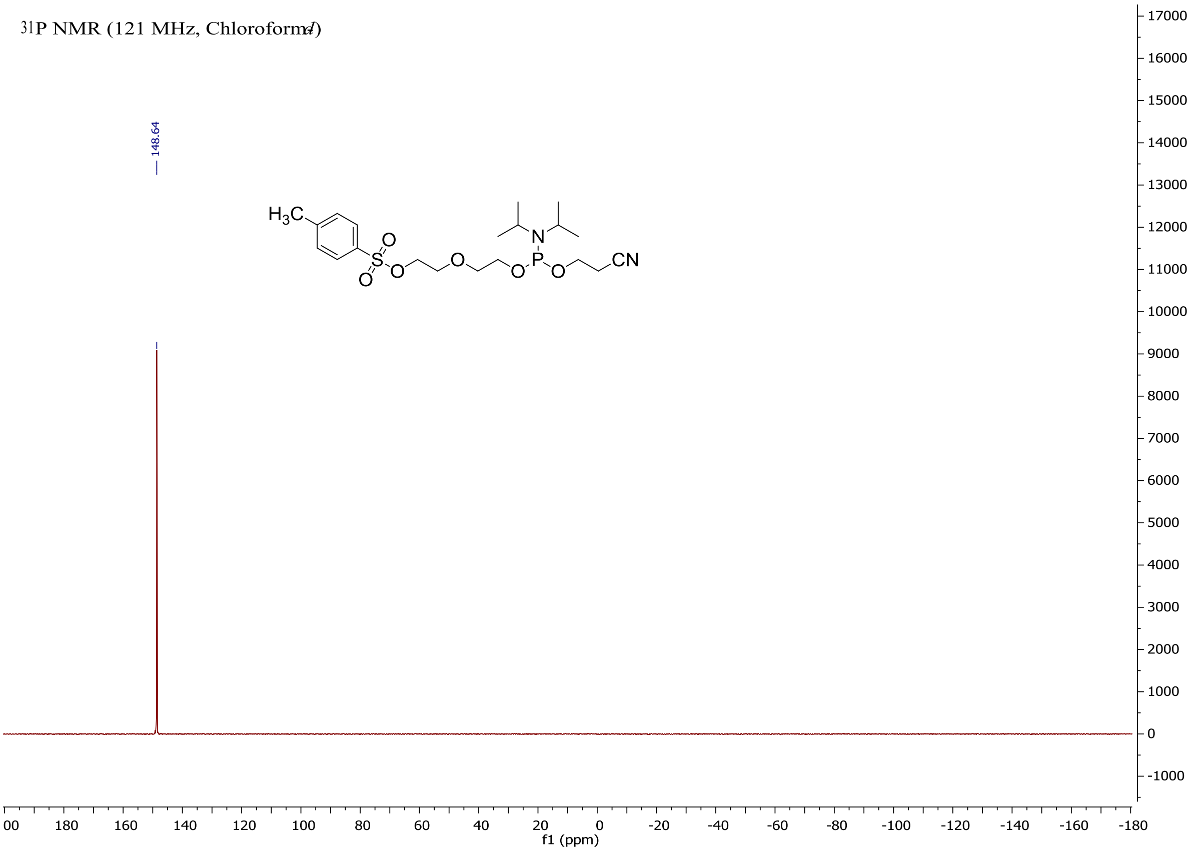
31P NMR (121 MHz, Chloroform-d)
148.64
H_3C
O
S
O
O
O
O
N
P
O
CN
f1 (ppm)

165b

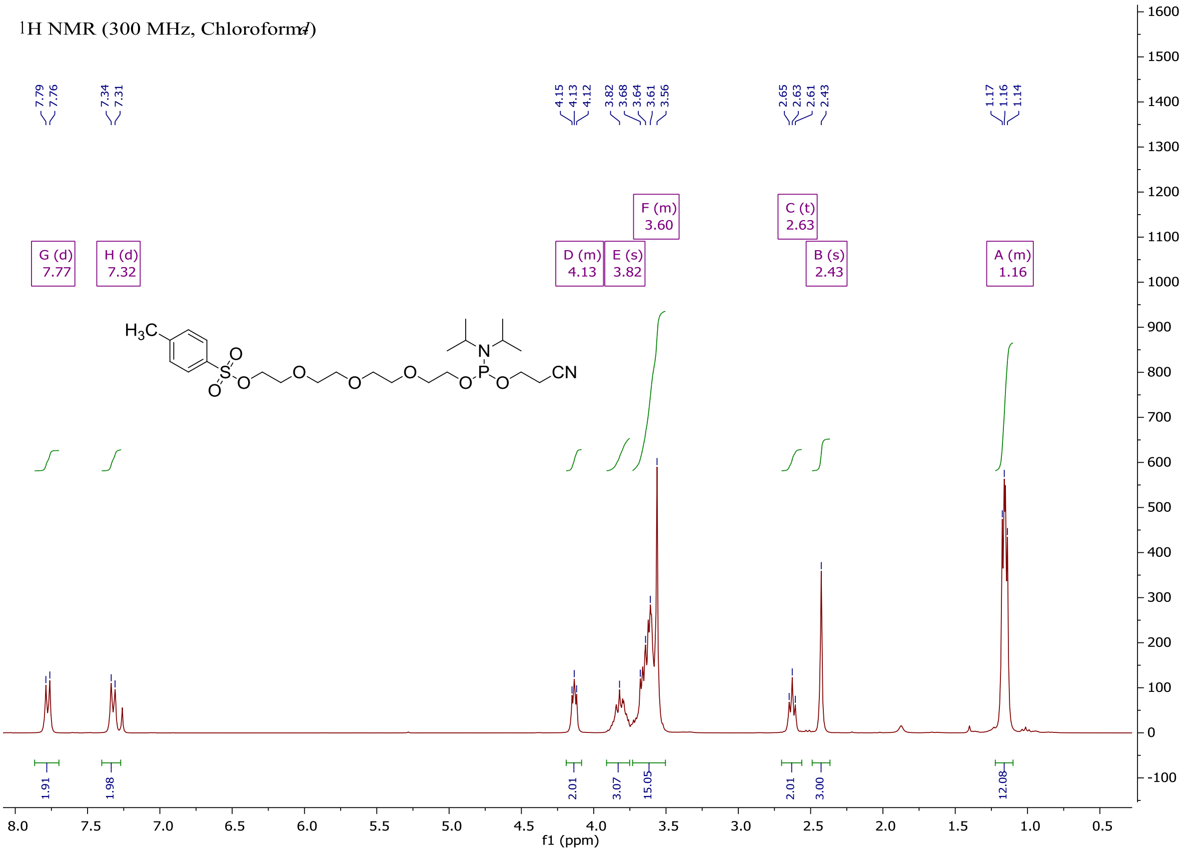
1H NMR (300 MHz, Chloroform-d)
7.79
7.76
7.34
7.31
4.15
4.13
4.12
3.82
3.68
3.64
3.61
3.56
2.65
2.63
2.61
2.43
1.17
1.16
1.14
G (d)
7.77
H (d)
7.32
D (m)
4.13
E (s)
3.82
F (m)
3.60
C (t)
2.63
B (s)
2.43
A (m)
1.16
H_3C
CN
1.91
1.98
2.01
3.07
15.05
2.01
3.00
12.08
f1 (ppm)

13C NMR (75 MHz, Chloroform-d)

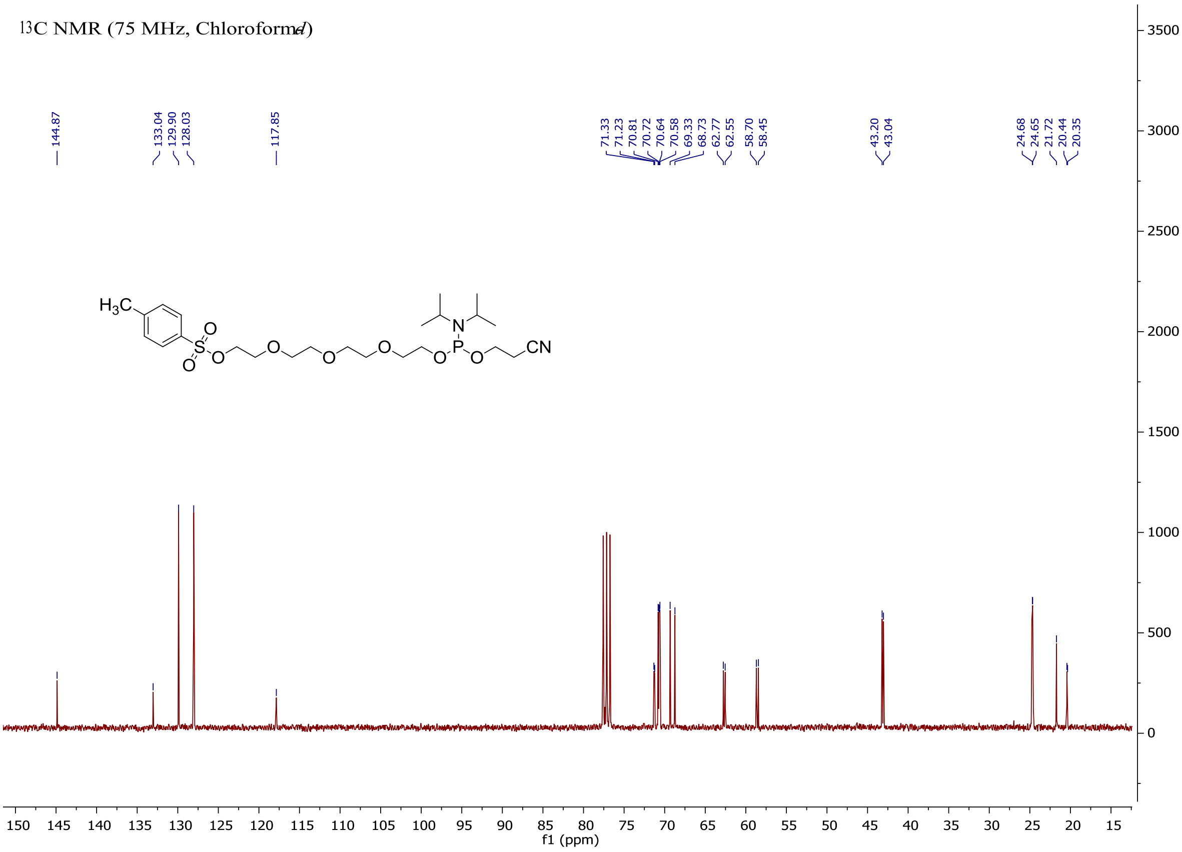

31P NMR (121 MHz, Chloroform-d)

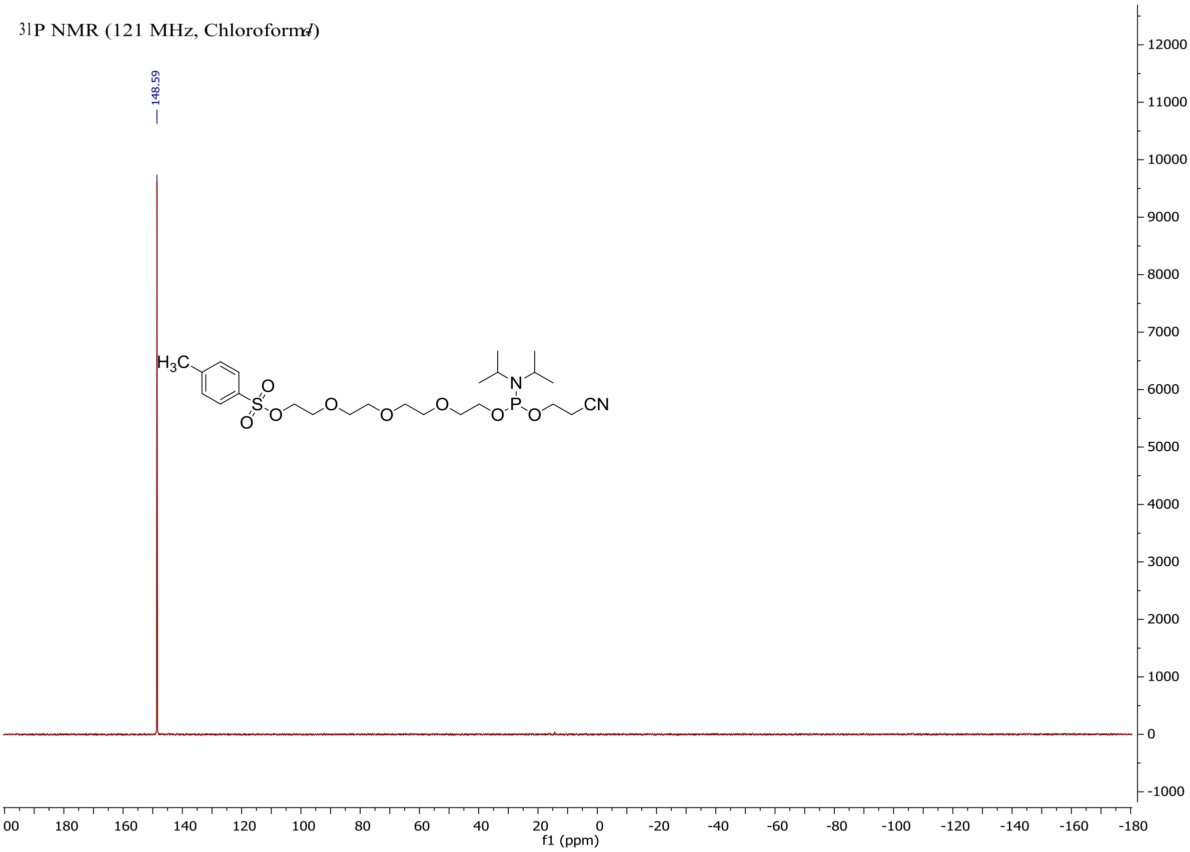

S4 characterization for propargylated oligosaccharides: Compounds 166 to 170

Compound 166: Lewis^{a} tetraose-NAc-propargyl - Galβ1-3(Fucα1-4)GlcNAcβ1-3Gal-NAc-CH2-CΞCH

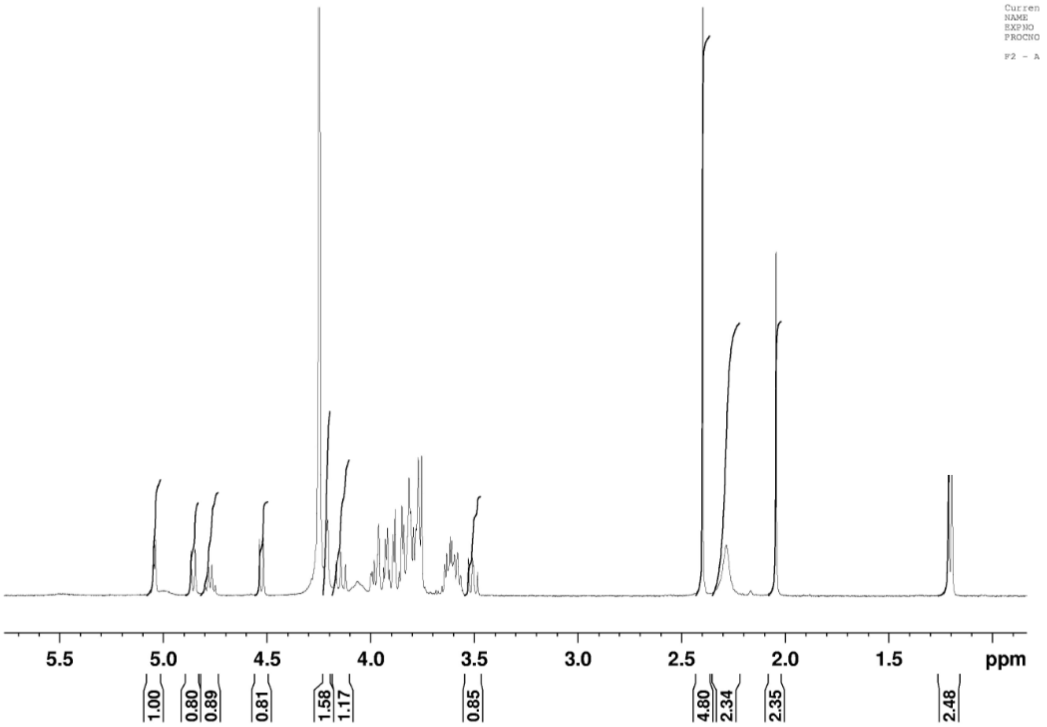

^{1}H-NMR spectrum

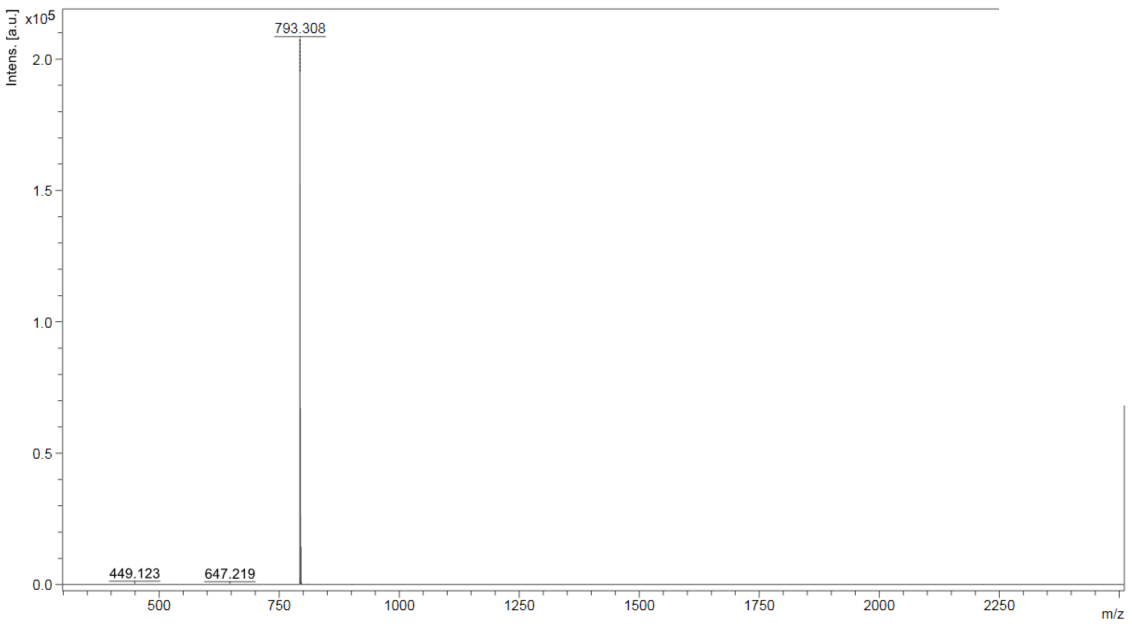

MALDI-TOF positive mode: M=770, m/z=793.282 [M+Na]$^+$

<u>Compound 167 : Lewis^b pentaose-N-acetyl-propargyl - Fucα1-2Galβ1-3(Fucα1-4)GlcNAcβ1-3Gal-NAc-CH[illegible]
CΞCH</u>

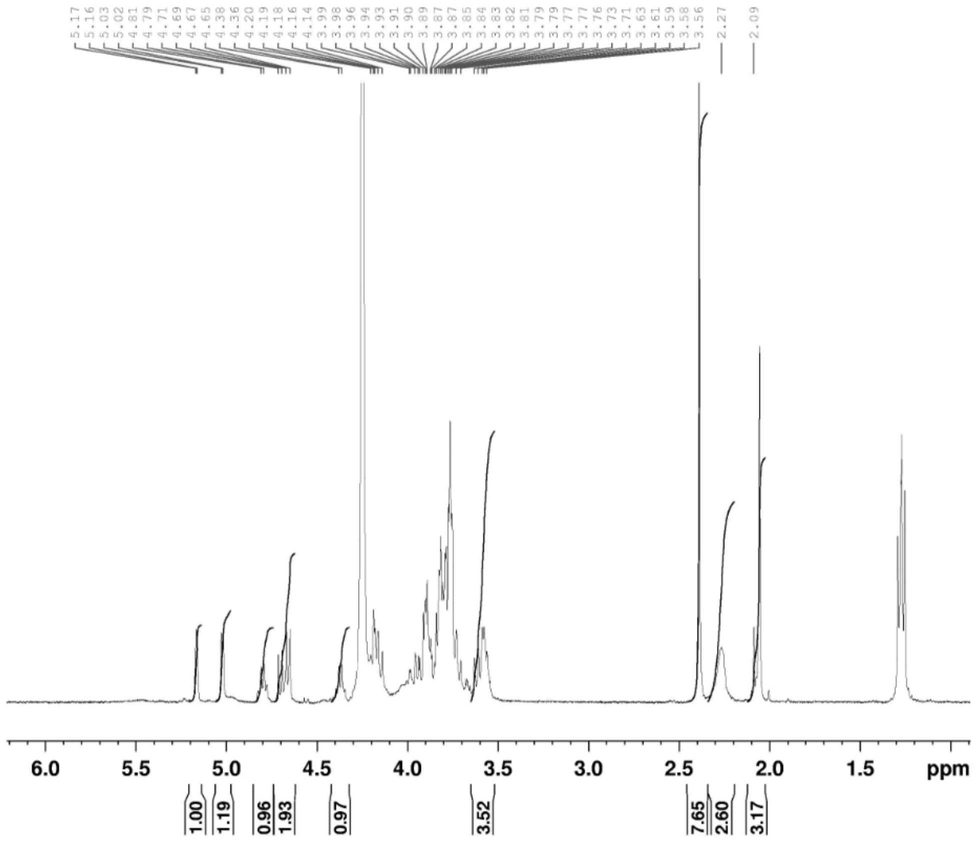

^{1}H-NMR spectrum

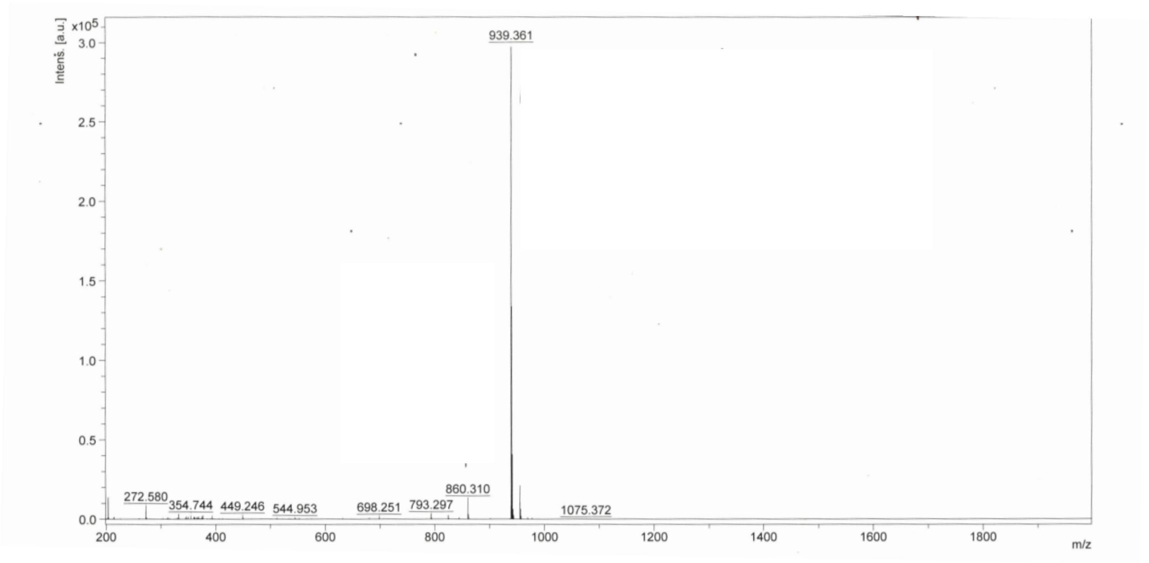

MALDI-TOF positive mode: M=916, m/z=939.361 [M+Na]$^+$

<u>Compound 168 : 3 fucosyllactose-NAc-propargyl - Galβ1-4(Fucα1-3)Glc-NAc-CH2-CΞCH</u>

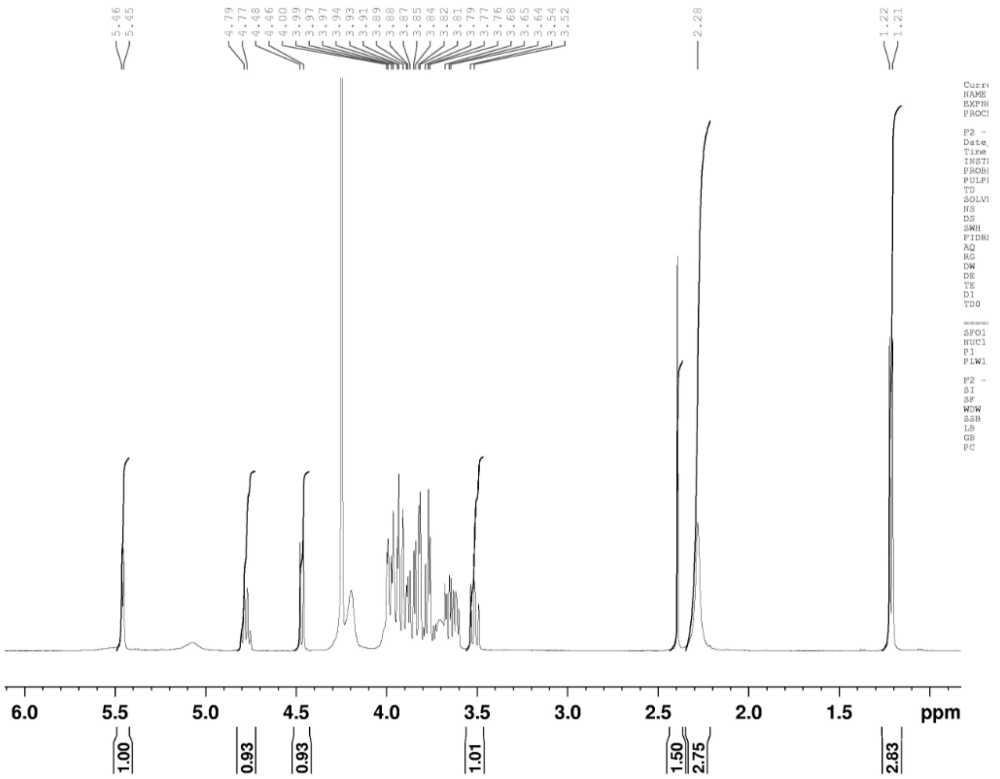

^{1}H-NMR spectrum

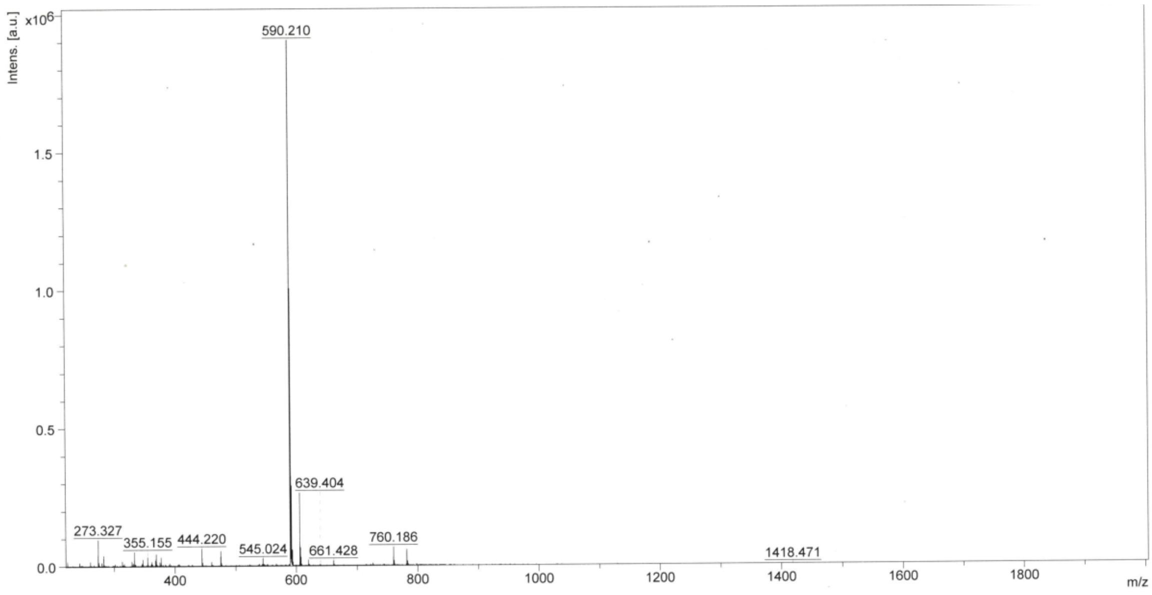

MALDI-TOF positive mode: M=567, m/z=590.210 $[M+Na]^+$

Compound 169 : Lewis^{x} tetraose-N-acetyl-propargyl - Galβ1-4(Fucα1-3)GlcNAcβ1-3Gal-NAc-CH2-CΞCH

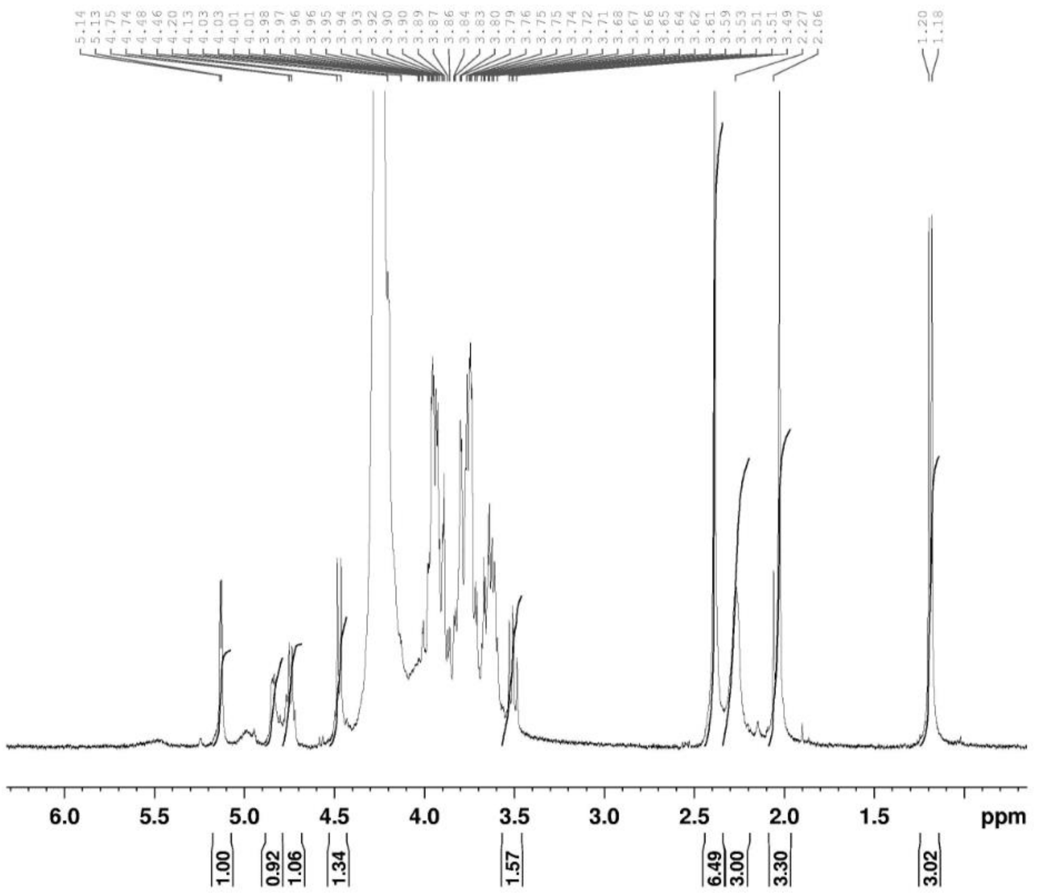

^{1}H-NMR spectrum

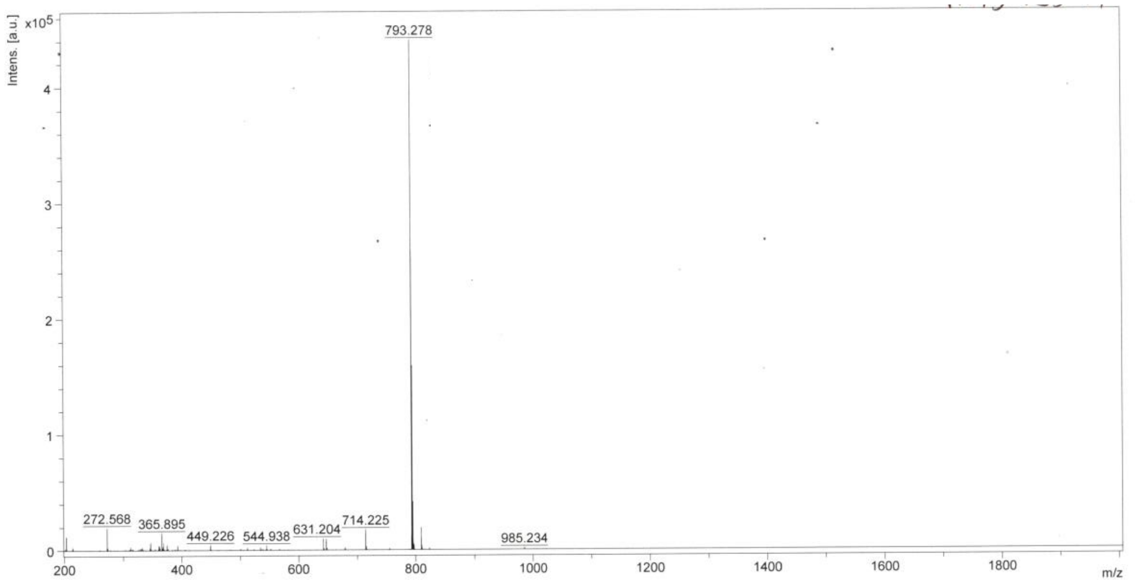

MALDI-TOF positive mode: M=770, m/z=793.278 [M+Na]$^{+}$

Compound 170 : Sialyl Lewis^{x} pentaose-N-acetyl-propargyl - Neu5α2-3AcGalβ1-4(Fucα1-3)GlcNAcβ1-3Ga
CH2-CΞCH

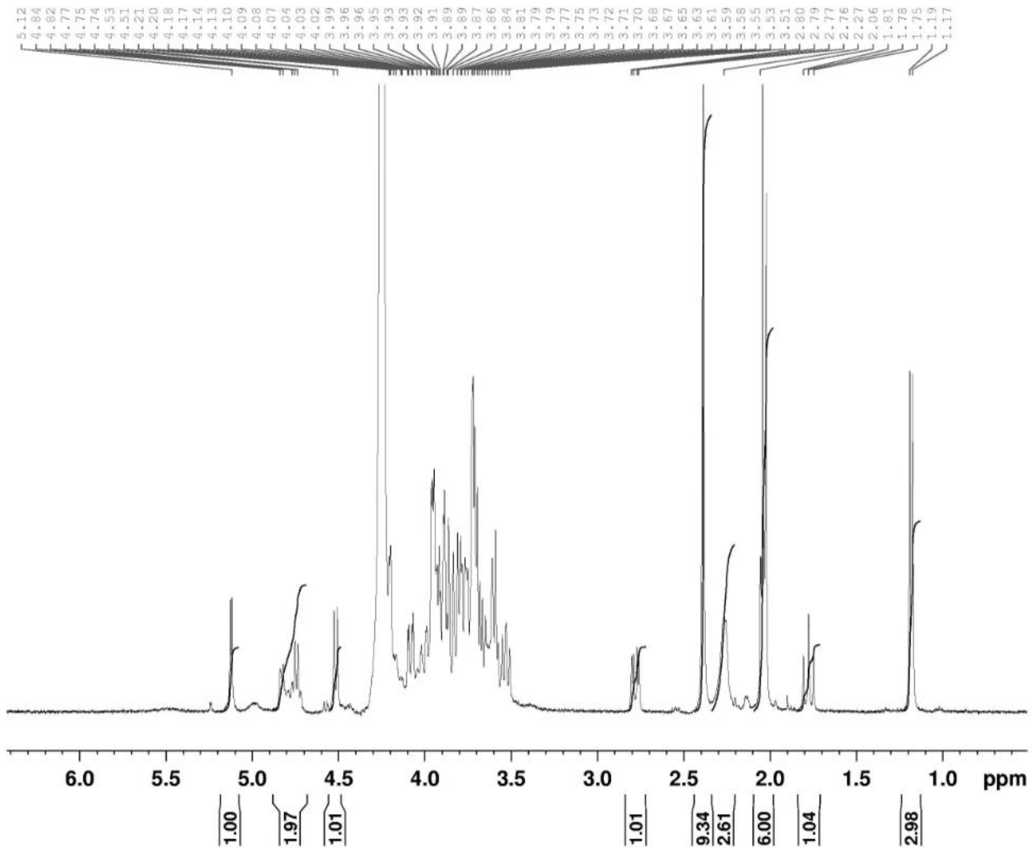

^{1}H-NMR spectrum

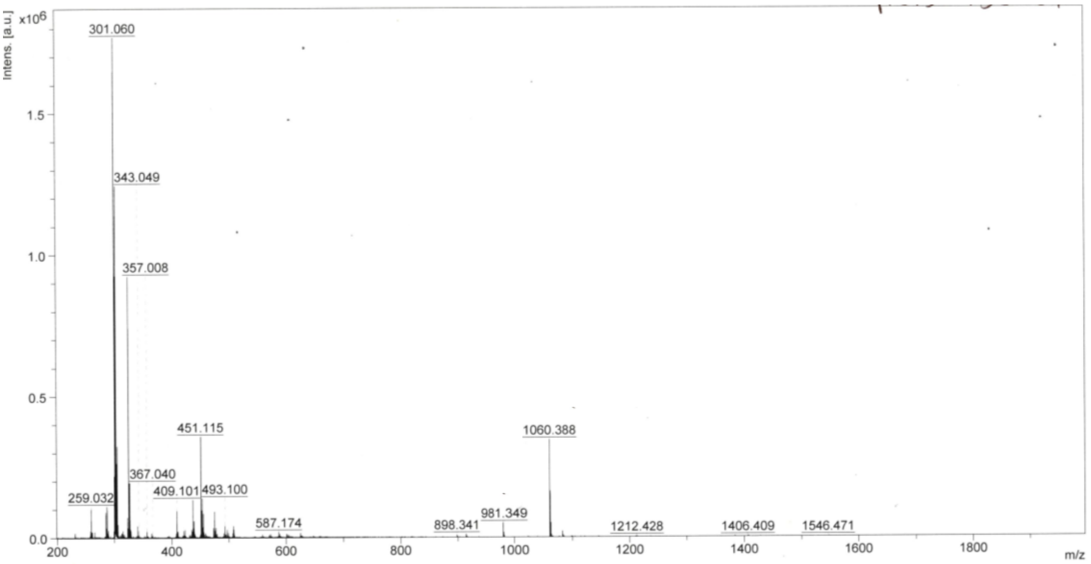

MALDI-TOF positive mode: M=1083, m/z=1060.388 $[M+Na]^+$

S5 Coupling conditions and characterization data for the 50 oligoglycoclusters and the 5 monovalent ligands.

compound	Core	linker	Sequence	Click Method	Amount (nmol)	MALDI-TOF MS Cald	[M-H]⁻ MS Found
M_LeA		Hex	2	A	10	6167.56	6167.56
M_LeB		Hex	2	A	12	6313.70	6312.91
M_3FL		Hex	5	A	6	5900.31	5900.22
M_LeX		Hex	2	A	8	6167.56	6168.88
M_SLeX		Hex	2	A	10	6458.81	6458.77
G1-LeA	Ara	EG_2	4	B	6	8506.51	8506.30
G1-LeB	Ara	EG_2	4	C	4	8944.94	8944.33
G1-3FL	Ara	EG_2	4	C	7	7896.94	7896.64
G1-LeX	Ara	EG_2	4	B	5	8506.51	8506.55
G2-LeA	Ara	EG_4	5	B	7	8770.83	8770.43
G2-LeB	Ara	EG_4	5	C	6	9209.25	9209.28
G2-3FL	Ara	EG_4	5	C	12	8161.25	8161.69
G2-LeX	Ara	EG_4	5	B	4	8770.83	8770.73
G3-LeA	Xylo	EG_2	2	B	5	8570.56	8570.12
G3-LeB	Xylo	EG_2	2	C	7	9008.98	9008.59
G3-3FL	Xylo	EG_2	2	C	9	7960.98	7960.67
G3-LeX	Xylo	EG_2	2	B	5	8570.56	8570.81
G4-LeA	Xylo	EG_4	3	B	8	8770.83	8769.89
G4-LeB	Xylo	EG_4	3	C	7	9209.25	9209.03
G4-3FL	Xylo	EG_4	3	C	11	8161.25	8161.60
G4-LeX	Xylo	EG_4	3	B	6	8770.83	8770.63
G5-LeA	Ribo	EG_2	1	B	4	8506.51	8506.91
G5-LeB	Ribo	EG_2	1	C	5	8944.94	8945.11
G5-3FL	Ribo	EG_2	1	C	5	7896.94	7896.15
G5-LeX	Ribo	EG_2	1	B	2	8506.51	8506.39
G6-LeA	Ribo	EG_4	3	B	13	8770.83	8770.96
G6-LeB	Ribo	EG_4	3	B	10	9209.25	9209.31
G6-3FL	Ribo	EG_4	6	B	5	8161.23	8161.38
G6-LeX	Ribo	EG_4	3	B	10	8770.83	8770.68
G6-SLeX	Ribo	EG_4	3	F	6	9644.58	9644.90
G7-LeA	Glc	EG_2	4	D	5	9406.30	9406.24

G7-LeB	Glc	EG_2	4	E	8	9990.86	9990.07
G7-3FL	Glc	EG_2	4	E	9	8593.53	8593.27
G7-LeX	Glc	EG_2	4	D	4	9406.30	9405.90
G8-LeA	Glc	EG_4	5	D	6	9758.72	9758.91
G8-LeB	Glc	EG_4	5	E	9	10343.28	10343.15
G8-3FL	Glc	EG_4	5	E	5	8945.95	8945.57
G8-LeX	Glc	EG_4	5	D	4	9758.72	9758.17
G9-LeA	Gal	EG_2	2	D		9470.34	9470.37
G9-LeB	Gal	EG_2	2	E	9	10054.91	10055.16
G9-3FL	Gal	EG_2	2	E	11	8657.57	8657.34
G9-LeX	Gal	EG_2	2	D	5	9470.34	9470.65
G10-LeA	Gal	EG_4	3	D	2	9758.72	9758.70
G10-LeB	Gal	EG_4	3	E	5	10343.28	10344.70
G10-3FL	Gal	EG_4	3	E	6	8945.96	8945.61
G10-LeX	Gal	EG_4	3	D	4	9758.72	9757.78
G11-LeA	Man	EG_2	1	D	4	9406.30	9407.10
G11-LeB	Man	EG_2	1	E	5	9990.86	9990.28
G11-3FL	Man	EG_2	1	E	5	8593.53	8593.78
G11-LeX	Man	EG_2	1	D	3	9406.30	9406.76
G12-LeA	Man	EG_4	4	D	9	9758.72	9758.30
G12-LeB	Man	EG_4	4	D	3	10343.28	10343.20
G12-3FL	Man	EG_4	7	D	9	8964.98	8964.56
G12-LeX	Man	EG_4	4	D	6	9758.72	9759.02
G12-SLeX	Man	EG_4	4	G	4	10923.72	10922.53

S6: HPLC chromatograms and MALDI-ToF spectra of oligoglycoclusters with 3-Fucosyl-Lactose (3-FL)

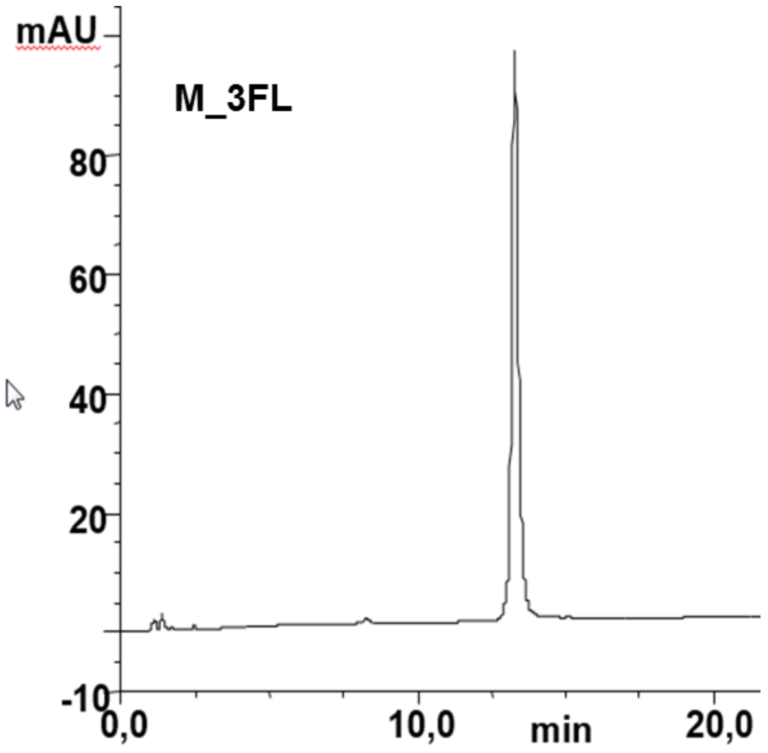

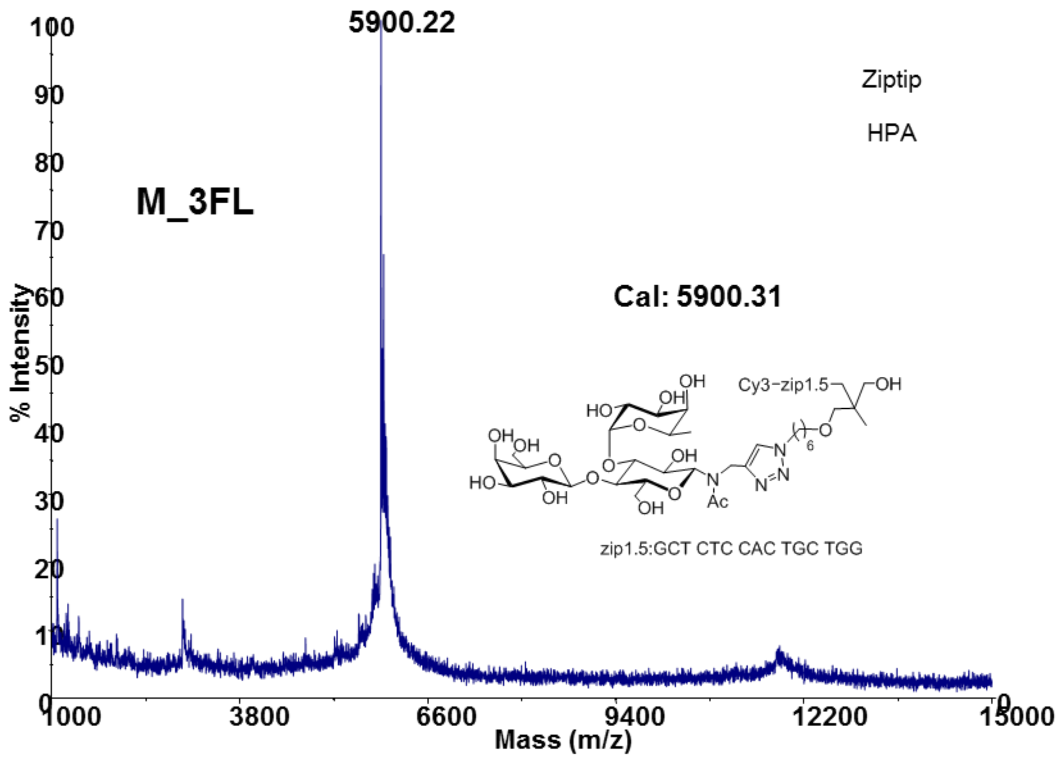

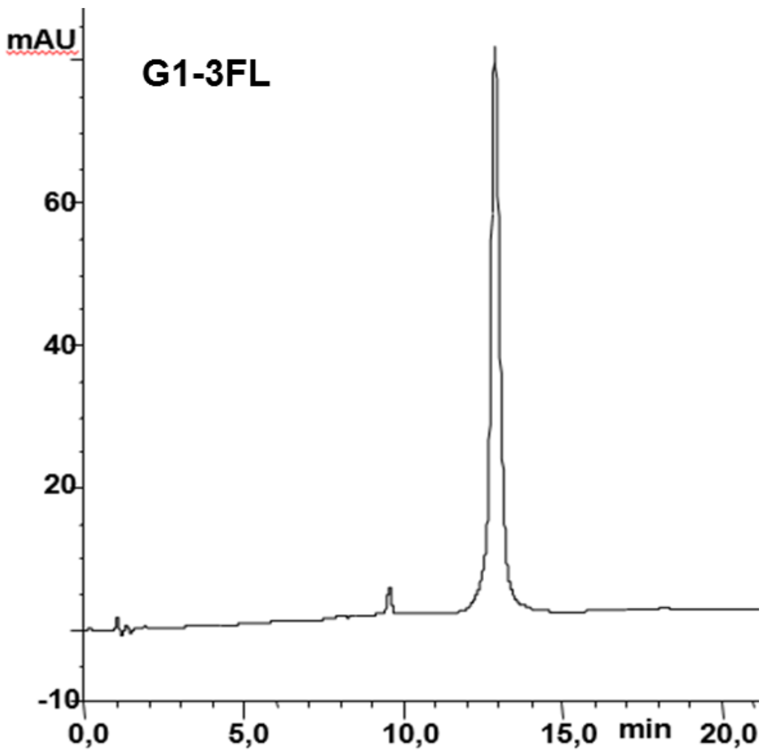
mAU
G1-3FL
60
40
20
-10
0,0
5,0
10,0
15,0
min
20,0

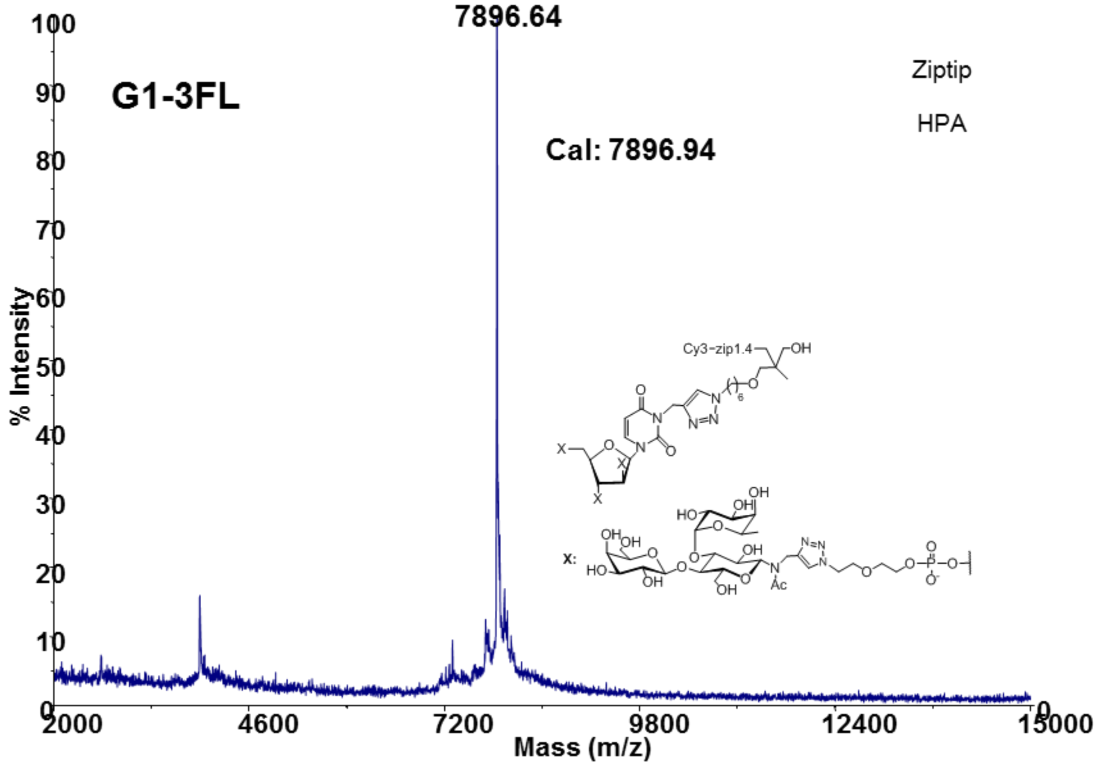
7896.64
100
90
80
70
60
50
40
30
20
10
0
G1-3FL
Ziptip
HPA
Cal: 7896.94
% Intensity
Cy3-zip1.4
OH
X:
2000
4600
7200
9800
12400
15000
Mass (m/z)

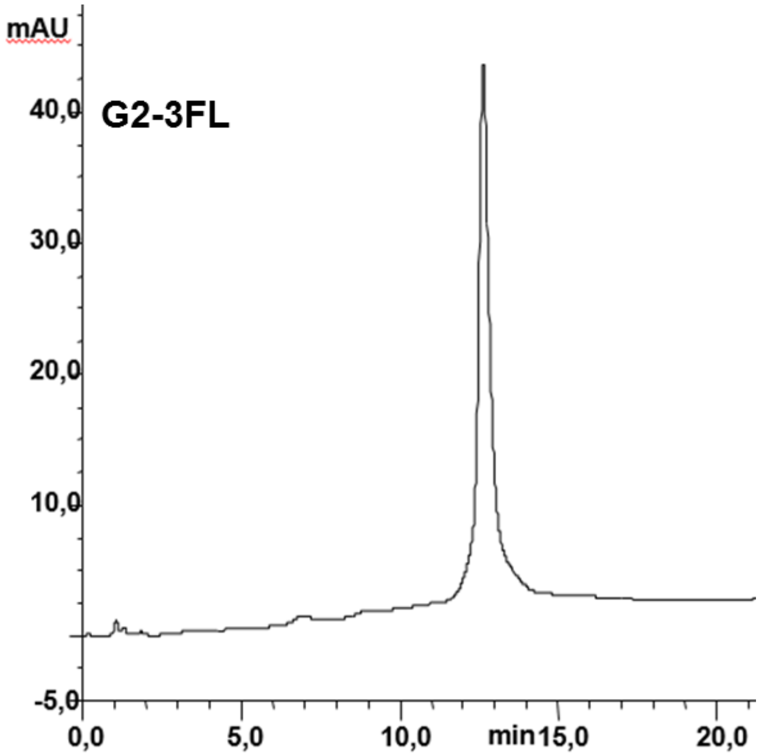
mAU
G2-3FL
40,0
30,0
20,0
10,0
-5,0
0,0
5,0
10,0
min
15,0
20,0

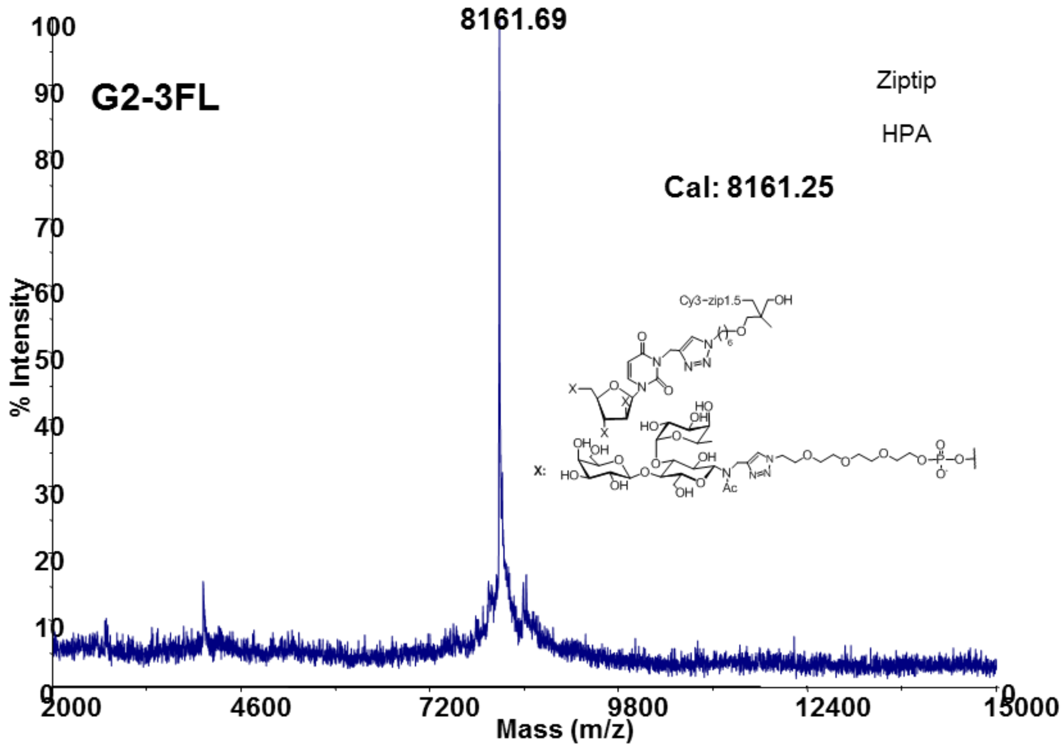
8161.69
G2-3FL
Ziptip
HPA
Cal: 8161.25
% Intensity
Mass (m/z)
Cy3-zip1.5
OH
X:
2000
4600
7200
9800
12400
15000

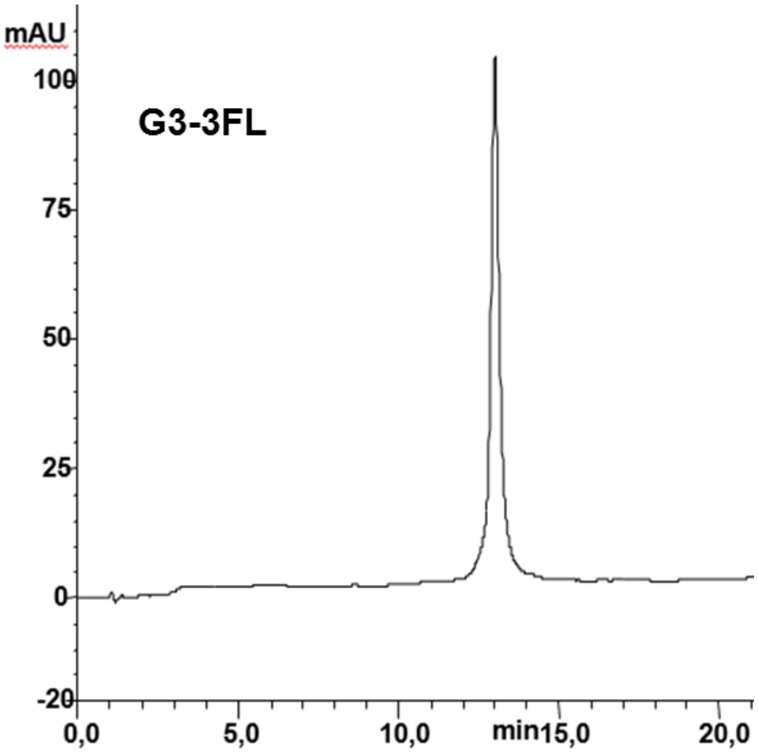
mAU
G3-3FL
100
75
50
25
0
-20
0,0
5,0
10,0
min15,0
20,0

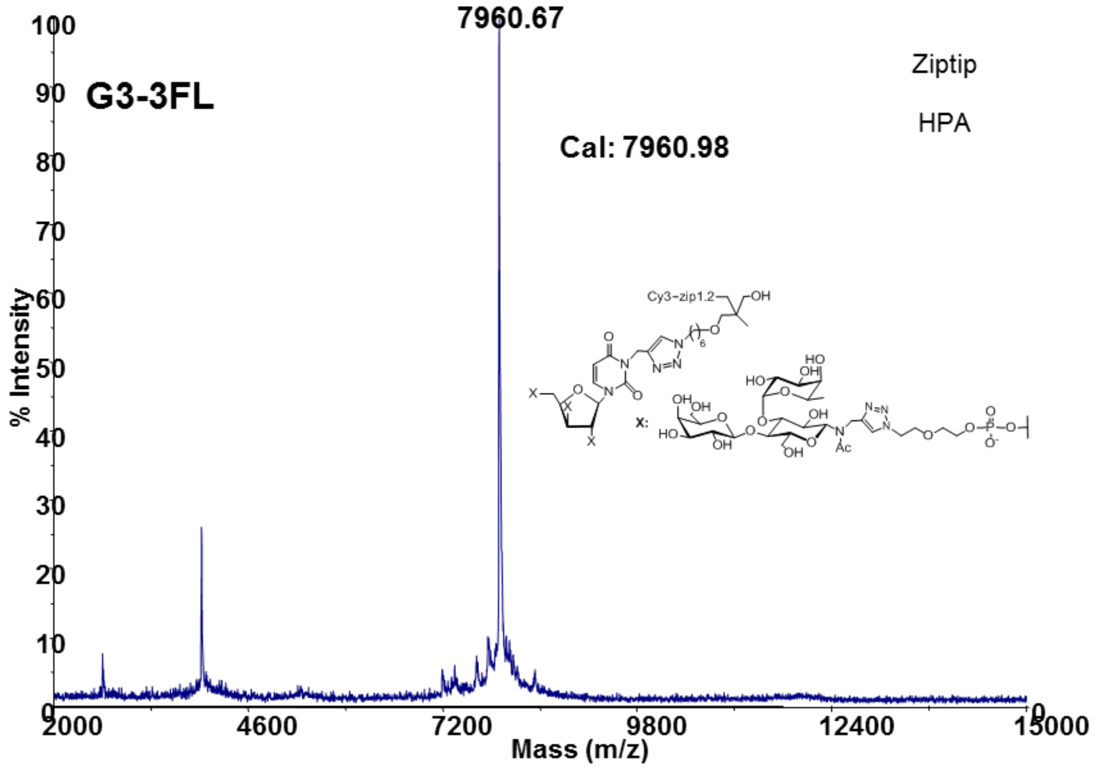
7960.67
G3-3FL
Ziptip
HPA
Cal: 7960.98
% Intensity
Mass (m/z)
2000
4600
7200
9800
12400
15000
Cy3-zip1.2
X:

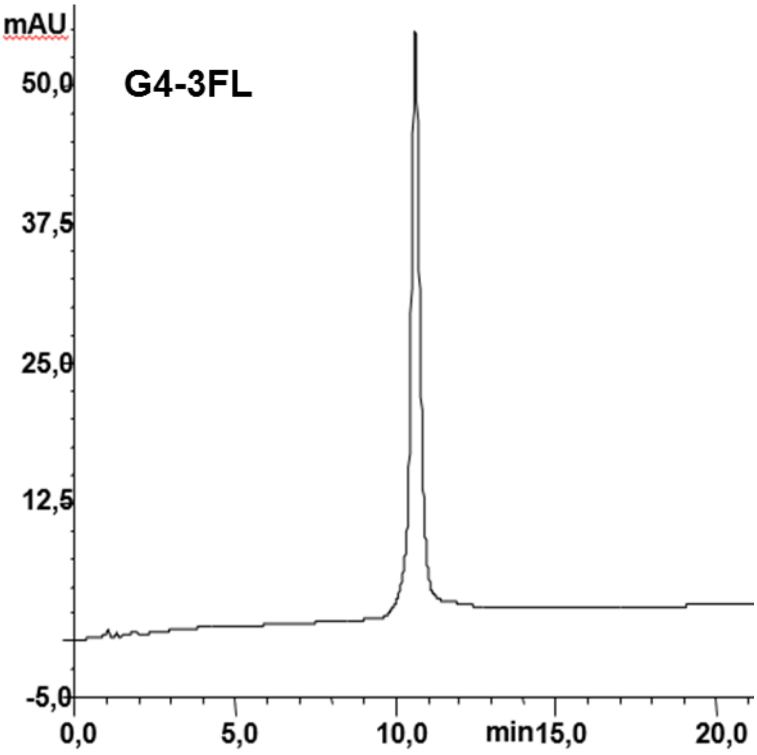
mAU
G4-3FL
50,0
37,5
25,0
12,5
-5,0
0,0
5,0
10,0
min
15,0
20,0

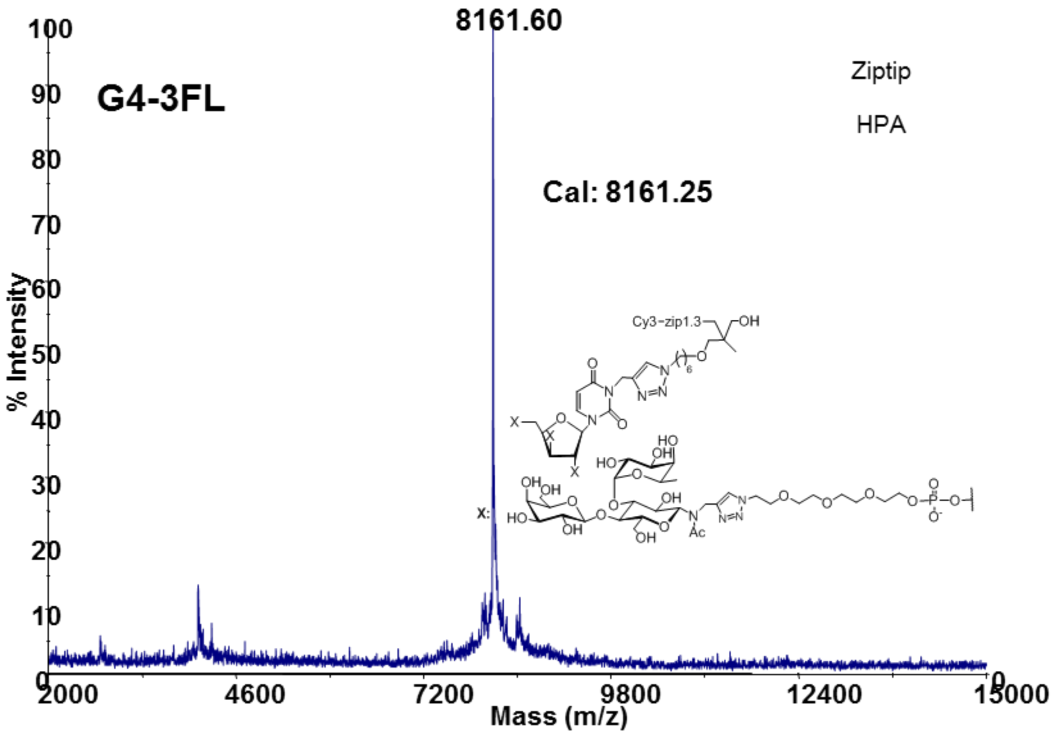
8161.60
G4-3FL
Ziptip
HPA
Cal: 8161.25
% Intensity
Mass (m/z)
100
90
80
70
60
50
40
30
20
10
0
2000
4600
7200
9800
12400
15000
Cy3-zip1.3
OH
X:

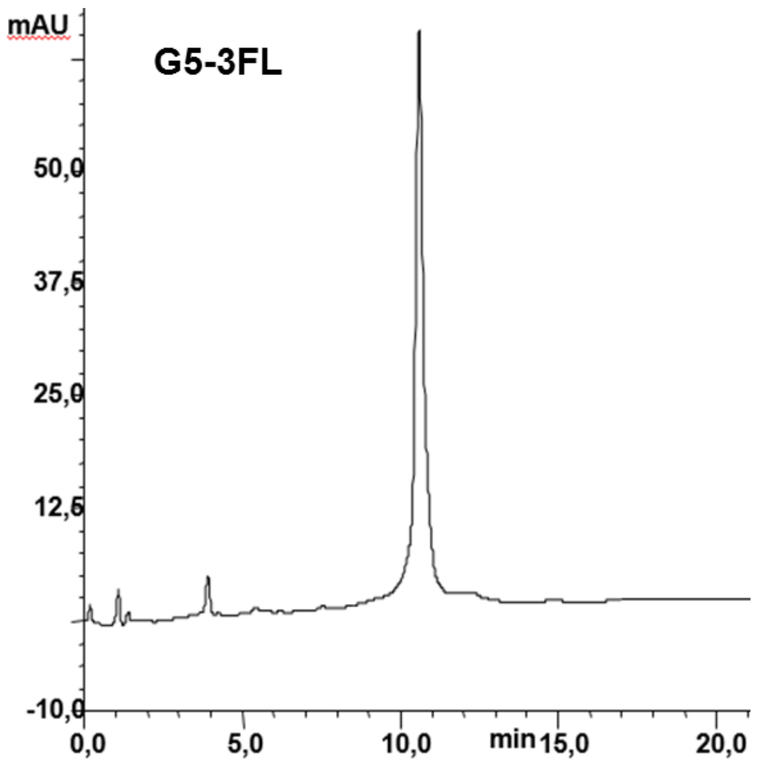
mAU
G5-3FL
50,0
37,5
25,0
12,5
-10,0
0,0
5,0
10,0
min
15,0
20,0

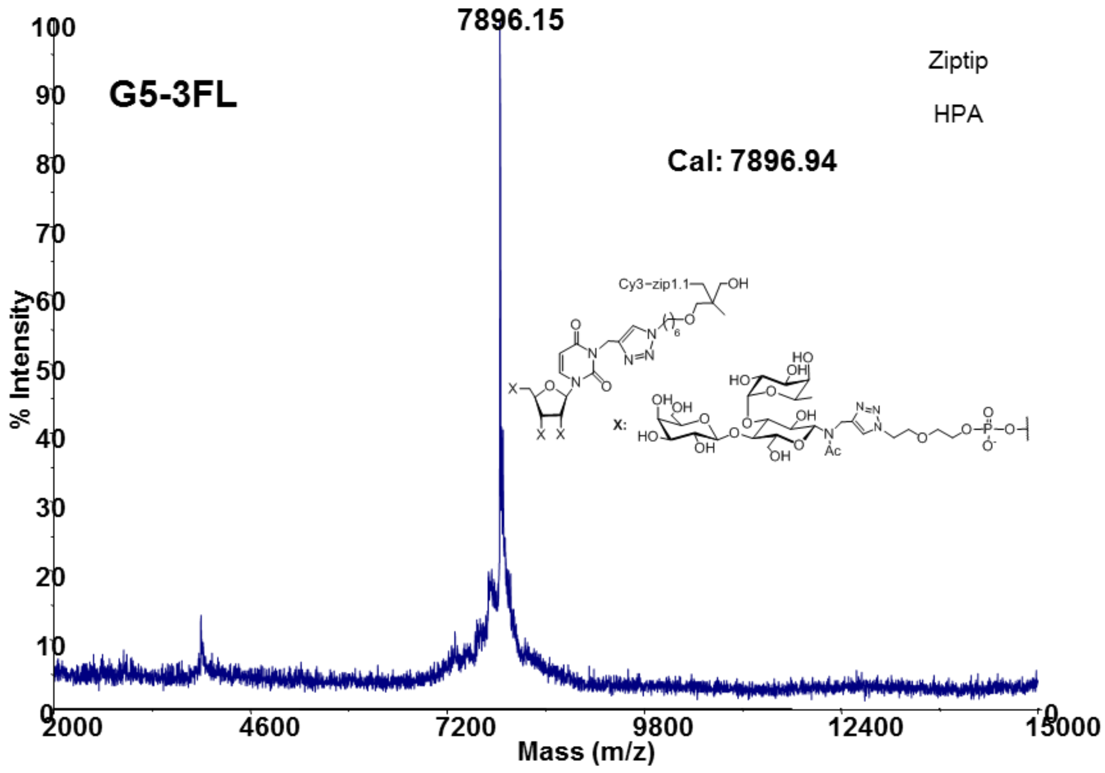
7896.15
G5-3FL
Ziptip
HPA
Cal: 7896.94
% Intensity
Mass (m/z)
Cy3-zip1.1
X:
2000
4600
7200
9800
12400
15000

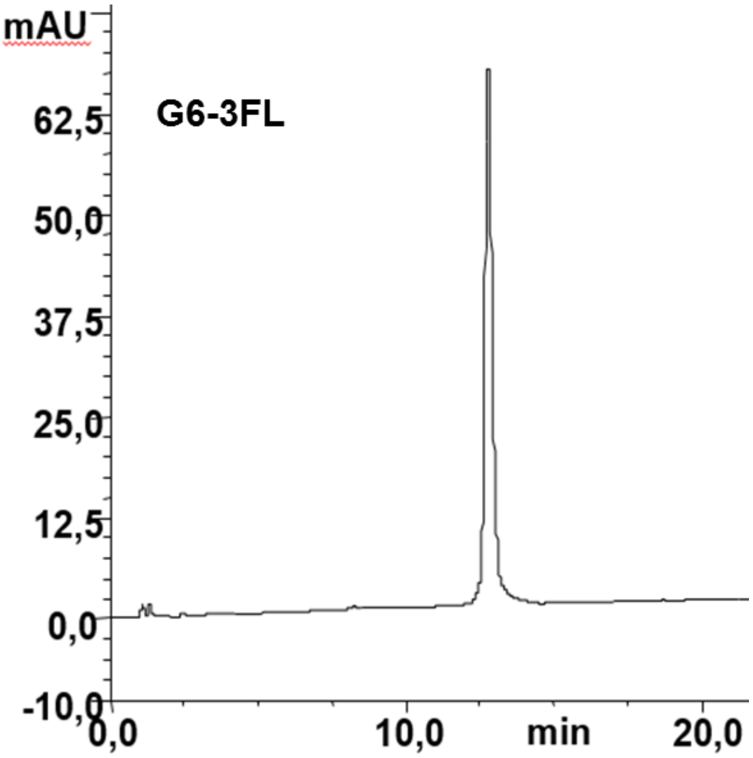
mAU
G6-3FL
62,5
50,0
37,5
25,0
12,5
0,0
-10,0
0,0
10,0
min
20,0

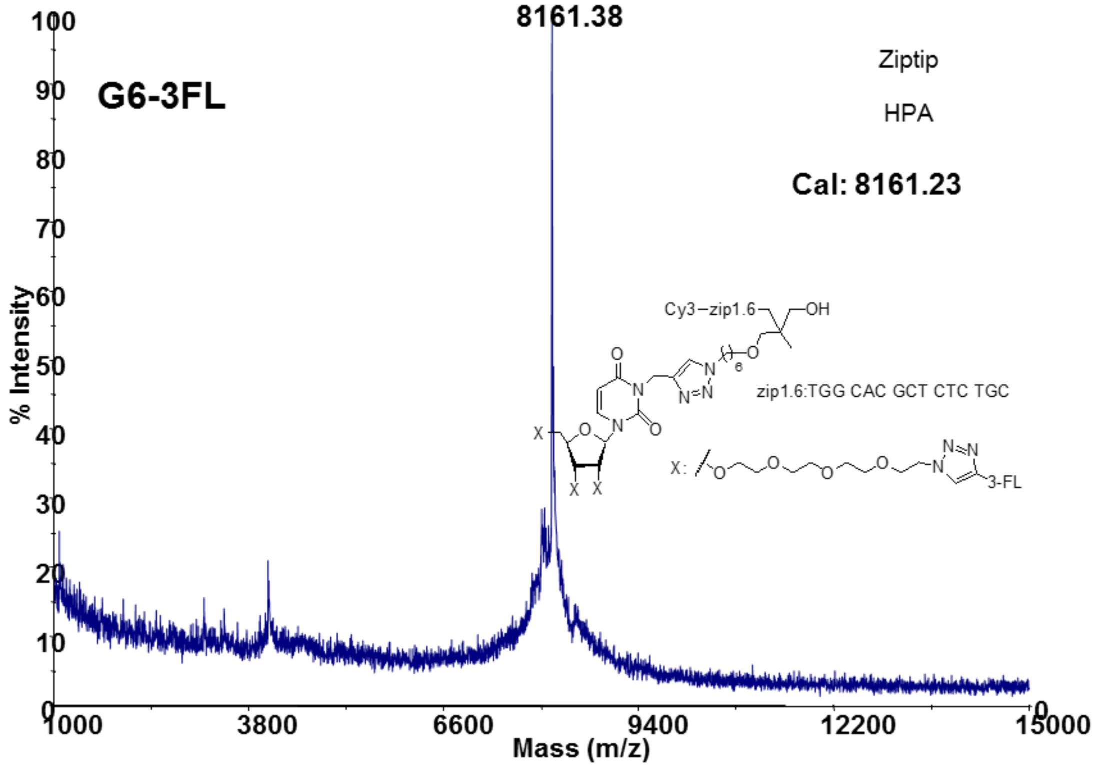
8161.38
G6-3FL
Ziptip
HPA
Cal: 8161.23
Cy3−zip1.6
OH
zip1.6:TGG CAC GCT CTC TGC
X:
3-FL
% Intensity
Mass (m/z)
1000
3800
6600
9400
12200
15000

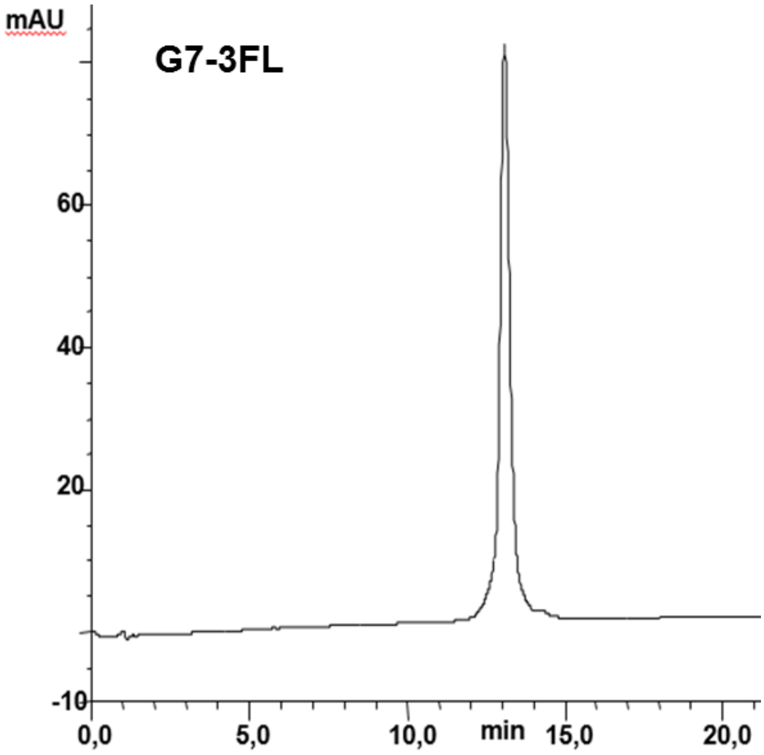

mAU
G7-3FL
60
40
20
-10
0,0
5,0
10,0
min
15,0
20,0

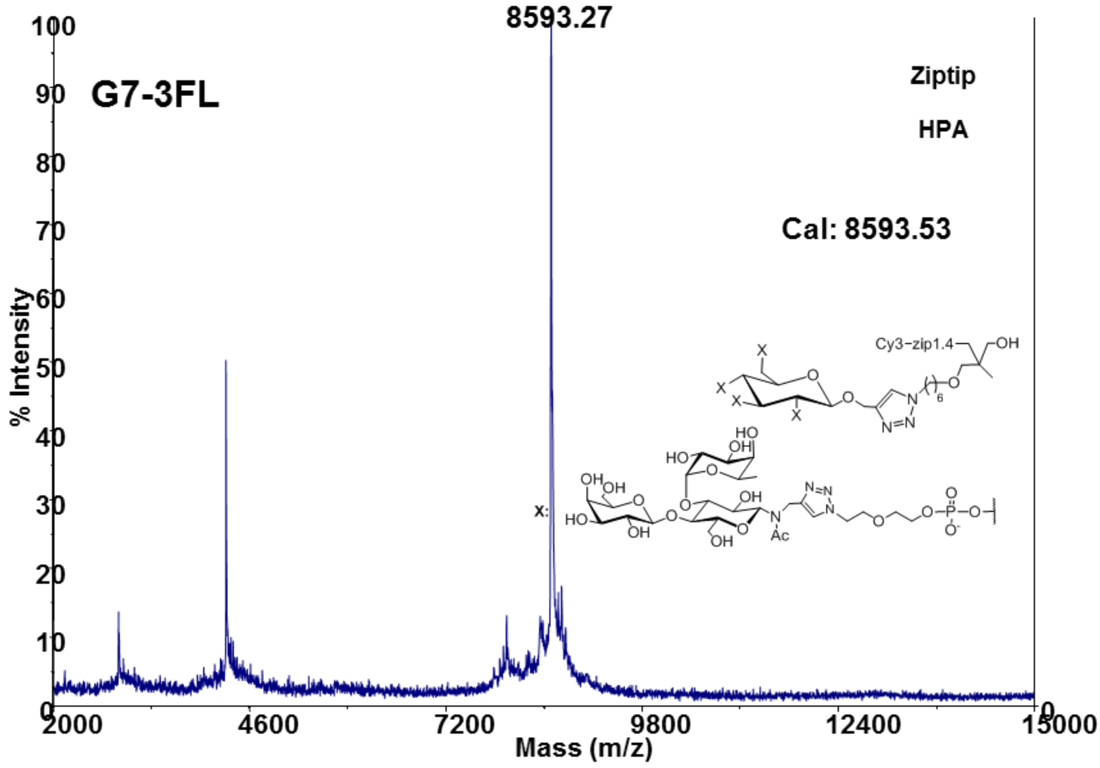

8593.27
G7-3FL
Ziptip
HPA
Cal: 8593.53
% Intensity
Mass (m/z)
100
90
80
70
60
50
40
30
20
10
0
2000
4600
7200
9800
12400
15000
Cy3–zip1.4
OH
X
O
N
N=N
6
HO
OH
Ac
O
P
X:

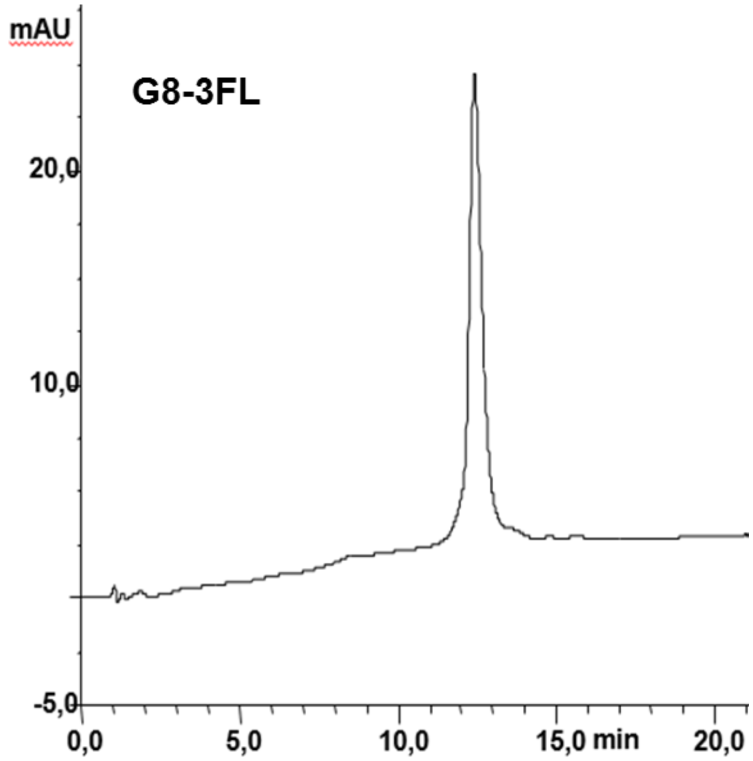
mAU
G8-3FL
20,0
10,0
-5,0
0,0
5,0
10,0
15,0 min
20,0

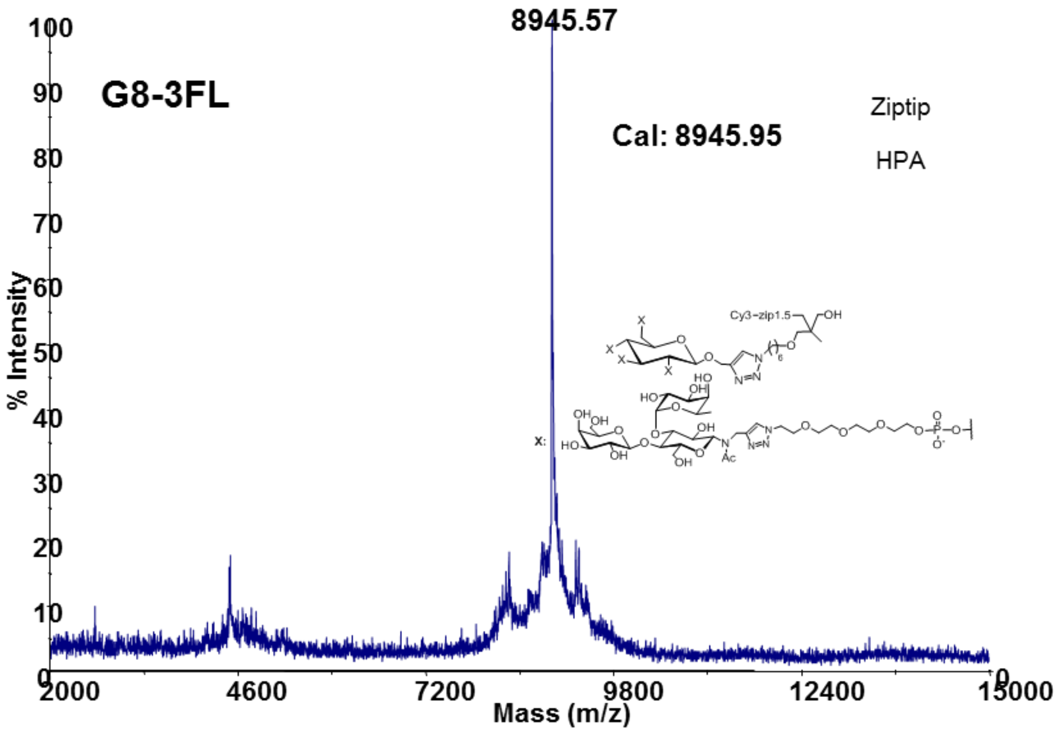
8945.57
G8-3FL
Cal: 8945.95
Ziptip
HPA
Cy3-zip1.5
% Intensity
Mass (m/z)
2000
4600
7200
9800
12400
15000

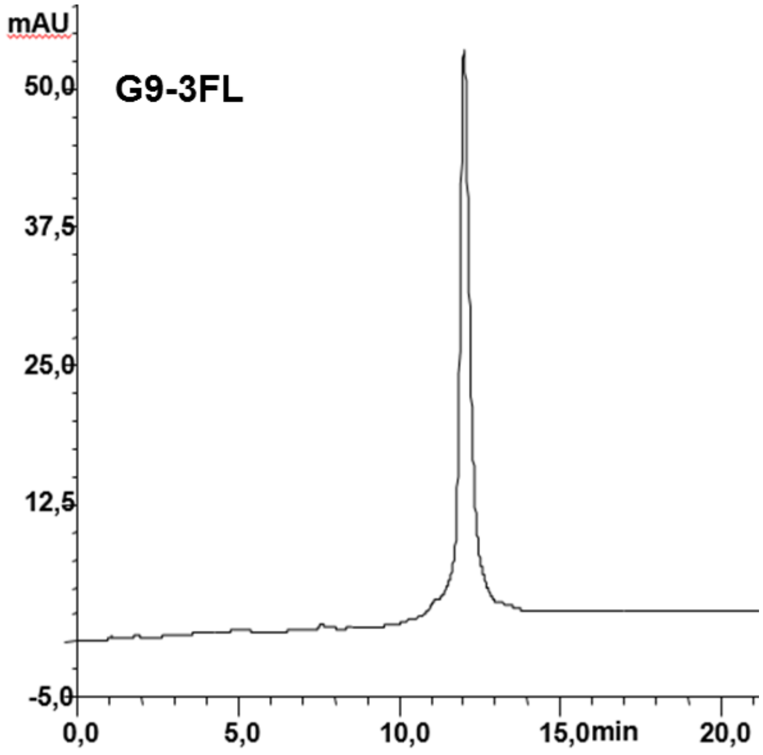
mAU
50,0
37,5
25,0
12,5
-5,0
G9-3FL
0,0
5,0
10,0
15,0min
20,0

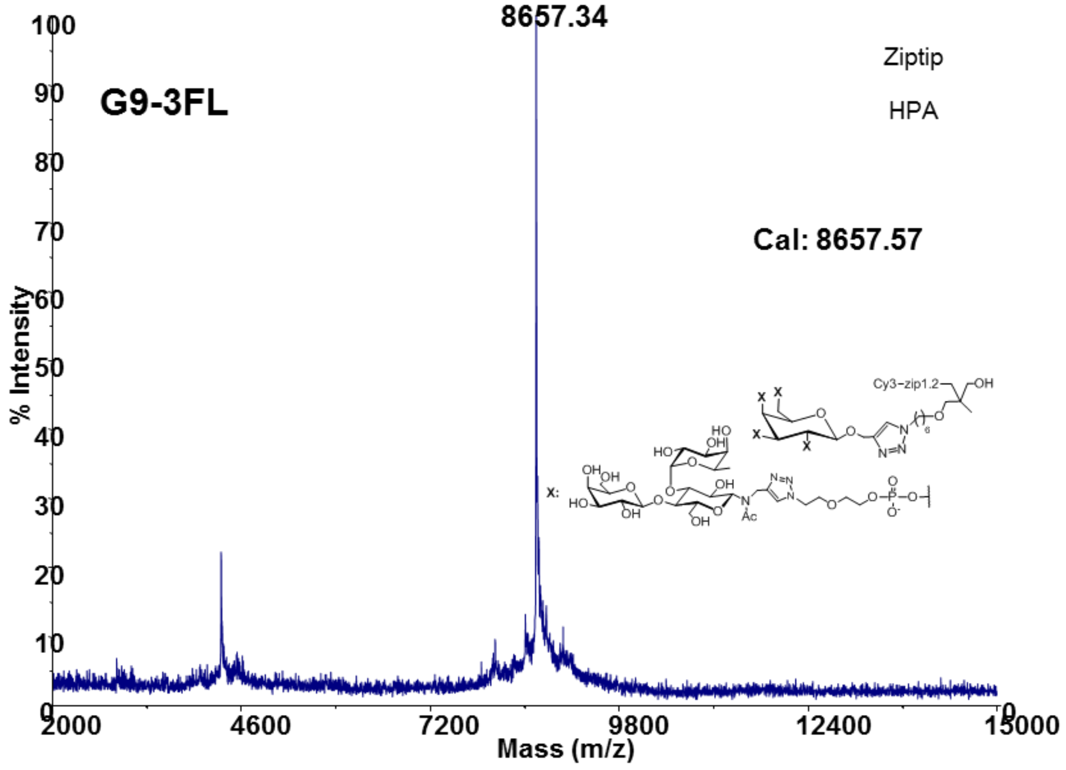
8657.34
G9-3FL
Ziptip
HPA
Cal: 8657.57
% Intensity
100
90
80
70
60
50
40
30
20
10
0
2000
4600
7200
9800
12400
15000
Mass (m/z)
Cy3-zip1.2
X:

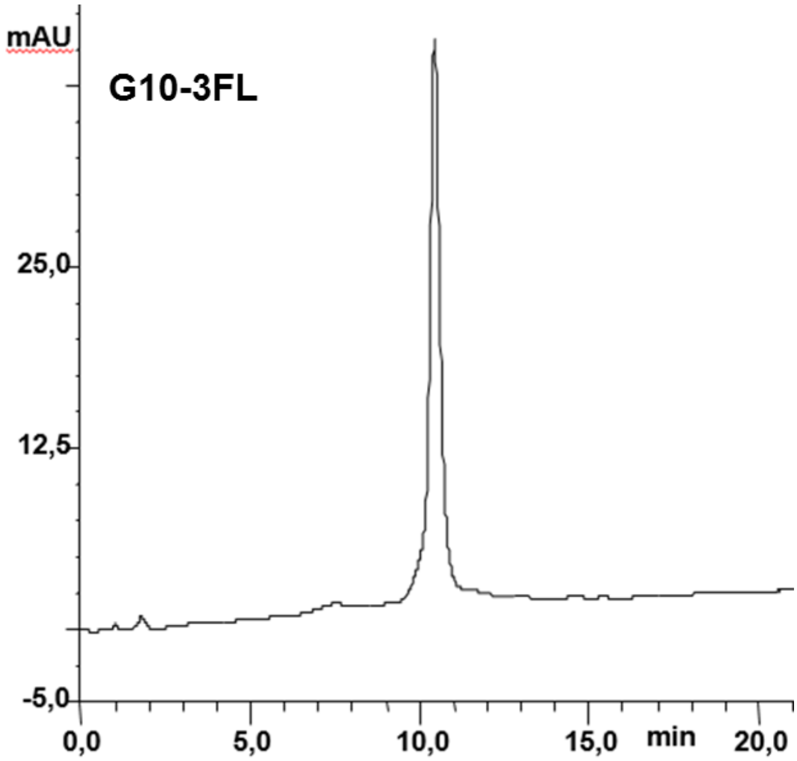
mAU
G10-3FL
25,0
12,5
-5,0
0,0
5,0
10,0
15,0
min
20,0

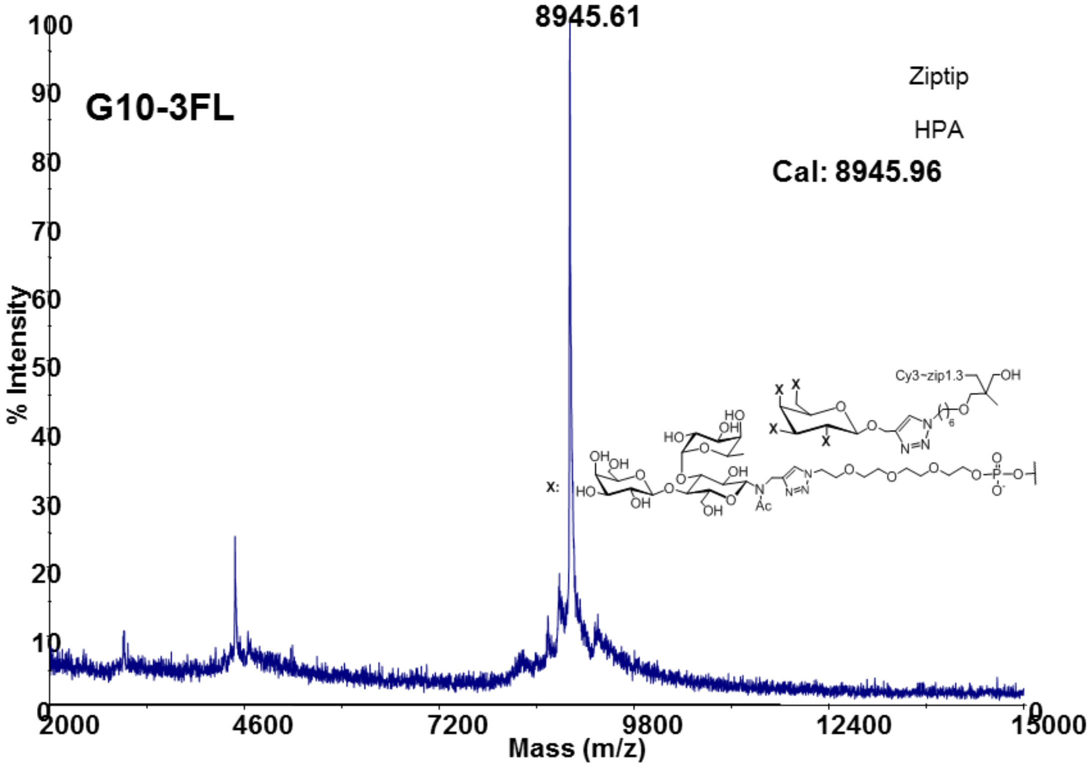
8945.61
G10-3FL
Ziptip
HPA
Cal: 8945.96
% Intensity
Mass (m/z)
2000
4600
7200
9800
12400
15000
Cy3-zip1.3
x:

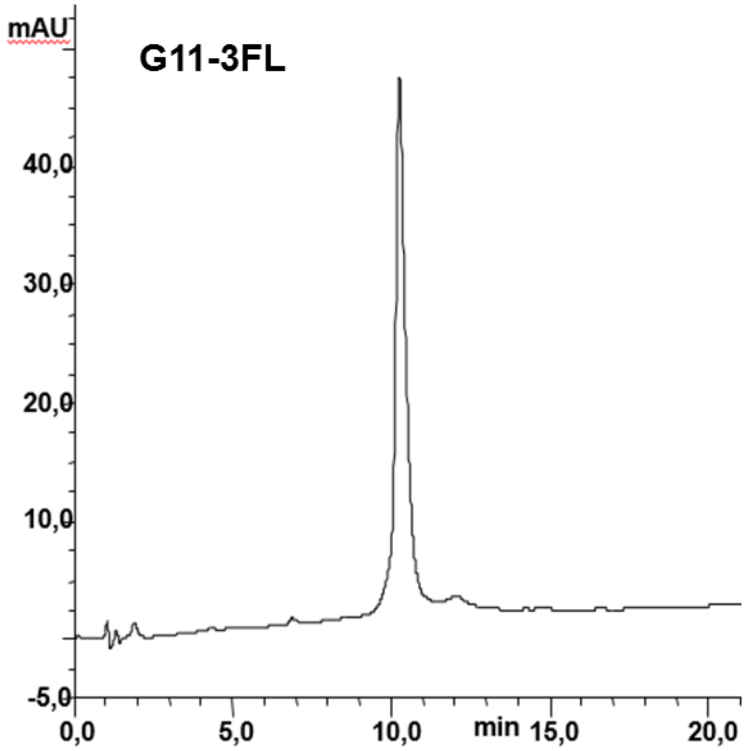
mAU
G11-3FL
40,0
30,0
20,0
10,0
-5,0
0,0
5,0
10,0
min
15,0
20,0

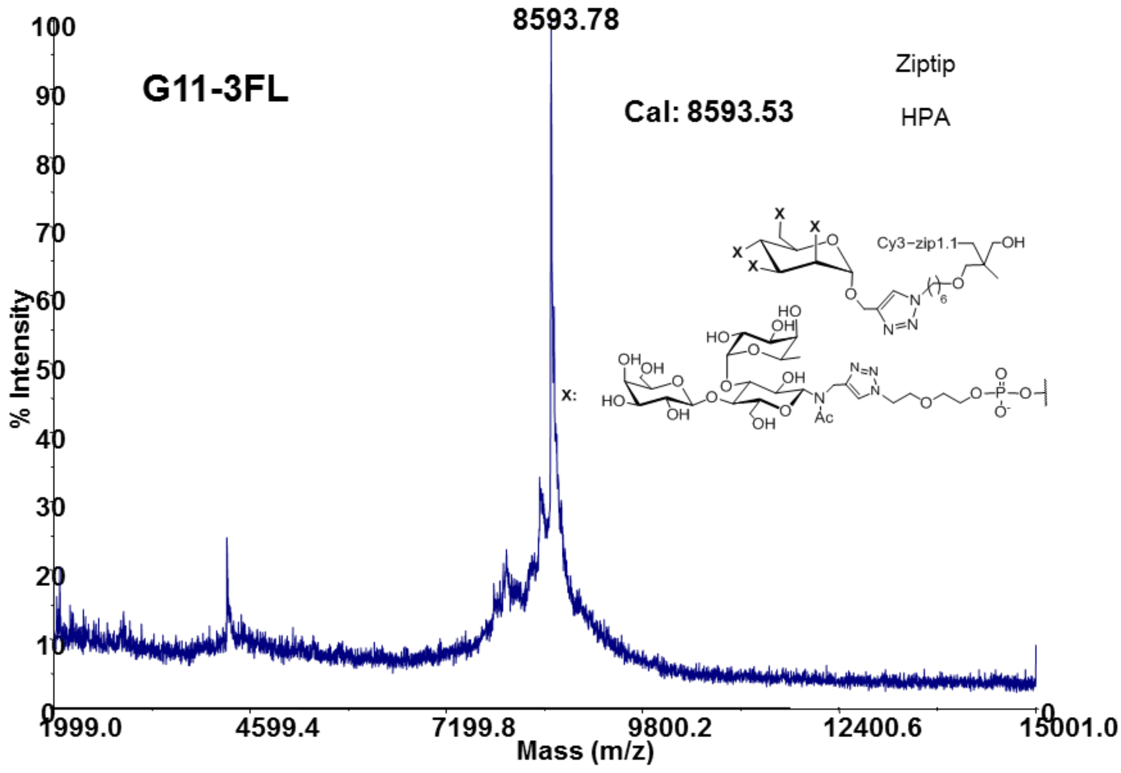
8593.78
G11-3FL
Cal: 8593.53
Ziptip
HPA
% Intensity
100
90
80
70
60
50
40
30
20
10
0
1999.0
4599.4
7199.8
9800.2
12400.6
15001.0
Mass (m/z)
Cy3-zip1.1
X:

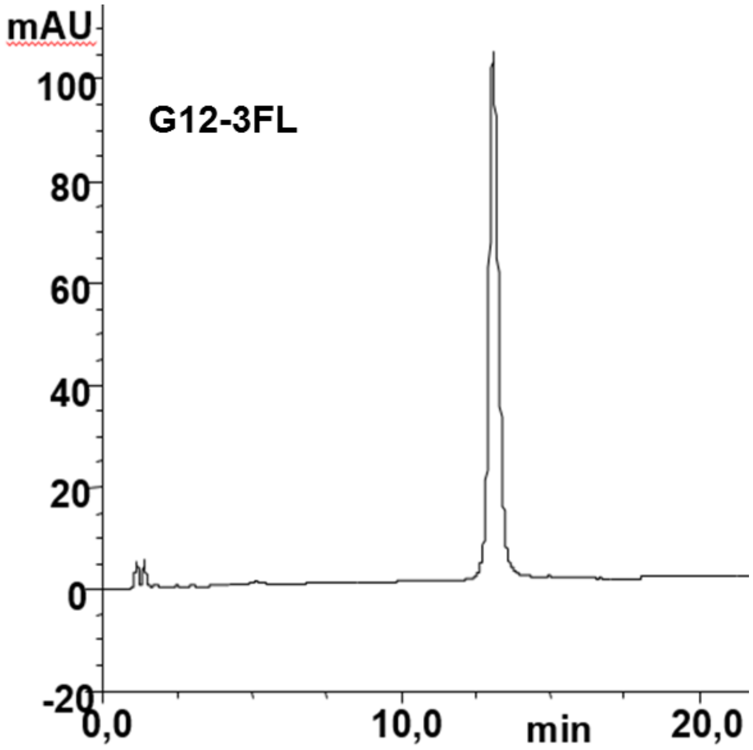
mAU
100
80
60
40
20
0
-20
G12-3FL
0,0
10,0
min
20,0

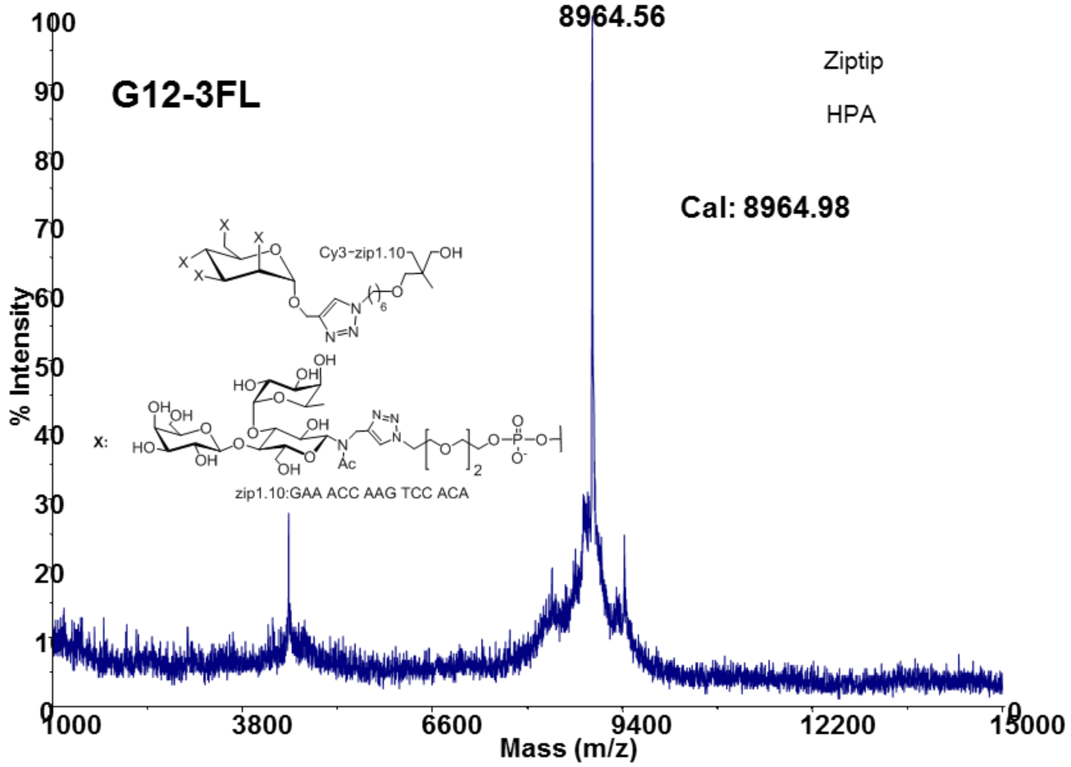
8964.56
Ziptip
HPA
G12-3FL
Cal: 8964.98
Cy3-zip1.10
zip1.10:GAA ACC AAG TCC ACA
% Intensity
Mass (m/z)
1000
3800
6600
9400
12200
15000

S7: HPLC chromatograms and MALDI-ToF spectra of oligoglycoclusters with Lewis[a]

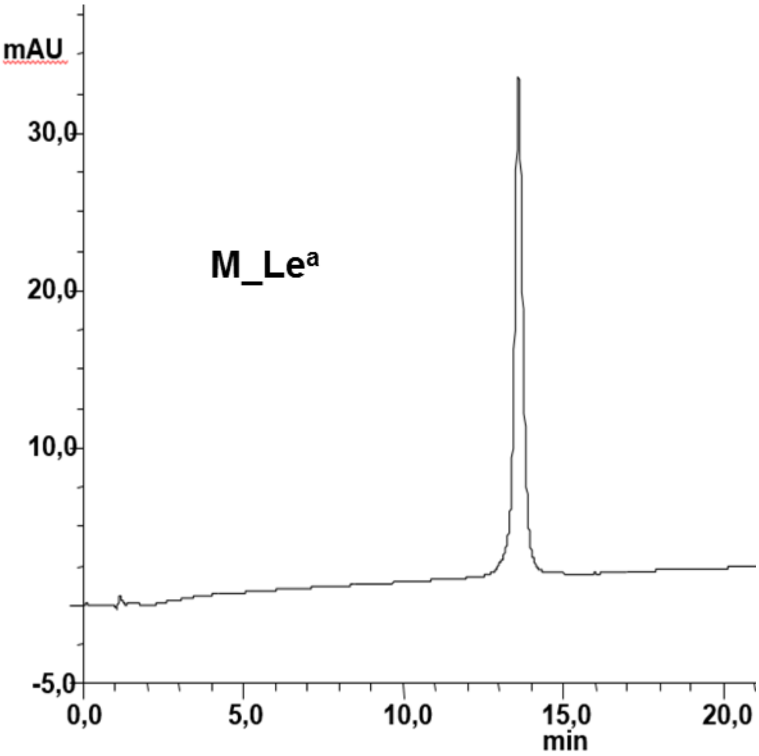

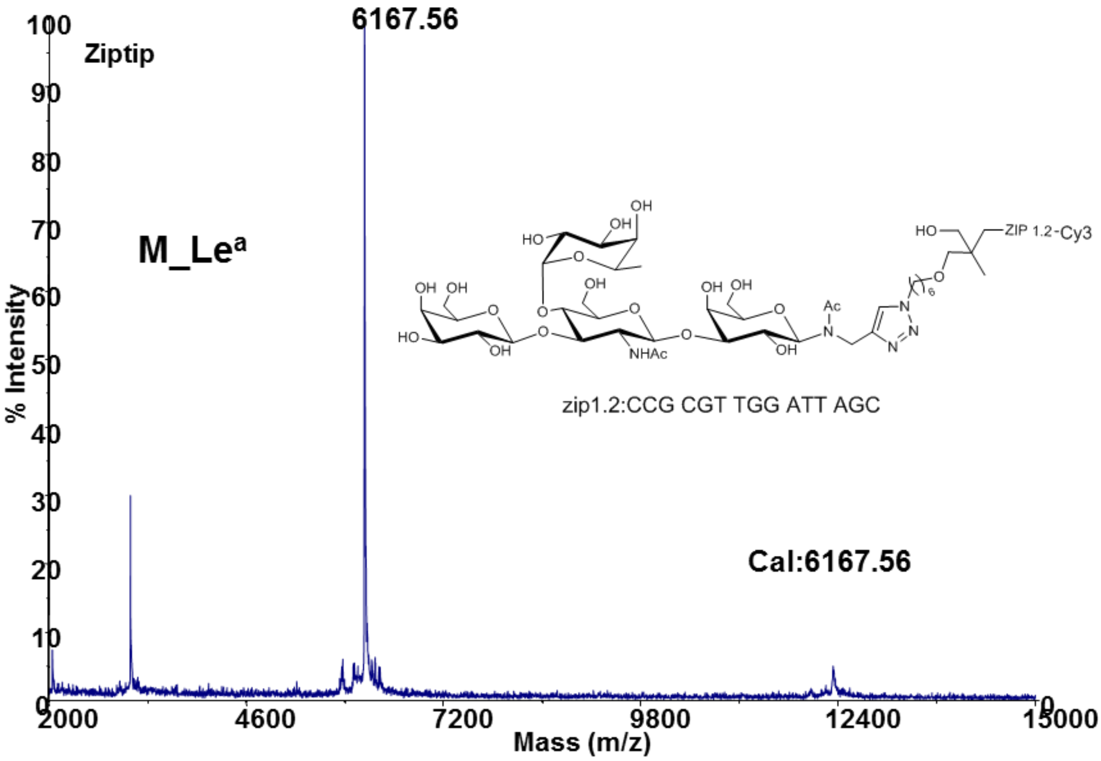

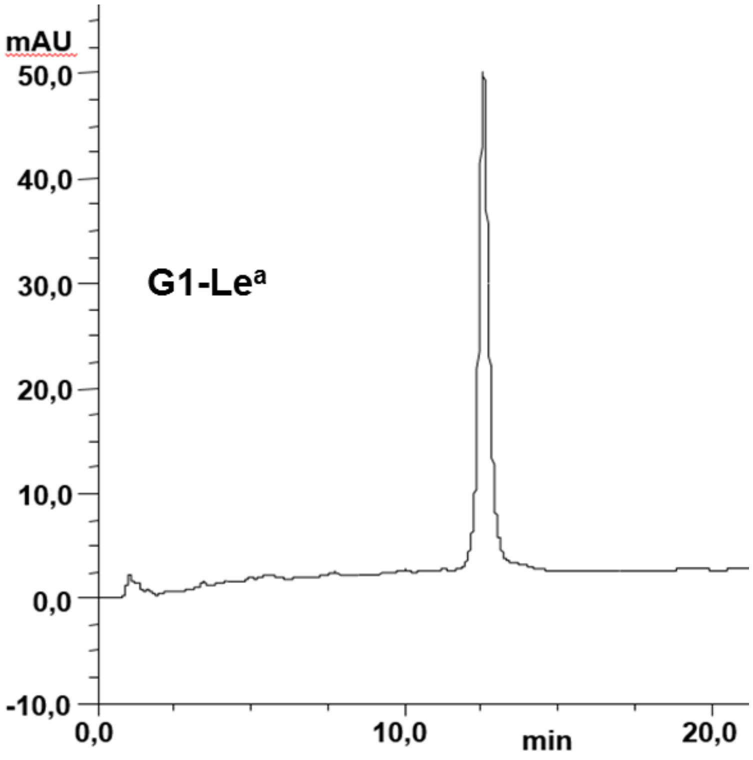
mAU
50,0
40,0
30,0
20,0
10,0
0,0
-10,0
G1-Le^a
0,0
10,0
min
20,0

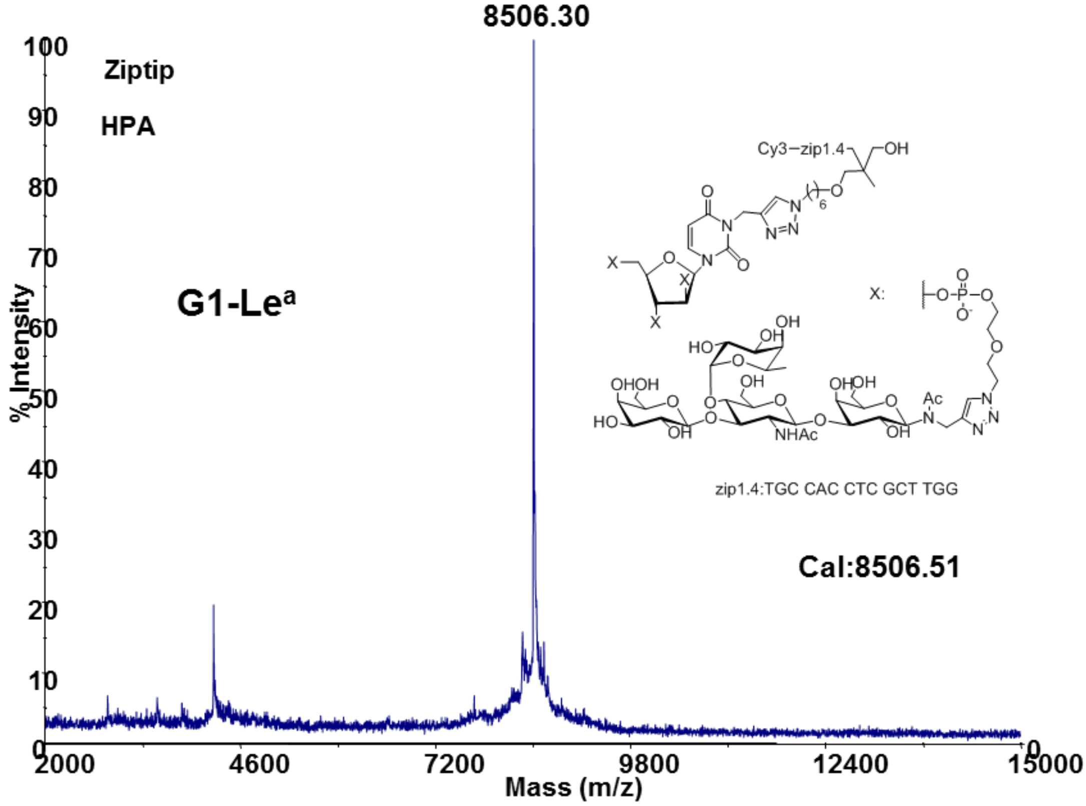
8506.30
Ziptip
HPA
G1-Le^a
% Intensity
100
90
80
70
60
50
40
30
20
10
0
Cy3−zip1.4
OH
6
X
X:
NHAc
Ac
zip1.4:TGC CAC CTC GCT TGG
Cal:8506.51
2000
4600
7200
9800
12400
15000
Mass (m/z)

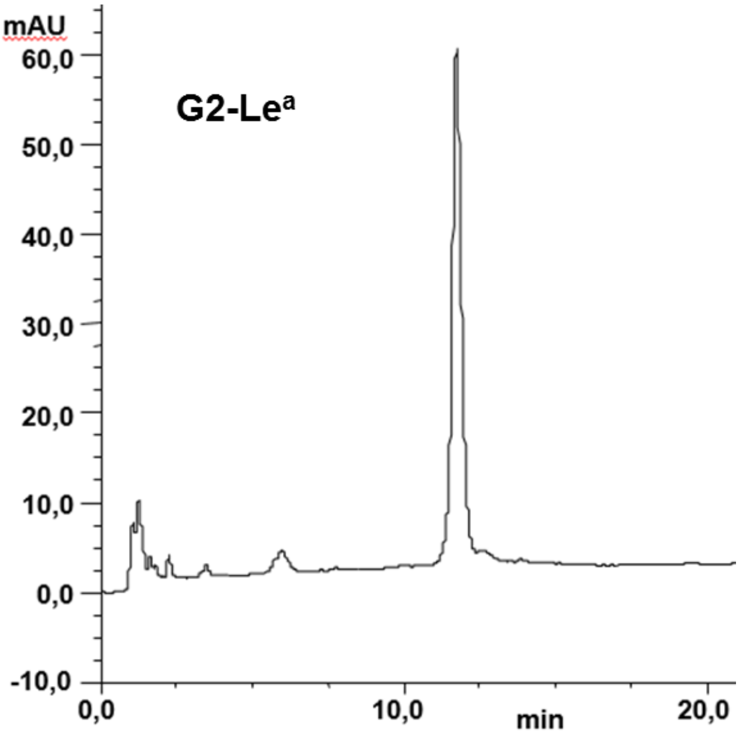
mAU
G2-Le^a
60,0
50,0
40,0
30,0
20,0
10,0
0,0
-10,0
0,0
10,0
min
20,0

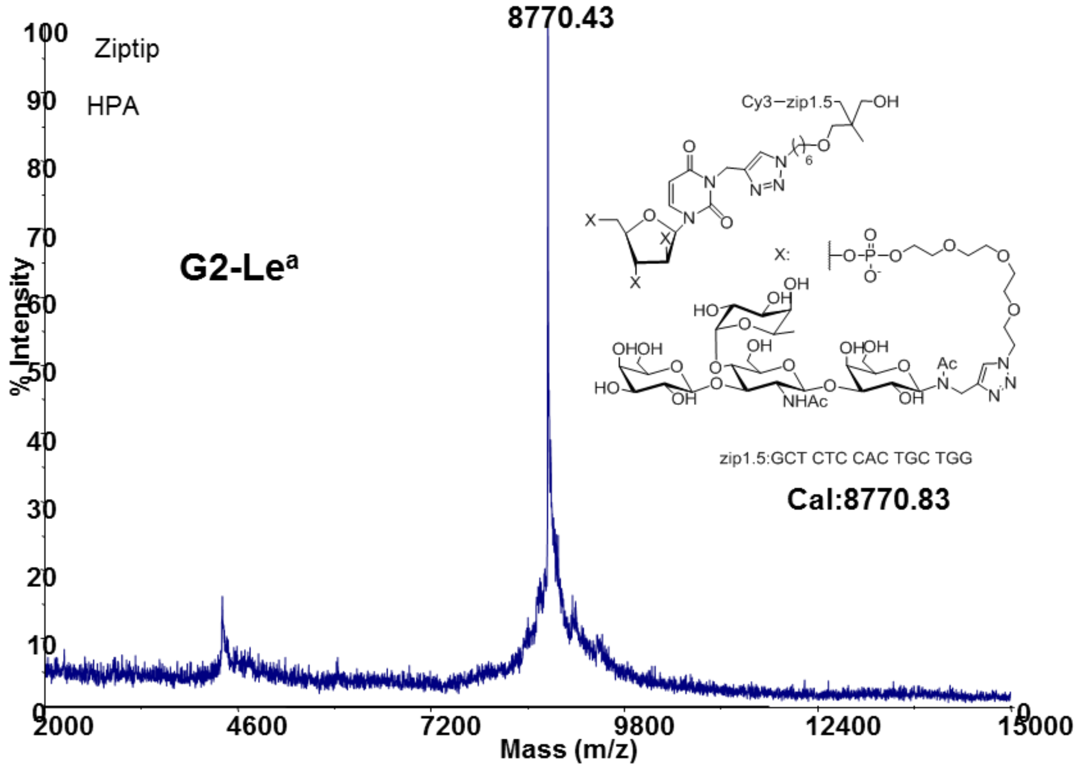
8770.43
Ziptip
HPA
G2-Le^a
% Intensity
100
90
80
70
60
50
40
30
20
10
0
2000
4600
7200
9800
12400
15000
Mass (m/z)
Cy3—zip1.5
OH
X:
NHAc
zip1.5:GCT CTC CAC TGC TGG
Cal:8770.83

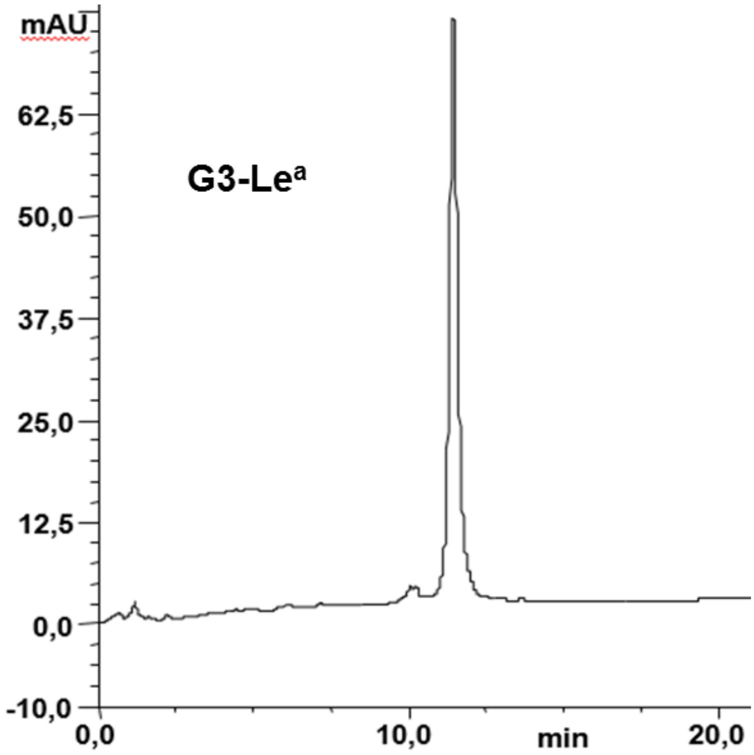
mAU
62,5
50,0
37,5
25,0
12,5
0,0
-10,0
0,0
10,0
min
20,0
G3-Le^a

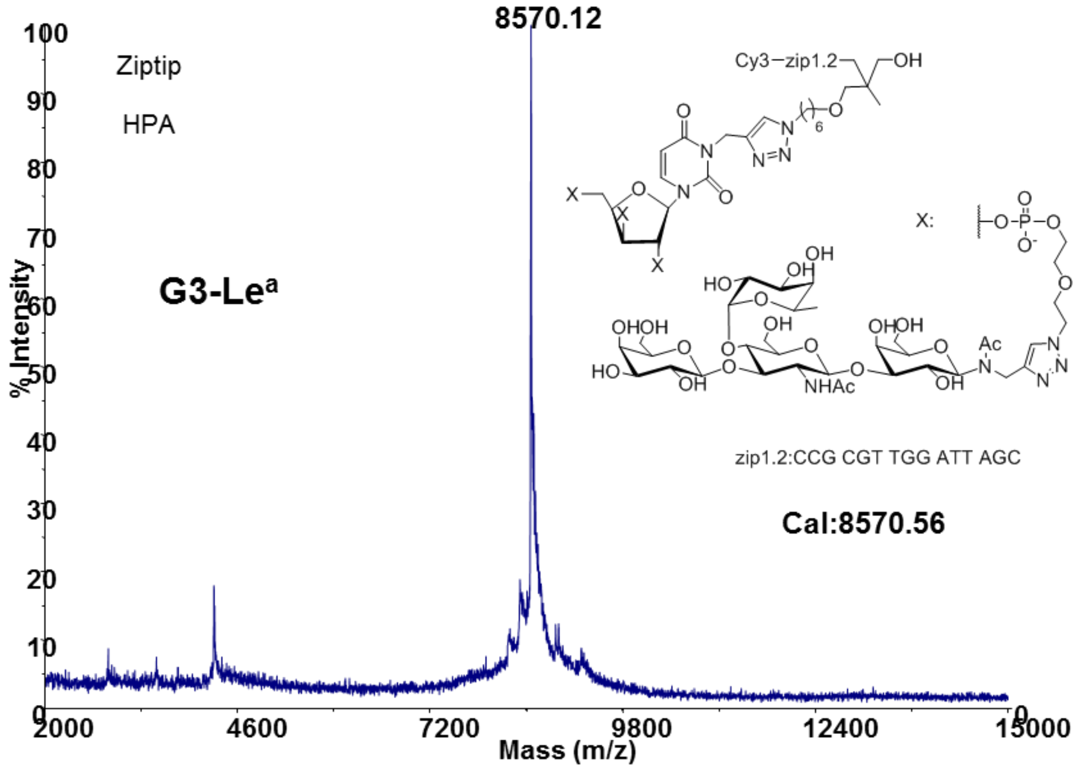
8570.12
Ziptip
HPA
G3-Le^a
% Intensity
Mass (m/z)
2000
4600
7200
9800
12400
15000
Cy3—zip1.2—
OH
X:
NHAc
zip1.2:CCG CGT TGG ATT AGC
Cal:8570.56

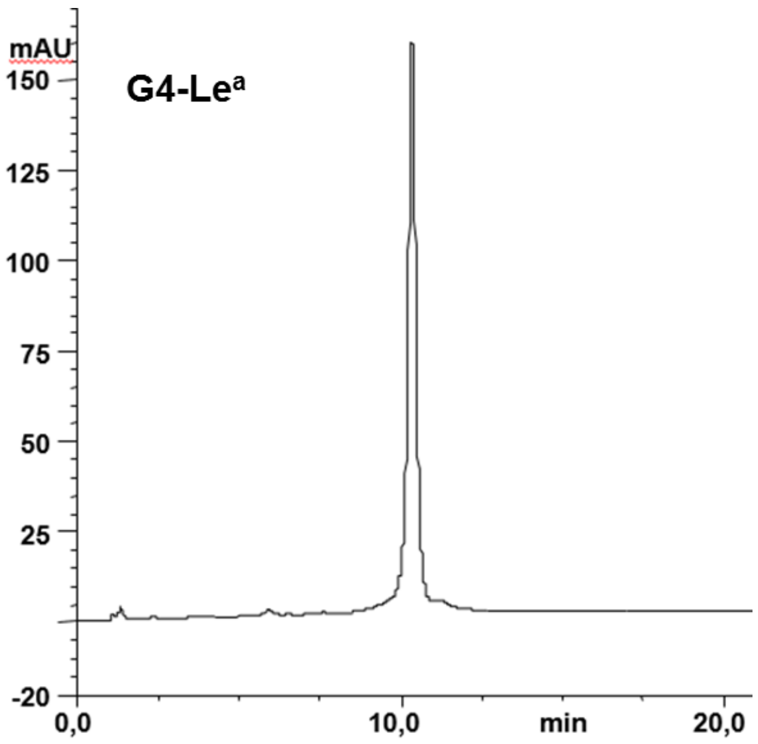
mAU
G4-Le^a
150
125
100
75
50
25
-20
0,0
10,0
min
20,0

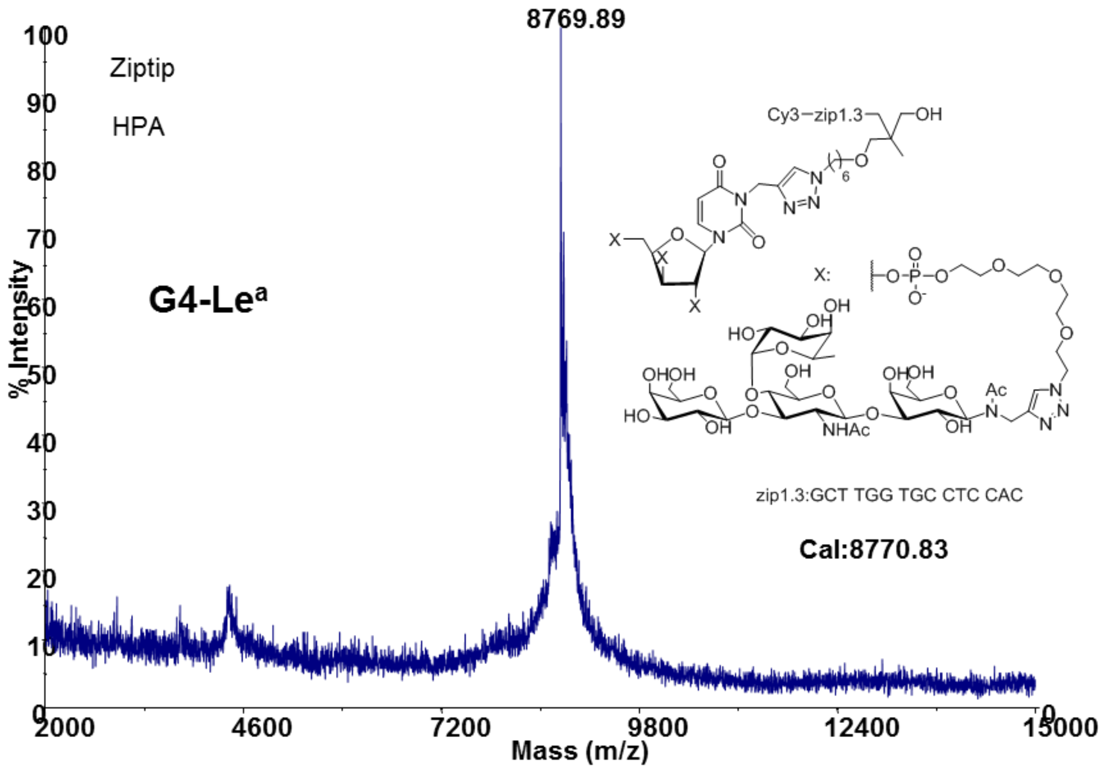
8769.89
Ziptip
HPA
G4-Le^a
% Intensity
Mass (m/z)
Cy3−zip1.3
X:
zip1.3:GCT TGG TGC CTC CAC
Cal:8770.83

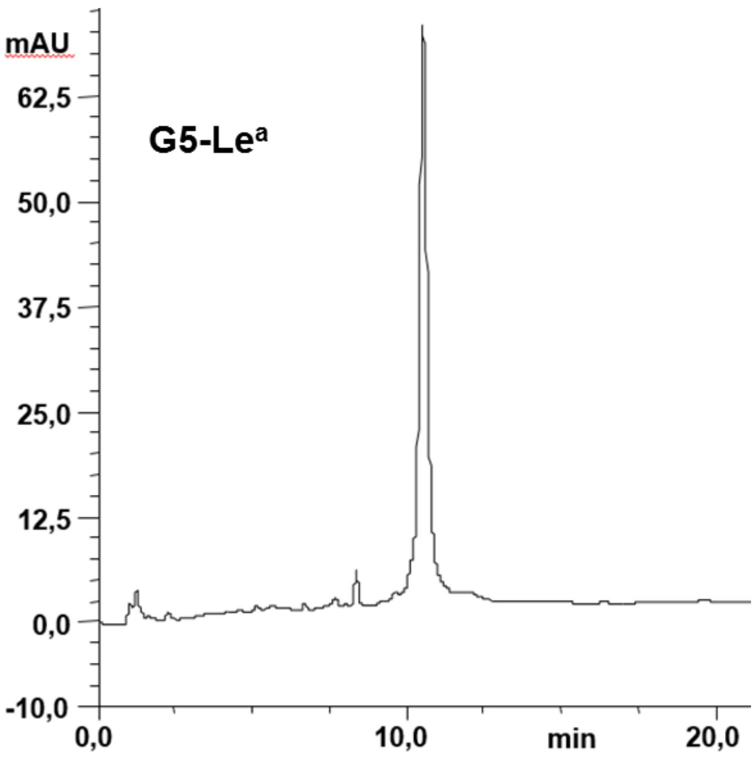
mAU
62,5
50,0
37,5
25,0
12,5
0,0
-10,0
0,0
10,0
min
20,0
G5-Le^a

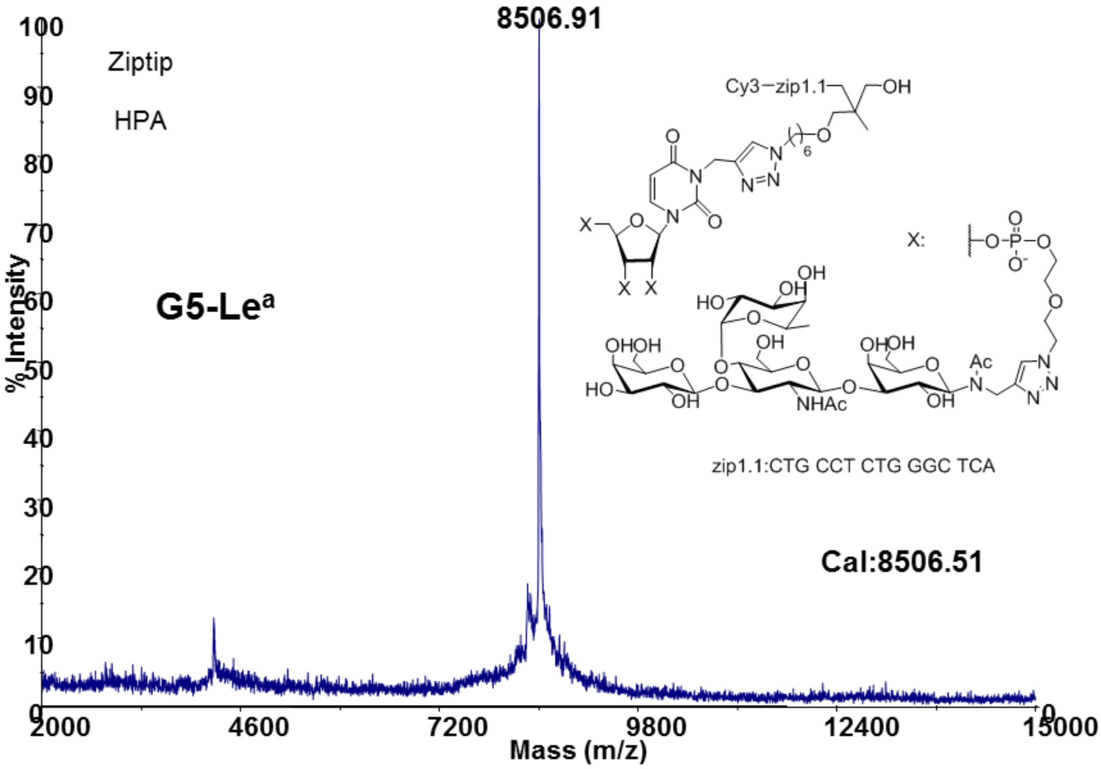
8506.91
Ziptip
HPA
G5-Le^a
% Intensity
100
90
80
70
60
50
40
30
20
10
0
2000
4600
7200
9800
12400
15000
Mass (m/z)
Cy3−zip1.1
OH
X:
zip1.1:CTG CCT CTG GGC TCA
Cal:8506.51

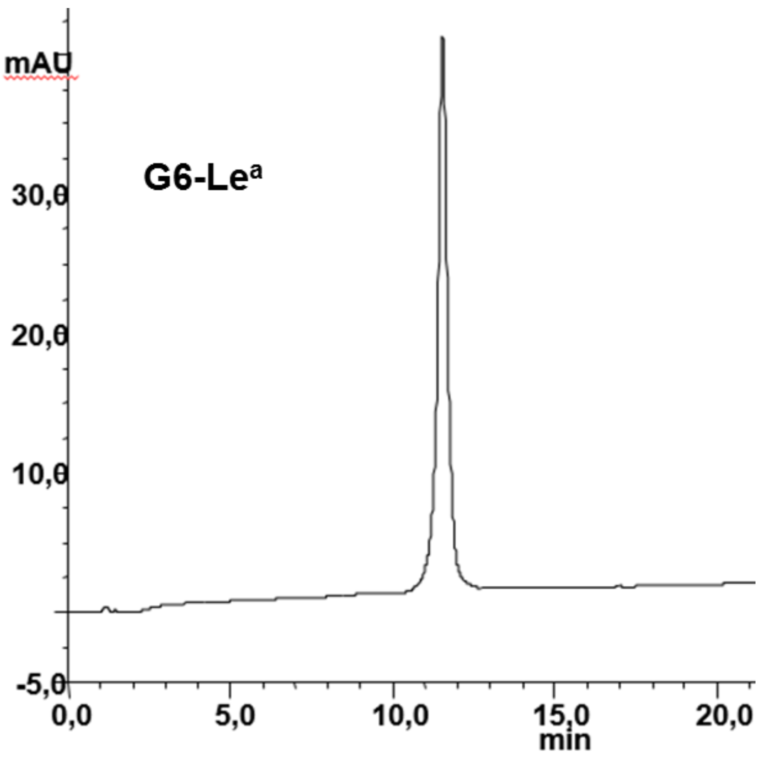

mAU
G6-Le^a
30,0
20,0
10,0
-5,0
0,0
5,0
10,0
15,0
20,0
min

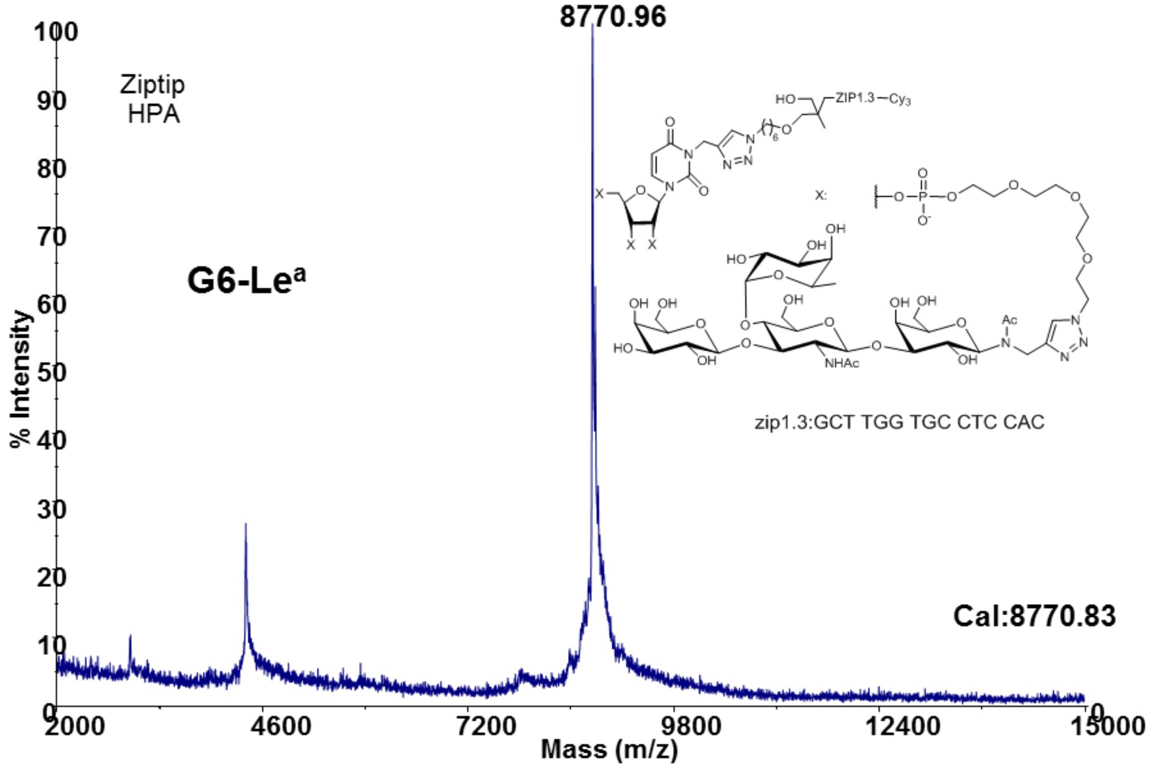

8770.96
Ziptip
HPA
G6-Le^a
% Intensity
100
90
80
70
60
50
40
30
20
10
0
HO
ZIP1.3—Cy3
X:
OH
NHAc
Ac
zip1.3:GCT TGG TGC CTC CAC
Cal:8770.83
2000
4600
7200
9800
12400
15000
Mass (m/z)

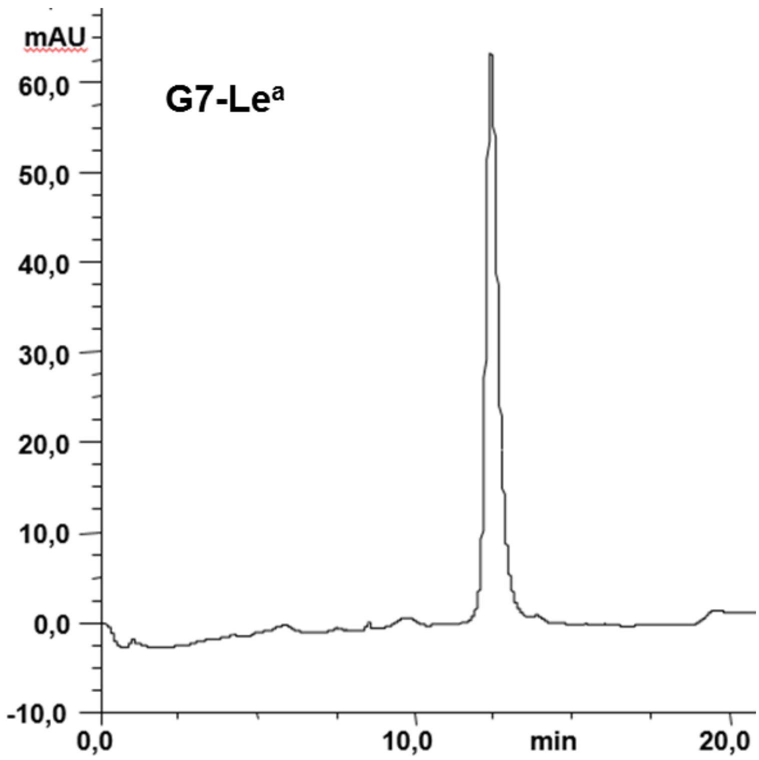
mAU
G7-Le^a
60,0
50,0
40,0
30,0
20,0
10,0
0,0
-10,0
0,0
10,0
min
20,0

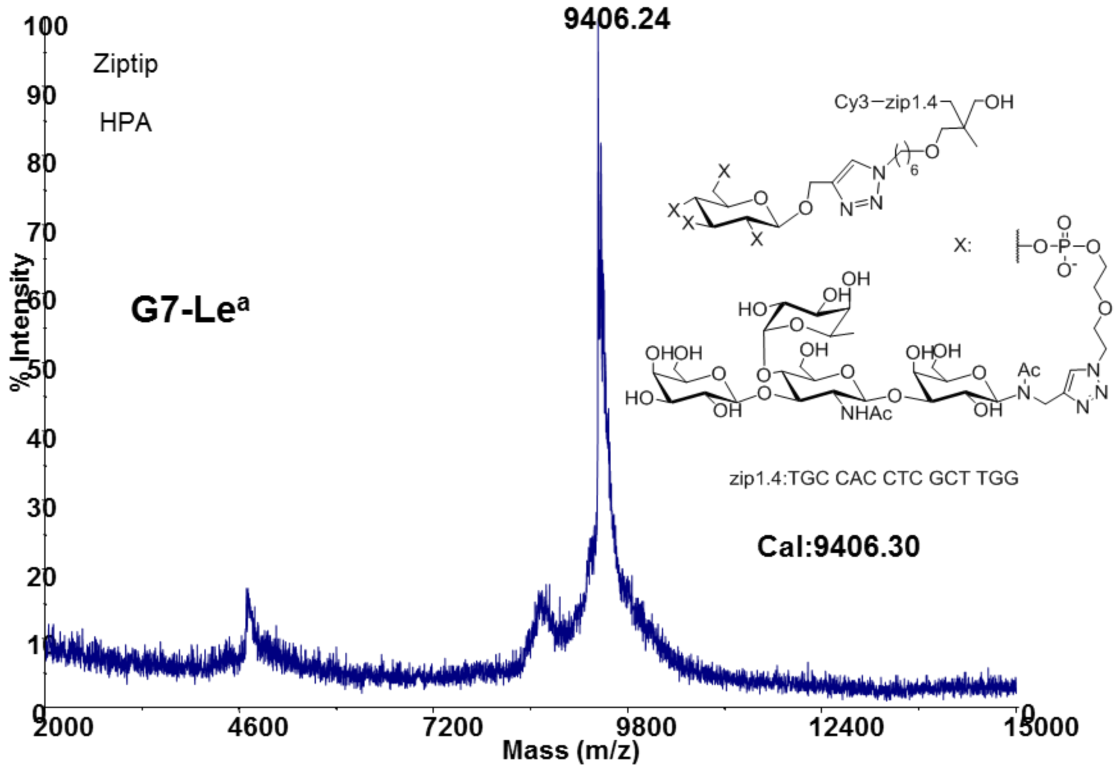
9406.24
Ziptip
HPA
G7-Le^a
% Intensity
100
90
80
70
60
50
40
30
20
10
0
2000
4600
7200
9800
12400
15000
Mass (m/z)
Cy3−zip1.4
OH
X:
zip1.4:TGC CAC CTC GCT TGG
Cal:9406.30

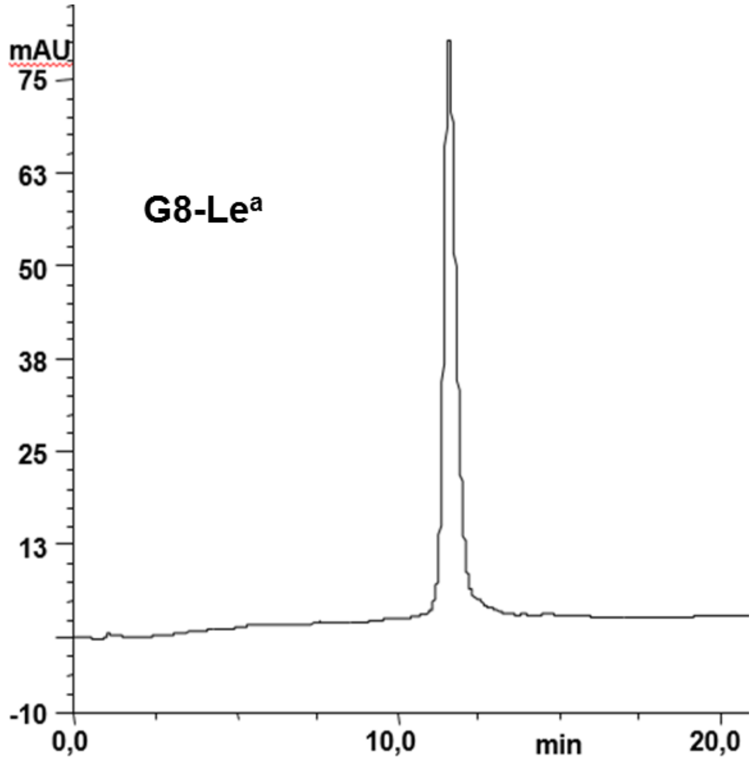
mAU
75
63
50
38
25
13
-10
0,0
10,0
min
20,0
G8-Le^a

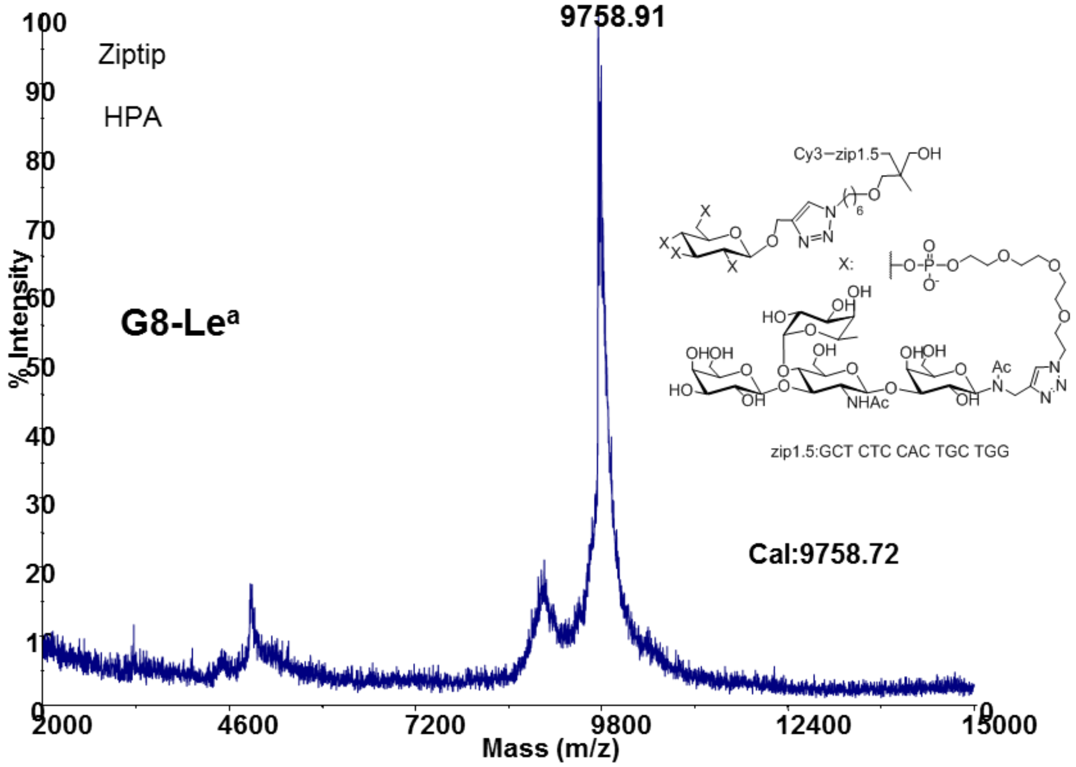
9758.91
Ziptip
HPA
G8-Le^a
% Intensity
Cy3−zip1.5
X:
zip1.5:GCT CTC CAC TGC TGG
Cal:9758.72
Mass (m/z)
2000
4600
7200
9800
12400
15000

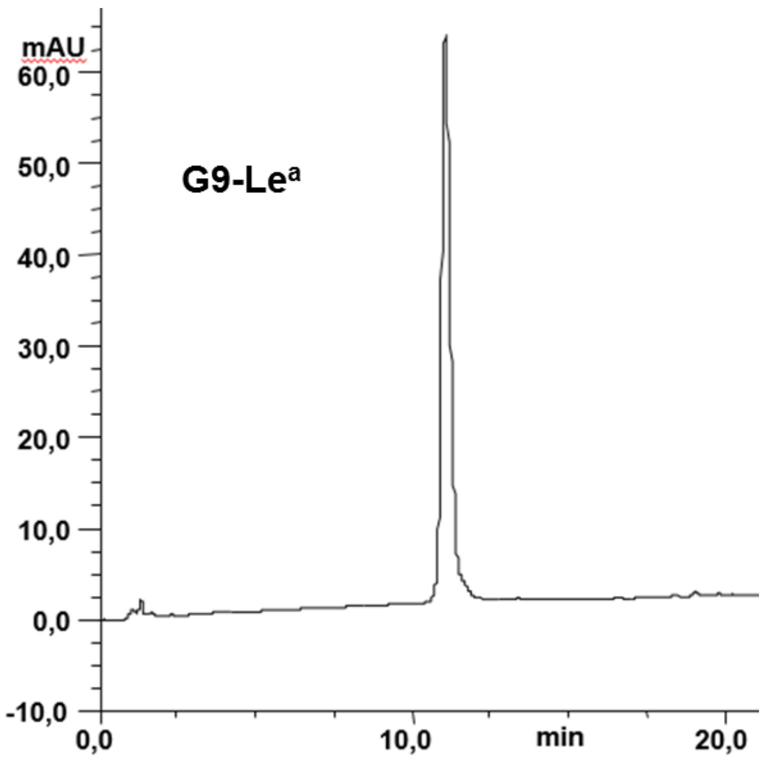
mAU
60,0
50,0
40,0
30,0
20,0
10,0
0,0
-10,0
G9-Le^a
0,0
10,0
min
20,0

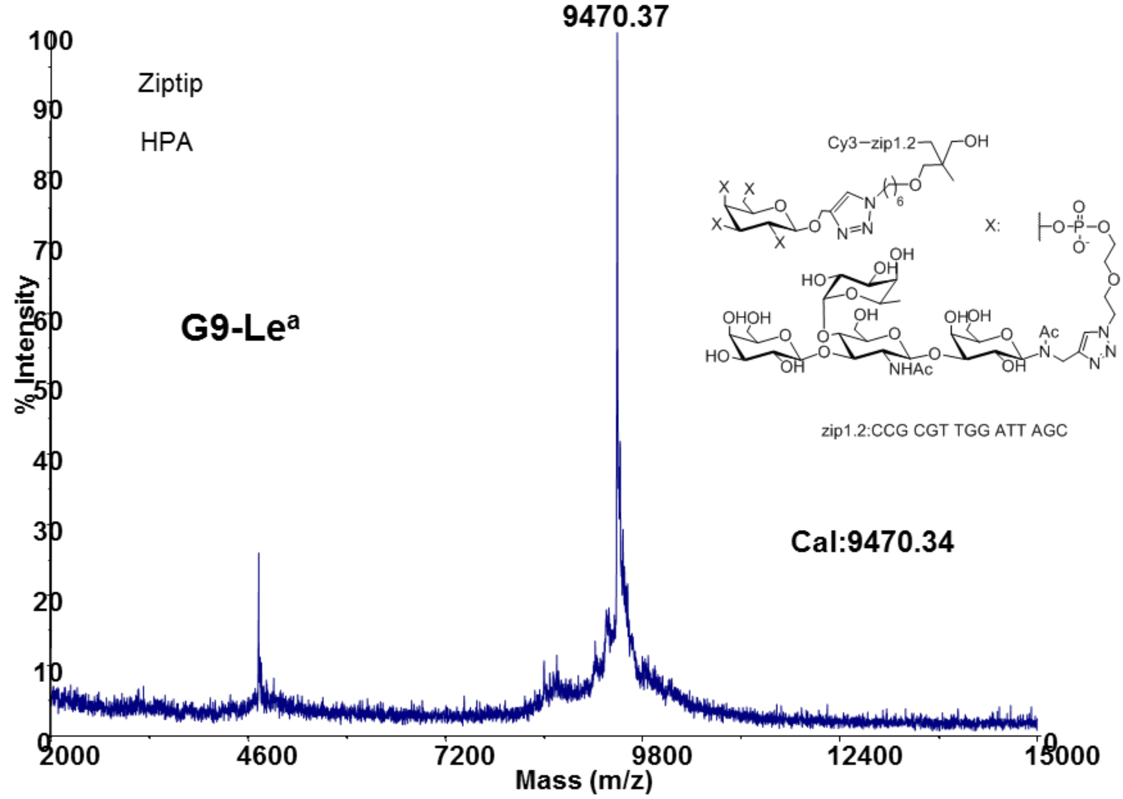
9470.37
100
90
80
70
60
50
40
30
20
10
0
Ziptip
HPA
G9-Le^a
% Intensity
Cy3−zip1.2
OH
X:
NHAc
zip1.2:CCG CGT TGG ATT AGC
Cal:9470.34
2000
4600
7200
9800
12400
15000
Mass (m/z)

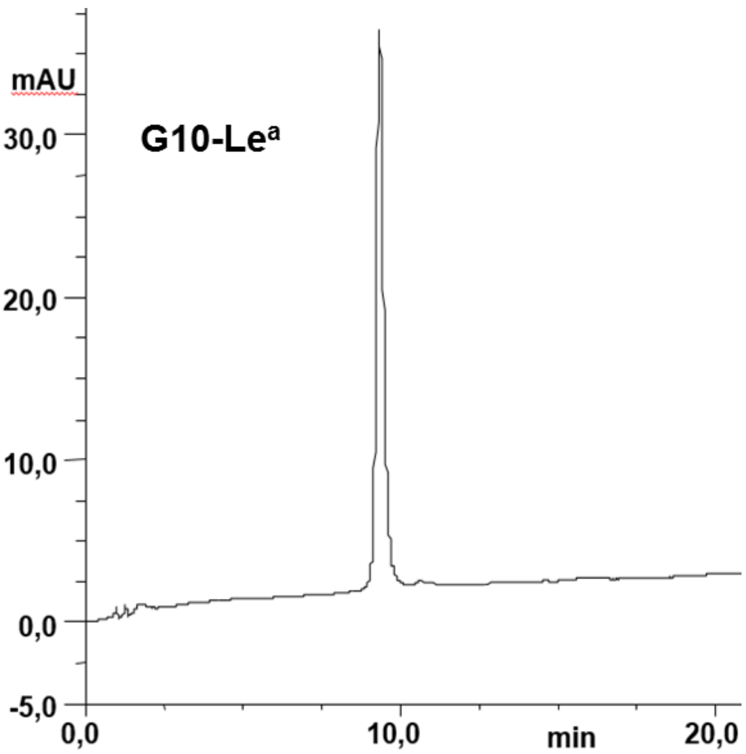
mAU
G10-Le^a
30,0
20,0
10,0
0,0
-5,0
0,0
10,0
min
20,0

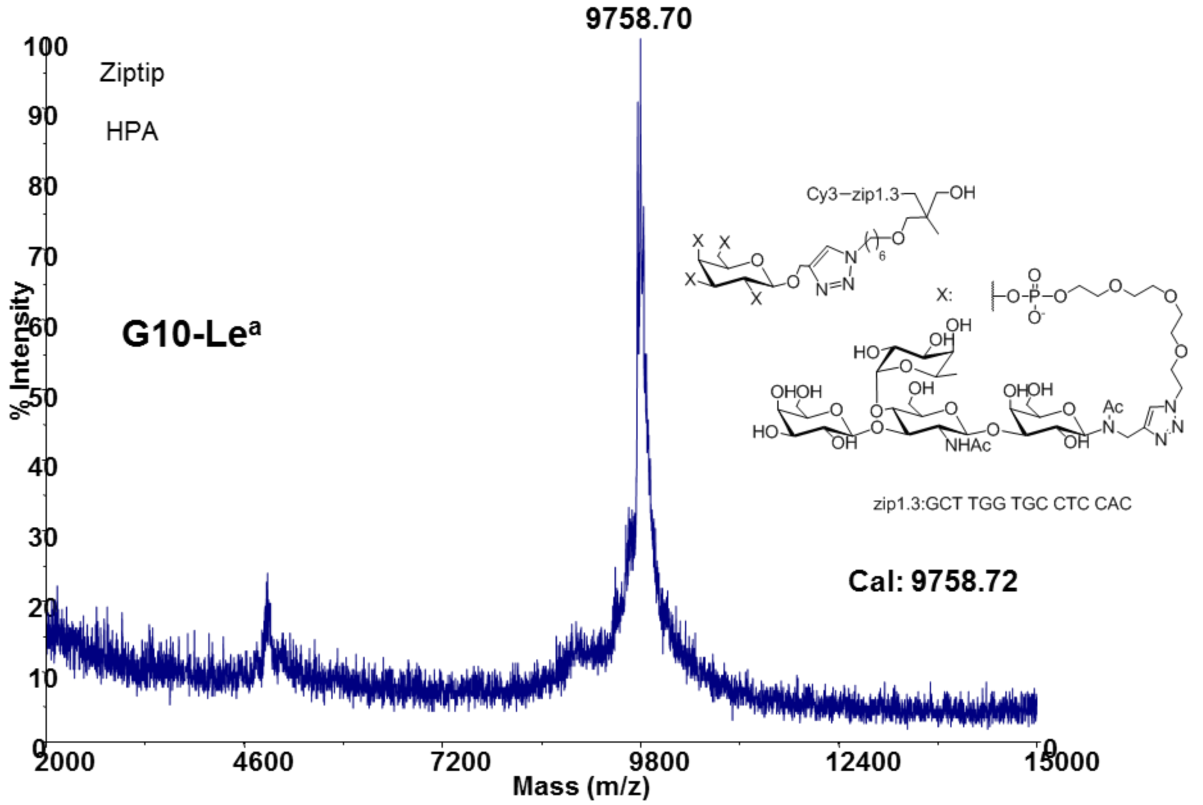
9758.70
Ziptip
HPA
G10-Le^a
% Intensity
Mass (m/z)
2000
4600
7200
9800
12400
15000
Cy3−zip1.3
OH
X:
NHAc
zip1.3:GCT TGG TGC CTC CAC
Cal: 9758.72

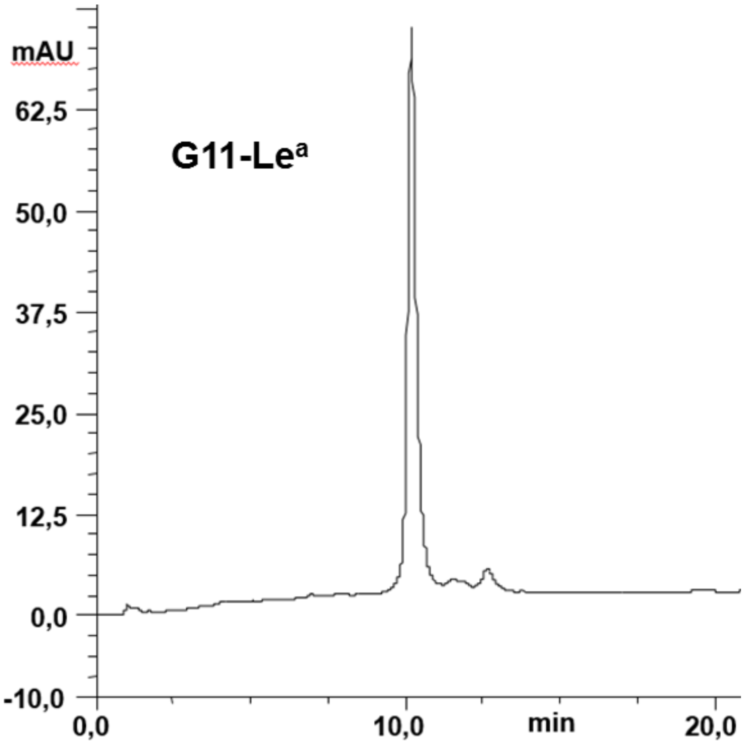
mAU
62,5
50,0
37,5
25,0
12,5
0,0
-10,0
0,0
10,0
min
20,0
G11-Le^a

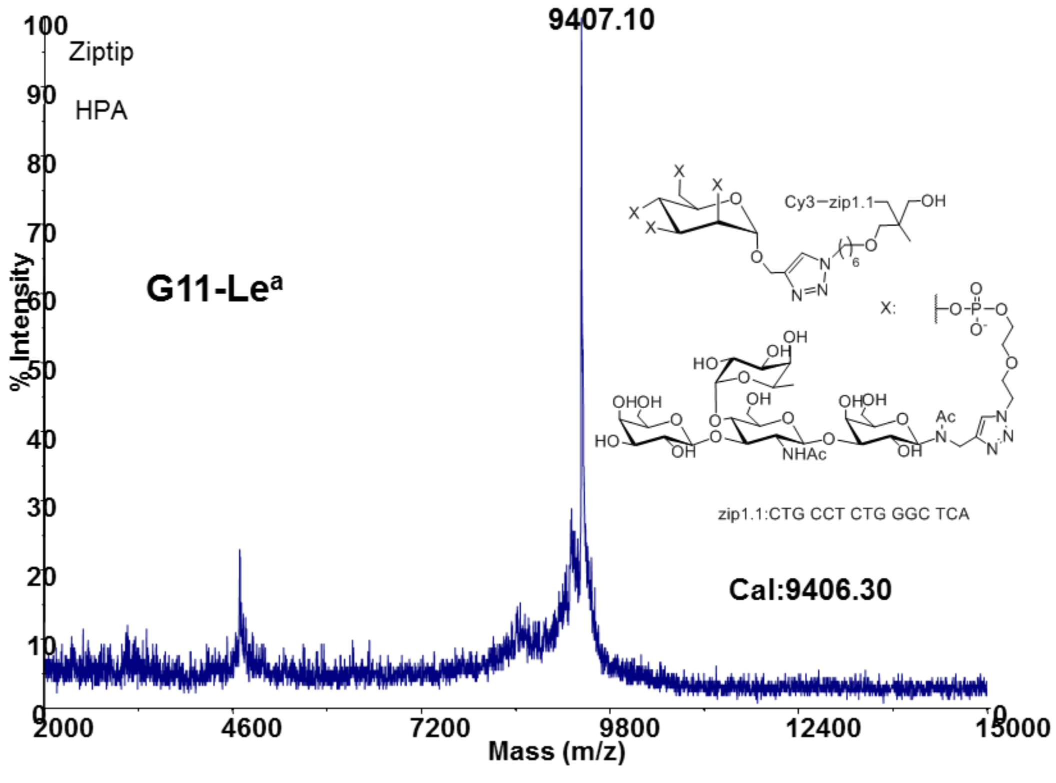
9407.10
Ziptip
HPA
G11-Le^a
% Intensity
Mass (m/z)
Cy3-zip1.1
X:
zip1.1:CTG CCT CTG GGC TCA
Cal:9406.30

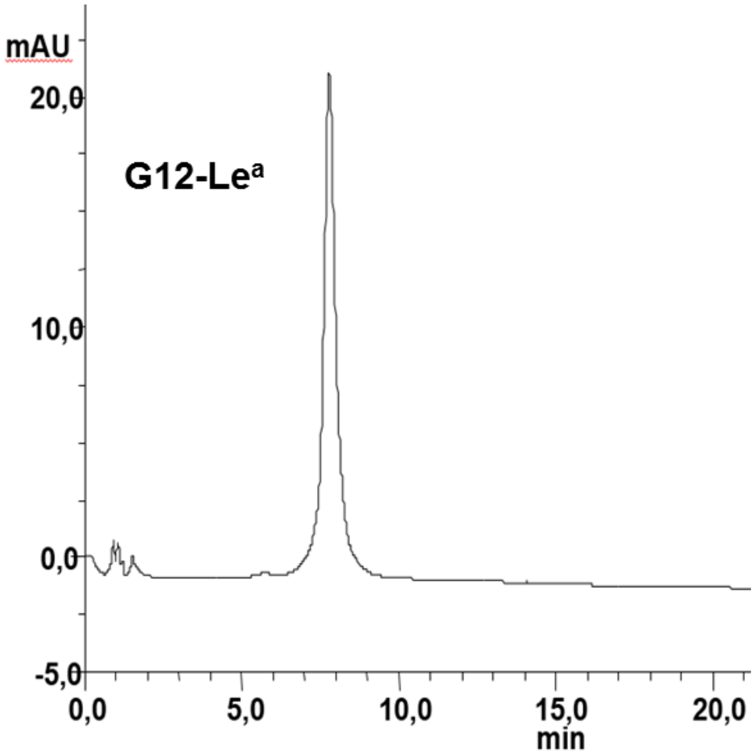
mAU
20,0
10,0
0,0
-5,0
G12-Le^a
0,0
5,0
10,0
15,0
20,0
min

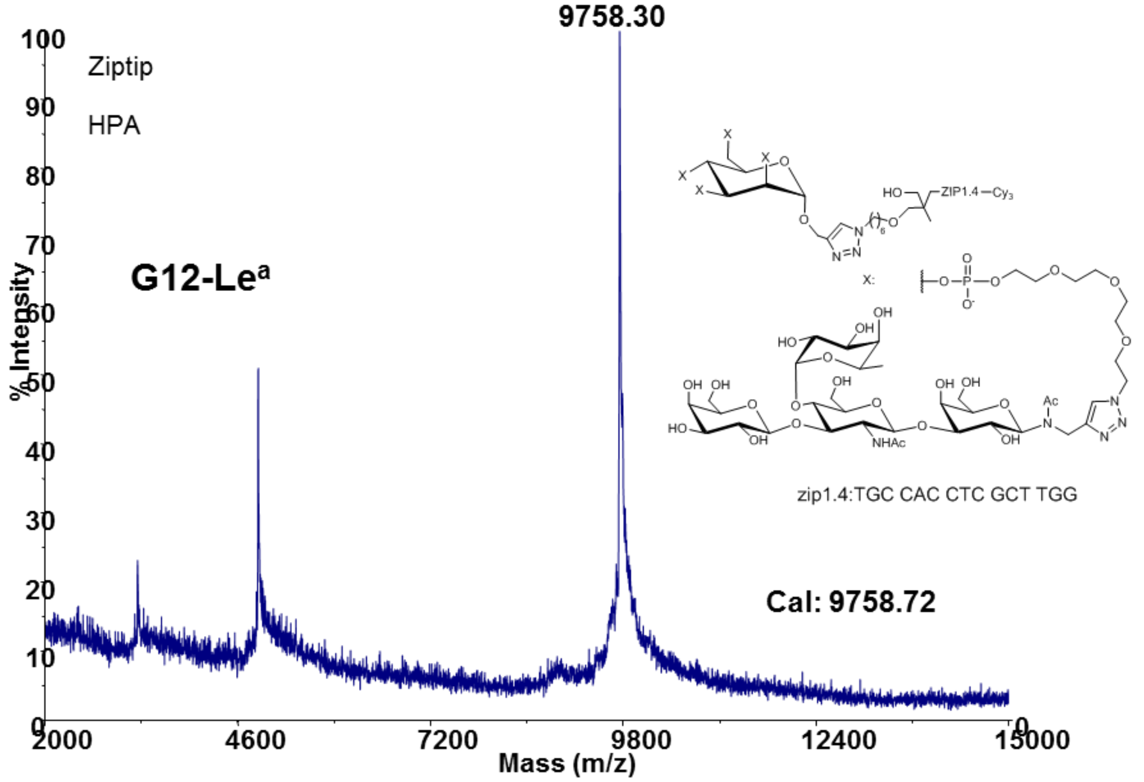
9758.30
Ziptip
HPA
G12-Le^a
% Intensity
Cal: 9758.72
zip1.4:TGC CAC CTC GCT TGG
Mass (m/z)
2000
4600
7200
9800
12400
15000

S8: HPLC chromatograms and MALDI-ToF spectra of oligoglycoclusters with Lewis[b]

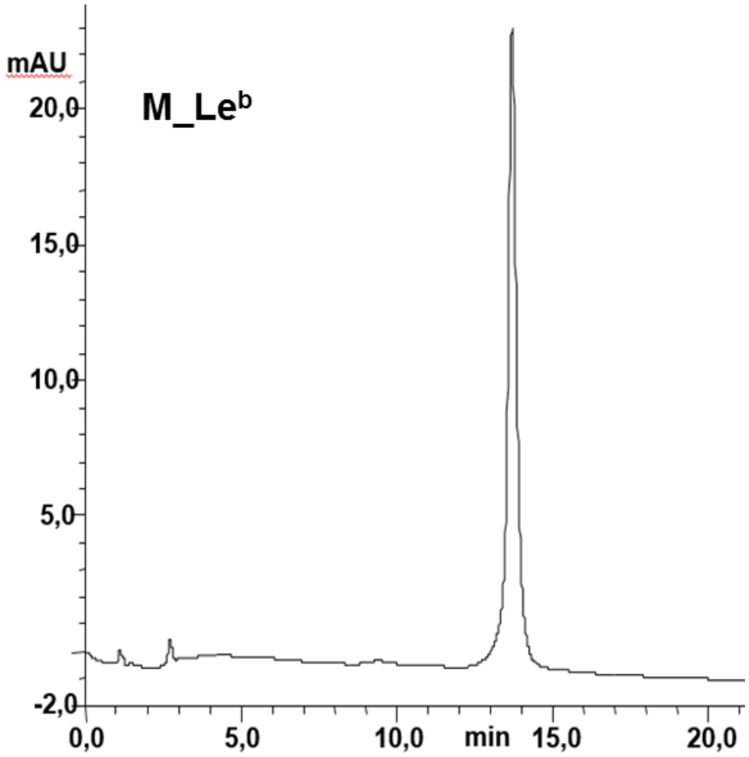

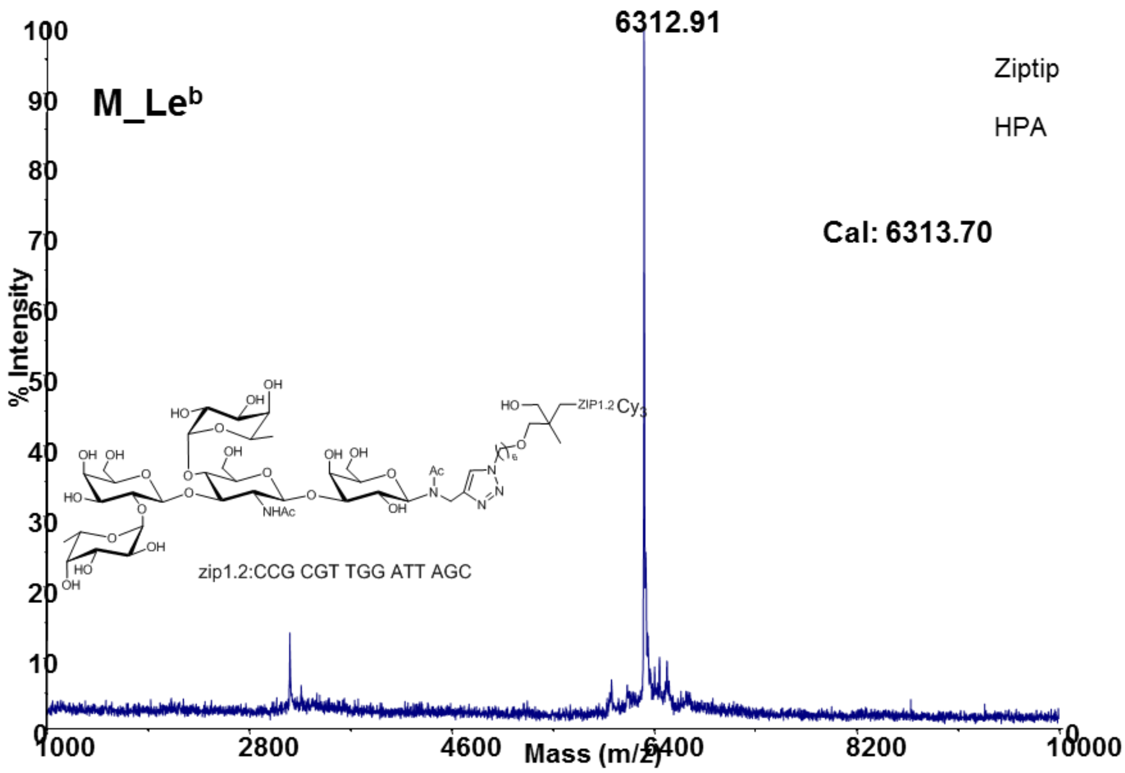

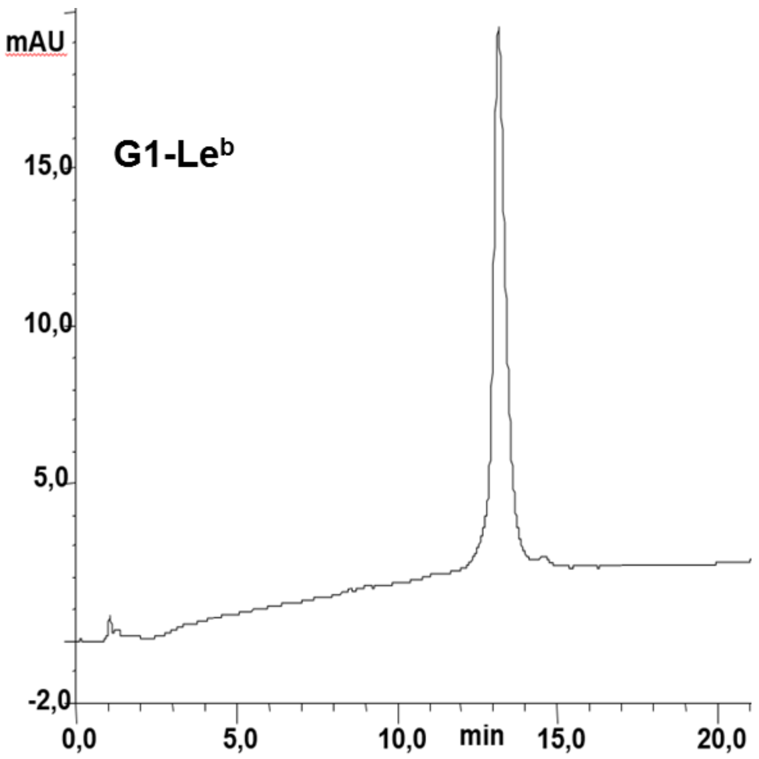
mAU
G1-Le b
15,0
10,0
5,0
-2,0
0,0
5,0
10,0
min
15,0
20,0

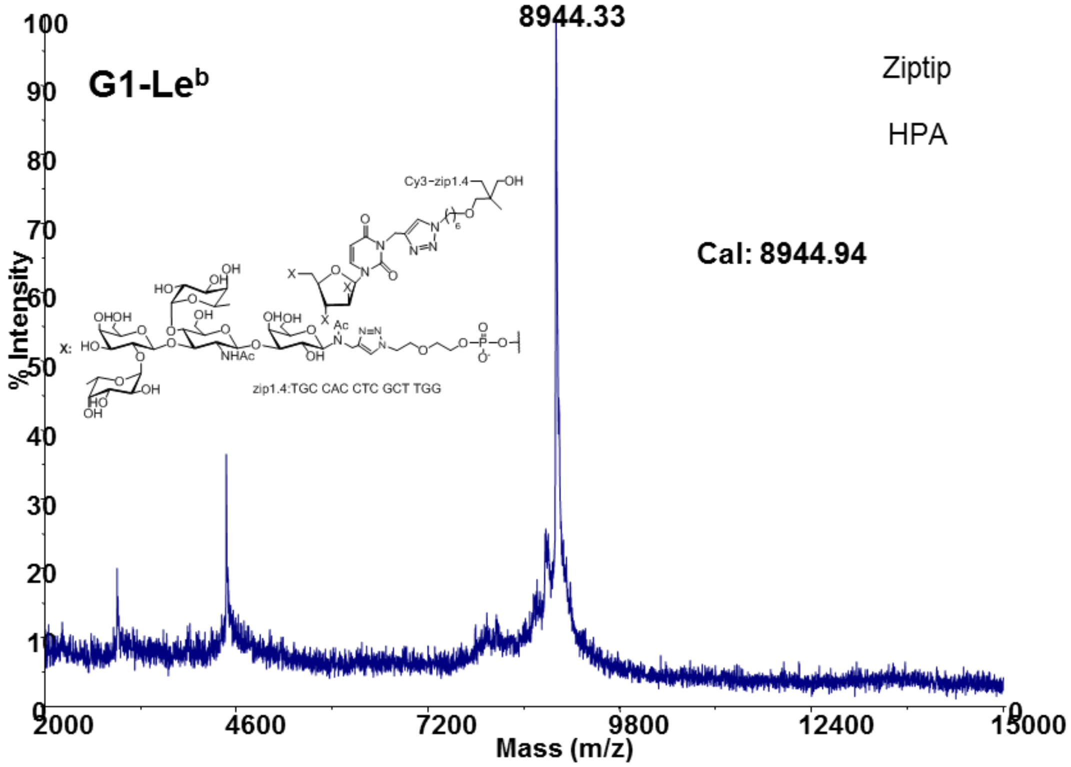
8944.33
G1-Le b
Ziptip
HPA
Cal: 8944.94
Cy3-zip1.4
zip1.4:TGC CAC CTC GCT TGG
% Intensity
Mass (m/z)
2000
4600
7200
9800
12400
15000
100
90
80
70
60
50
40
30
20
10
0

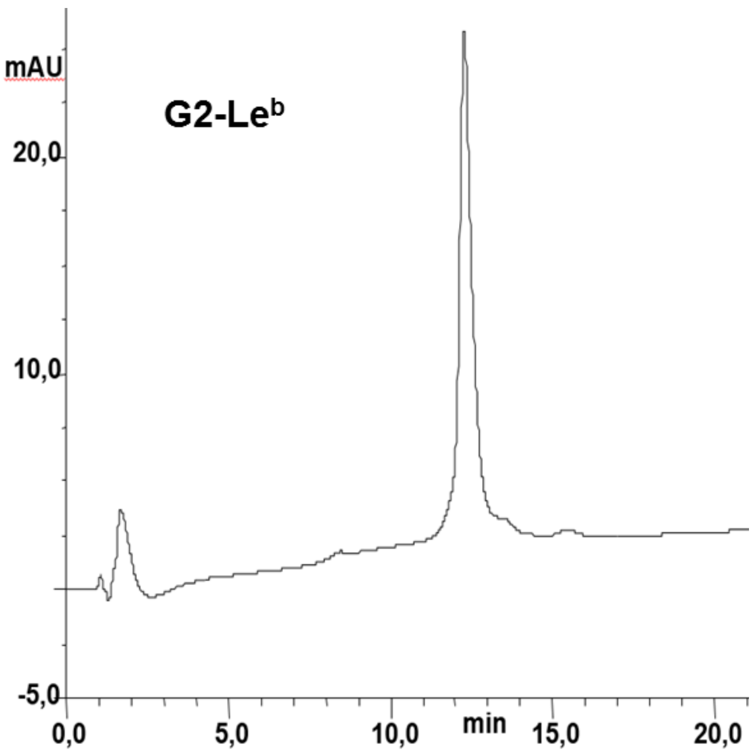
mAU
G2-Le^b
20,0
10,0
-5,0
0,0
5,0
10,0
min
15,0
20,0

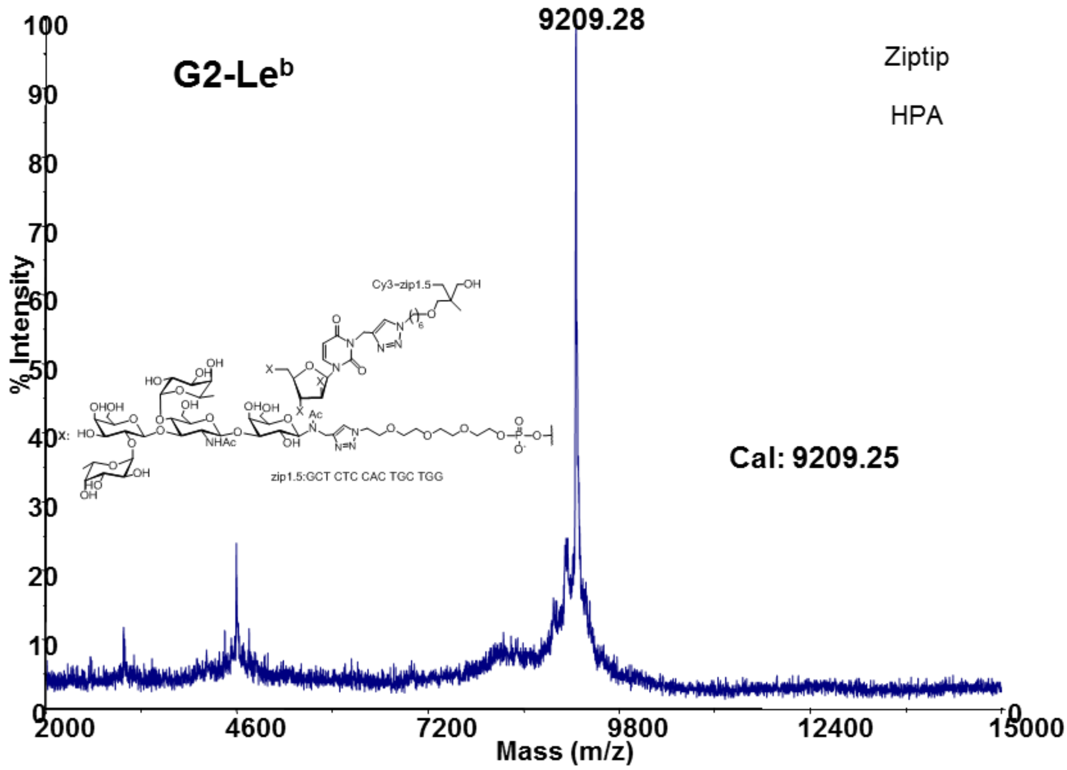
9209.28
G2-Le^b
Ziptip
HPA
% Intensity
Cy3-zip1.5
zip1.5:GCT CTC CAC TGC TGG
Cal: 9209.25
Mass (m/z)
2000
4600
7200
9800
12400
15000

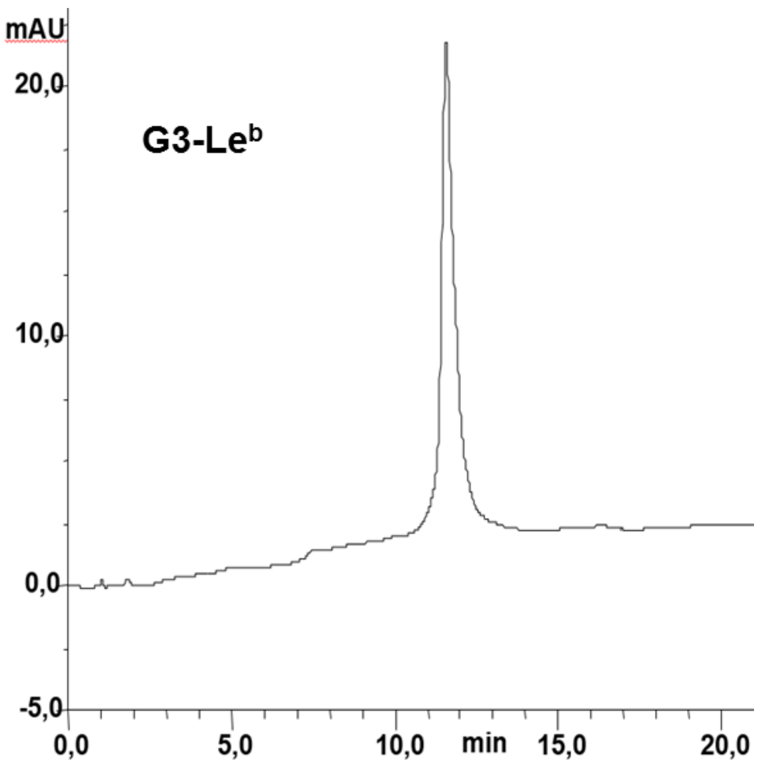
mAU
20,0
10,0
0,0
-5,0
G3-Le^b
0,0
5,0
10,0
min
15,0
20,0

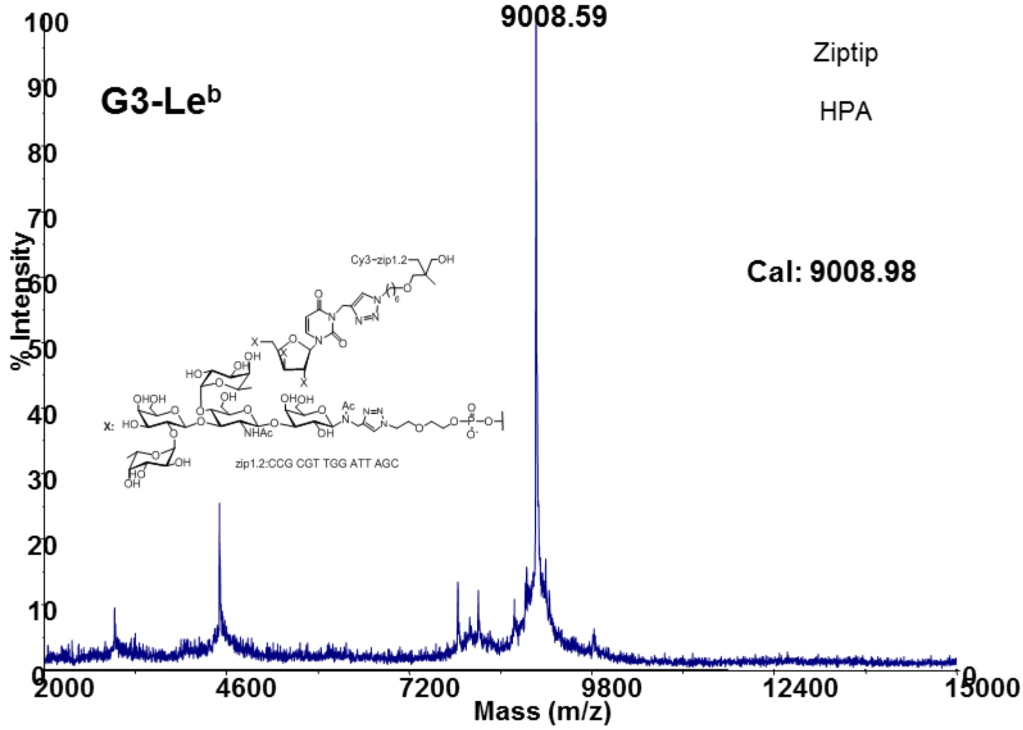
9008.59
Ziptip
HPA
G3-Le^b
Cal: 9008.98
% Intensity
Mass (m/z)
Cy3-zip1.2
zip1.2:CCG CGT TGG ATT AGC
100
90
80
70
60
50
40
30
20
10
0
2000
4600
7200
9800
12400
15000

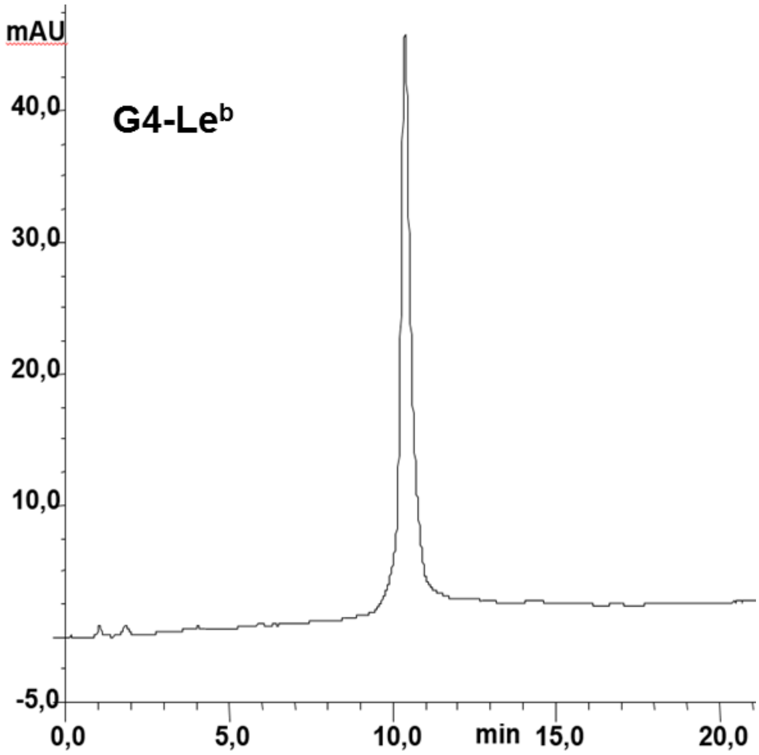

mAU
G4-Le^b
40,0
30,0
20,0
10,0
-5,0
0,0
5,0
10,0
min
15,0
20,0

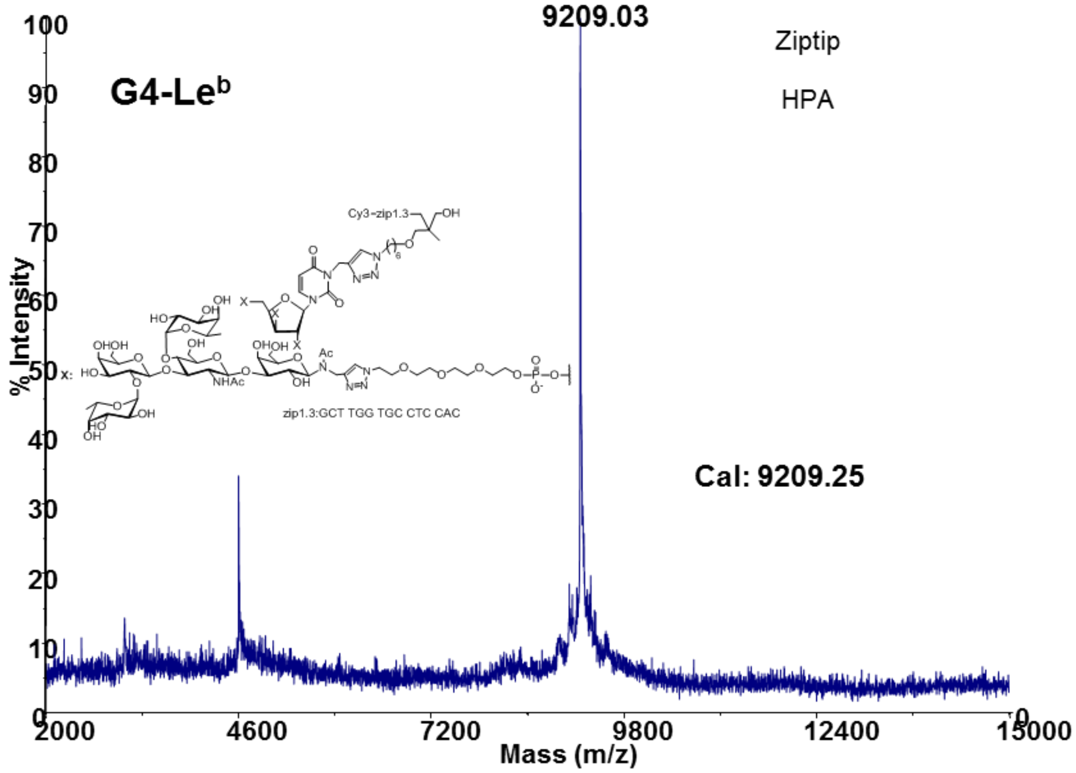

9209.03
Ziptip
HPA
G4-Le^b
Cy3-zip1.3
zip1.3:GCT TGG TGC CTC CAC
Cal: 9209.25
% Intensity
Mass (m/z)
2000
4600
7200
9800
12400
15000

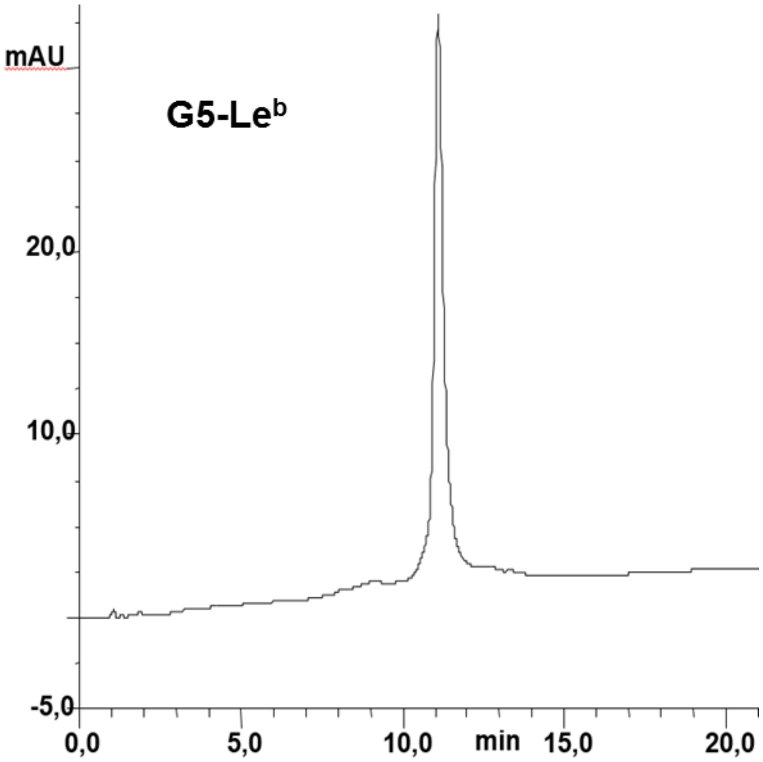
mAU
G5-Le^b
20,0
10,0
-5,0
0,0
5,0
10,0
min
15,0
20,0

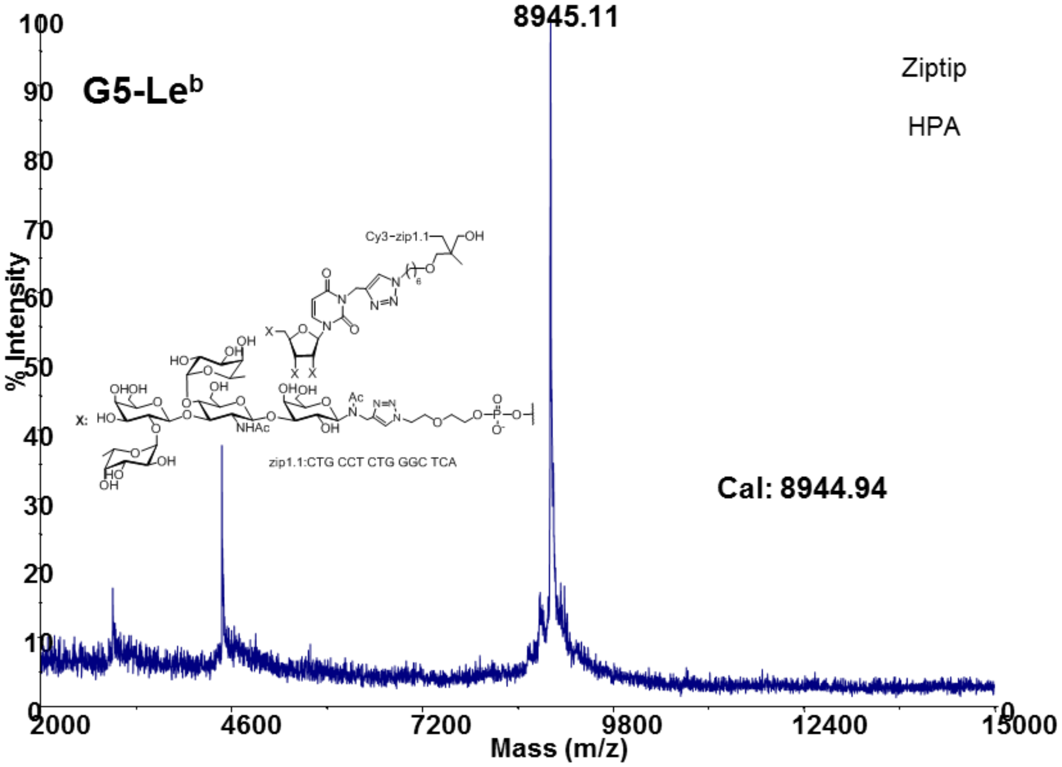
8945.11
G5-Le^b
Ziptip
HPA
Cal: 8944.94
% Intensity
Mass (m/z)
2000
4600
7200
9800
12400
15000
Cy3-zip1.1
zip1.1:CTG CCT CTG GGC TCA

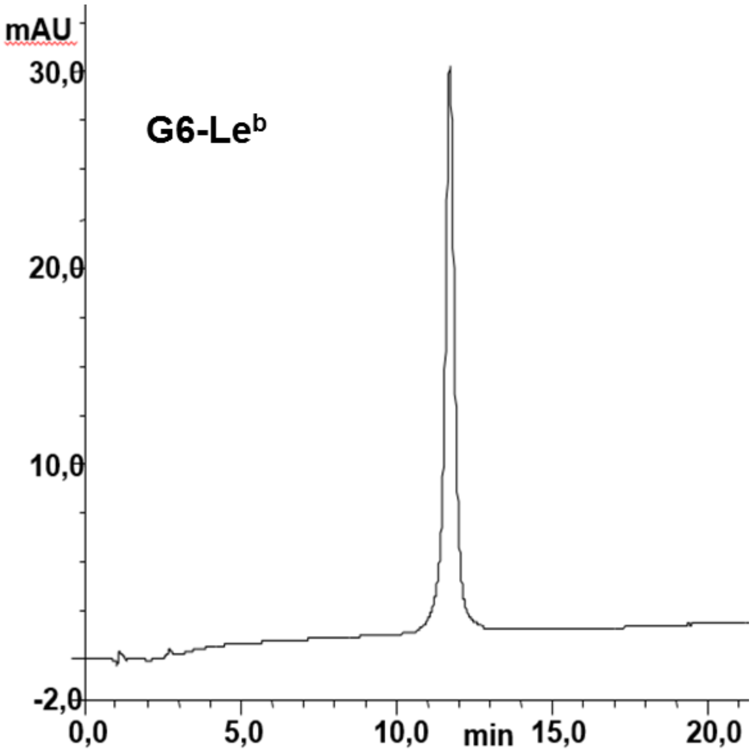

mAU
30,0
20,0
10,0
-2,0
G6-Le^b
0,0
5,0
10,0
min
15,0
20,0

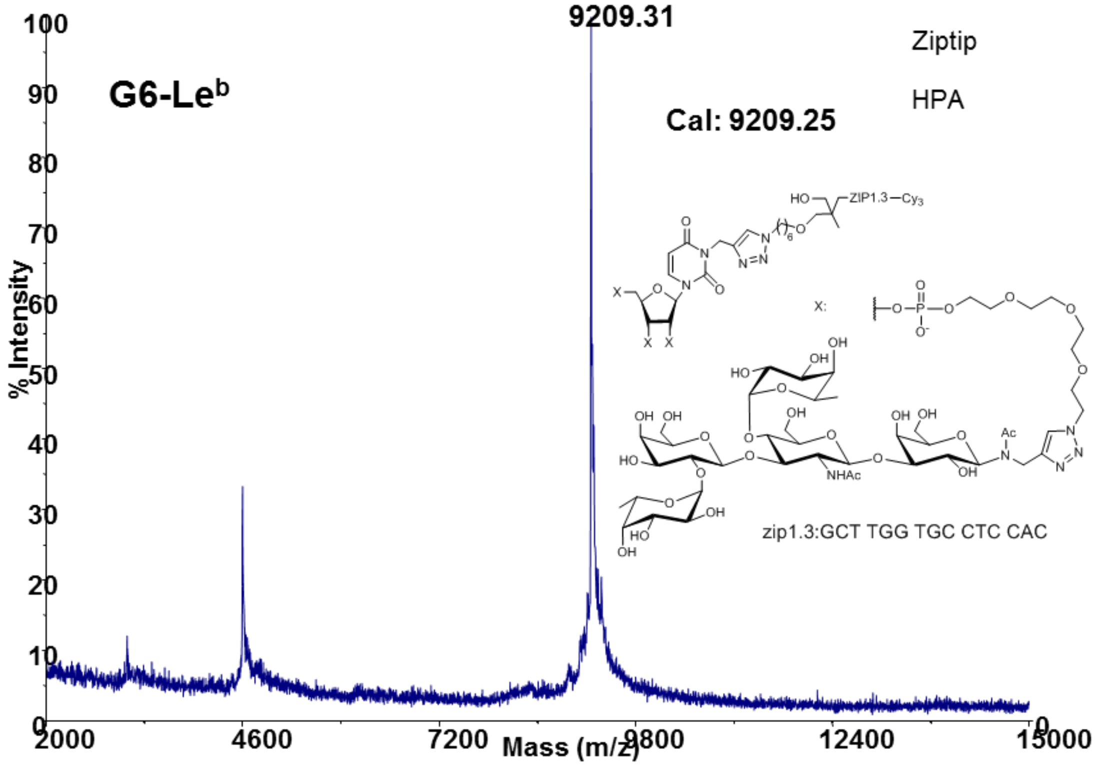

9209.31
Ziptip
HPA
G6-Le^b
Cal: 9209.25
% Intensity
Mass (m/z)
2000
4600
7200
9800
12400
15000
HO
ZIP1.3—Cy$_3$
X:
NHAc
Ac
zip1.3:GCT TGG TGC CTC CAC

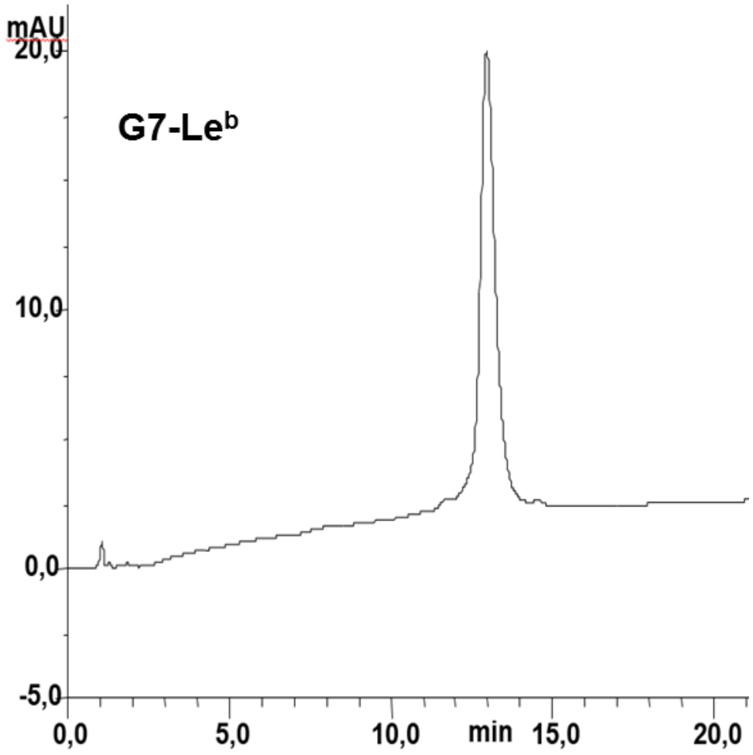
mAU
20,0
10,0
0,0
-5,0
G7-Leb
0,0
5,0
10,0
min
15,0
20,0

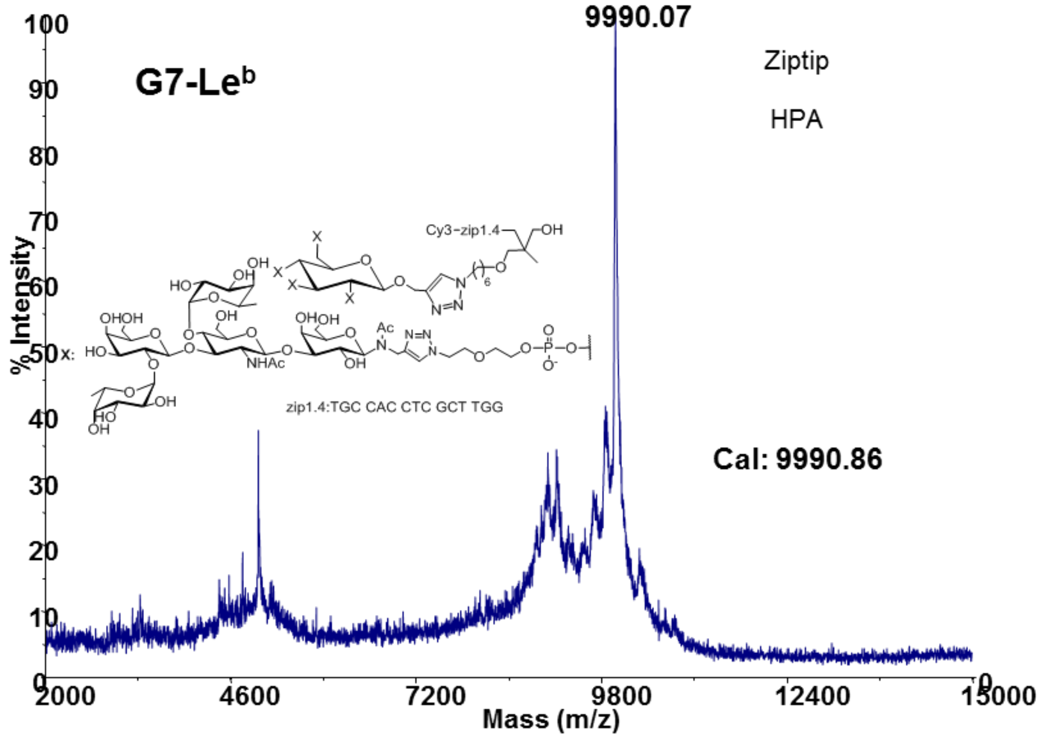
9990.07
G7-Leb
Ziptip
HPA
Cy3-zip1.4
zip1.4:TGC CAC CTC GCT TGG
Cal: 9990.86
% Intensity
100
90
80
70
60
50
40
30
20
10
0
2000
4600
7200
9800
12400
15000
Mass (m/z)

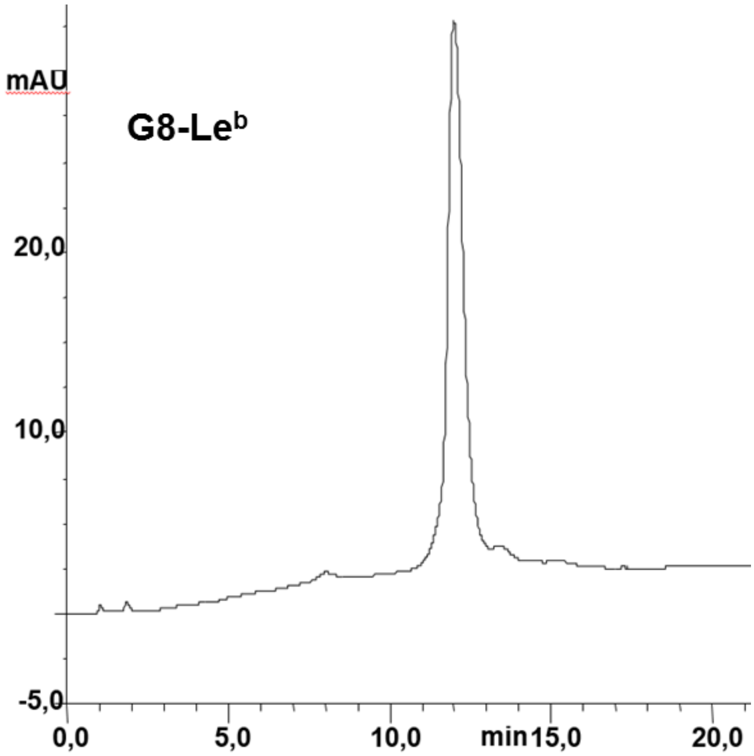
mAU
G8-Le^b
20,0
10,0
-5,0
0,0
5,0
10,0
min
15,0
20,0

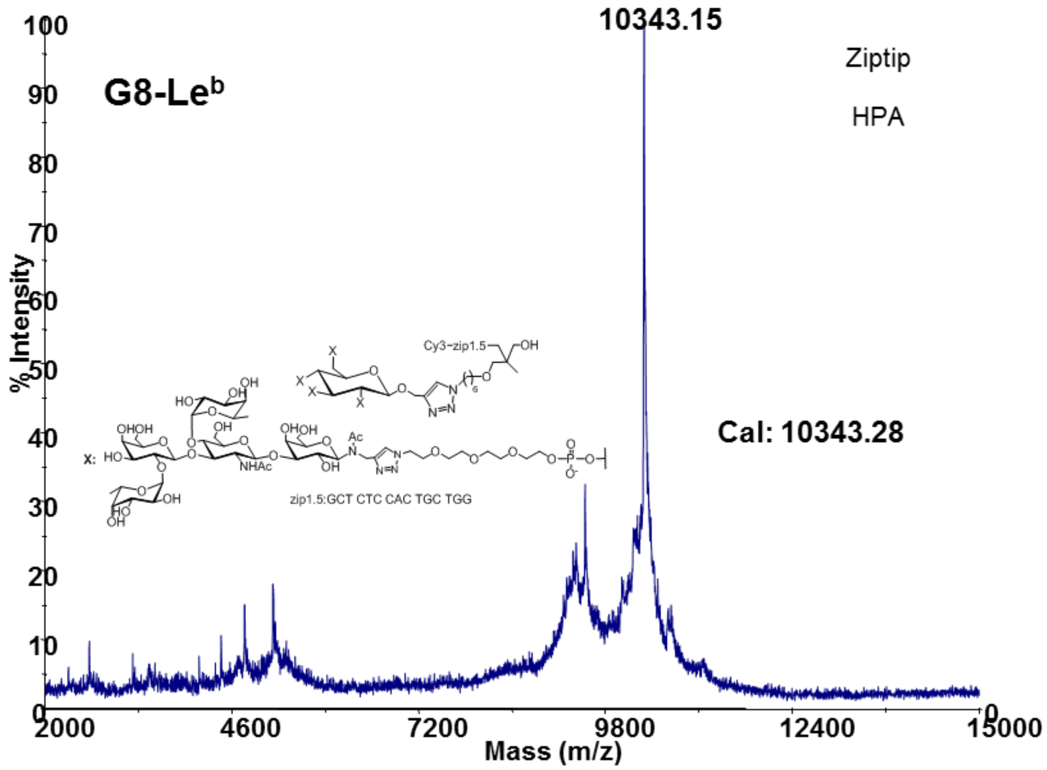
10343.15
G8-Le^b
Ziptip
HPA
Cal: 10343.28
% Intensity
Mass (m/z)
zip1.5:GCT CTC CAC TGC TGG
Cy3-zip1.5
2000
4600
7200
9800
12400
15000

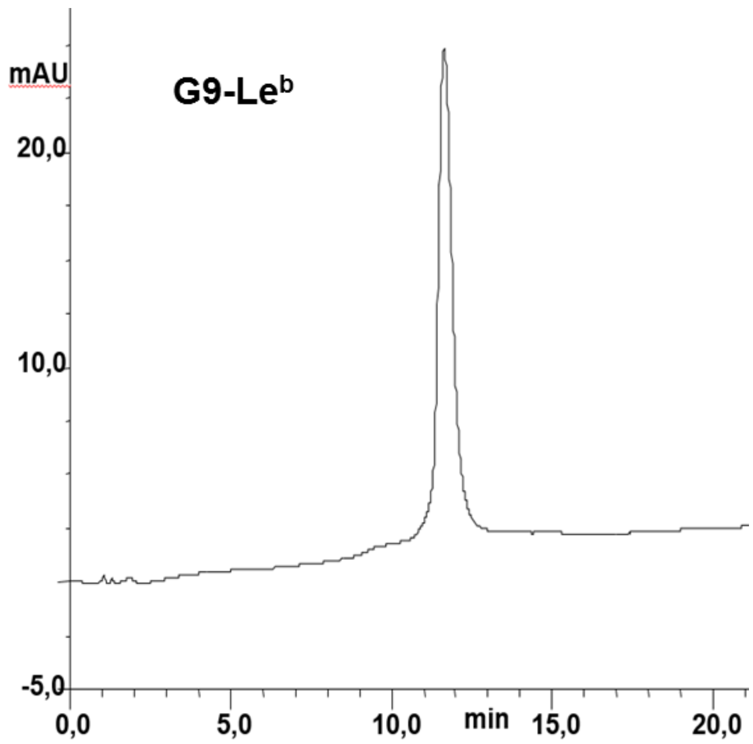
mAU
G9-Le^b
20,0
10,0
-5,0
0,0
5,0
10,0
min
15,0
20,0

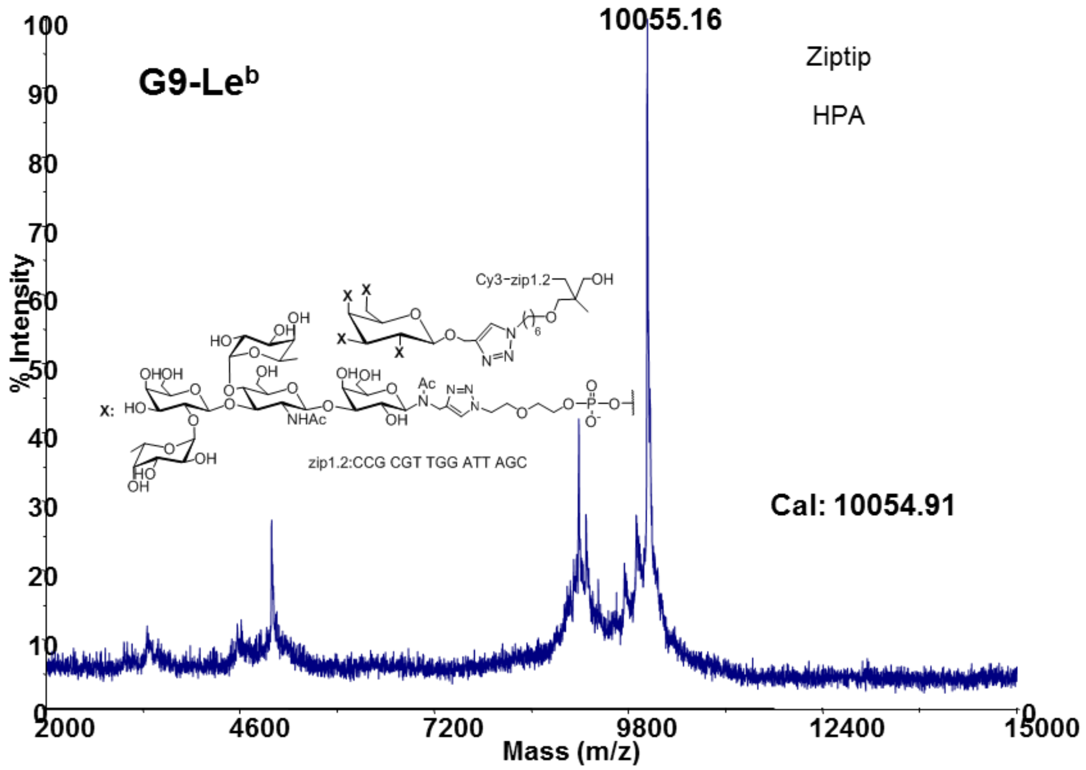
10055.16
G9-Le^b
Ziptip
HPA
% Intensity
Cy3-zip1.2
zip1.2:CCG CGT TGG ATT AGC
Cal: 10054.91
Mass (m/z)
2000
4600
7200
9800
12400
15000

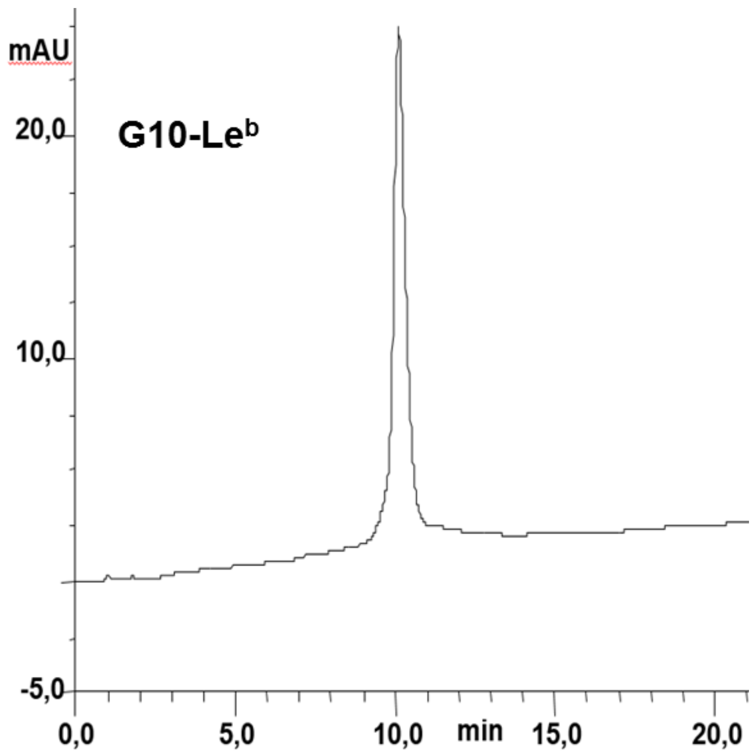
mAU
G10-Le^{b}
20,0
10,0
-5,0
0,0
5,0
10,0
min
15,0
20,0

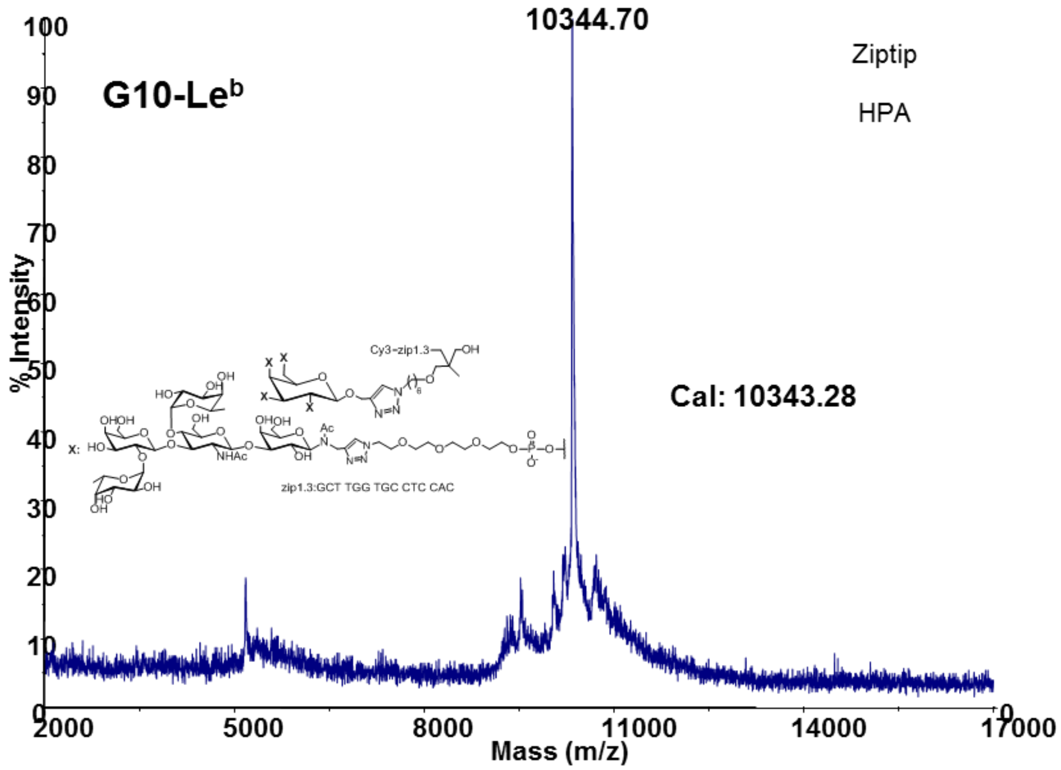
10344.70
G10-Le^{b}
Ziptip
HPA
Cal: 10343.28
Cy3-zip1.3
zip1.3:GCT TGG TGC CTC CAC
% Intensity
Mass (m/z)
2000
5000
8000
11000
14000
17000
0
10
20
30
40
50
60
70
80
90
100

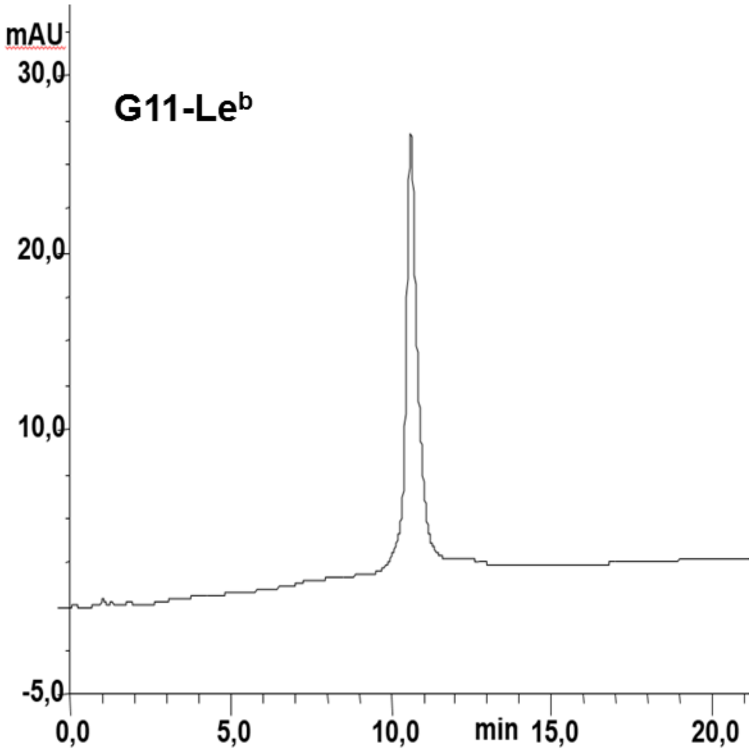

mAU
30,0
20,0
10,0
-5,0
G11-Le^b
0,0
5,0
10,0
min 15,0
20,0

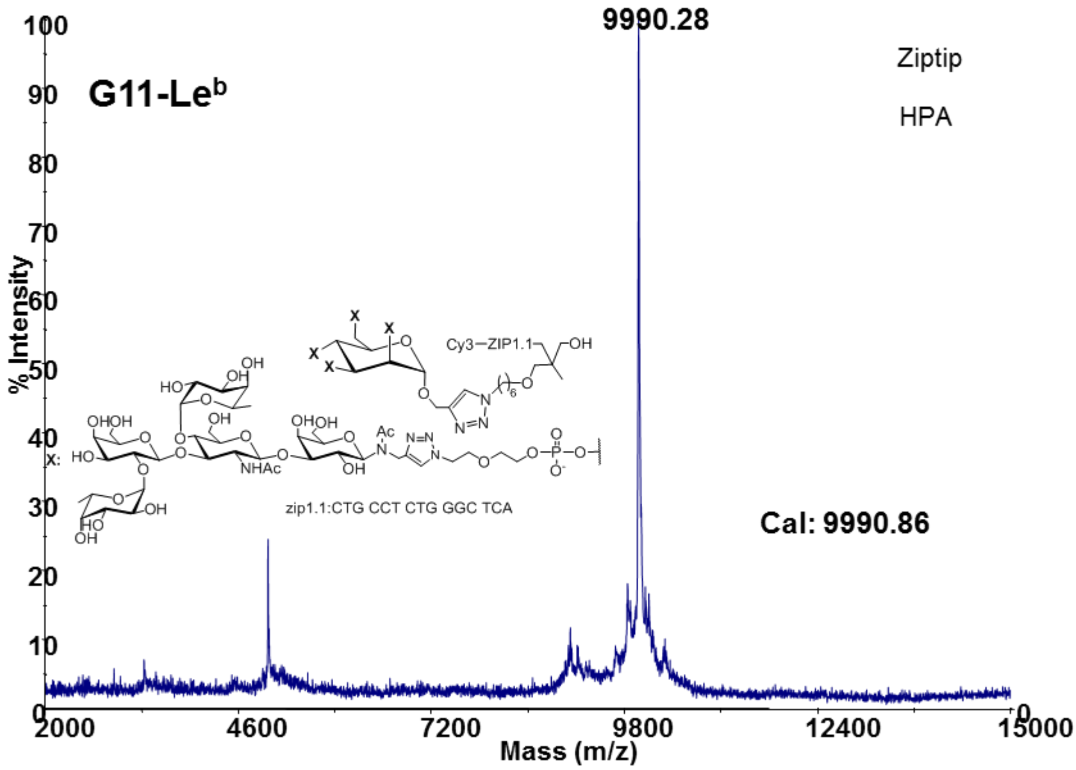

100
90
80
70
60
50
40
30
20
10
0
% Intensity
G11-Le^b
9990.28
Ziptip
HPA
Cal: 9990.86
Cy3—ZIP1.1
zip1.1:CTG CCT CTG GGC TCA
2000
4600
7200
9800
12400
15000
Mass (m/z)

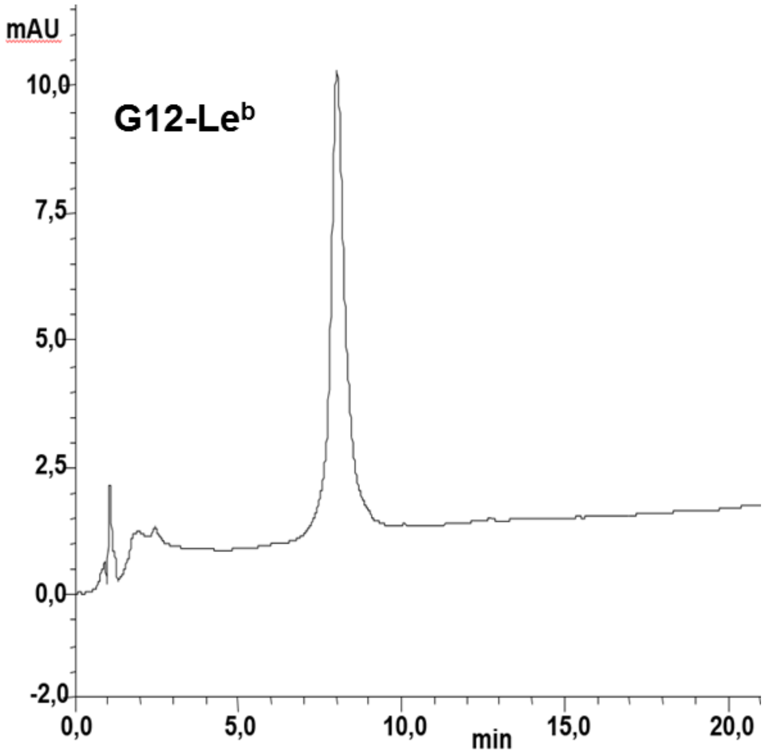
mAU
G12-Le^b
10,0
7,5
5,0
2,5
0,0
-2,0
0,0
5,0
10,0
15,0
20,0
min

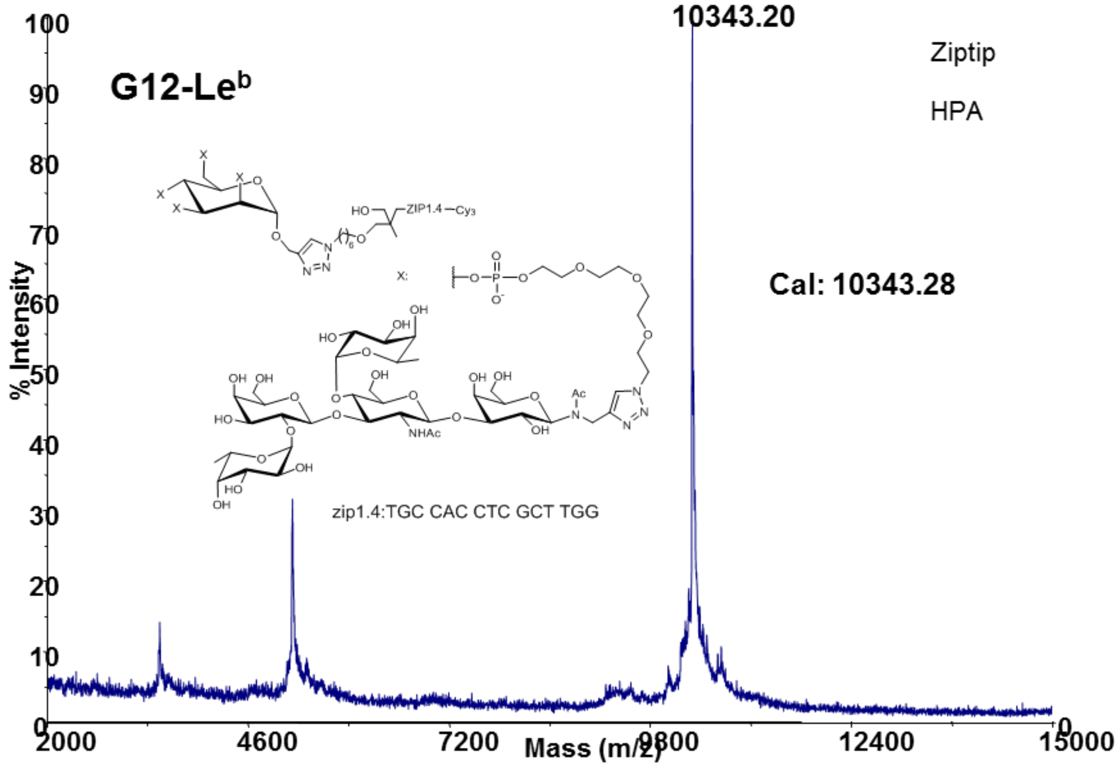
10343.20
Ziptip
HPA
G12-Le^b
Cal: 10343.28
ZIP1.4—Cy3
zip1.4:TGC CAC CTC GCT TGG
% Intensity
Mass (m/z)
2000
4600
7200
9800
12400
15000

S9: HPLC chromatograms and MALDI-ToF spectra of oligoglycoclusters with Lewis^x

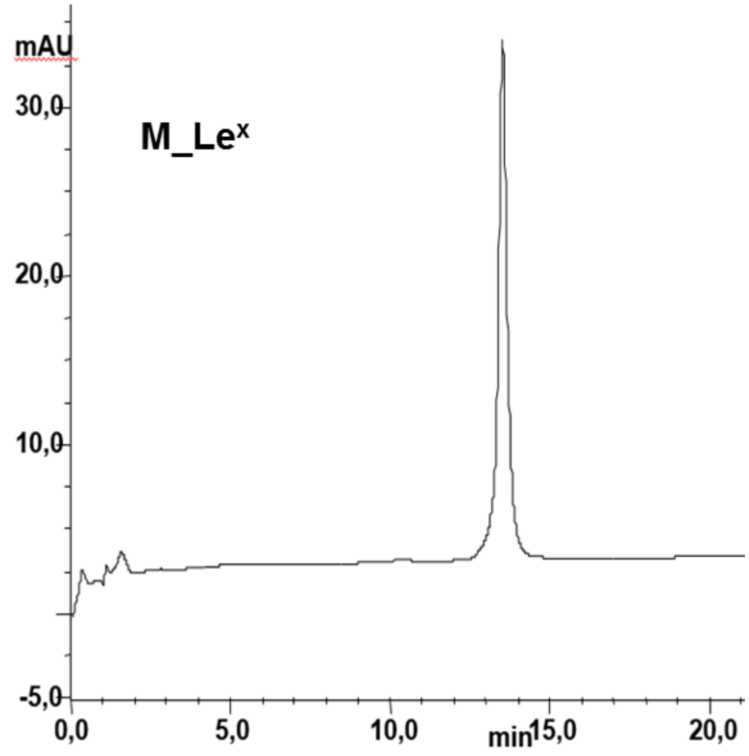

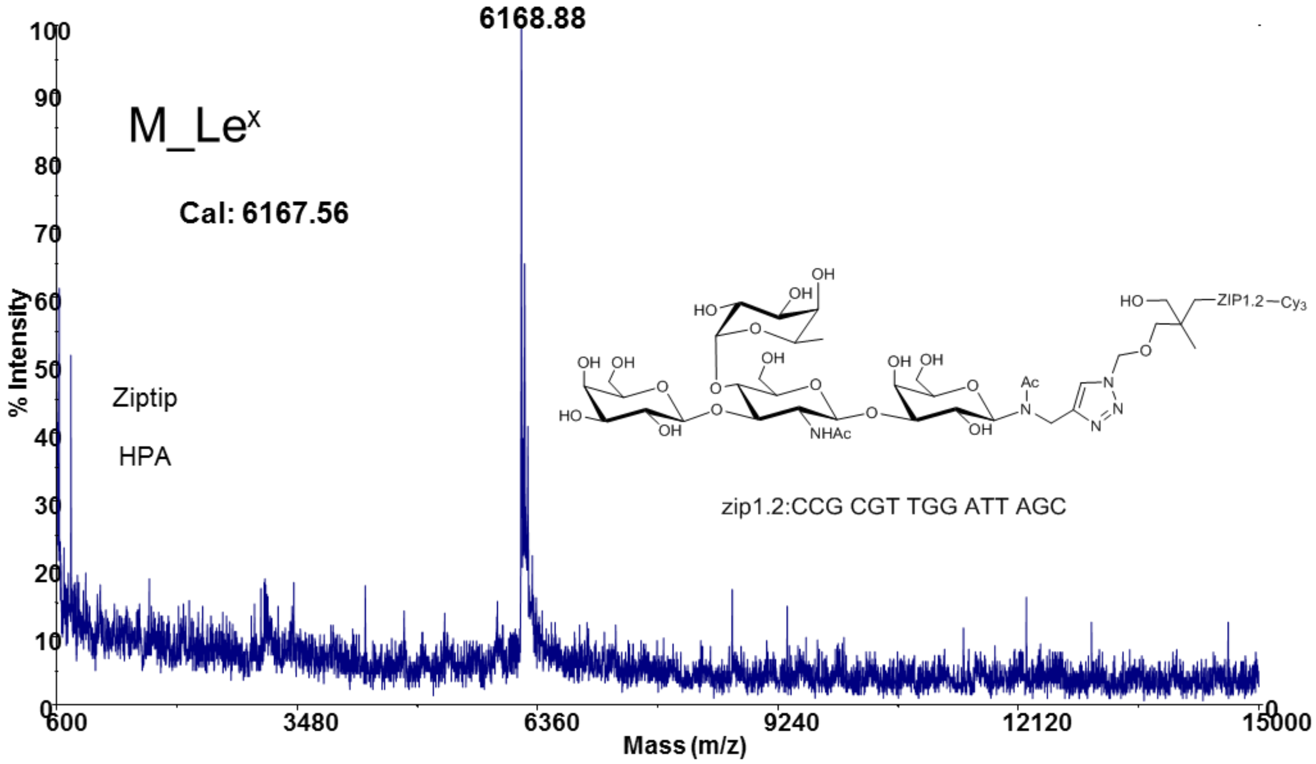

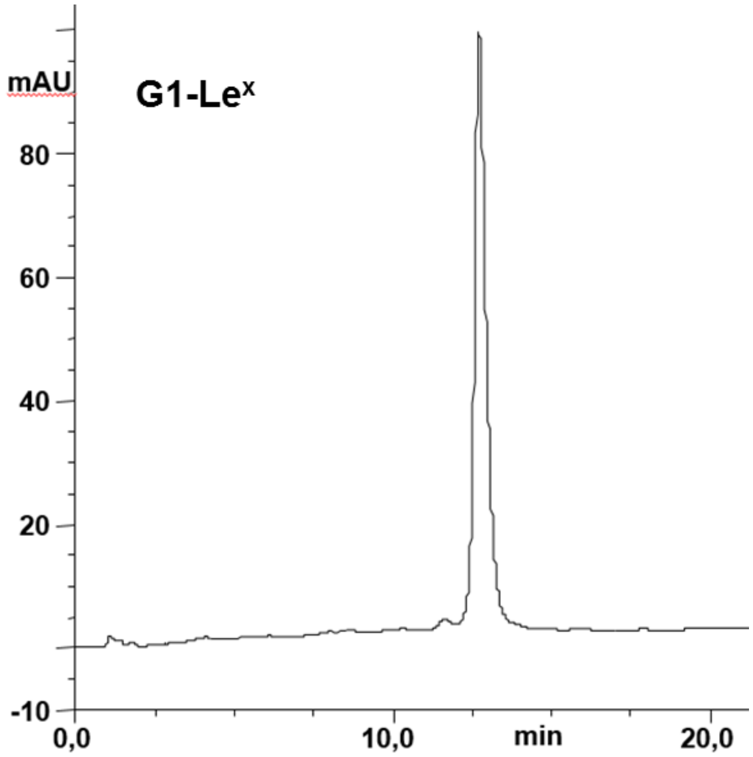
mAU
G1-Le^x
80
60
40
20
-10
0,0
10,0
min
20,0

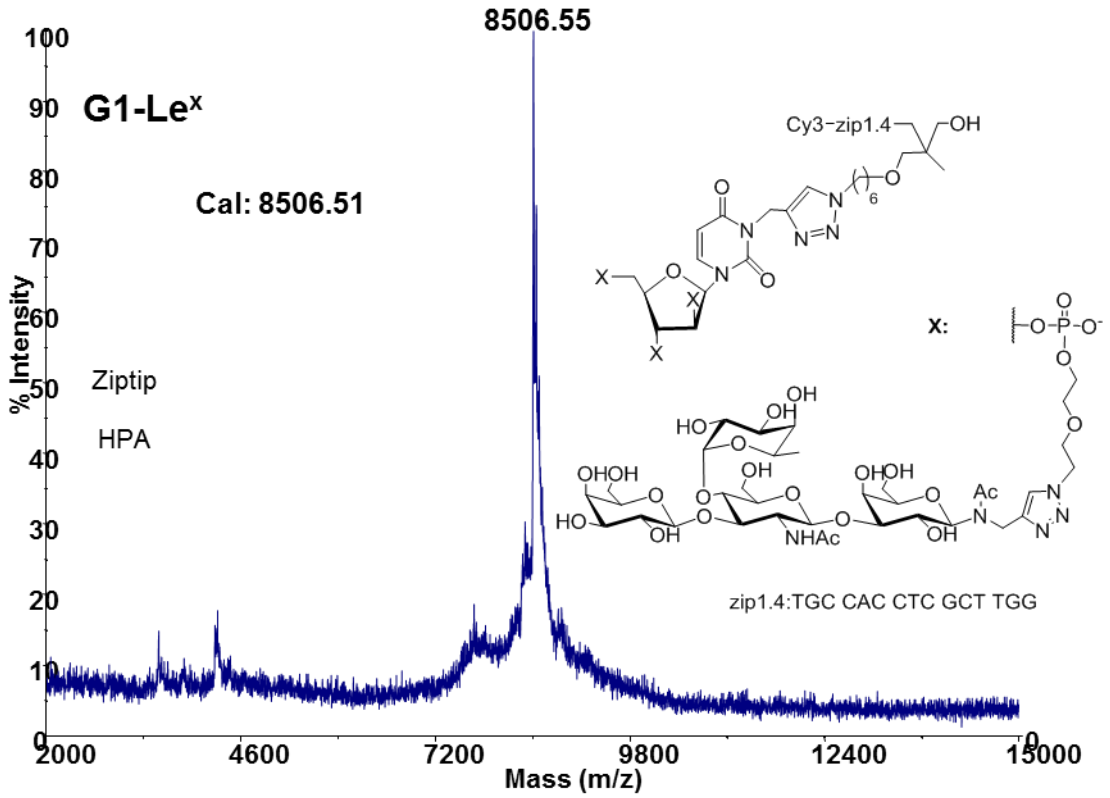
8506.55
G1-Le^x
Cal: 8506.51
Ziptip
HPA
% Intensity
Mass (m/z)
2000
4600
7200
9800
12400
15000
Cy3-zip1.4
X:
zip1.4:TGC CAC CTC GCT TGG

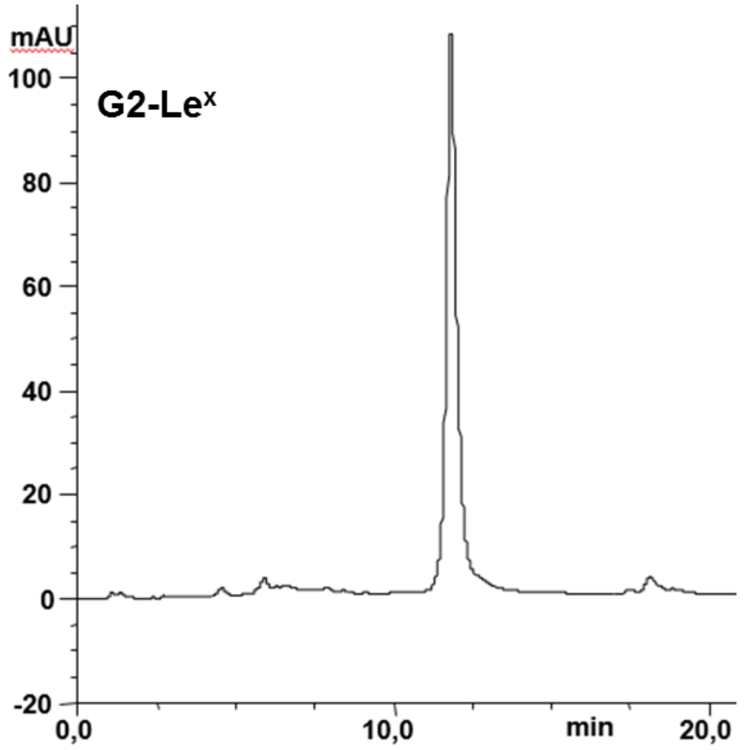
mAU
100
80
60
40
20
0
-20
0,0
10,0
min
20,0
G2-Le^x

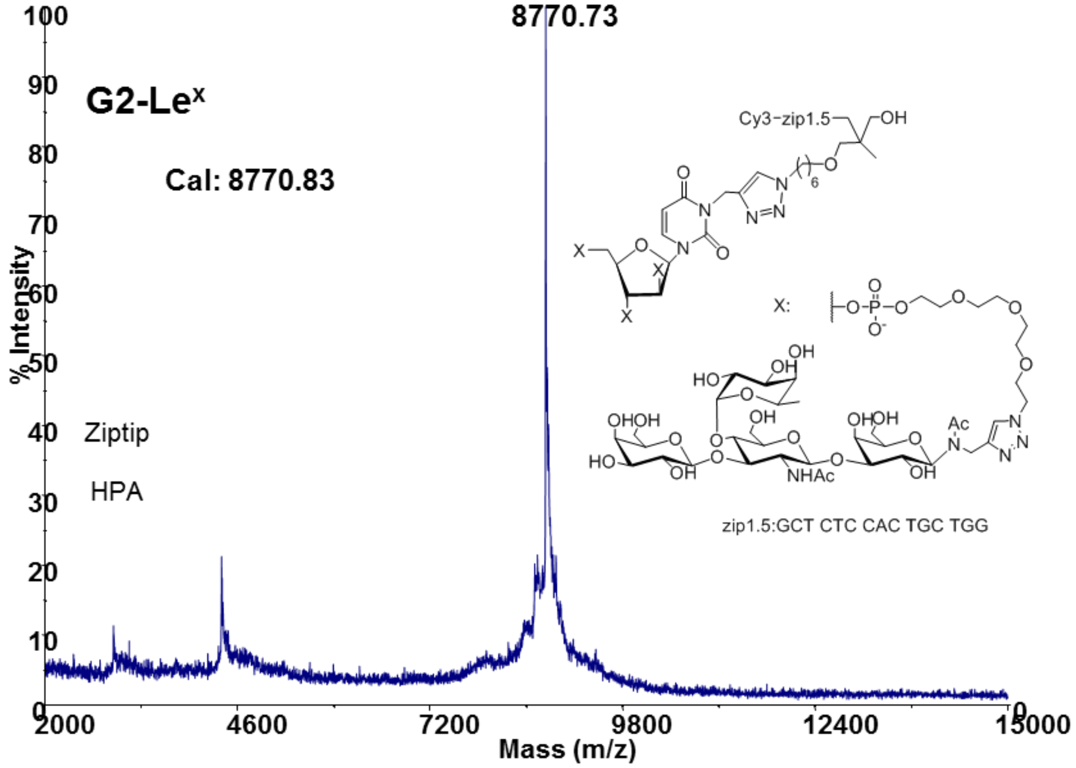
8770.73
G2-Le^x
Cal: 8770.83
Ziptip
HPA
% Intensity
Mass (m/z)
2000
4600
7200
9800
12400
15000
Cy3-zip1.5
zip1.5:GCT CTC CAC TGC TGG

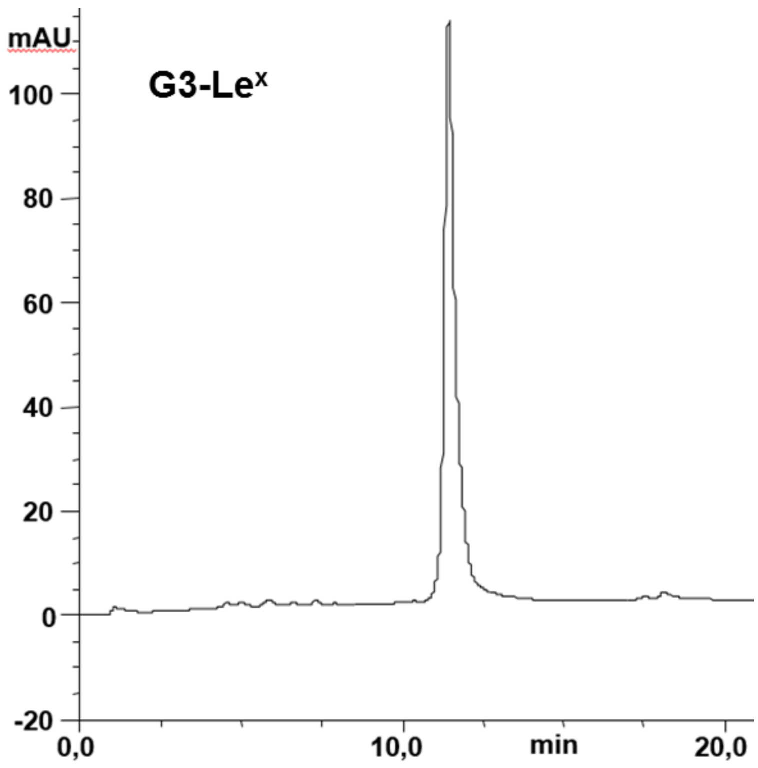
mAU
G3-Lex
100
80
60
40
20
0
-20
0,0
10,0
min
20,0

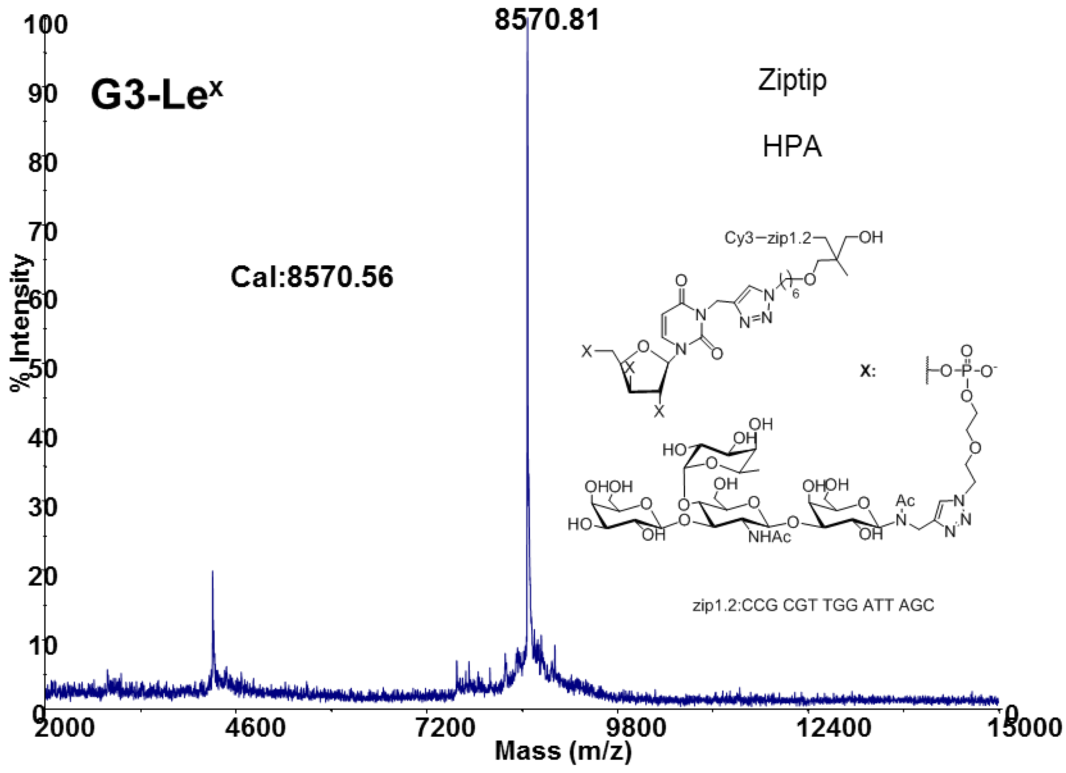
8570.81
G3-Lex
Ziptip
HPA
Cal:8570.56
% Intensity
Mass (m/z)
2000
4600
7200
9800
12400
15000
Cy3−zip1.2
X:
zip1.2:CCG CGT TGG ATT AGC

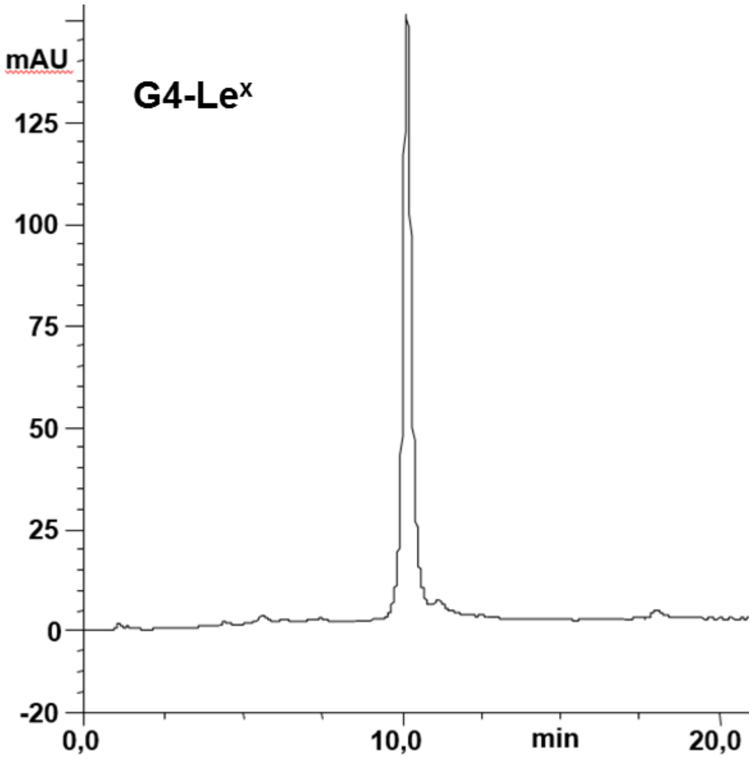
mAU
G4-Lex
125
100
75
50
25
0
-20
0,0
10,0
min
20,0

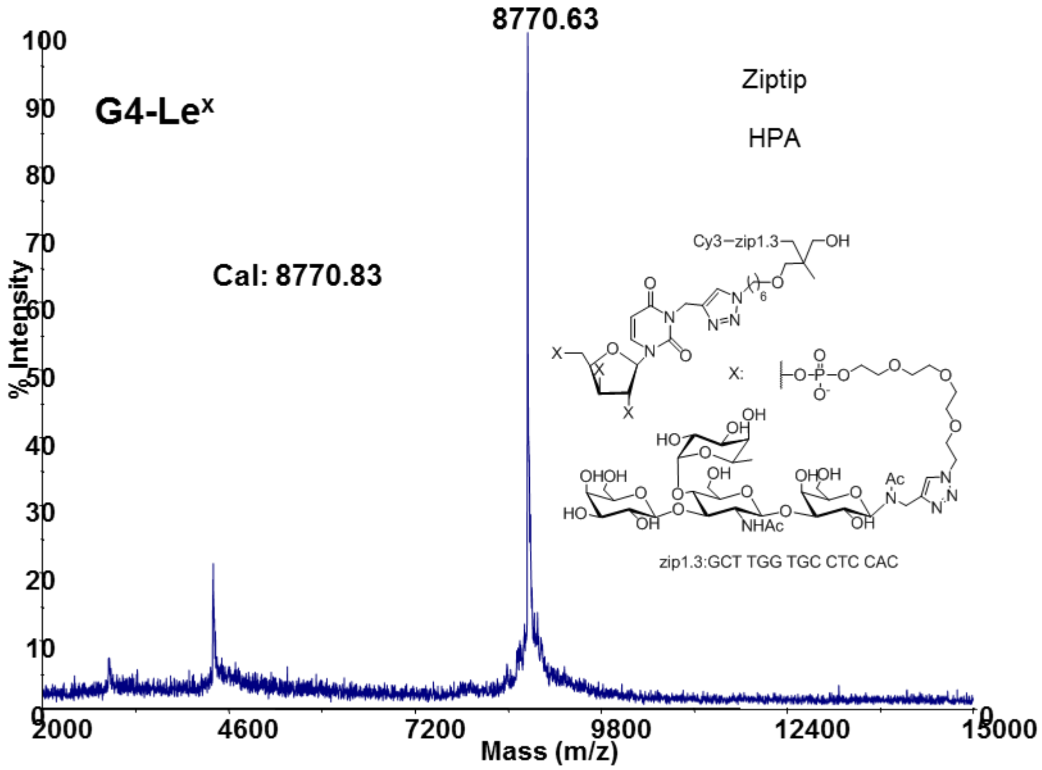
8770.63
G4-Lex
Ziptip
HPA
Cal: 8770.83
% Intensity
Mass (m/z)
2000
4600
7200
9800
12400
15000
Cy3−zip1.3
zip1.3:GCT TGG TGC CTC CAC

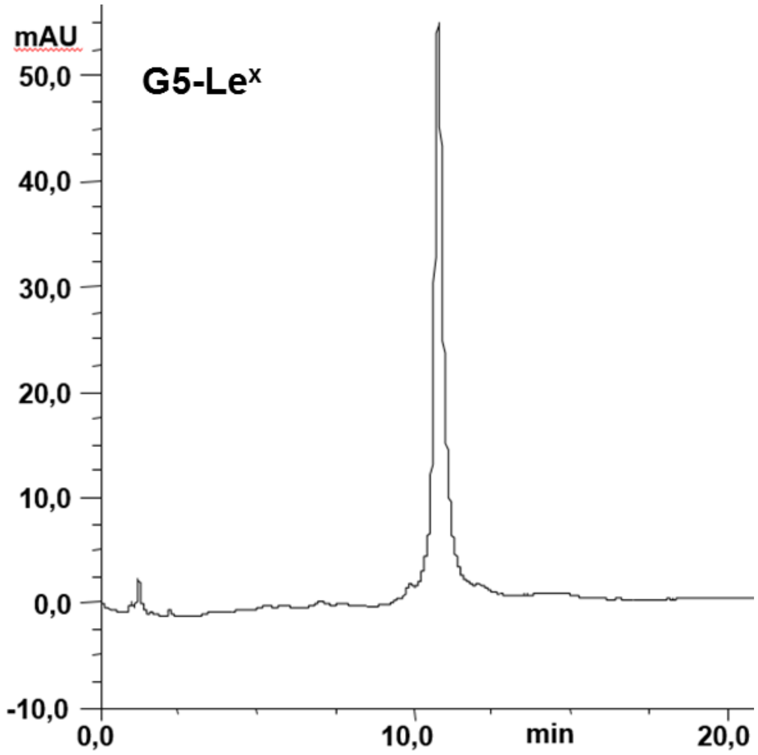
mAU
G5-Le^x
50,0
40,0
30,0
20,0
10,0
0,0
-10,0
0,0
10,0
min
20,0

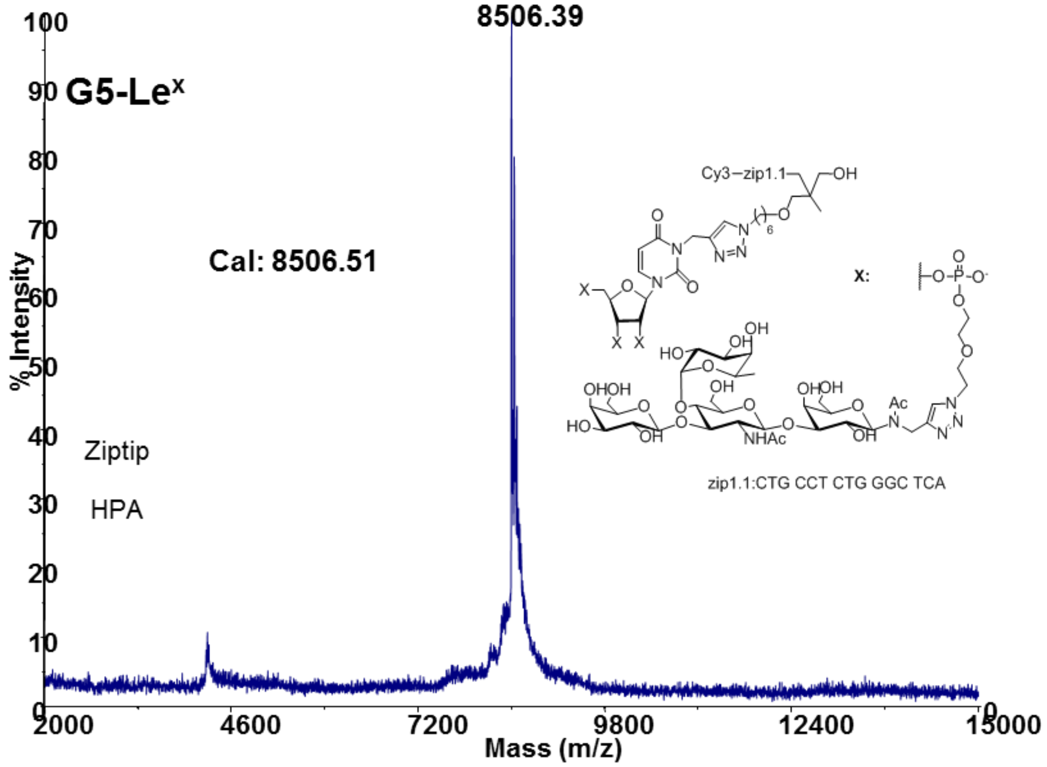
8506.39
G5-Le^x
Cal: 8506.51
Ziptip
HPA
% Intensity
Mass (m/z)
2000
4600
7200
9800
12400
15000
Cy3–zip1.1
zip1.1:CTG CCT CTG GGC TCA

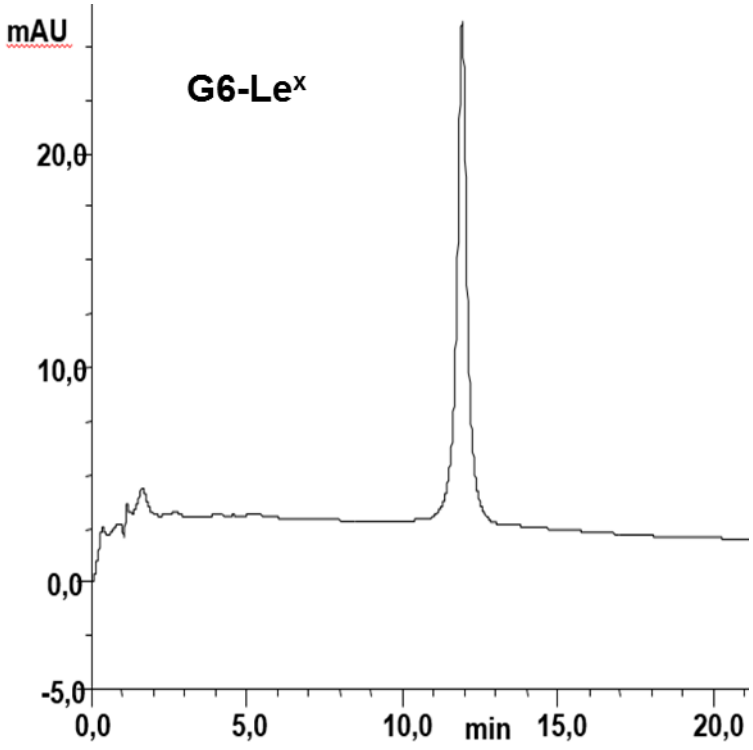

mAU
G6-Le^x
20,0
10,0
0,0
-5,0
0,0
5,0
10,0
min
15,0
20,0

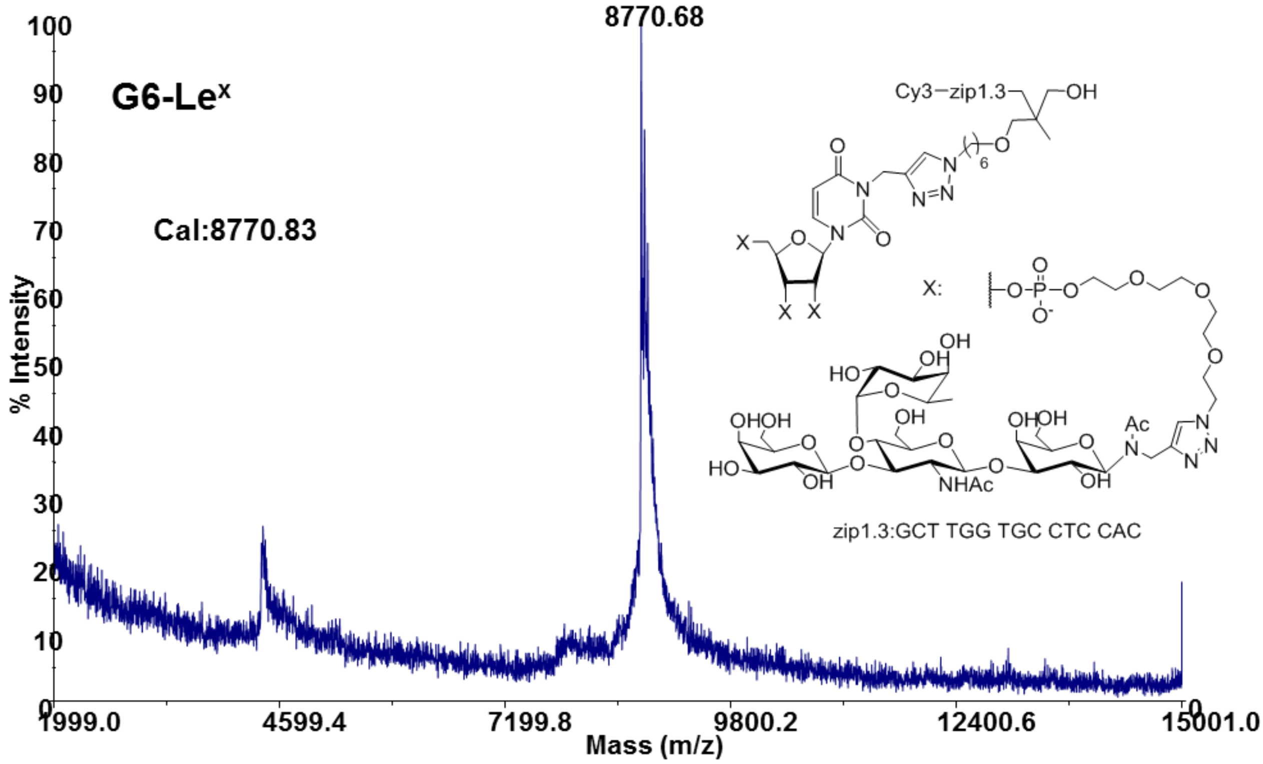

8770.68
G6-Le^x
Cal:8770.83
% Intensity
Mass (m/z)
1999.0
4599.4
7199.8
9800.2
12400.6
15001.0
Cy3—zip1.3
X:
zip1.3:GCT TGG TGC CTC CAC

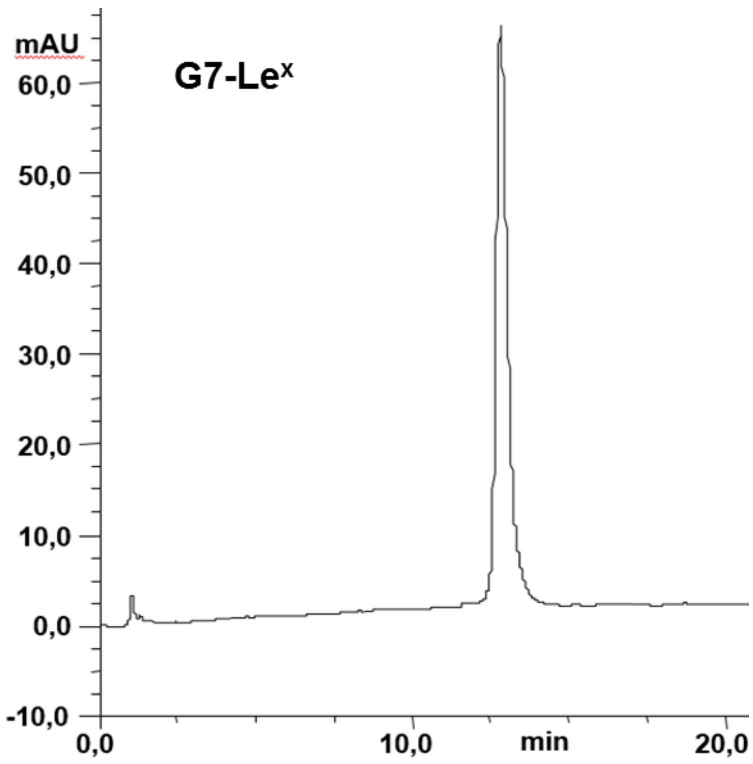
mAU
G7-Le^x
60,0
50,0
40,0
30,0
20,0
10,0
0,0
-10,0
0,0
10,0
min
20,0

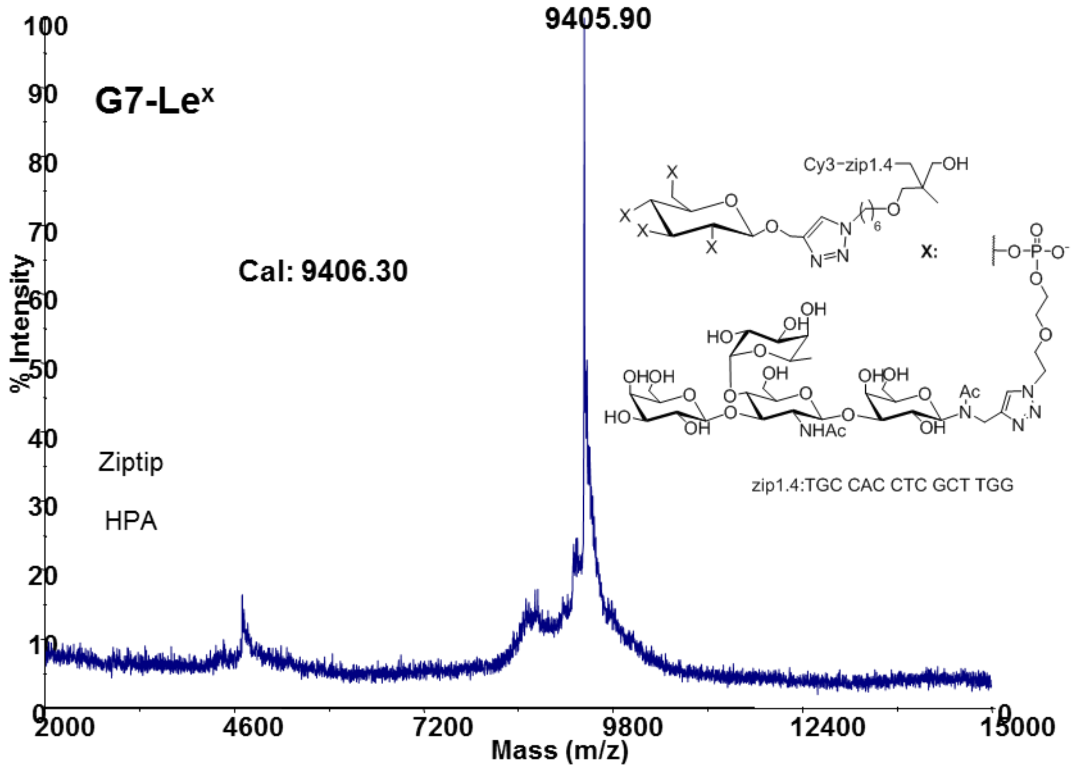
9405.90
G7-Le^x
Cal: 9406.30
Ziptip
HPA
% Intensity
Mass (m/z)
2000
4600
7200
9800
12400
15000
Cy3-zip1.4
OH
X:
zip1.4:TGC CAC CTC GCT TGG

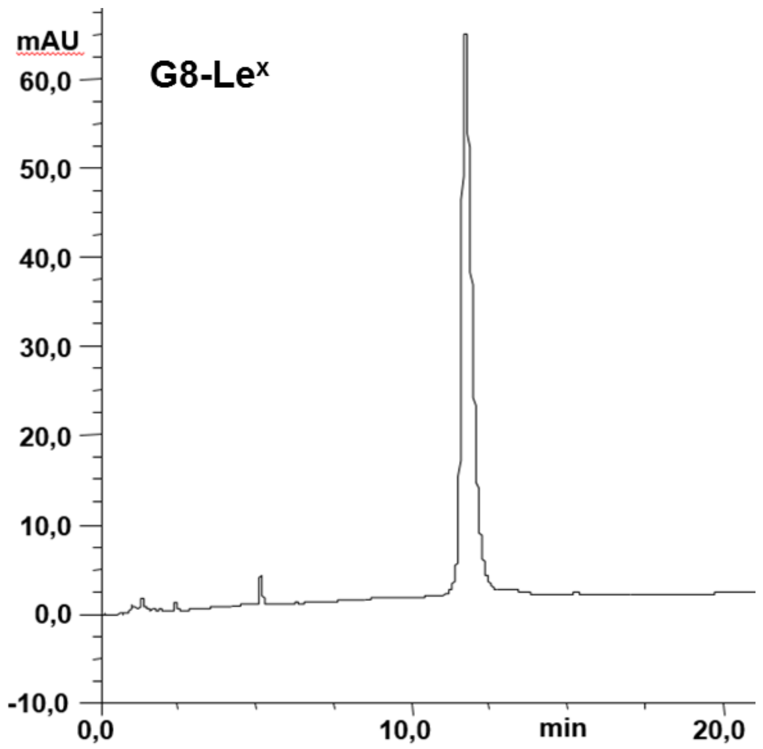
mAU
G8-Le^x
60,0
50,0
40,0
30,0
20,0
10,0
0,0
-10,0
0,0
10,0
min
20,0

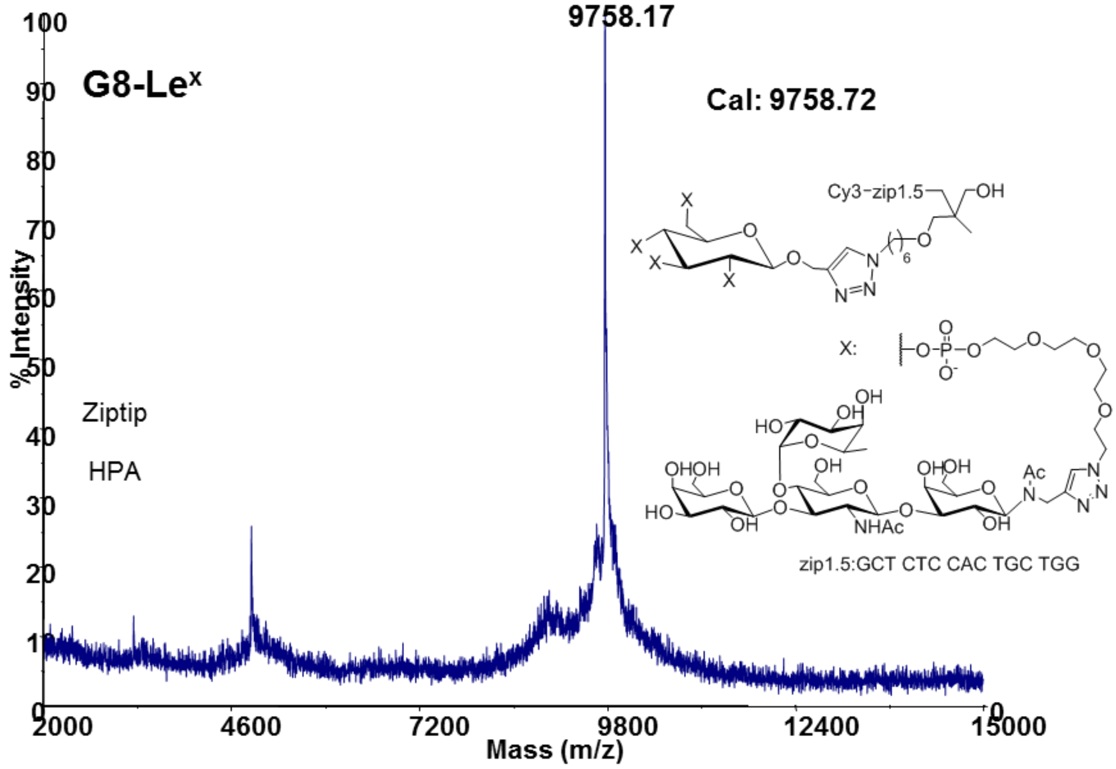
9758.17
G8-Le^x
Cal: 9758.72
% Intensity
Ziptip
HPA
Cy3-zip1.5
OH
X:
zip1.5:GCT CTC CAC TGC TGG
Mass (m/z)
2000
4600
7200
9800
12400
15000

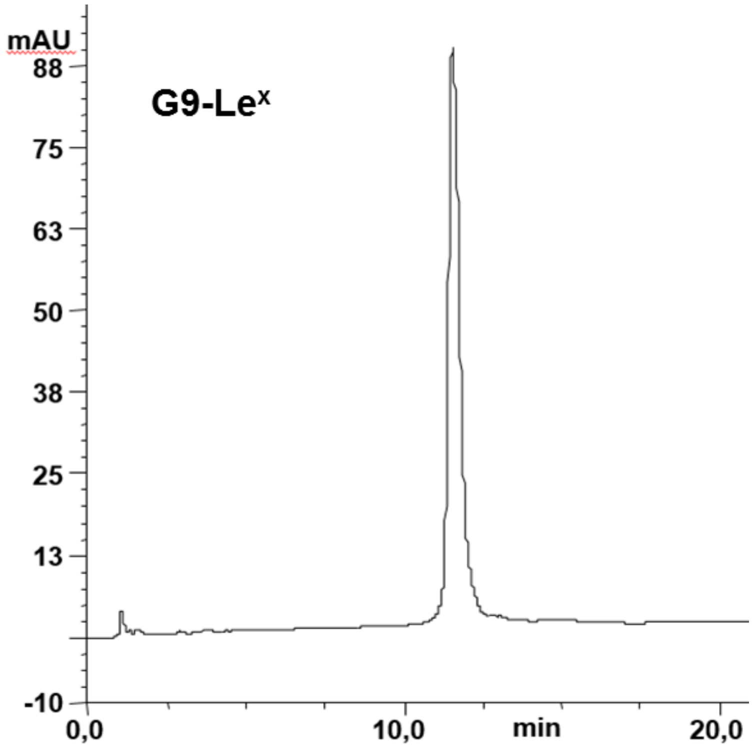

mAU
88
75
63
50
38
25
13
-10
0,0
10,0
min
20,0
G9-Le^x

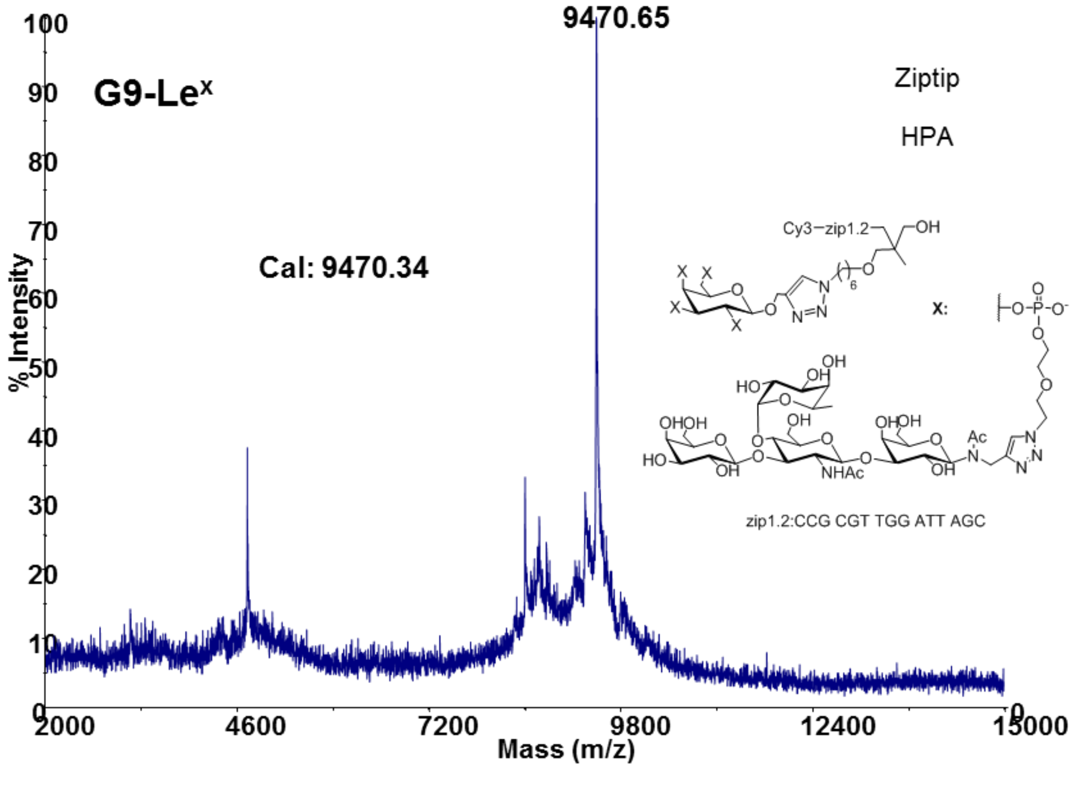

9470.65
G9-Le^x
Cal: 9470.34
Ziptip
HPA
Cy3−zip1.2
zip1.2:CCG CGT TGG ATT AGC
% Intensity
Mass (m/z)
2000
4600
7200
9800
12400
15000

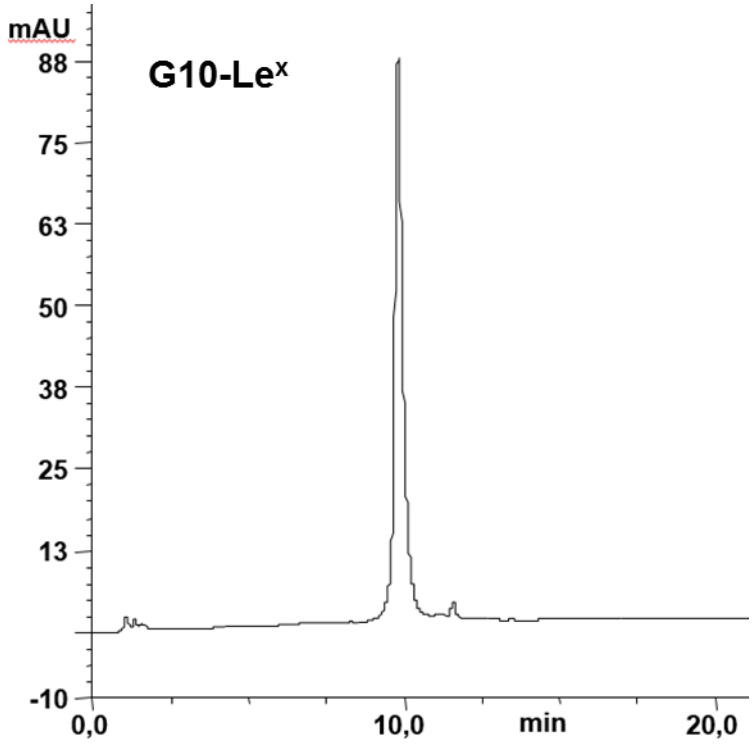
mAU
G10-Le^x
88
75
63
50
38
25
13
-10
0,0
10,0
min
20,0

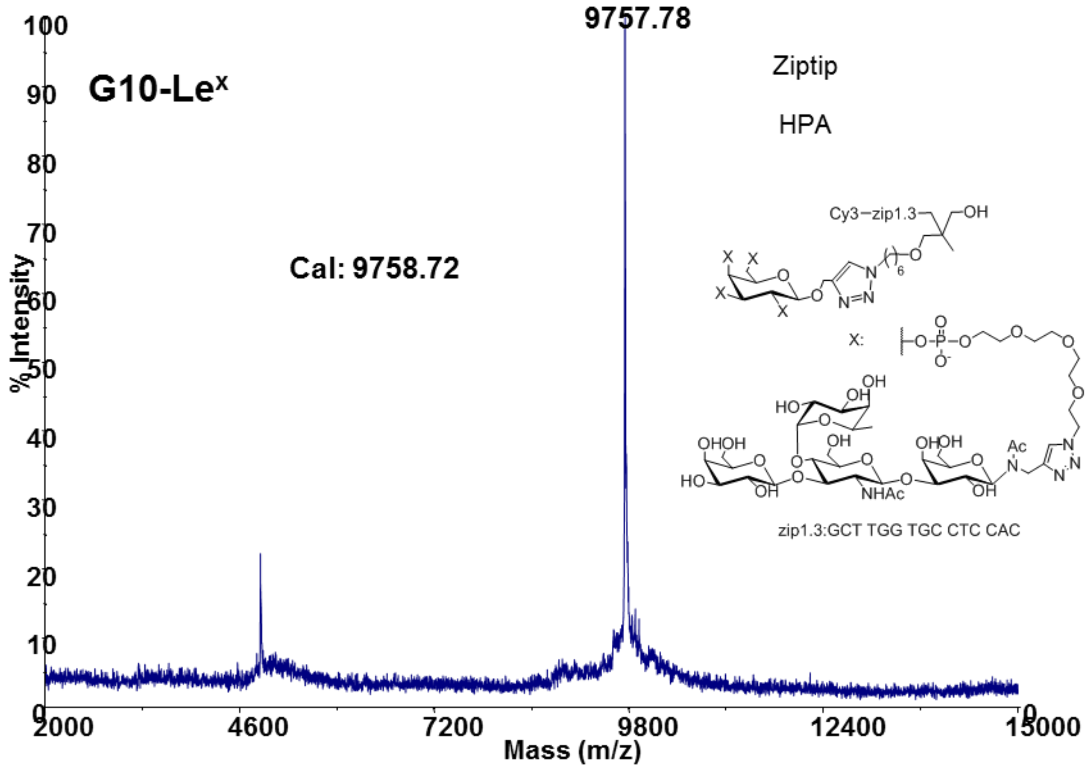
9757.78
G10-Le^x
Ziptip
HPA
Cal: 9758.72
% Intensity
Mass (m/z)
2000
4600
7200
9800
12400
15000
Cy3−zip1.3
zip1.3:GCT TGG TGC CTC CAC

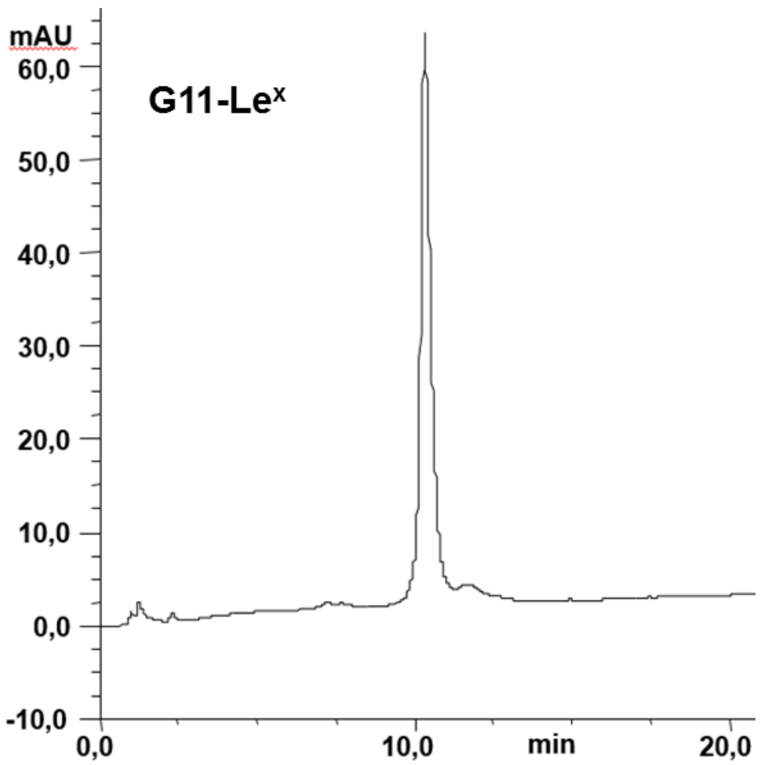
mAU
G11-Le^x
60,0
50,0
40,0
30,0
20,0
10,0
0,0
-10,0
0,0
10,0
min
20,0

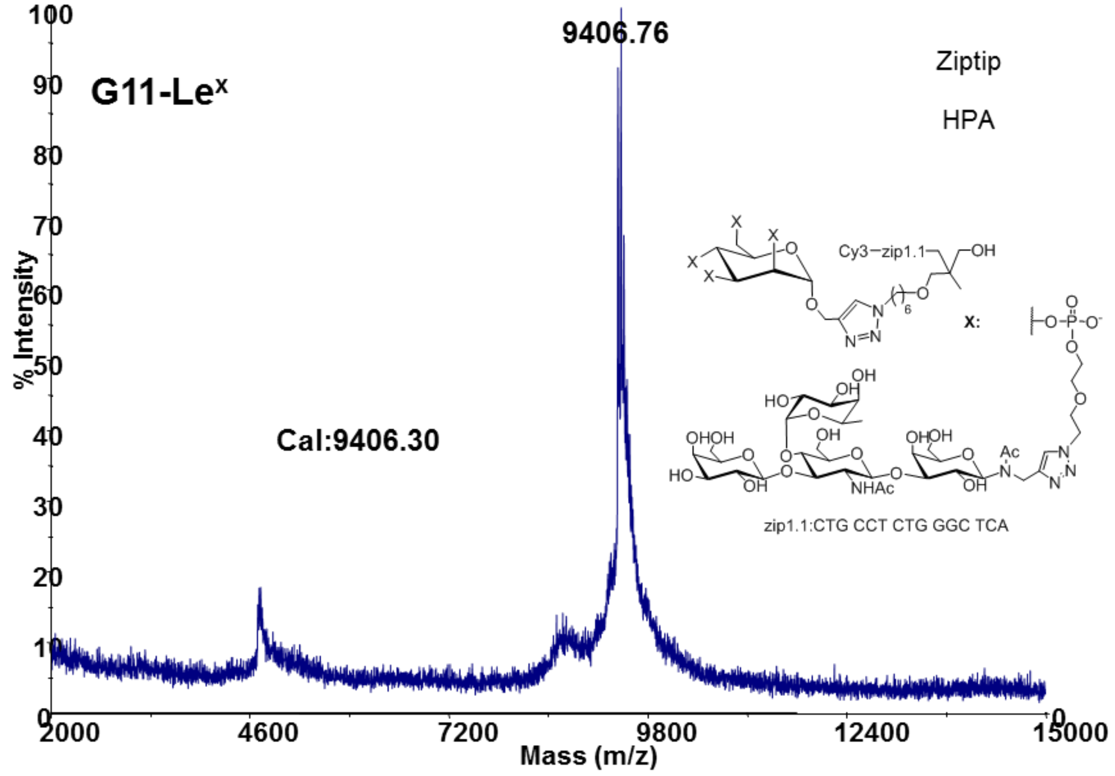
9406.76
G11-Le^x
Ziptip
HPA
Cal:9406.30
% Intensity
Mass (m/z)
2000
4600
7200
9800
12400
15000
Cy3–zip1.1
OH
X:
NHAc
zip1.1:CTG CCT CTG GGC TCA

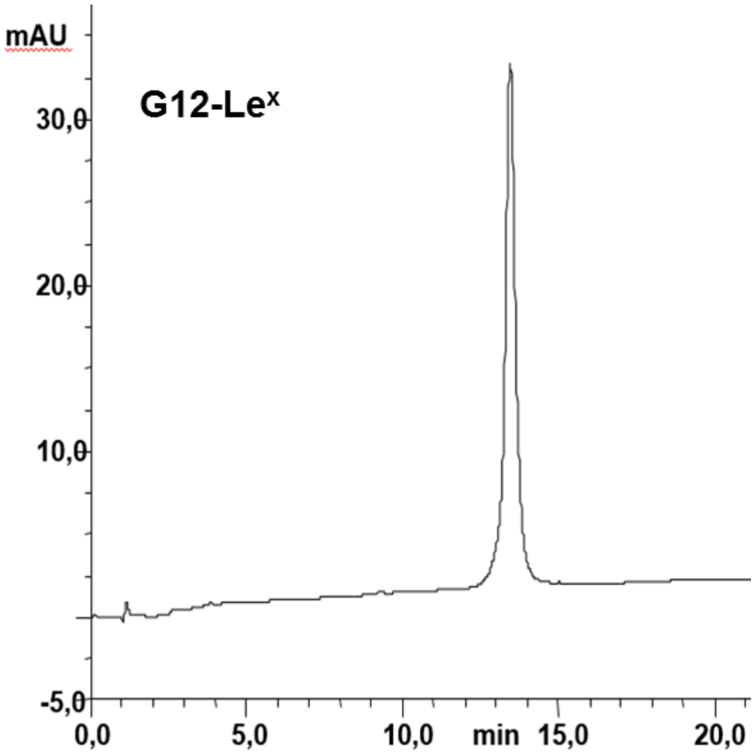

mAU
G12-Le^x
30,0
20,0
10,0
-5,0
0,0
5,0
10,0
min
15,0
20,0

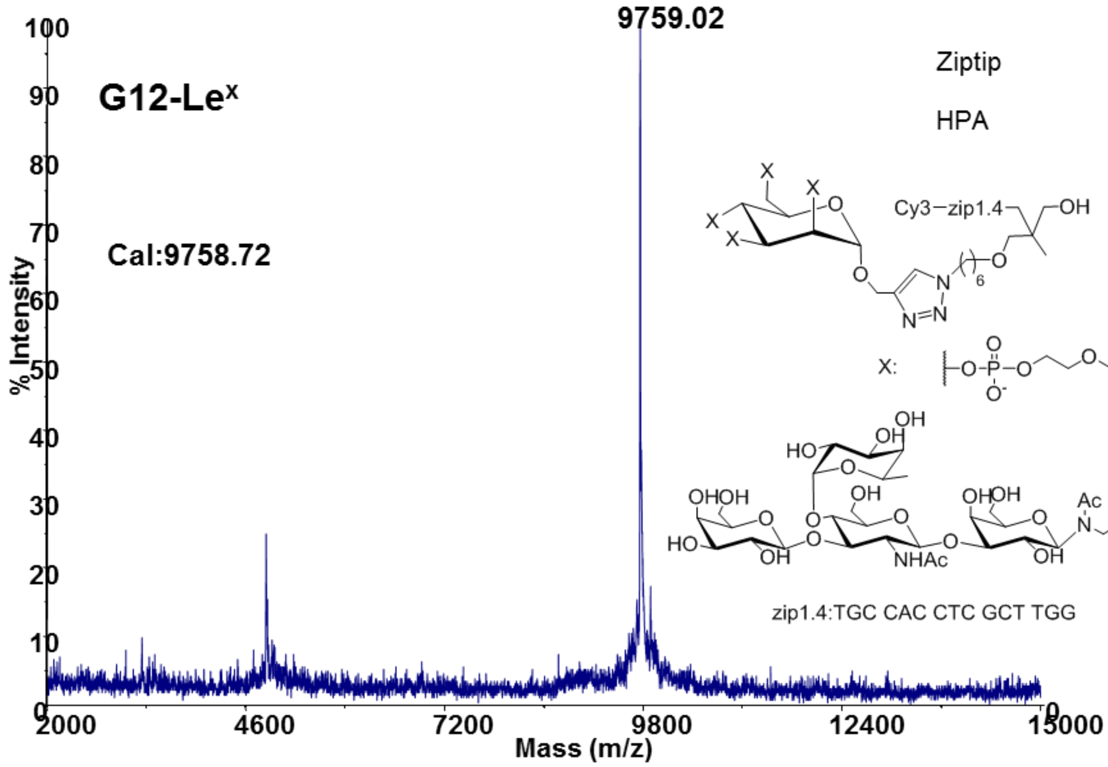

9759.02
G12-Le^x
Cal:9758.72
Ziptip
HPA
Cy3−zip1.4
OH
X:
NHAc
zip1.4:TGC CAC CTC GCT TGG
% Intensity
Mass (m/z)
2000
4600
7200
9800
12400
15000

S10: HPLC chromatograms and MALDI-ToF spectra of oligoglycoclusters with Sialyl Lewis^x

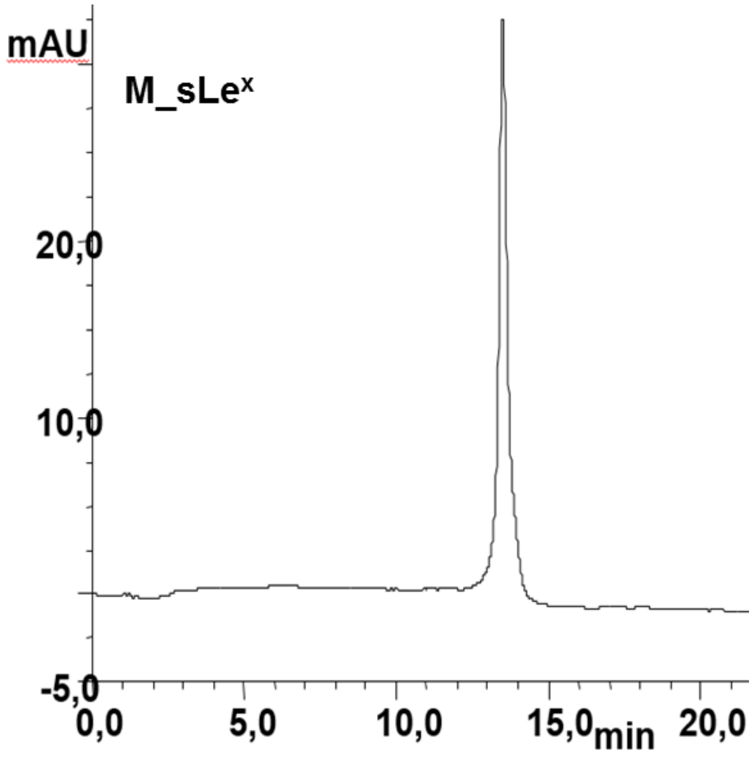

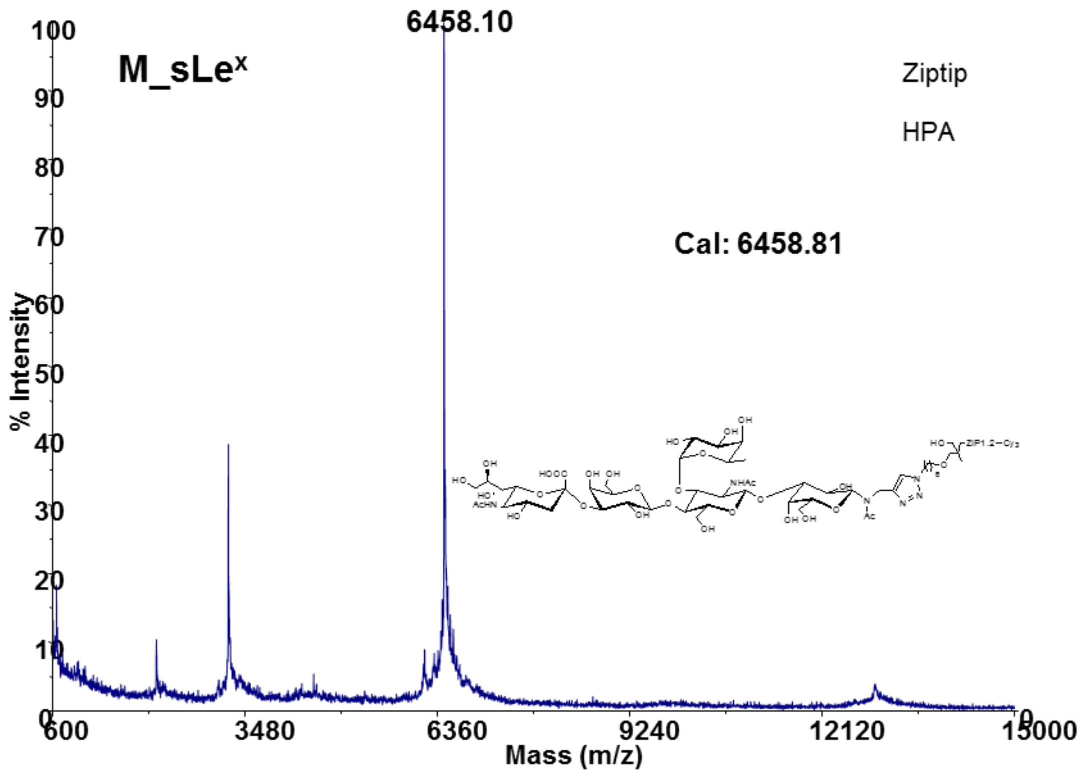

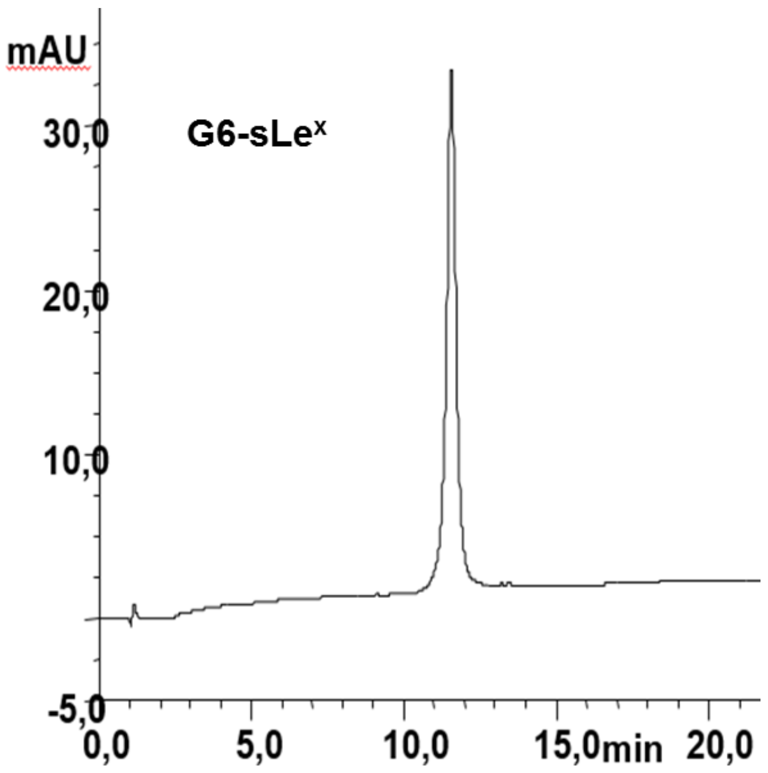
mAU
30,0
20,0
10,0
-5,0
G6-sLe^x
0,0
5,0
10,0
15,0
min
20,0

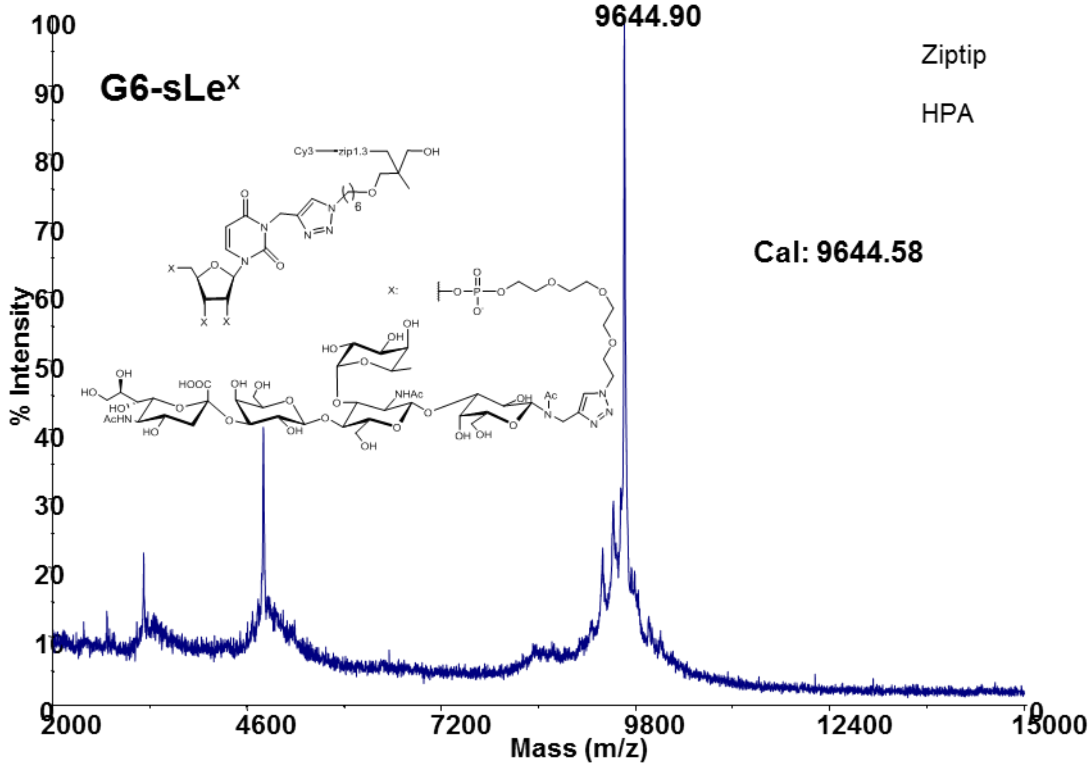
9644.90
Ziptip
HPA
G6-sLe^x
Cal: 9644.58
% Intensity
Mass (m/z)
100
90
80
70
60
50
40
30
20
10
0
2000
4600
7200
9800
12400
15000
Cy3
zip1.3
OH
X:

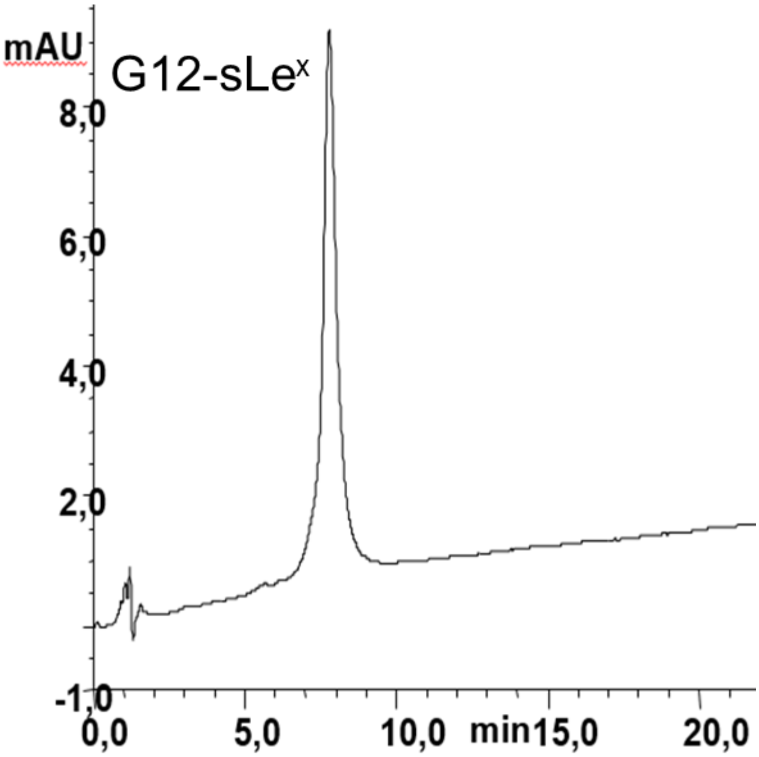
mAU
G12-sLe^{x}
8,0
6,0
4,0
2,0
-1,0
0,0
5,0
10,0
min
15,0
20,0

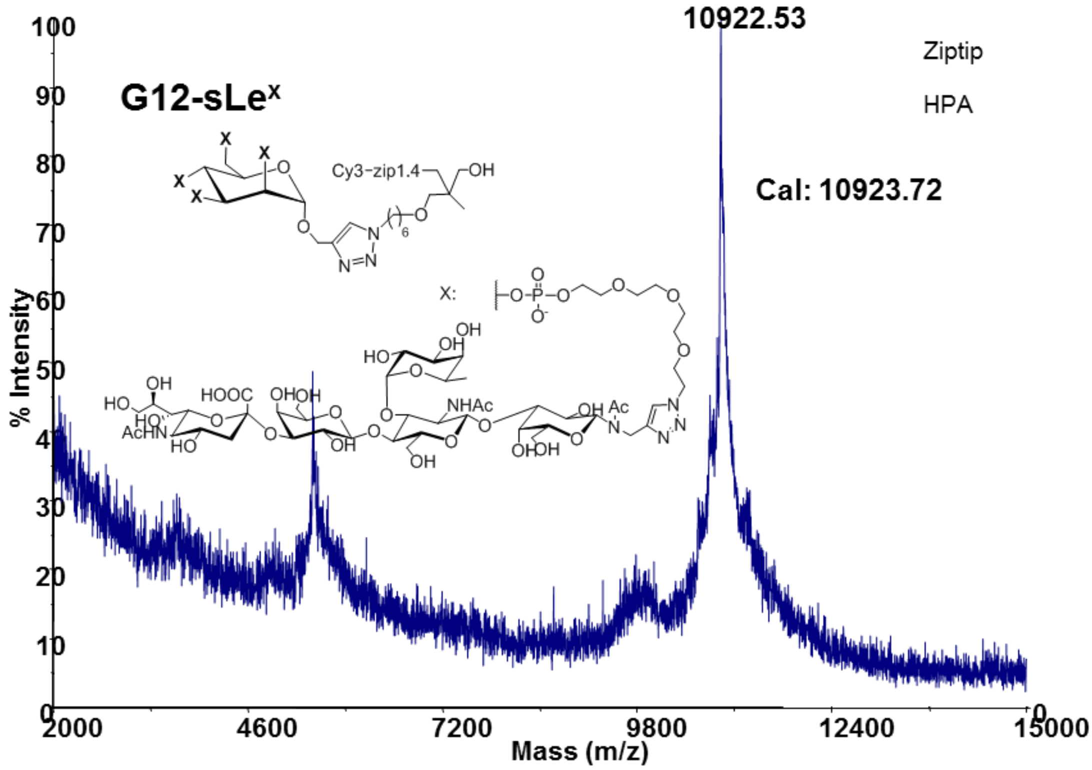
10922.53
Ziptip
HPA
Cal: 10923.72
G12-sLe^{x}
Cy3-zip1.4
OH
X:
% Intensity
Mass (m/z)
100
90
80
70
60
50
40
30
20
10
0
2000
4600
7200
9800
12400
15000
